# Kinetic Modeling of Vitamin C Degradation for Predicting Shelf Life in Tropical Juices Made from Camu Camu and Naranjilla Under Accelerated Storage Conditions

**DOI:** 10.3390/foods15101722

**Published:** 2026-05-14

**Authors:** Frank Fernandez-Rosillo, Diner Mori-Mestanza, Aleida Soledad Cabrejos-Barrios, Marleni Medina-Mendoza, Eliana Milagros Cabrejos-Barrios, Noemí León-Roque, Ernesto Hernández-Martínez, Ralph Rivera-Botonares, Efraín M. Castro-Alayo, Hans Minchán-Velayarce, César R. Balcázar-Zumaeta

**Affiliations:** 1 Grupo de Modelamiento y Simulación de Procesos en la Industria Alimentaria (MOSIPRIA), Instituto de Investigación de Ciencia de Datos (INSCID), Universidad Nacional de Jaén (UNJ), Carretera Jaén—San Ignacio KM 24, Cajamarca 06801, Peru; frank_fernandez@unj.edu.pe (F.F.-R.); acabrejosb@gmail.com (A.S.C.-B.); eliana_cabrejos@unj.edu.pe (E.M.C.-B.); ernesto.hernandez@unj.edu.pe (E.H.-M.); ralph_rivera@unj.edu.pe (R.R.-B.); hans.minchan@unj.edu.pe (H.M.-V.); 2 Instituto de Investigación, Innovación y Desarrollo para el Sector Agrario y Agroindustrial (IIDAA), Facultad de Ingeniería y Ciencias Agrarias, Universidad Nacional Toribio Rodríguez de Mendoza de Amazonas, Chachapoyas 01001, Perumarleni.medina@untrm.edu.pe (M.M.-M.); efrain.castro@untrm.edu.pe (E.M.C.-A.); 3 Programa de Doctorado en Ingeniería de Alimentos, Escuela de Posgrado, Universidad Nacional del Santa (UNS), Urb. Av. Universitaria s/n, Chimbote 02712, Peru; 4 Facultad de Ingeniería Química e Industrias Alimentarias, Universidad Nacional Pedro Ruiz Gallo, Lambayeque 14013, Peru; nleonr@unprg.edu.pe

**Keywords:** ascorbic acid, camu camu, degradation kinetics, naranjilla, shelf-life, tropical juices

## Abstract

The objective of this study was to predict the degradation kinetics of vitamin C in camu camu and naranjilla juices using accelerated storage tests. The juices were produced under controlled processing conditions, including physicochemical standardization, pasteurization, and hot filling into glass containers. They were then stored at 35, 45, and 55 °C for 21, 14, and 7 days, respectively. The vitamin C content was quantified using high-performance liquid chromatography, showing a progressive decrease depending on temperature. The kinetic data were fitted to zero-order and first-order models, as well as to the nonlinear Weibull model, the latter presenting the best statistical fit (R^2^ = 0.9678–0.9931) and adequately describing the nonlinear degradation behavior of vitamin C. Temperature dependence was modeled using the Arrhenius equation, allowing activation energy to be estimated and confirming temperature-dependent degradation behavior in both juices, with different thermal responses depending on the modeling approach. Likewise, shelf life (*t*_80_) was estimated using the Weibull model and showed a significant reduction with increasing storage temperature. Arrhenius-based shelf-life predictions suggested greater vitamin C retention in camu camu juice at lower storage temperatures, whereas naranjilla exhibited a more pronounced decrease as temperature increased. The physicochemical parameters (pH, acidity, and °Brix) showed moderate changes, maintaining the stability of the system during storage. The results confirm the applicability of the Weibull model to describe vitamin C degradation in complex matrices and highlight the importance of thermal control in the preservation of bioactive compounds in tropical juices.

## 1. Introduction

Camu camu (*Myrciaria dubia*) is a native Amazonian fruit distributed across floodplain ecosystems [1], where it has gained increasing attention due to its exceptional nutritional value and potential application in functional beverage formulations [2,3,4,5,6]. This species is recognized as one of the richest natural sources of vitamin C reported among edible fruits, with concentrations typically ranging from approximately 1200 to 3000 mg of vitamin C per 100 g of fresh pulp, depending on genotype, ripening stage, and environmental conditions [7,8]. Reported average values of approximately 1800–2000 mg 100 g^−1^ fresh pulp are commonly observed under commercial maturity conditions [9,10]. In addition to its high vitamin C content, camu camu pulp contains significant levels of phenolic compounds, carotenoids such as β-carotene [11], essential minerals [12] and amino acids that contribute to its antioxidant capacity and functional properties [2,13]. These compositional attributes position camu camu as a promising raw material for the development of nutritionally enriched beverages [5,14].

Naranjilla (*Solanum quitoense* Lam.), also known as lulo, is a tropical Andean fruit widely cultivated [15], highly valued for its intense aroma, refreshing acidic flavor, and suitability for beverages and juice production [16]. From a nutritional standpoint, naranjilla pulp represents an important source of bioactive compounds, including vitamin C, carotenoids, phenolic acids, and essential minerals such as potassium, calcium, and magnesium [15,17]. Reported vitamin C contents in fresh pulp typically range from 11 to 95 mg/100 g, depending on genotype, maturity stage, and cultivation conditions, with values commonly between 30 and 50 mg/100 g in commercially available Andean fruits [18]. This compositional variability highlights the potential of naranjilla as a promising raw material for functional beverage development and for investigating vitamin C stability during accelerated thermal storage, particularly in comparison with conventional tropical fruit juices.

From a technological and nutritional perspective, these tropical fruits represent two distinct yet complementary systems for studying the degradation kinetics of vitamin C during storage [19,20]. While camu camu is characterized by exceptionally high initial concentrations of vitamin C [21], among the highest recorded in edible fruits worldwide, naranjilla has moderate but nutritionally relevant levels [17], comparable to those of conventional citrus juices. This compositional contrast provides a valuable framework for evaluating how the initial concentration of vitamin C, the matrix composition, and antioxidant co-constituents influence degradation pathways under accelerated thermal conditions. Consequently, comparing these two tropical juice systems allows for a more robust evaluation of nonlinear kinetic approaches, such as the Weibull model, to describe nutrient stability and predict shelf life in functional beverages subjected to accelerated storage testing.

Vitamin C (ascorbic acid) is a water-soluble micronutrient naturally present in a wide variety of fruits and represents one of the most important dietary antioxidants [22,23,24], as it is an essential nutrient that humans cannot synthesize endogenously [25]. However, vitamin C is also recognized as one of the most unstable vitamins in food systems, since its degradation is strongly influenced by oxygen exposure, temperature, light, pH, metal ions, and the presence of reducing sugars during processing and storage [20,24,26]. In tropical fruit juices, these reactions are frequently associated with non-enzymatic browning pathways involving interactions between vitamin C, amino acids, and carbohydrates, leading to progressive losses in nutritional quality and sensory acceptability [26,27,28]. Consequently, the retention of vitamin C has become a critical quality indicator for the fruit juice industry, particularly under thermal treatments such as pasteurization that are required to ensure microbiological safety but may accelerate nutrient degradation [29]. Moreover, variations in titratable acidity, soluble solids, and color observed during storage are often directly linked to vitamin C oxidation kinetics, reinforcing the importance of accurately determining degradation rates for shelf-life prediction [30,31,32]. Given the growing global demand for tropical fruit beverages with preserved functional value, the development of reliable kinetic models capable of describing vitamin C stability under accelerated storage conditions remains essential for optimizing processing strategies and improving nutritional quality retention in commercial juice systems.

Accelerated shelf-life testing has been widely applied to estimate nutrient retention in fruit-based beverages; however, most kinetic studies on citrus and tropical juices have traditionally relied on zero- or first-order reaction models to describe vitamin degradation during storage [33,34,35,36,37,38,39]. In particular, the degradation of vitamin C in fruit matrices such as orange juice and thermally processed plant products has frequently been modeled using pseudo-first-order kinetics combined with Arrhenius-type temperature dependence to estimate reaction rate constants and activation energies [40,41,42]. Although this classical framework has proven useful for shelf-life prediction under isothermal conditions, it assumes that degradation reactions can be adequately represented by a single kinetic constant and a fixed reaction order, which may not fully reflect the complexity of real food systems [43]. For this reason, alternative nonlinear approaches, including Weibull-type formulations, have been proposed as more flexible tools to describe nutrient degradation patterns in complex matrices [44,45,46]. However, the integration of Weibull modeling with Arrhenius-type temperature dependence has been only sparingly explored for predicting vitamin C stability in tropical fruit juices such as camu camu and naranjilla. Therefore, the present study aimed to evaluate the applicability of an integrated Weibull–Arrhenius modeling framework to describe vitamin C degradation kinetics and predict shelf life in these two contrasting tropical juice systems under accelerated storage conditions.

## 2. Materials and Methods

### 2.1. Raw Materials

Fresh camu camu fruits were harvested in Yurimaguas, Loreto, Peru, while naranjilla fruits were collected in Moyobamba, San Martín, Peru, in July 2025. Fruits were selected at commercial maturity based on external color uniformity, absence of mechanical damage, and soluble solids content consistent with reported maturity ranges for each species (approximately 10–11 °Brix for camu camu and 9–11 °Brix for naranjilla). After harvest, fruits were transported under ambient conditions to the Food Technology Laboratory of the Universidad Nacional de Jaén, Peru, and processed within 24 h. Prior to processing, fruits were stored at 4 °C and manually sorted to remove defective samples.

### 2.2. Juice Processing

For each fruit matrix, a single production batch of standardized juice was prepared under controlled laboratory conditions (Figure S1). Selected fruits were washed with potable water and sanitized by immersion in an aqueous chlorine dioxide solution (Ecolab Inc., St. Paul, MN, USA) (1 mL/L) for 5 min under gentle agitation to reduce surface microbial load prior to processing. Camu camu fruits were mechanically homogenized using an industrial blender (LAR-25, SKYMSEN Ltd., Brusque, SC, Brazil), whereas naranjilla pulp was first obtained using a fruit pulper (DFA-50B, BOXA Industrial S.A.C., Lima, Peru) and subsequently homogenized in the same blender. The resulting suspensions were filtered through a 1 mm stainless-steel mesh to remove seeds and coarse fibrous material. The filtered pulps were diluted with potable water at ratios of 3 L pulp:18 L water for camu camu and 4 L pulp:16 L water for naranjilla. Initial physicochemical standardization of the juices was performed using a benchtop pH meter (HI6221, Hanna Instruments, Woonsocket, RI, USA) and a digital refractometer (MA871, Milwaukee Instruments, Rocky Mount, NC, USA). Food-grade sucrose (Paramonga S.A.A., Lima, Peru) was incorporated as a soluble solid’s adjuster (2.1 kg for camu camu; 1.55 kg for naranjilla), together with carboxymethyl cellulose (0.05%, *w*/*w*) as stabilizer and potassium sorbate (0.03%, *w*/*w*) as antimicrobial agent. Final formulations were standardized to 10 °Brix and target pH values of 3.22 (camu camu) and 3.65 (naranjilla) prior to pasteurization. However, after thermal processing and hot filling, slight variations in both pH and soluble solids were observed due to physicochemical changes induced by heating. Therefore, the values reported at the beginning of accelerated storage correspond to the actual physicochemical conditions of the bottled samples rather than the nominal formulation targets. Juices were pasteurized at 87 °C for 10 min, hot-filled (85 °C) into sterilized 250 mL glass bottles with twist-off caps and rapidly cooled by immersion in cold water to promote vacuum formation. The final batches consisted of 75 bottles of juice for each variety (camu camu and naranjilla).

### 2.3. Accelerated Storage Conditions

The accelerated storage experiments were conducted using three temperature-controlled laboratory incubators (GX45BE, FAITHFUL Instrument Co., Ltd., Ningbo, China), with a temperature stability of ±1 °C according to manufacturer specification. The samples were stored at 35 °C for 21 days (sampling every 3 days), 45 °C for 14 days (sampling every 2 days), and 55 °C for 7 days (daily sampling). Temperature conditions were monitored throughout storage using the incubator’s internal digital control system. Sampling intervals were selected following the principles of accelerated shelf-life testing to obtain degradation datasets suitable for kinetic modeling and Arrhenius analysis. All analyses were performed in triplicate.

Juice samples were stored in hermetically sealed glass containers with minimal headspace in order to reduce oxygen exchange with the surrounding environment during accelerated storage. Although dissolved oxygen levels were not directly monitored, the use of glass containers with very low oxygen permeability helped minimize external oxygen diffusion and ensured consistent storage conditions across all temperature treatments.

### 2.4. Determination of Physicochemical Parameters

#### 2.4.1. pH Measurement

pH was determined according to AOAC Method 981.12 [47] using a digital pH meter (PH700, Apera Instruments, Shanghai, China). Juice samples were diluted (10 mL sample:100 mL distilled water), homogenized, and measured at room temperature. Measurements were performed in triplicate for each storage condition.

#### 2.4.2. Total Soluble Solids (°Brix)

Total soluble solids were measured using a digital refractometer (MA871, Milwaukee Instruments, Rocky Mount, NC, USA) calibrated at 20 °C according to standard refractometric procedures described by Rangana [48]. Diluted samples (10 mL sample:100 mL distilled water) were analyzed in triplicate. Results were expressed as °Brix.

#### 2.4.3. Titratable Acidity

Titratable acidity was determined by acid–base titration following AOAC Method 942.15 [47] and standard procedures described by Nielsen [49]. Diluted samples (10 mL juice in 100 mL distilled water) were titrated with 0.1 N NaOH using phenolphthalein as indicator until a persistent pale pink coloration appeared. Titratable acidity was expressed as g citric acid per 100 mL (Equation (1)). All determinations were performed in triplicate.

(1) $$\%\;\mathrm{Titratable}\;\mathrm{acidity}\;(g\;\mathrm{citric}\;\mathrm{acid}\;\mathrm{per}\;100\;\mathrm{mL})=\frac{V\times N\times 64.04}{Sample\;Volume}\times 100$$

where *V* is NaOH volume (mL), *N* is NaOH normality (0.1 N), 64.04 is equivalent weight of citric acid (g eq^−1^) and *Sample Volume* is volume of juice sample (mL).

### 2.5. HPLC Quantification of Vitamin C

Vitamin C content was determined by high-performance liquid chromatography (HPLC) following the method described by Oruña-Concha [50], with minor modifications adapted for tropical fruit juice matrices.

#### 2.5.1. Sample Preparation

Juice samples were homogenized prior to analysis. For naranjilla juice, 15 mL aliquots were transferred into centrifuge tubes and centrifuged at 6000 rpm for 5 min (ISOLAB Laborgeräte GmbH, Eschau, Germany). The supernatant was filtered through 0.22 µm syringe filters (nylon membrane) before chromatographic injection. Due to the high vitamin C concentration of camu camu juice, samples were diluted with ultrapure water at a ratio of 1:3 (*v*/*v*) prior to filtration. All sample preparations were performed in triplicate under reduced light exposure to minimize oxidative degradation.

#### 2.5.2. Preparation of Calibration Curve

Quantification was performed using an external standard calibration method based on Doner & Hicks [51], with modifications. A stock solution of L-ascorbic acid standard (Sigma-Aldrich, St. Louis, MO, USA) (2000 mg/L) was prepared in ultrapure water and used to obtain working standards at concentrations of 1000, 500, 250, 125, 50 and 25 mg/L. Calibration curves were constructed by plotting peak area versus concentration. Linearity of the detector response was verified prior to sample analysis (R^2^ > 0.999).

#### 2.5.3. Chromatographic Conditions

Chromatographic analyses were carried out using an HPLC system (DGU-20A3R, Shimadzu Corporation, Kyoto, Japan) equipped with an autosampler (SIL-30AC), column oven (CTO-20AC), and diode-array detector (SPD-M30A). Separation was achieved using a RESTEK Ultra C18 column (150 × 4.6 mm, 5 µm particle size; Restek Corporation, Bellefonte, PA, USA). The mobile phase consisted of 0.05 M phosphoric acid (Merck KGaA, Darmstadt, Germany) under isocratic elution conditions at a flow rate of 1.32 mL/min. The column temperature was maintained at 30 °C and the injection volume was set at 1 µL. This low injection volume was intentionally selected to avoid detector saturation due to the high vitamin C concentration of the juice samples, particularly camu camu, and to maintain the chromatographic response within the linear range of the external calibration curve. Detection was performed at 245 nm. Quantification of vitamin C was carried out by external calibration using peak area integration. All analyses were performed in triplicate, and results were expressed as mg ascorbic acid per 100 mL of juice. The chromatographic identification and quantification reliability were supported by the retention time consistency observed in the reference standard and sample chromatograms (Files S1 and S2).

Method performance was evaluated in terms of linearity, limits of detection (LOD), limits of quantification (LOQ), recovery, and repeatability. The LOD and LOQ were estimated from the calibration curve as signal-to-noise ratios of 3 and 10, respectively. The calculated LOD and LOQ values were 3.8 mg/L and 12.6 mg/L, respectively. Method precision, expressed as relative standard deviation (RSD), was below 2.5% for replicate injections (*n* = 3). Recovery tests performed by spiking known amounts of L-ascorbic acid into juice samples yielded values between 96.2% and 103.4%, confirming the accuracy of the method for vitamin C determination in tropical juice matrices.

### 2.6. Kinetic Modeling and Arrhenius Analysis

The degradation kinetics of vitamin C during storage were evaluated using both classical reaction-order models (zero- and first-order kinetics) and a nonlinear Weibull model to describe the temperature-dependent behavior of vitamin C loss in tropical juice matrices. Vitamin C concentration was expressed as C (mg/100 mL) for kinetic modeling purposes.

#### 2.6.1. Determination of Reaction Order

Zero-order and first-order kinetic models were tested to describe the degradation of vitamin C during storage according to the following:

Zero-order model:

(2) $$\frac{d\left[C_{t}\right]}{dt}=-k$$

whose integrated form is

(3) $$C_{t}-C_{0}=-k_{1}t$$

First-order model:

(4) $$\frac{d\left[C_{t}\right]}{dt}=-k\left[C_{t}\right]$$

with integrated expression

(5) $$ln\left[C_{t}\right]=ln\left[C_{0}\right]-k_{2}t$$

where $C_{t}$ is the vitamin C concentration at time t, $C_{0}$ is the initial concentration, $k_{1}$ and $k_{2}$ are degradation rate constants, and *t* is storage time (days).

Linear regression analyses were performed by plotting C versus t (zero-order) and lnC versus t (first-order). The most appropriate kinetic model was selected based on the highest coefficient of determination ($R^{2}$) obtained at each storage temperature.

#### 2.6.2. Weibull Model

In addition to classical kinetic approaches, degradation data were fitted using the Weibull model (Equation (6)) proposed by Corradini & Peleg [45], which has demonstrated improved flexibility in describing nonlinear degradation behavior in complex food systems:

(6) $$Y(t)\;=\;\frac{C(t)\;}{C_{0}}=exp\left[-b\left(T\right)t^{n\left(T\right)}\right]$$

where *Y(t)* is the normalized vitamin C concentration, *C*(*t*) is the concentration of vitamin C at time t, $C_{0}$ is initial concentration, $b\left(T\right)$ is the scale parameter (temperature-dependent degradation rate), $n\left(T\right)$ is the shape parameter describing deviation from linear kinetics, and t is storage time (days).

The temperature dependence of parameter b(T) was described using the log-logistic relationship proposed by Corradini & Peleg [45,52]:

(7) $$b(T)\;={log}_{e}\left\{1+\exp\left[k_{3}(T-T_{c})\right]\right\}$$

where $k_{3}$ is a temperature sensitivity constant, $T_{c}$ is the characteristic temperature, and T is storage temperature (°C). Model parameters were estimated by nonlinear regression using experimental degradation data obtained at each storage condition.

#### 2.6.3. Arrhenius Analysis

The temperature dependence of the Weibull rate parameter b(T) was described using an Arrhenius-type relationship:

Arrhenius-type relationship:

(8) $$b(T)=b_{0\;}\exp\left(-\left(\frac{E_{a}}{R}\right)\frac{1}{\left(T+273.15\right)}\right)$$

whose linearized form is

(9) $$ln\left[b(T)\right]=\ln\left[b_{0\;}\right]-\left[\left(\frac{E_{a}}{R}\right)\frac{1}{\left(T+273.15\right)}\right]$$

where $b_{0}$ is the pre-exponential factor, $E_{a}$ is the activation energy (kJ/mol), R is the universal gas constant (8.314 J/mol K), and T is the storage temperature (°C) expressed in absolute temperature as T + 273.15 (K). Activation energy values were calculated from the slope of the linear regression of $\ln b\left(T\right)$ versus 1/T.

#### 2.6.4. Shelf-Life Prediction Based on Vitamin C Retention

Shelf life was estimated as the time required to reach 80% retention of the initial vitamin C concentration ($t_{80}$), which is commonly used as a nutritional acceptability threshold for fruit-based beverages:

Using the Weibull model,

(10) $$t_{80}={\left[\frac{-ln\left[0.80\right]}{b}\right]}^{1/n}$$

where $t_{80}$ is the predicted shelf life (days), *b* is the Weibull scale parameter, and *n* is the Weibull shape parameter.

Shelf-life predictions were obtained at experimental temperatures and extrapolated to lower storage temperatures using Arrhenius relationships.

### 2.7. Statistical Analysis

All physicochemical measurements were performed in triplicate and results were expressed as mean ± standard deviation. The effects of storage temperature, time, and their interaction on physicochemical parameters and vitamin C degradation were evaluated using two-way analysis of variance (ANOVA). Model significance was assessed at a confidence level of 95% (*p* < 0.05). Effect sizes were estimated using partial eta squared (η^2^), and model goodness-of-fit was evaluated through the coefficient of determination (R^2^). Pearson correlation analysis was performed to explore relationships between vitamin C retention and explanatory variables including titratable acidity, pH, total soluble solids (°Brix), temperature, and storage time.

Isothermal degradation kinetics of vitamin C were modeled using zero-order, first-order, and Weibull equations. Nonlinear regression analysis was applied to estimate the Weibull parameters b(T) and n(T) according to the temperature-dependent Weibull model proposed by Corradini & Peleg [45]. Model performance was evaluated using the coefficient of determination (R^2^), mean squared error (MSE), and root mean square error (RMSE). The temperature dependence of the Weibull rate parameter b(T) was further described using both the Arrhenius secondary model and the log-logistic relationship. Activation energy (E_a_) values were estimated from the linearized Arrhenius equation using regression of ln(b) versus reciprocal absolute temperature (1/T). Shelf-life values corresponding to 80% vitamin C retention (*t*_80_) were calculated from Weibull parameters and subsequently extrapolated to lower storage temperatures using Arrhenius-type relationships. Nonlinear kinetic modeling and parameter estimation were performed using Python (version 3.11; Python Software Foundation, Wilmington, DE, USA) with least-squares optimization routines implemented in the SciPy library (version 1.11.4). Statistical analyses and regression models were complemented using OriginPro 2023 (OriginLab Corp., Northampton, MA, USA) for graphical fitting and validation of kinetic relationships.

## 3. Results

### 3.1. Physicochemical Evolution During Accelerated Storage

**Figure 1:**
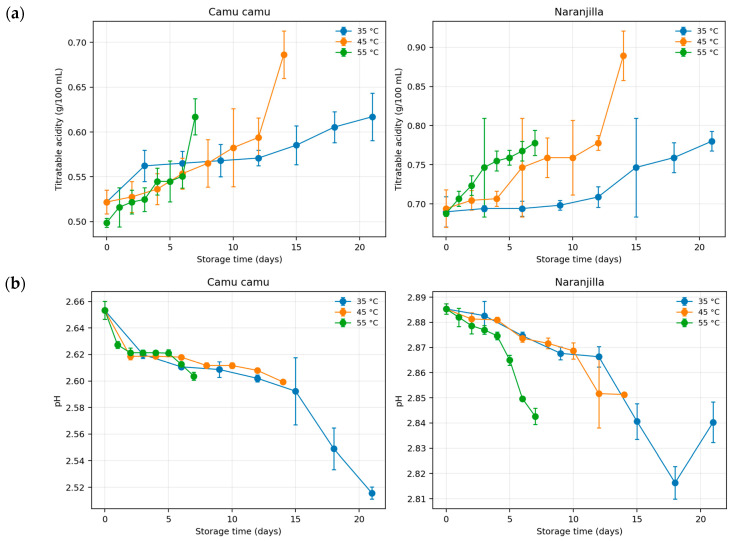

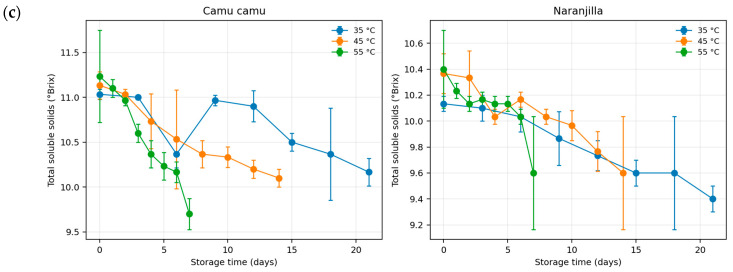
Changes in titratable acidity (**a**), pH level (**b**), and total soluble solids (°Brix) (**c**) during accelerated storage of tropical juices. Values are mean ± SD (n = 3).

Camu camu juice exhibited the strongest physicochemical response to storage, with titratable acidity increasing from 0.522 to 0.617 g/100 mL at 35 °C and from 0.499 to 0.617 g/100 mL at 55 °C, while pH decreased from 2.653 to 2.516 and 2.604, respectively (Figure 1). The corresponding °Brix values generally decreased with storage, suggesting soluble solids consumption and/or thermal breakdown of sugars. Naranjilla juice showed a more buffered behavior, with acidity increasing from approximately 0.69 to 0.78 g/100 mL and pH remaining in the 2.84–2.89 range, although the decline in °Brix was still evident under severe storage conditions. These results indicate that temperature accelerates matrix changes parallel with vitamin C degradation, particularly in the more reactive camu camu formulation.

Temperature and storage time exerted significant combined effects on the physicochemical variables (Table 1). In camu camu juice, the most pronounced statistical responses were observed for °Brix reduction and vitamin C degradation, whereas pH variation was primarily explained by storage time rather than temperature. In addition, the regression model describing vitamin C degradation explained approximately 70% of the observed variance (R^2^ = 0.70), indicating moderate predictive performance. This behavior is expected in complex fruit matrices with very high initial vitamin C concentrations, where degradation kinetics are influenced not only by temperature and storage time but also by oxygen availability, phenolic interactions, and matrix buffering effects. Therefore, the factorial regression captures the main storage trends, while the nonlinear Weibull model provided a more accurate description of degradation behavior. In contrast, naranjilla juice showed stronger temperature-driven effects on titratable acidity and vitamin C retention, while pH remained comparatively stable throughout storage. These results confirm that thermal exposure accelerates compositional shifts in both matrices, although the magnitude and sensitivity of the responses depend on fruit-specific buffering capacity and initial organic acid composition.

### 3.2. HPLC Quantification and Vitamin C Degradation

#### 3.2.1. Vitamin C Calibration Curve

**Figure 2:**
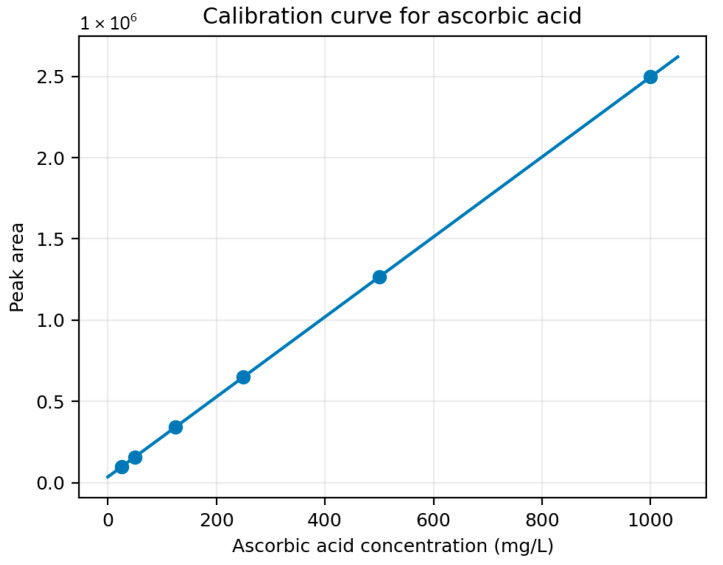
Calibration curve for ascorbic acid based on the reported external-standard equation.

Figure 2 shows the vitamin C calibration curve, with the regression model established as y = 2460.9x + 35708 and R^2^ of 0.9967470.

The calibration curve showed a high coefficient of determination (R^2^ = 0.9967), confirming excellent linearity within the studied concentration range (25–1000 mg L^−1^). The positive intercept observed in the regression equation is attributed to baseline absorbance contributions from the mobile phase and detector response at 245 nm, which are commonly reported in diode-array detection systems. Since quantification was performed within the validated calibration interval and sample concentrations were well above the lower calibration limit, the regression model was considered appropriate for accurate determination of vitamin C.

#### 3.2.2. Experimental Evolution of Vitamin C Concentration During Storage

**Figure 3:**
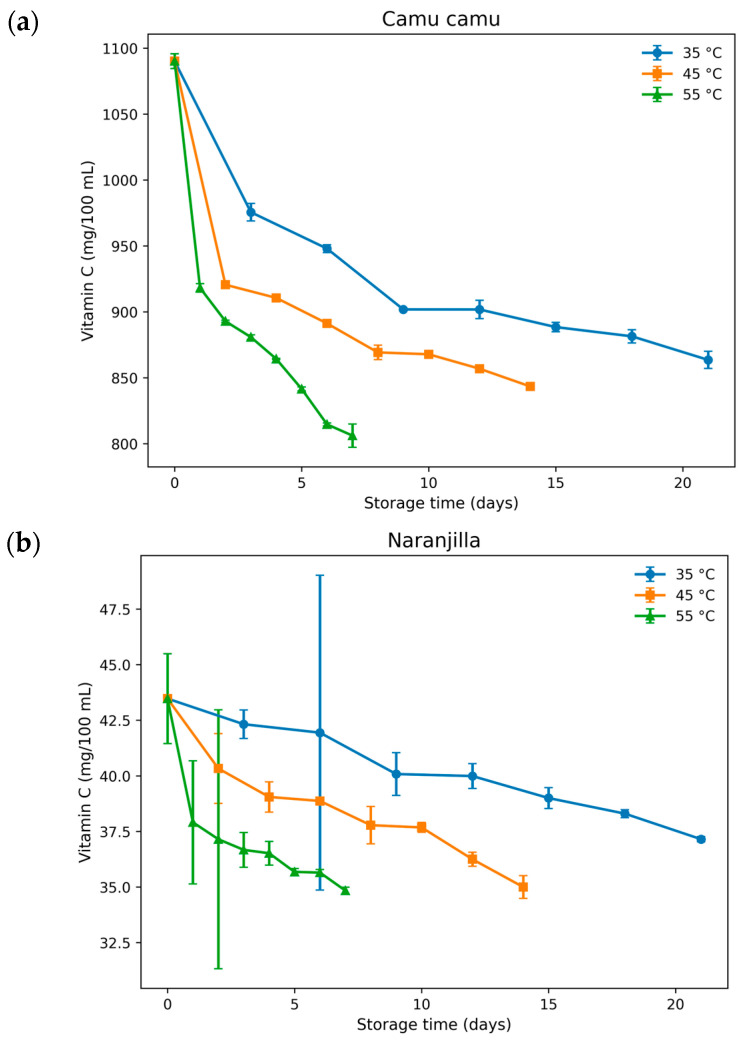
Degradation kinetics of vitamin C in camu camu (**a**) and naranjilla (**b**) juices during accelerated storage at 35, 45, and 55 °C (mean ± SD).

Vitamin C degradation during accelerated storage showed a clear temperature-dependent behavior in both juice matrices (Figure 3). Camu camu juice exhibited an initially high vitamin C concentration (1090.17 ± 5.63 mg/100 mL), which decreased progressively to 863.54 ± 6.51 mg/100 mL after 21 days at 35 °C (Figure 3a), whereas more pronounced reductions were observed at 45 °C and 55 °C, reaching final values of 843.40 ± 2.02 and 806.13 ± 8.79 mg/100 mL, respectively.

Similarly, naranjilla juice showed a gradual decline from 43.47 ± 2.02 to 37.15 ± 0.12 mg/100 mL at 35 °C, while storage at 45 °C and 55 °C accelerated the degradation process (Figure 3b), reducing the concentration to 35.00 ± 0.51 and 34.84 ± 0.15 mg/100 mL, respectively. The relatively small standard deviations observed throughout storage confirm the reproducibility of the analytical measurements and the stability of the experimental system. Statistical analysis indicated significant differences among storage temperatures (*p* < 0.05), confirming that thermal exposure strongly influenced vitamin C retention in both tropical juices. Overall, camu camu exhibited substantially higher absolute vitamin C levels than naranjilla, although both matrices followed comparable degradation trends characterized by monotonic decreases with increasing storage temperature. The figures in the Supplementary Materials, File S2, show representative chromatograms of the vitamin C standard, the camu camu and naranjilla juice samples.

### 3.3. Relationship Between Physicochemical Change and Vitamin C Retention

**Figure 4:**
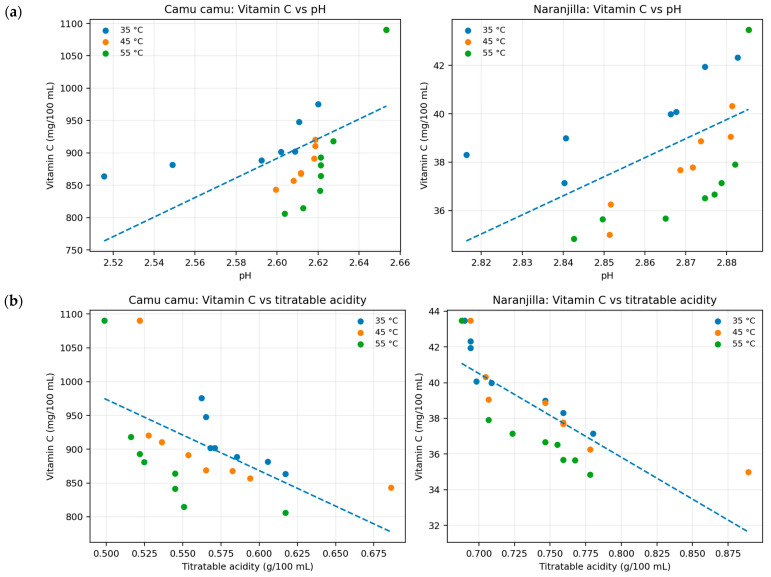
Relationship between pH and vitamin C content (**a**), and titratable acidity and vitamin C (**b**) during accelerated storage. Dashed lines represent global linear regression trends fitted using all experimental data points for each juice matrix.

Pearson analysis (Figure 4 and Table 2) showed that vitamin C was negatively associated with acidity and positively associated with pH in both juices. In camu camu, the strongest practical relationship was observed between vitamin C and °Brix (r = 0.770), while in naranjilla the inverse association with acidity was more pronounced (r = −0.788). These patterns support the interpretation that matrix acidification accompanied vitamin C degradation during storage.

### 3.4. Kinetic and Shelf-Life Interpretation

#### 3.4.1. Selection of the Kinetic Model for Vitamin C Degradation

Table 3 summarizes the regression equations and coefficients of determination (R^2^) obtained for zero-order, first-order, and Weibull models describing vitamin C degradation in camu camu and naranjilla juices under accelerated storage conditions. In both matrices, the Weibull model consistently provided the best fit to the experimental data across all temperatures, with R^2^ values above 0.98, indicating superior flexibility in describing the nonlinear degradation behavior of vitamin C compared with conventional kinetic approaches. These results confirm that vitamin C loss in the tropical juices studied follows a Weibull-type degradation pattern rather than a simple zero- or first-order reaction mechanism.

#### 3.4.2. Weibull Model Fitting and Log-Logistic Parameterization

##### Camu Camu Juice

**Figure 5:**
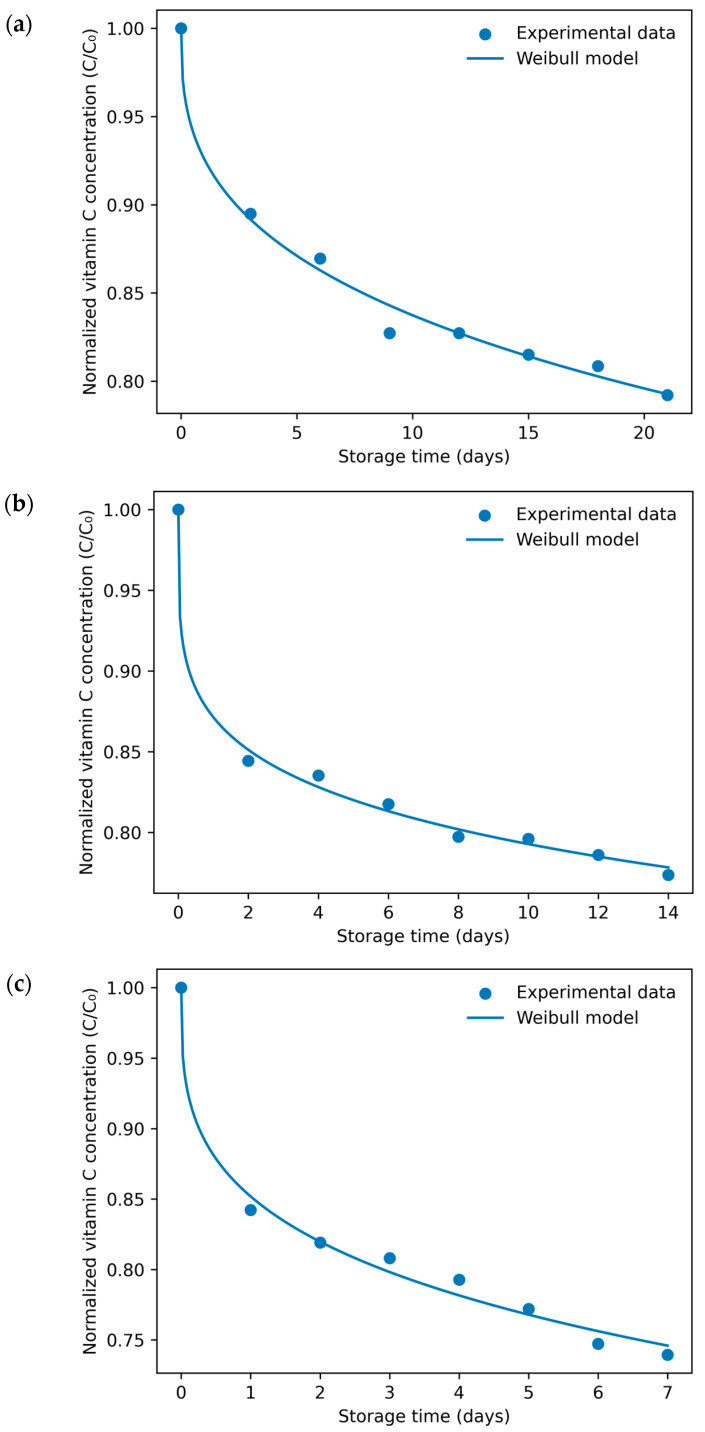
Weibull model fitting of vitamin C degradation kinetics in camu camu juice during accelerated storage at 35 °C (**a**), 45 °C (**b**), and 55 °C (**c**). Symbols represent experimental data, and solid lines correspond to model predictions.

The degradation kinetics of vitamin C in camu camu juice during accelerated storage at 35, 45, and 55 °C were accurately described using the Weibull model proposed by Corradini and Peleg [45] (Figure 5). Excellent goodness-of-fit was obtained under all storage conditions, with coefficients of determination (R^2^) higher than 0.98 and very low MSE and RMSE values (Table 4), confirming the suitability of the Weibull model to represent nonlinear degradation behavior in highly acidic tropical fruit matrices.

The Weibull scale parameter (b) increased progressively with storage temperature, rising from 0.07695 at 35 °C to 0.16037 at 55 °C (Table 4), indicating a marked acceleration of vitamin C degradation under thermal stress. This behavior reflects the thermolabile nature of vitamin C and agrees with previous observations reported for citrus-, acerola-, and mango-based beverages subjected to accelerated storage conditions. Temperature-driven increases in b are commonly associated with enhanced oxidative pathways and increased molecular mobility within liquid matrices.

The shape parameter (n) remained consistently below unity (0.22–0.36), revealing deviation from exponential decay and indicating a degradation profile characterized by an initially rapid loss followed by progressive stabilization. This tailing behavior has been attributed to reduced oxygen availability during storage, possible protection associated with naturally occurring phenolic compounds reported in these matrices, and diffusion-limited reaction mechanisms occurring in complex fruit systems. Such nonlinear survival patterns are typical of nutrient degradation processes occurring in multi-component matrices with strong buffering capacity. Although the Weibull shape parameter (n) did not follow a strictly monotonic trend with increasing temperature (0.363 at 35 °C, 0.227 at 45 °C, and 0.310 at 55 °C), all values remained below unity, confirming a consistent non-exponential degradation pattern characterized by an initial rapid loss followed by stabilization. From a kinetic perspective, n < 1 indicates that the apparent degradation rate decreases with storage time, suggesting that vitamin C loss is faster at early stages and progressively slows as the system evolves, which is consistent with heterogeneous reaction environments typically observed in complex liquid food matrices. Such variability in n with temperature has been reported in complex fruit matrices and may be associated with combined effects of oxygen availability, antioxidant interactions, and diffusion-limited reaction mechanisms rather than a single temperature-driven kinetic pathway.

**Figure 6:**
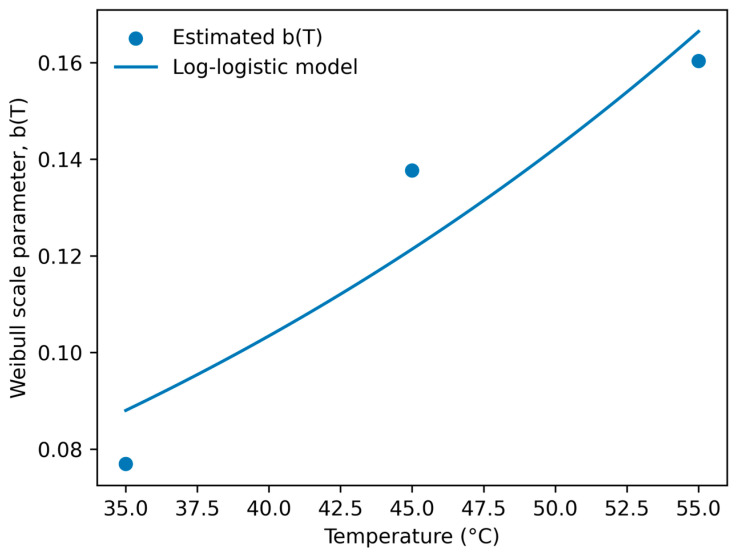
Log-logistic temperature dependence of Weibull parameter b for camu camu juice.

The temperature dependence of parameter b was further described using the log-logistic model (Figure 6), yielding values of *k* = 0.03386 and *T_c_* = 105.46 °C (R^2^ = 0.8858). Because the model was fitted using only three temperature levels, the resulting regression should be interpreted as a descriptive representation of the temperature-response trend rather than a statistically robust predictive relationship. Nevertheless, the use of three temperature conditions is consistent with common accelerated shelf-life testing (ASLT) designs applied in Arrhenius-type kinetic modeling, and the estimated critical temperature suggests a gradual thermal activation pattern within the evaluated range.

##### Naranjilla Juice

**Figure 7:**
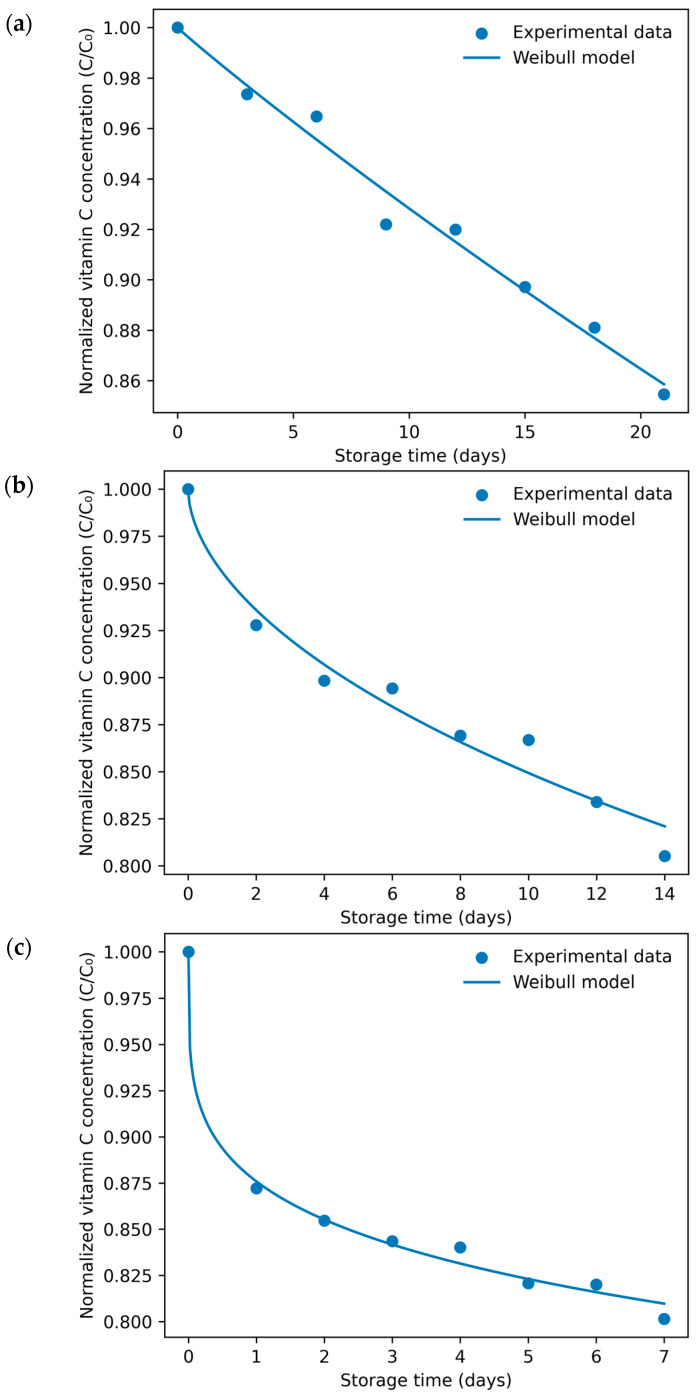
Weibull model fitting of vitamin C degradation kinetics in naranjilla juice during accelerated storage at 35 °C (**a**), 45 °C (**b**), and 55 °C (**c**). Symbols represent experimental data, and solid lines correspond to model predictions.

Vitamin C degradation in naranjilla juice was also successfully described by the Weibull model across all storage temperatures, with coefficients of determination exceeding 0.96 (Figure 7; Table 5). As observed in camu camu juice, the scale parameter (b) increased markedly with temperature, confirming the dominant role of thermal exposure in controlling degradation rates in this matrix.

In contrast to camu camu juice, the evolution of the shape parameter (n) revealed a clear transition in kinetic behavior. At 35 °C (n ≈ 0.97), degradation followed a near-exponential pattern consistent with pseudo-first-order kinetics, whereas at higher storage temperatures (45–55 °C) n decreased substantially (0.56–0.24), indicating progressively stronger deviations from linear degradation behavior. This transition suggests that elevated temperatures promote diffusion-controlled oxidation mechanisms and matrix-dependent protective effects that alter reaction-rate dynamics over time.

**Figure 8:**
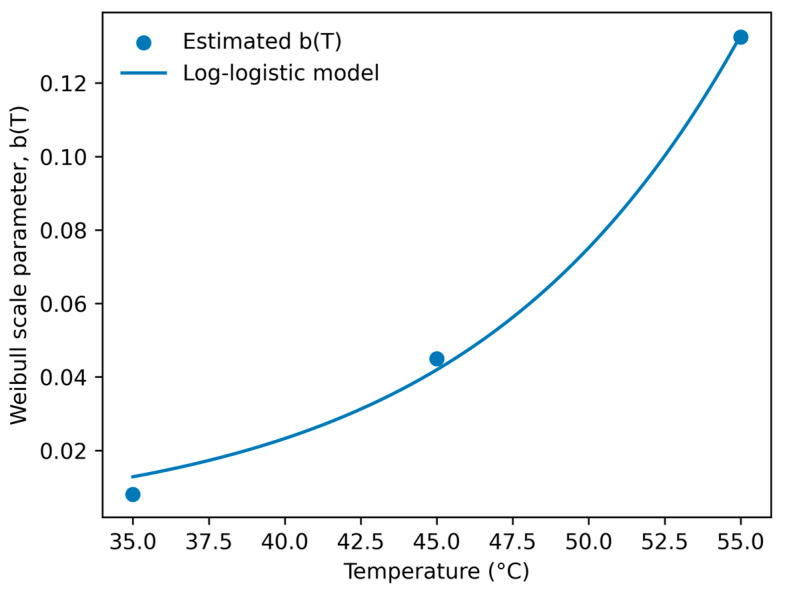
Log-logistic temperature dependence of Weibull parameter b for naranjilla juice.

The thermal dependence of the Weibull rate parameter *b* was accurately described using the log-logistic relationship (Figure 8), producing parameter estimates of *k* = 0.11997 and *T_c_* = 71.26 °C with excellent predictive performance (R^2^ = 0.9961). Compared with camu camu juice, the lower critical temperature indicates a greater sensitivity of the naranjilla matrix to thermal activation of degradation pathways, likely associated with its lower antioxidant buffering capacity and reduced organic acid stabilization.

##### Comparative Kinetic Behavior Between Tropical Juice Matrices

A comparative evaluation of Weibull parameters revealed matrix-dependent differences in degradation dynamics between camu camu and naranjilla juices. Although both systems exhibited temperature-dependent increases in the scale parameter (b), indicating accelerated degradation rates under thermal stress, camu camu juice showed consistently lower curvature variability and higher kinetic stability across storage conditions. This behavior is consistent with its elevated initial vitamin C concentration and the presence of naturally occurring antioxidant compounds capable of delaying oxidative reactions. In contrast, the stronger temperature sensitivity observed in naranjilla juice, reflected by lower T_c_ values and pronounced reductions in the shape parameter (n), suggests that degradation mechanisms become increasingly diffusion-limited and matrix-controlled at higher temperatures. These differences highlight the importance of compositional factors such as the organic acid profile, phenolic content, and buffering capacity in determining vitamin C stability during storage.

Overall, the Weibull–log-logistic modeling framework provided a robust description of vitamin C degradation kinetics in both tropical juice matrices and allowed identification of matrix-specific thermal sensitivities relevant for predictive shelf-life estimation under accelerated storage conditions.

#### 3.4.3. Arrhenius Relationship and Activation Energy

The temperature dependence of the Weibull scale parameter (*b*) was evaluated using the secondary Arrhenius model, allowing the estimation of the activation energy (*E_a_*) and pre-exponential factor (*b*_0_) for vitamin *C* degradation in both tropical juice matrices. The Arrhenius parameters obtained from linear regression of ln *b*(*T*) versus reciprocal absolute temperature (1/T) are presented in Table 6.

Comparison between matrices showed that the estimated activation energy for naranjilla juice was substantially higher than that obtained for camu camu juice, indicating a stronger temperature dependence of the Weibull rate parameter in this system. The higher Arrhenius coefficient of determination observed for naranjilla juice (R^2^ = 0.995) further supports a predominantly temperature-driven degradation mechanism in this system. Conversely, the lower, but still acceptable, fit obtained for camu camu juice (R^2^ = 0.908) suggests that vitamin C degradation in this matrix is influenced by multiple simultaneous mechanisms beyond purely thermal activation. Overall, the Arrhenius analysis confirmed matrix-dependent differences in thermal sensitivity of vitamin C degradation, with camu camu juice showing greater resistance to temperature-induced deterioration and naranjilla juice exhibiting a markedly stronger kinetic response to thermal stress. These findings reinforce the relevance of combining Weibull primary modeling with Arrhenius secondary relationships for reliable prediction of nutrient stability in complex tropical fruit beverages under accelerated storage conditions.

#### 3.4.4. Shelf-Life Prediction (*t*_80_)

##### Shelf-Life Estimation Based on Vitamin C Retention

The shelf life of camu camu and naranjilla juices was estimated using the Weibull kinetic model considering 80% retention of the initial vitamin C concentration (*t*_80_). The calculated Weibull parameters and corresponding shelf-life values at each storage temperature are presented in Table 7.

In the case of camu camu juice, the estimated *t*_80_ values showed a progressive decrease in the retention time of vitamin C as storage temperature increased. Similarly, naranjilla juice exhibited *t*_80_ values that confirmed the strong influence of temperature on the degradation kinetics in both matrices. Shelf-life extrapolation to lower storage temperatures was performed using the Arrhenius relationship derived from the Weibull rate parameter. For camu camu juice, predicted *t*_80_ values ranged from 524 days at 5 °C to 32 days at 30 °C, indicating a substantial increase in vitamin C retention at reduced temperatures. In contrast, the predicted shelf life of naranjilla juice showed a similar temperature-dependent trend but with shorter retention times under equivalent storage conditions. Comparison between matrices revealed longer *t*_80_ values for naranjilla juice than for camu camu juice at the evaluated accelerated storage temperatures, particularly at 35 °C and 45 °C, reflecting differences in the temperature dependence of the Weibull degradation parameters.

##### Prediction of Shelf Life Based on Vitamin C Retention

Shelf-life prediction for camu camu and naranjilla juices at refrigeration and ambient storage temperatures (5–30 °C) was performed using an Arrhenius-type secondary model based on the *t*_80_ values previously estimated from the Weibull kinetic parameters at accelerated storage conditions (35, 45, and 55 °C). The regression equations obtained from the linearized Arrhenius relationship (Equation (11)) and the corresponding activation energy values are presented in Table 8.

(11) $$ln\left[t_{80\;}\right]=\ln\left[A\right]-\left[\left(\frac{E_{a}}{R}\right)\frac{1}{\left(T+273.15\right)}\right]$$

The Arrhenius-type regression showed excellent goodness-of-fit for both juice matrices, with coefficients of determination of 0.9907 for camu camu and 0.9959 for naranjilla juice. The activation energy estimated from the temperature dependence of *t*_80_ was 78.3 kJ/mol for camu camu juice and 52.9 kJ/mol for naranjilla juice, confirming the strong influence of temperature on vitamin C retention time in both systems. Predicted shelf-life values expressed as *t*_80_ for storage temperatures between 5 and 30 °C are summarized in Table 9. For camu camu juice, the predicted retention time ranged from 524 days at 5 °C to 32 days at 30 °C, whereas for naranjilla juice the corresponding values ranged from 292 days at 5 °C to 44 days at 30 °C. In both matrices, *t*_80_ decreased progressively with increasing storage temperature, following the exponential temperature dependence described by the Arrhenius relationship.

It should be noted that the activation energy values obtained from the Arrhenius relationship applied to the Weibull rate parameter b(T) (Table 6) differ from those derived from the temperature dependence of shelf-life parameter *t*_80_ (Table 8) because each approach describes a different kinetic descriptor. While E_a_ estimated from b(T) reflects the temperature sensitivity of degradation rate, E_a_ derived from *t*_80_ represents the temperature dependence of nutrient retention time. Therefore, both analyses provide complementary rather than contradictory information about thermal effects on vitamin C stability.

At intermediate temperatures, predicted shelf-life values showed comparable retention times for both juices, particularly between 20 and 30 °C. Specifically, estimated *t*_80_ values at 20 °C were 93 days for camu camu and 91 days for naranjilla juice, while at 25 °C the predicted retention times were 54 days and 63 days, respectively. These results indicate that Arrhenius-based secondary modeling provides a useful framework for estimating vitamin C retention under non-accelerated storage conditions using Weibull-derived degradation parameters.

## 4. Discussion

The degradation kinetics of vitamin C observed in both camu camu and naranjilla juices showed clear deviations from classical zero and first order reaction models, supporting the suitability of the Weibull formulation for describing nutrient loss in complex fruit matrices. Similar findings have been reported in citrus and berry-based beverages, where oxygen availability, matrix heterogeneity, and antioxidant co-components produce non-exponential degradation patterns better captured by empirical survival-type models [45,46,53,54].

The evolution of physicochemical parameters during storage was consistent with the observed degradation trends. The increase in titratable acidity and reduction in pH detected during accelerated storage are commonly associated with vitamin C oxidation and the formation of low-molecular-weight organic acids derived from its degradation pathway [55]. Similarly, the moderate decline in soluble solids observed in both juices agrees with previous studies reporting temperature-dependent compositional shifts during storage of acidic fruit beverages [29,56].

The consistently high goodness-of-fit obtained for the Weibull model (R^2^ > 0.98) confirms its robustness for describing vitamin C degradation under accelerated storage conditions in tropical juices. In both matrices, the increase in the scale parameter b with temperature reflected the expected thermally activated nature of vitamin C oxidation, as previously reported for orange, mango, and acerola beverages subjected to similar storage conditions [33,57]. Moreover, the shape parameter values (n < 1) indicated non-exponential degradation behavior characterized by an initial rapid loss followed by progressive stabilization, a pattern frequently associated with oxygen depletion and antioxidant protection effects in liquid food systems [53].

This moderate value indicates that temperature exerts a measurable but not dominant control over degradation kinetics, suggesting that additional physicochemical factors, including matrix acidity and oxygen availability, as well as bioactive constituents reported in these fruit matrices, may also influence vitamin C stability during storage. The relatively high acidity of camu camu juice (pH ≈ 2.65) likely contributed to enhanced stabilization of vitamin C, since degradation reactions proceed more slowly under acidic conditions than near-neutral pH environments [58]. Furthermore, storage in hermetically sealed glass containers, characterized by extremely low oxygen permeability, may have reduced oxidative pathways responsible for vitamin C conversion into dehydroascorbic acid, thereby contributing to the lower thermal sensitivity observed in this matrix. Although phenolic compounds and antioxidant capacity were not directly quantified in the present study, previous reports describing the composition of camu camu and naranjilla juices suggest that these constituents may contribute to the observed stability patterns. Therefore, their role is discussed here as a plausible matrix-related factor rather than a directly measured variable. This consideration is particularly relevant when interpreting the matrix-dependent differences observed in the Arrhenius parameters between both juices.

Matrix-dependent differences between juices were evident in both Weibull and Arrhenius parameters. Camu camu juice showed lower activation energy values (31.05 kJ/mol), consistent with previously reported ranges for vitamin C degradation in acidic fruit systems (approximately 30–70 kJ/mol) [57]. The relatively moderate temperature sensitivity observed in this matrix may be associated with its extremely high initial vitamin C concentration and with phenolic compounds previously reported in camu camu, which have been suggested in the literature to contribute to delaying oxidative reactions through radical scavenging mechanisms [7,11]. In contrast, the higher activation energy estimated for naranjilla juice (117.8 kJ/mol) indicates a stronger dependence of degradation rate on temperature, suggesting reduced intrinsic protection against oxidative pathways compared with camu camu. Although this value exceeds the range commonly reported for simpler fruit systems, elevated apparent activation energies have been described in complex matrices where interactions among organic acids, phenolic compounds, and dissolved oxygen may influence the temperature dependence of degradation kinetics. In the present study, storage in hermetically sealed glass containers with limited headspace likely reduced external oxygen diffusion; however, dissolved oxygen levels were not directly quantified and therefore cannot be considered a determining factor in the observed kinetic differences between matrices.

Such elevated E_a_ values suggest that small increases in storage temperature produce disproportionately large increases in the rate of vitamin C loss. Compared with camu camu juice, this behavior reflects lower intrinsic matrix protection against oxidation, possibly associated with reduced buffering capacity and lower concentrations of naturally occurring antioxidant compounds. It should be noted, however, that apparent differences in thermal sensitivity depended on whether the Arrhenius relationship was applied to the Weibull rate parameter b(T) or to the predicted shelf-life parameter *t*_80_, highlighting the influence of the selected kinetic descriptor on the interpretation of temperature effects. Considering that the Arrhenius relationship was derived from only three temperature levels and applied to the Weibull rate parameter b(T), the estimated E_a_ should be interpreted as an indicator of relative thermal sensitivity rather than an absolute mechanistic constant.

Shelf-life estimation based on 80% vitamin C retention (*t*_80_) further confirmed the strong temperature dependence of degradation kinetics. The progressive reduction in *t*_80_ with increasing storage temperature observed in both matrices follows the classical Arrhenius behavior reported for citrus and tropical juices, where vitamin C degradation rates may increase severalfold for each 10 °C rise in temperature [57,59]. Predicted retention times exceeding 500 days at refrigeration temperature for camu camu juice suggest a strong potential benefit of low-temperature storage for preserving functional quality in vitamin-C-rich beverages. However, because these values were obtained by Arrhenius extrapolation from accelerated storage conditions, they should be interpreted as indicative estimates rather than exact shelf-life predictions.

Although accelerated storage experiments provide an efficient framework for estimating degradation kinetics within a practical experimental timeframe, extrapolation of Arrhenius-based predictions to real storage conditions may involve additional sources of uncertainty. In particular, factors such as light exposure, packaging conditions, and matrix-specific interactions during commercial distribution were not explicitly evaluated in the present study and may influence vitamin C stability. Therefore, the proposed modeling framework should be interpreted primarily as a predictive comparative tool rather than an absolute descriptor of stability under all storage scenarios. The combined application of Weibull primary modeling and Arrhenius-type secondary relationships therefore provides a reliable framework for predicting vitamin C stability in tropical juice systems. These results confirm that nonlinear kinetic approaches offer improved flexibility compared with classical reaction-order models and represent a suitable strategy for shelf-life prediction in complex matrices naturally rich in bioactive compounds such as camu camu and naranjilla juices.

The results obtained in this study support the initial hypothesis that vitamin C degradation in tropical juices such as camu camu and naranjilla cannot be adequately described using conventional zero- or first-order kinetic models alone, and that the Weibull model provides a more accurate representation of degradation behavior under accelerated storage conditions. The consistently higher goodness-of-fit obtained for the Weibull model compared with classical kinetic approaches (R^2^ > 0.98) confirms its suitability for capturing nonlinear degradation patterns associated with matrix composition and temperature-dependent effects. Furthermore, the successful integration of Weibull primary modeling with Arrhenius-type secondary relationships allowed reliable prediction of vitamin C retention times across a wide temperature range, demonstrating the applicability of this combined modeling framework for shelf-life estimation in complex tropical juice systems.

## 5. Conclusions

The degradation kinetics of vitamin C in camu camu (*M. dubia*) and naranjilla (*S. quitoense* Lam.) juices during accelerated storage were successfully described using a Weibull modeling framework combined with Arrhenius-type temperature dependence. Among the evaluated kinetic approaches, the Weibull model consistently provided the best statistical fit (R^2^ > 0.98), confirming its suitability for representing nonlinear degradation behavior in complex tropical juice matrices. Temperature significantly affected vitamin C stability in both juices, with the Weibull scale parameter increasing as storage temperature rose from 35 to 55 °C. Matrix-dependent differences in apparent thermal sensitivity were observed, depending on whether Arrhenius analysis was applied to the Weibull rate parameter b(T) or to the shelf-life descriptor *t*_80_. Shelf-life estimation based on 80% vitamin C retention showed a pronounced exponential dependence on storage temperature, with predicted retention times ranging from 19 to 3 days for camu camu juice and from 31 to 9 days for naranjilla juice under accelerated conditions, and extrapolated values at refrigeration temperatures reaching up to 524 and 292 days, respectively.

These findings highlight the importance of temperature control for preserving the nutritional quality of tropical fruit beverages during storage. The integration of Weibull primary modeling with Arrhenius-type secondary relationships provides a useful predictive tool for estimating vitamin C retention under different storage scenarios, supporting shelf-life labeling, optimization of distribution conditions, and selection of storage temperatures for functional juice products.

## Figures and Tables

**Table 1:** Regression-based significance analysis for the physicochemical dataset.

Fruit	Variable	*p* (Temp)	*p* (Time)	*p* (Temp × Time)	η^2^ Temp	η^2^ Time	R^2^
Camu camu	Acidity	4.440 × 10^−1^	8.755 × 10^−8^	1.694 × 10^−4^	0.01	0.61	0.84
	pH	5.978 × 10^−1^	1.421 × 10^−8^	4.103 × 10^−2^	0.00	0.77	0.85
	Brix	1.706 × 10^−4^	5.707 × 10^−7^	1.074 × 10^−4^	0.18	0.45	0.79
	Vitamin C	1.743 × 10^−4^	6.585 × 10^−6^	2.251 × 10^−2^	0.25	0.44	0.70
Naranjilla	Acidity	8.732 × 10^−6^	4.461 × 10^−8^	1.142 × 10^−4^	0.23	0.48	0.83
	pH	4.622 × 10^−2^	1.060 × 10^−7^	8.049 × 10^−1^	0.05	0.72	0.78
	Brix	9.145 × 10^−1^	1.211 × 10^−8^	1.633 × 10^−1^	0.00	0.79	0.85
	Vitamin C	1.211 × 10^−8^	2.635 × 10^−8^	2.346 × 10^−3^	0.44	0.40	0.86

Note. Regression-based factorial analysis evaluated the influence of storage temperature (Temp), storage time (Time), and their interaction (Temp × Time) on physicochemical properties and vitamin C degradation in camu camu and naranjilla juices during accelerated storage. Reported values correspond to *p*-values from the regression models, partial eta-squared (η^2^) effect sizes, and coefficients of determination (R^2^). Effect sizes were interpreted as small (η^2^ ≈ 0.01), moderate (η^2^ ≈ 0.06), and large (η^2^ ≥ 0.14). Statistical significance was established at *p* < 0.05. Analyses were performed using averaged triplicate measurements for each sampling condition.

**Table 2:** Pearson correlations between vitamin C and explanatory variables.

Fruit	Vitamin C vs. Acidity	Vitamin C vs. pH	Vitamin C vs. °Brix	Vitamin C vs. Temperature	Vitamin C vs. Time
Camu camu	−0.555	0.559	0.770	−0.224	−0.532
Naranjilla	−0.788	0.532	0.460	−0.470	−0.407

Note. Pearson correlation coefficients (r) describe the association between vitamin C and physicochemical variables during storage (n = number of sampling points per temperature condition). Positive values indicate direct relationships and negative values indicate inverse relationships. Correlations were interpreted as strong (|r| ≥ 0.70), moderate (0.40–0.69), or weak (<0.40). Statistical significance was established at *p* < 0.05. Correlation analyses were performed using averaged triplicate measurements.

**Table 3:** Regression equations and coefficients of determination (R^2^) for zero-order, first-order, and Weibull models describing vitamin C degradation kinetics.

Tropical Juice	Model	Temperature (°C)			
		35	45	55	Selection
		Function (R^2^)	Function (R^2^)	Function (R^2^)	
Camu camu	Zero-order	y = −8.8722x + 1024.5 (0.7803)	y = −13.072x + 997.74 (0.6597)	y = −31.866x + 1000.1 (0.7526)	Weibull model
	First-order	y = −0.0093x + 6.9313 (0.8051)	y = −0.0138x + 6.9031 (0.691)	y = −0.0346x + 6.9067 (0.7914	
	Weibull model	y = $exp(-0.07695t^{0.36305})$ (0.9895)	y = $exp(-0.1377t^{0.22699})$ (0.9954)	y = $exp(-0.16037t^{0.309870})$ (0.9905)	
Naranjilla	Zero-order	y = −0.2908x + 43.334 (0.9807)	y = −0.5053x + 42.091 (0.9133)	y = −0.9083x + 40.416 (0.6816)	Weibull model
	First-order	y = −0.0072x + 3.7704 (0.9815)	y = −0.013x + 3.7412 (0.926)	y = −0.0236x + 3.6978 (0.7087)	
	Weibull model	y = $exp(-0.00804t^{0.96644})$ (0.9812)	y = $exp(-0.04493t^{0.56064})$ (0.9678)	y = $exp(-0.1325t^{0.23923})$ (0.9931)	

Note. The model with the highest coefficient of determination (R^2^) at each storage temperature was selected as the best descriptor of vitamin C degradation kinetics among zero-order, first-order, and Weibull models.

**Table 4:** Weibull and log–logistic model parameters for camu camu juice.

**Weibull model (Equation (6))**					
**Temperature (°C)**	**b(T)**	**n(T)**	**R^2^**	**MSE**	**RMSE**
35	0.07695	0.36305	0.9895	0.000042	0.00648
45	0.1377	0.22699	0.9954	0.000021	0.00461
55	0.16037	0.30987	0.9905	0.000057	0.00752
**Log-logistic model (Equation (7))**					
b(T) vs. T	**k_3_**	**T_c_**	**R^2^**	**MSE**	**RMSE**
Weibull	0.03386	105.46 °C	0.8858	0.000142	0.01190

**Table 5:** Weibull and log-logistic model parameters for naranjilla juice.

**Weibull model (Equation (6))**					
**Temperature (°C)**	**b(T)**	**n(T)**	**R^2^**	**MSE**	**RMSE**
35	0.00804	0.96644	0.9812	4.1 × 10^−5^	0.00637
45	0.04493	0.56064	0.9678	1.0 × 10^−4^	0.01000
55	0.13250	0.23923	0.9931	2.3 × 10^−5^	0.00480
**Log-logistic model (Equation (7))**					
b(T) vs. T	**k_3_**	**T_c_**	**R^2^**	**MSE**	**RMSE**
Weibull	0.11997	71.26	0.9961	1.07 × 10^−5^	0.00327

**Table 6:** Arrhenius parameters describing the temperature dependence of the Weibull rate parameter b(T) for vitamin C degradation in tropical juices.

Tropical Juice	*E_a_* (kJ/mol)	SE (kJ/mol)	*b*_0_	Arrhenius Equation Calculated	R^2^
Camu camu	31.05	±4.82	1.505 × 10^4^	$b(T)=1.505\times 10^{4}e^{\left[-\left(\frac{31.05}{RT}\right)\right]}$	0.908
Naranjilla	117.8	±18.64	1.3 × 10^17^	$b(T)=1.3\times 10^{17}e^{\left[-\left(\frac{117.8}{RT}\right)\right]}$	0.995

Note. Standard errors (SE) of activation energy (*E_a_*) were obtained from linear regression of Arrhenius plots (ln b versus 1/T). Because the model was fitted using three temperature levels, the reported SE values reflect the uncertainty associated with limited degrees of freedom.

**Table 7:** Estimated shelf life (*t*_80_) for the degradation of vitamin C in tropical juices.

Tropical Juice	Temperature (°C)	*b*	*n*	*t*_80_ Weibull (Days)
Camu camu	35	0.07695	0.36305	19
	45	0.1377	0.22699	8
	55	0.16037	0.30987	3
Naranjilla	35	0.00804	0.96644	31
	45	0.04493	0.56064	17
	55	0.13250	0.23923	9

**Table 8:** Parameters of the Arrhenius-type fit vs. estimated *t*_80_.

Tropical Juice	Regression	$E_{a}$ (kJ/mol)	*A*	R^2^
Camu camu	y = 9420.4x − 27.588	78.3	1.04422 × 10^−12^	0.9907
Naranjilla	y = 6363.1x − 17.188	52.9	3.42981 × 10^−8^	0.9959

**Table 9:** Predicted shelf life (*t*_80_) for the degradation of vitamin C at refrigeration and everyday storage temperatures.

Temperature (°C)	*t*_80_ (Days) Weibull	
	Camu Camu	Naranjilla
5	524	292
10	288	195
15	162	132
20	93	91
25	54	63
30	32	44

## Data Availability

The original contributions presented in the study are included in the article/Supplementary Materials; further inquiries can be directed to the corresponding author.

## Supplementary Materials

The following supporting information can be downloaded at: https://www.mdpi.com/article/10.3390/foods15101722/s1, Figure S1: Flow diagram of camu camu juice processing; Figure S2: Flow diagram of naranjilla juice processing; File S1: Report HPLC chromatogram of obtained from camu camu juice extract and vitamin C standard solution used for compound identification and retention time determination; File S2: Report HPLC chromatogram of obtained from naranjilla juice extract and vitamin C standard solution used for compound identification and retention time determination.
