# Supplementary material for: Kinetic Modeling of Vitamin C Degradation for Predicting Shelf Life in Tropical Juices Made from Camu Camu and Naranjilla Under Accelerated Storage Conditions
Source: Foods. 2026 May 14;15(10):1722. doi: 10.3390/foods15101722 (PMC13205238; doi:10.3390/foods15101722)
Supplement: Supplementary file 1 [file foods-15-01722-s001.zip › Figure S1. Flow diagram of camu-camu juice processing.pdf]

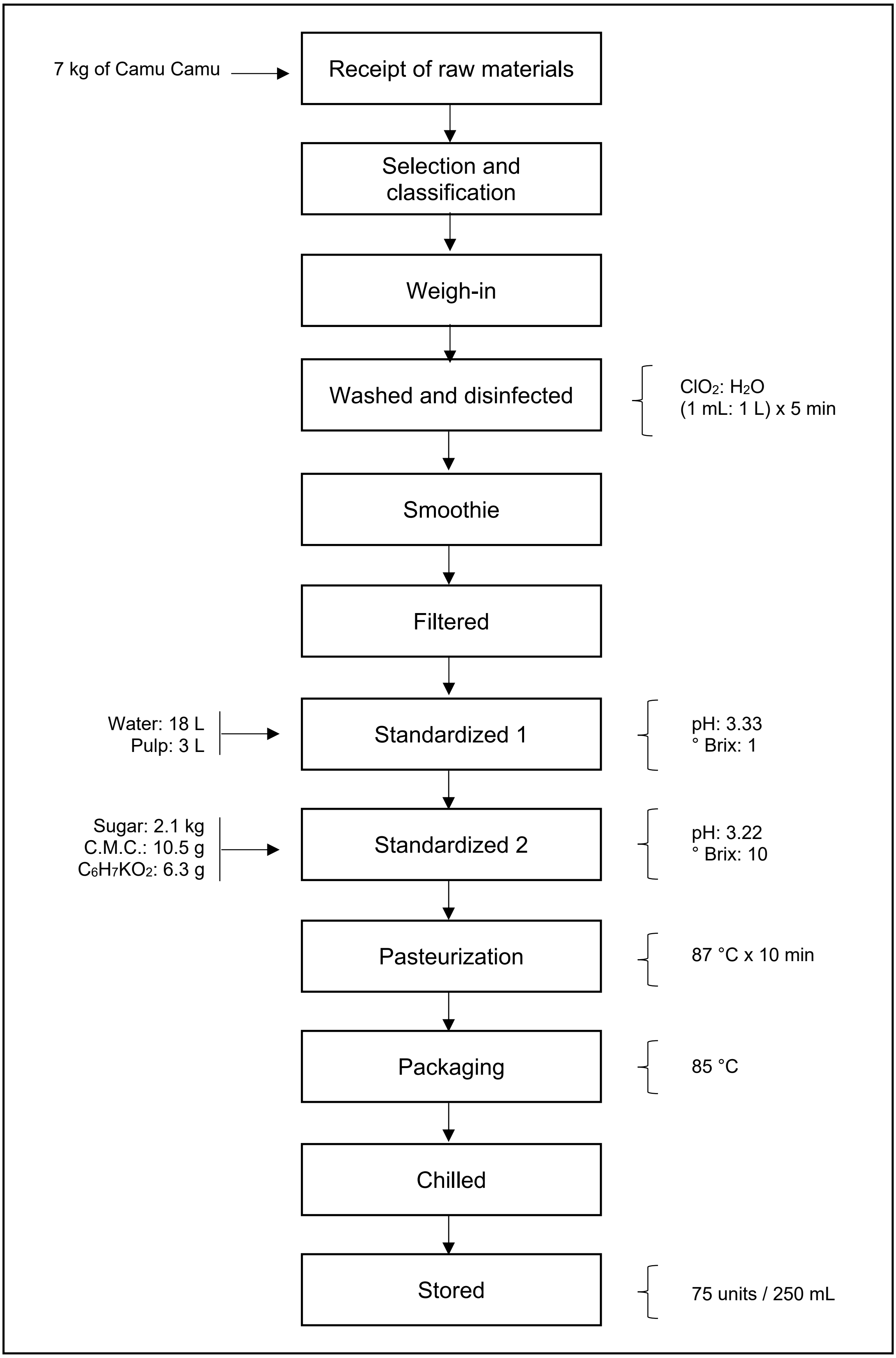

Figure S1. Flow diagram of camu camu juice processing including washing, chlorine dioxide disinfection (1 mL L^{-1}, 5 min), homogenization, filtration (1 mm mesh), physicochemical standardization (pH and °Brix adjustment), pasteurization (87 °C, 10 min), hot filling (~85 °C), rapid cooling, and accelerated storage at 35, 45, and 55 °C.
