# Supplementary material for: Kinetic Modeling of Vitamin C Degradation for Predicting Shelf Life in Tropical Juices Made from Camu Camu and Naranjilla Under Accelerated Storage Conditions
Source: Foods. 2026 May 14;15(10):1722. doi: 10.3390/foods15101722 (PMC13205238; doi:10.3390/foods15101722)
Supplement: Supplementary file 1 [file foods-15-01722-s001.zip › Figure S2. Flow diagram of naranjilla juice processing.pdf]

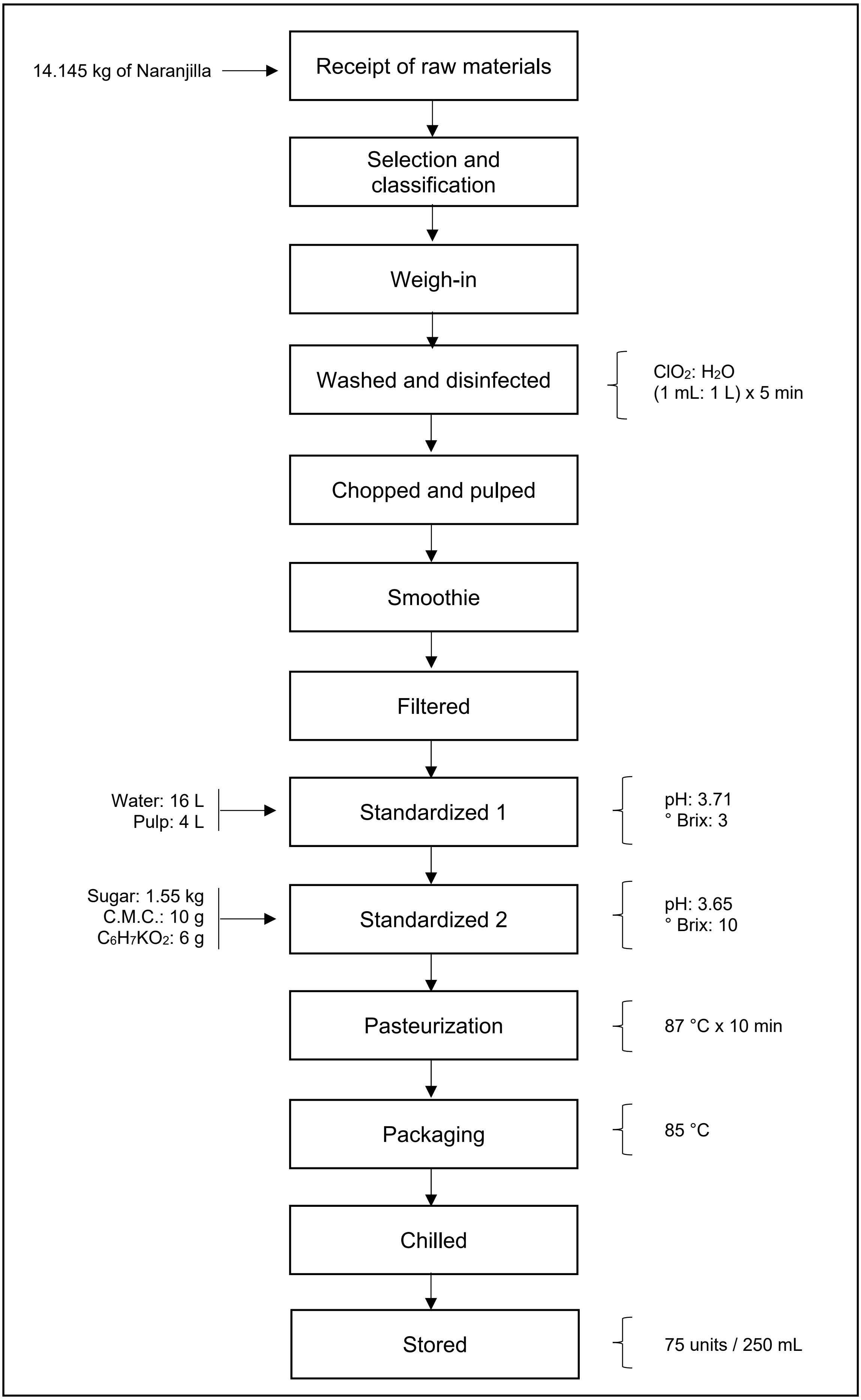

Figure S2. Flow diagram of naranjilla juice processing including washing, chlorine dioxide disinfection (1 mL L^{-1}, 5 min), pulping, homogenization, filtration (1 mm mesh), formulation adjustment (pH and °Brix), pasteurization (87 °C, 10 min), hot filling (~85 °C), rapid cooling, and accelerated storage under controlled temperature conditions
