# Supplementary material for: Kinetic Modeling of Vitamin C Degradation for Predicting Shelf Life in Tropical Juices Made from Camu Camu and Naranjilla Under Accelerated Storage Conditions
Source: Foods. 2026 May 14;15(10):1722. doi: 10.3390/foods15101722 (PMC13205238; doi:10.3390/foods15101722)
Supplement: Supplementary file 1 [file foods-15-01722-s001.zip › File S1. Report HPLC chromatogram of obtained from Camu-Camu juice extract and ascorbic acid standard solution used for compound identification and retention time determination.PDF]

Shimadzu LabSolutions Report
PEDRO RUIZ GALLO NATIONAL UNIVERSITY-FIQIA
VITAMIN C CHROMATOGRAM FOR CAMU JUICE CAMU

Summary(Compound)

CAMU55-D1-VITC-01.lcd

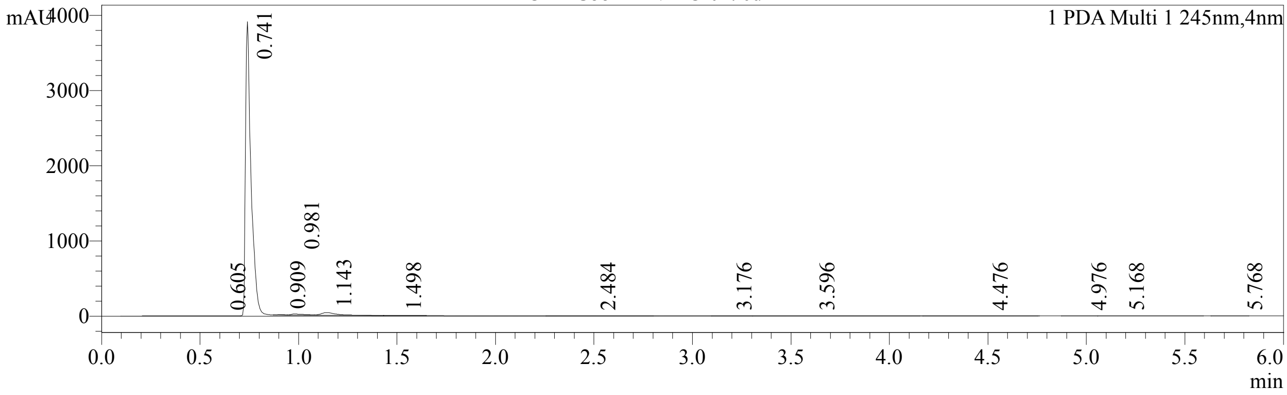

CAMU55-D1-VITC-02.lcd

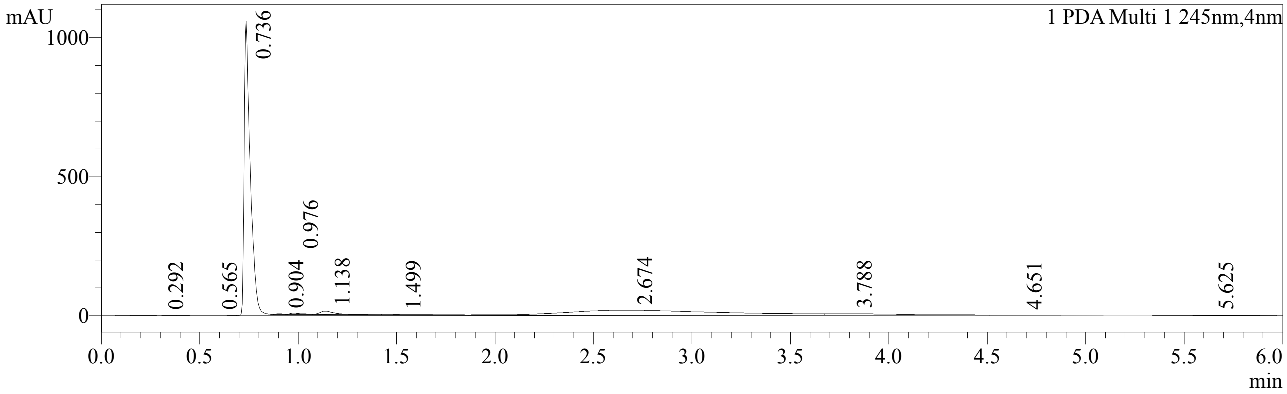

CAMU55-D1-VITC-03.lcd

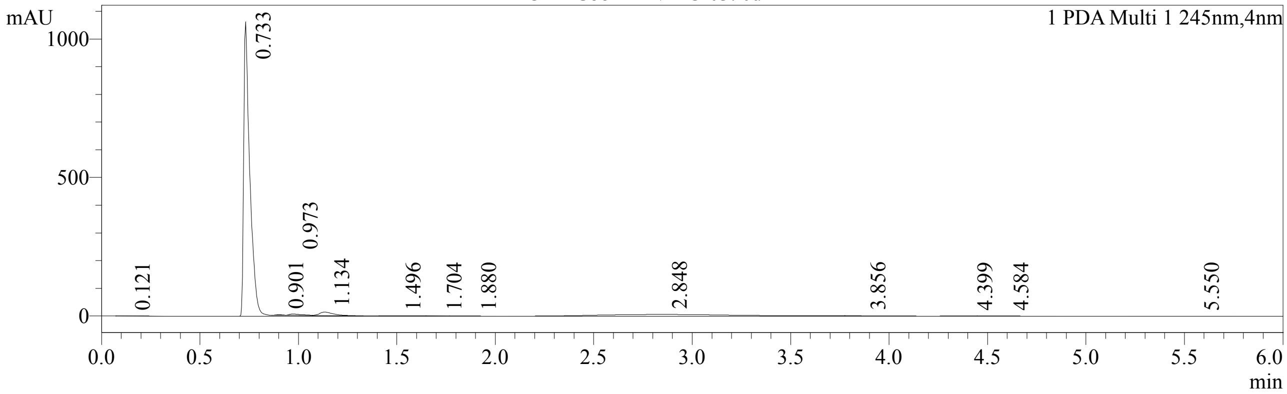

CAMU55-D1D-VITC-01.lcd

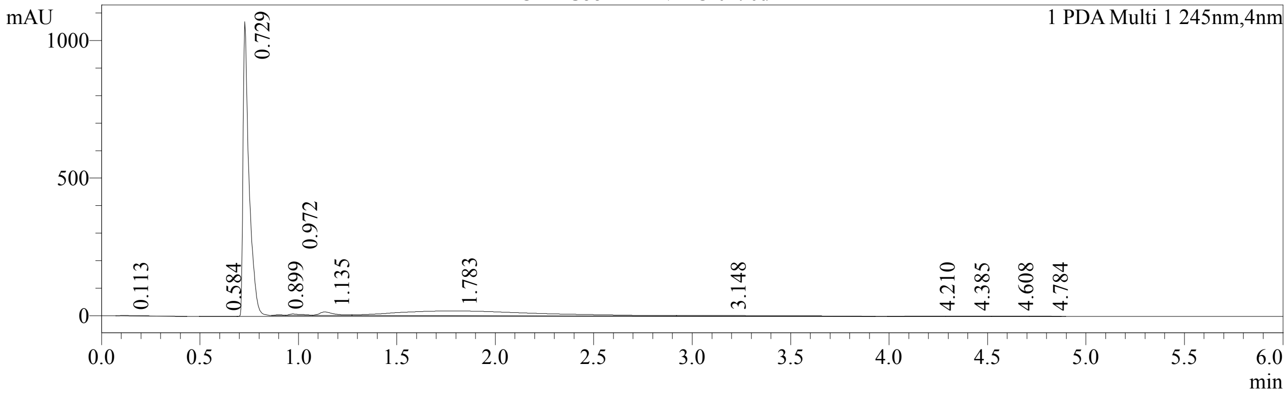

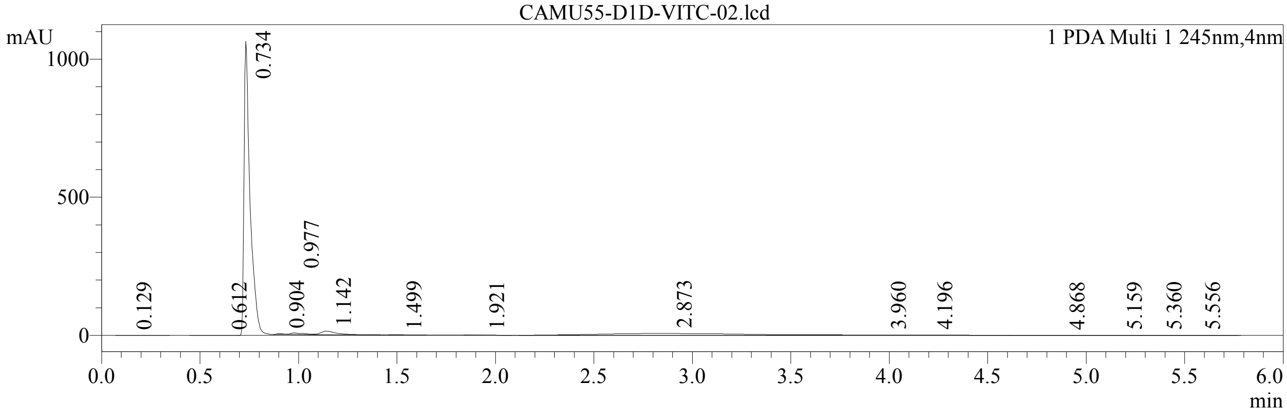
CAMU55-D1D-VITC-02.lcd
mAU
1 PDA Multi 1 245nm,4nm
0.734
0.977
0.129
0.612
0.904
1.142
1.499
1.921
2.873
3.960
4.196
4.868
5.159
5.360
5.556
min

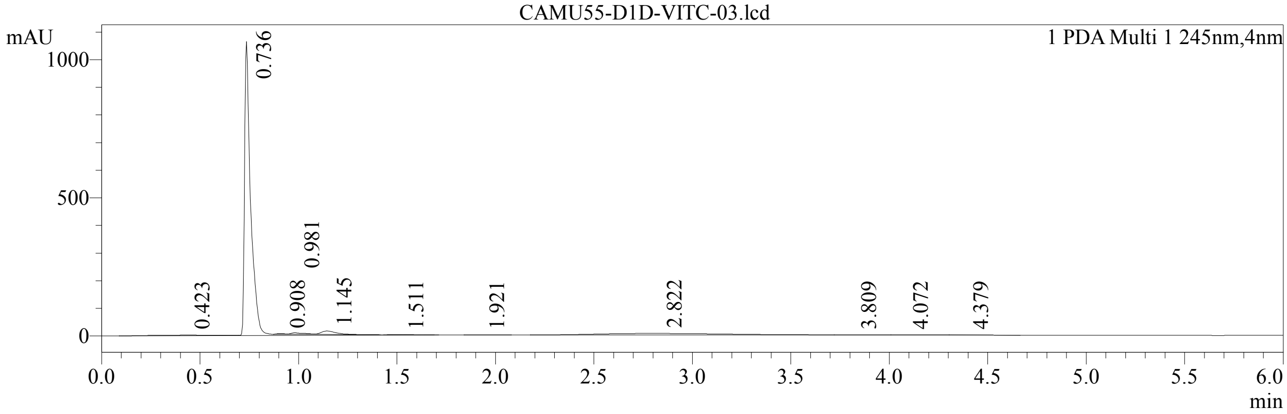
CAMU55-D1D-VITC-03.lcd
mAU
1 PDA Multi 1 245nm,4nm
0.736
0.981
0.423
0.908
1.145
1.511
1.921
2.822
3.809
4.072
4.379
min

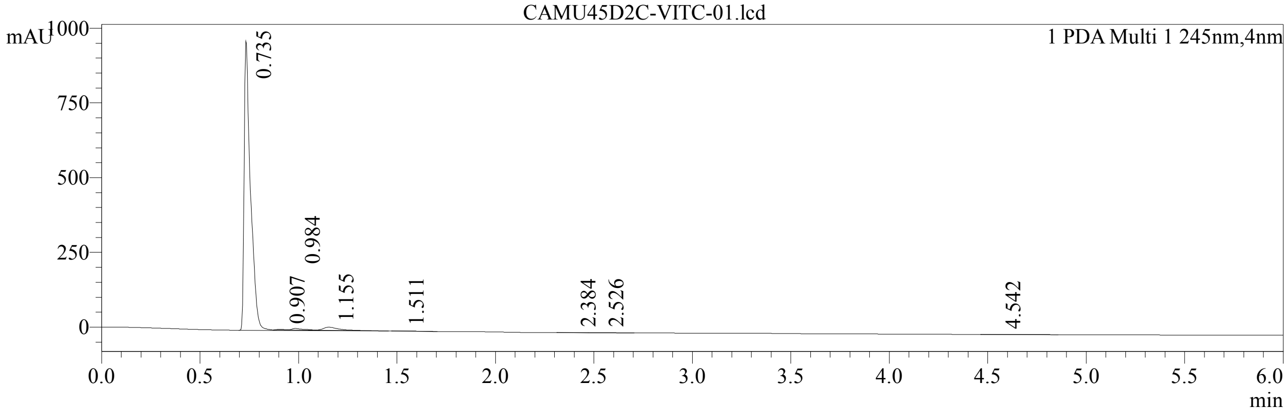
CAMU45D2C-VITC-01.lcd
mAU
1 PDA Multi 1 245nm,4nm
0.735
0.984
0.907
1.155
1.511
2.384
2.526
4.542
min

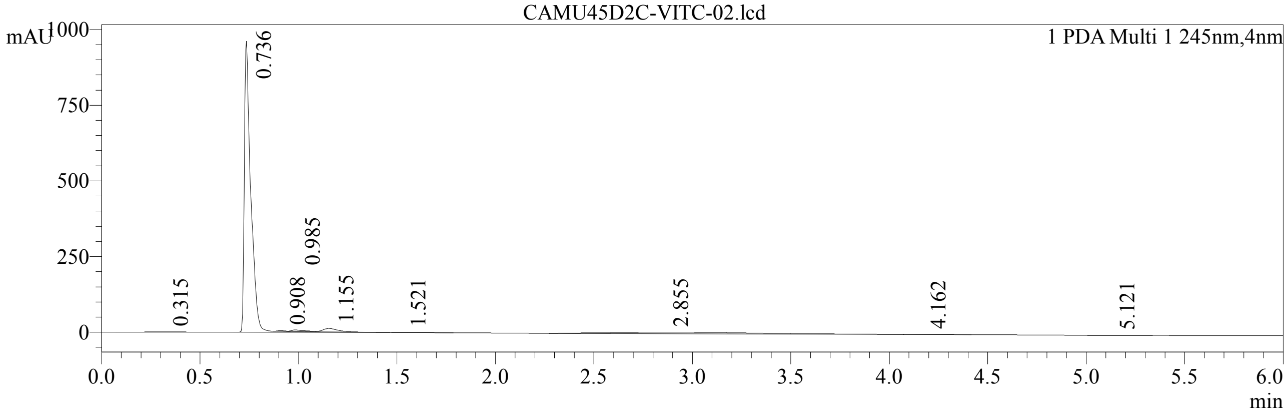
CAMU45D2C-VITC-02.lcd
mAU
1 PDA Multi 1 245nm,4nm
0.736
0.985
0.315
0.908
1.155
1.521
2.855
4.162
5.121
min

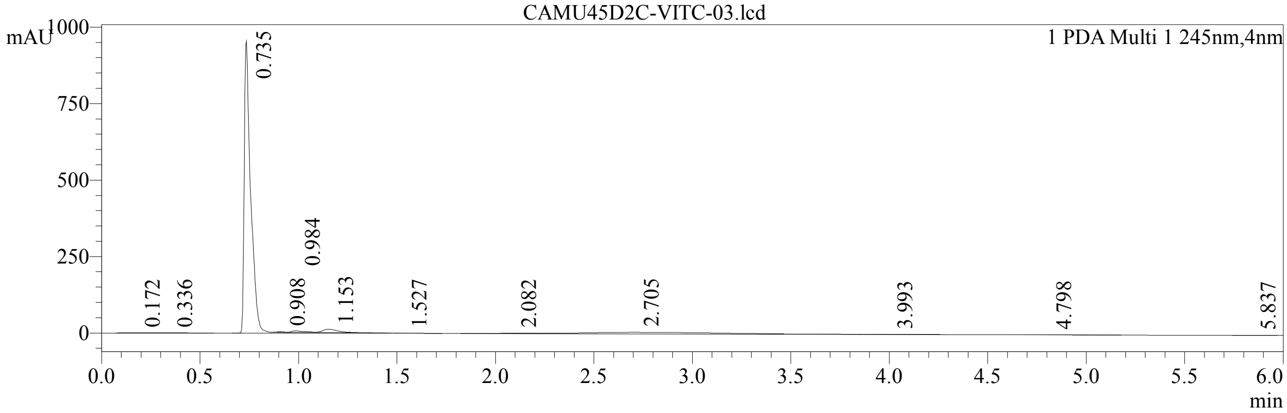
CAMU45D2C-VITC-03.lcd
mAU
1 PDA Multi 1 245nm,4nm
0.735
0.984
0.172
0.336
0.908
1.153
1.527
2.082
2.705
3.993
4.798
5.837
min

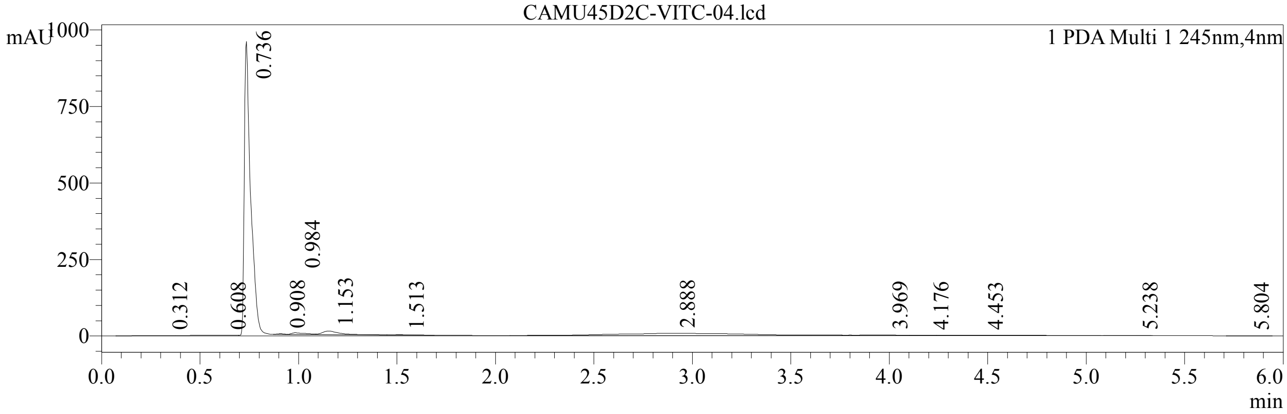
CAMU45D2C-VITC-04.lcd
mAU
1 PDA Multi 1 245nm,4nm
0.736
0.984
0.312
0.608
0.908
1.153
1.513
2.888
3.969
4.176
4.453
5.238
5.804
min

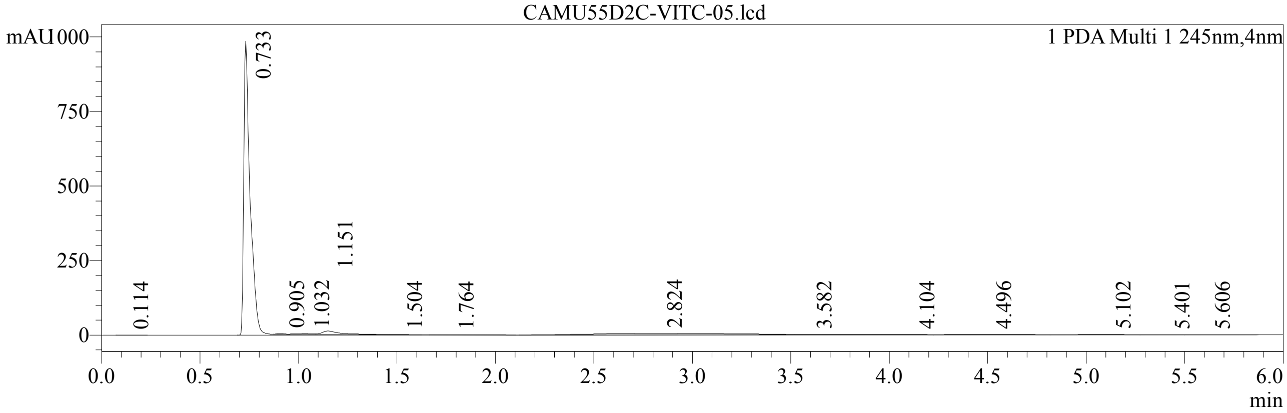
CAMU55D2C-VITC-05.lcd
mAU
1 PDA Multi 1 245nm,4nm
0.733
1.151
0.114
0.905
1.032
1.504
1.764
2.824
3.582
4.104
4.496
5.102
5.401
5.606
min

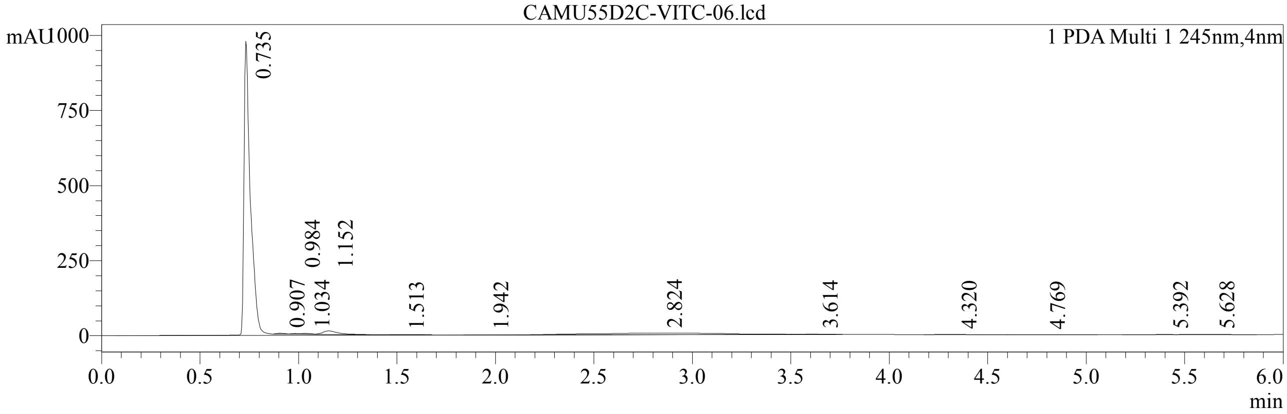
CAMU55D2C-VITC-06.lcd
mAU
1 PDA Multi 1 245nm,4nm
0.735
0.984
1.152
0.907
1.034
1.513
1.942
2.824
3.614
4.320
4.769
5.392
5.628
min

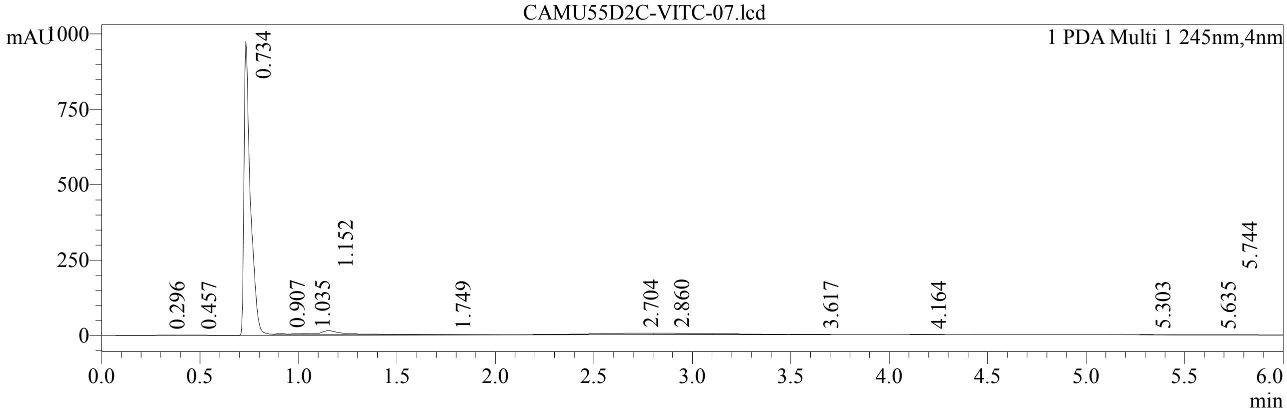
CAMU55D2C-VITC-07.lcd
mAU
1 PDA Multi 1 245nm,4nm
0.734
1.152
5.744
0.296
0.457
0.907
1.035
1.749
2.704
2.860
3.617
4.164
5.303
5.635
min

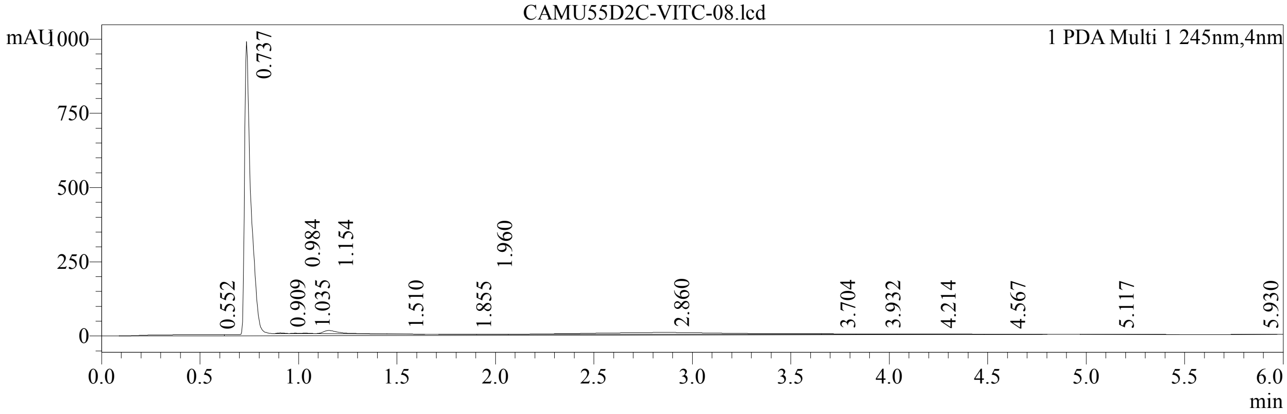
CAMU55D2C-VITC-08.lcd
mAU
1 PDA Multi 1 245nm,4nm
0.737
0.984
1.154
1.960
0.552
0.909
1.035
1.510
1.855
2.860
3.704
3.932
4.214
4.567
5.117
5.930
min

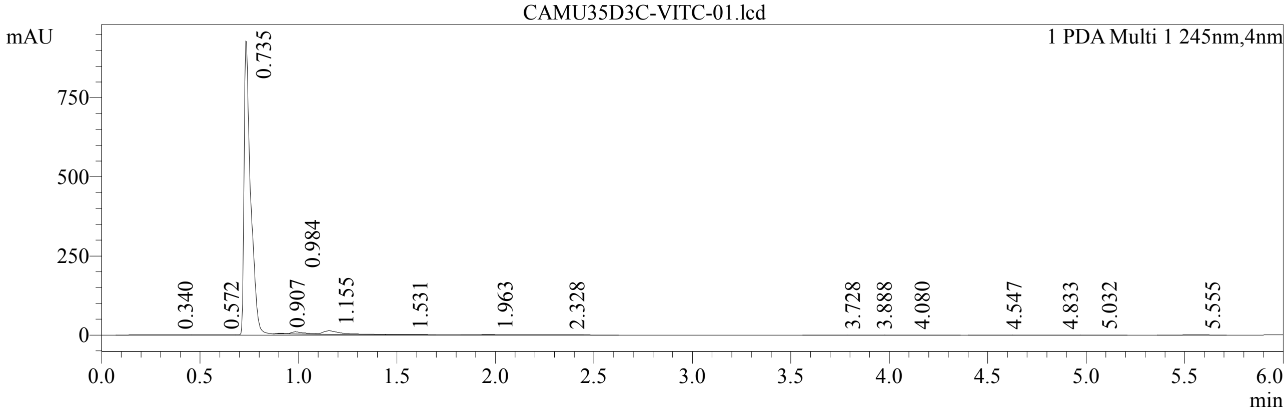
CAMU35D3C-VITC-01.lcd
mAU
1 PDA Multi 1 245nm,4nm
0.735
0.984
0.340
0.572
0.907
1.155
1.531
1.963
2.328
3.728
3.888
4.080
4.547
4.833
5.032
5.555
min

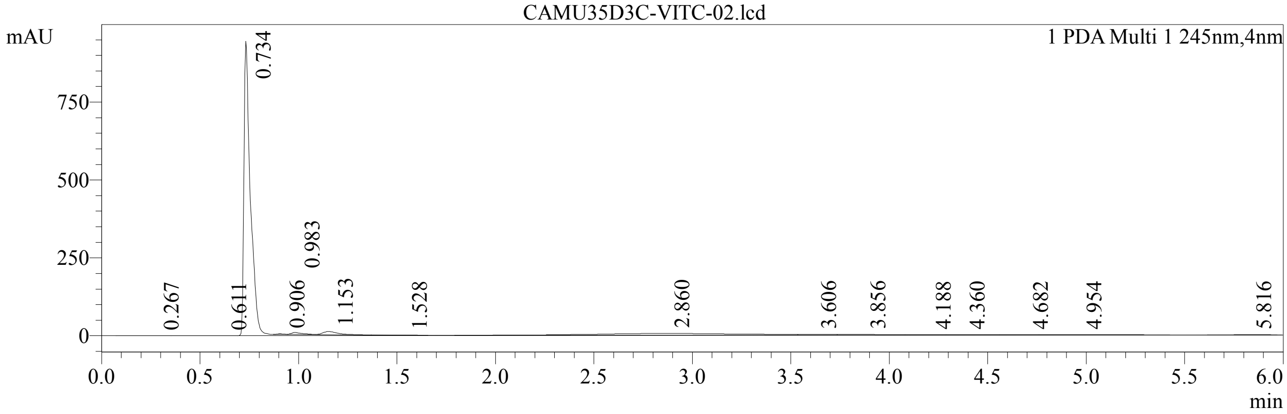
CAMU35D3C-VITC-02.lcd
mAU
1 PDA Multi 1 245nm,4nm
0.734
0.983
0.267
0.611
0.906
1.153
1.528
2.860
3.606
3.856
4.188
4.360
4.682
4.954
5.816
min

CAMU35D3C-VITC-03.lcd

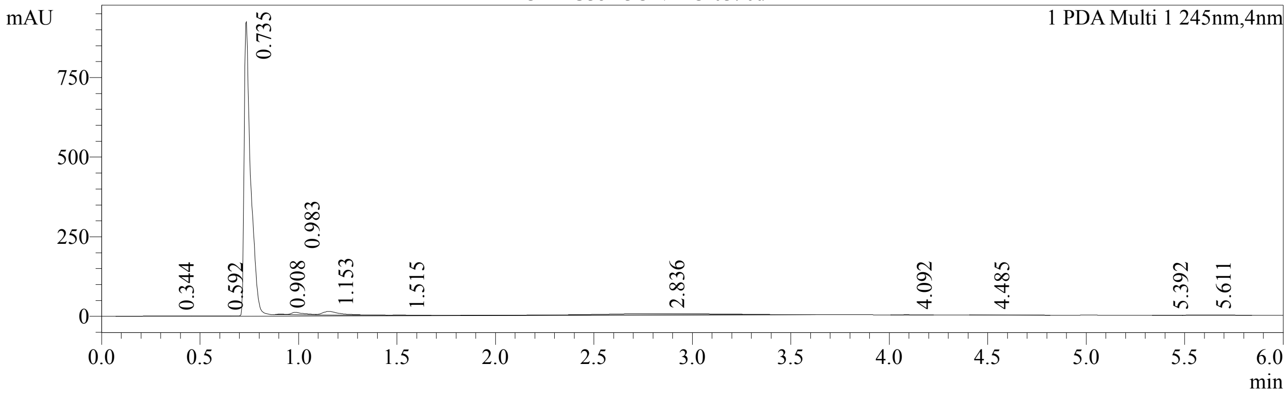

CAMU35D3C-VITC-04.lcd

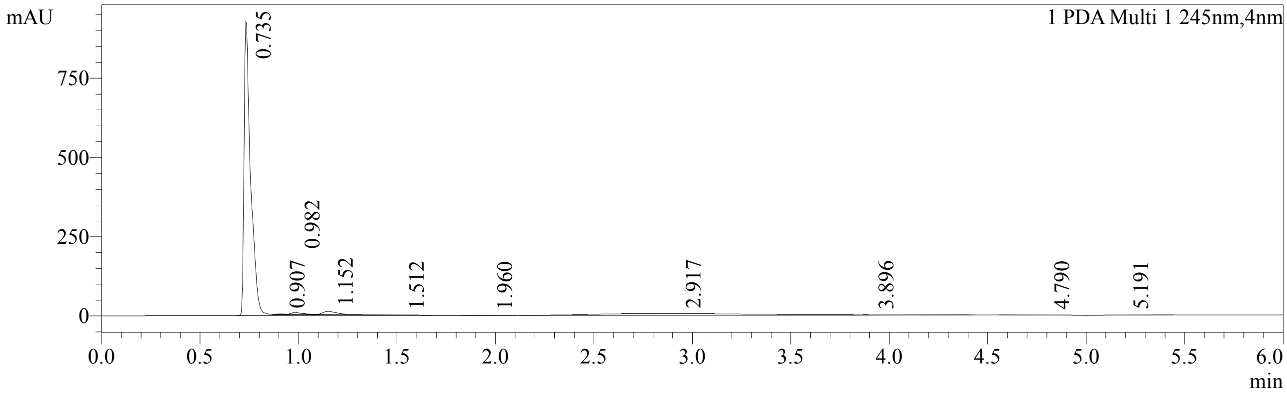

CAMU55D3C-VITC-05.lcd

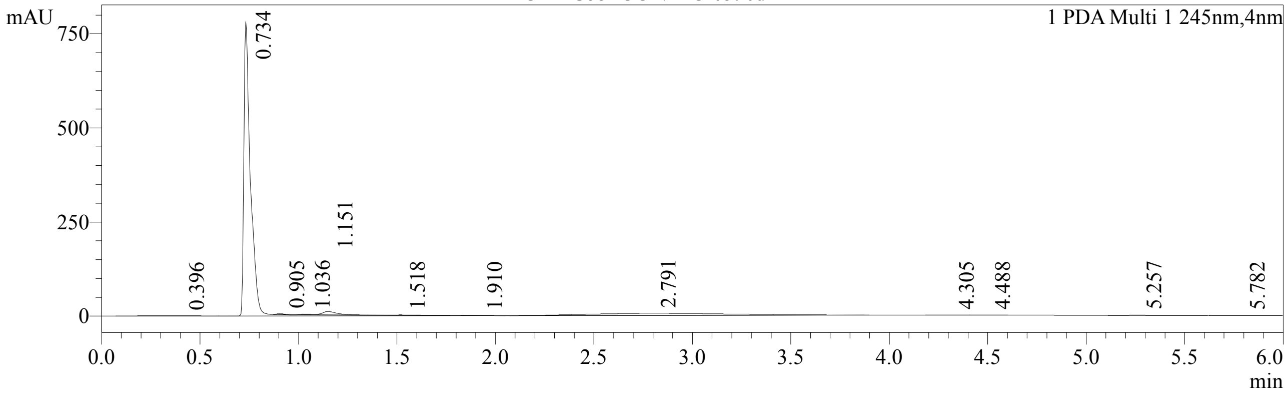

CAMU55D3C-VITC-06.lcd

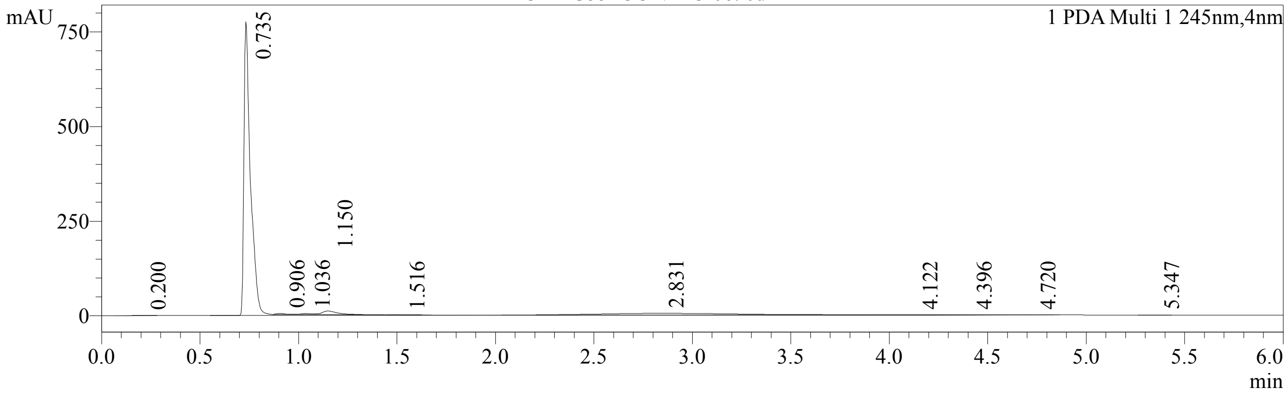

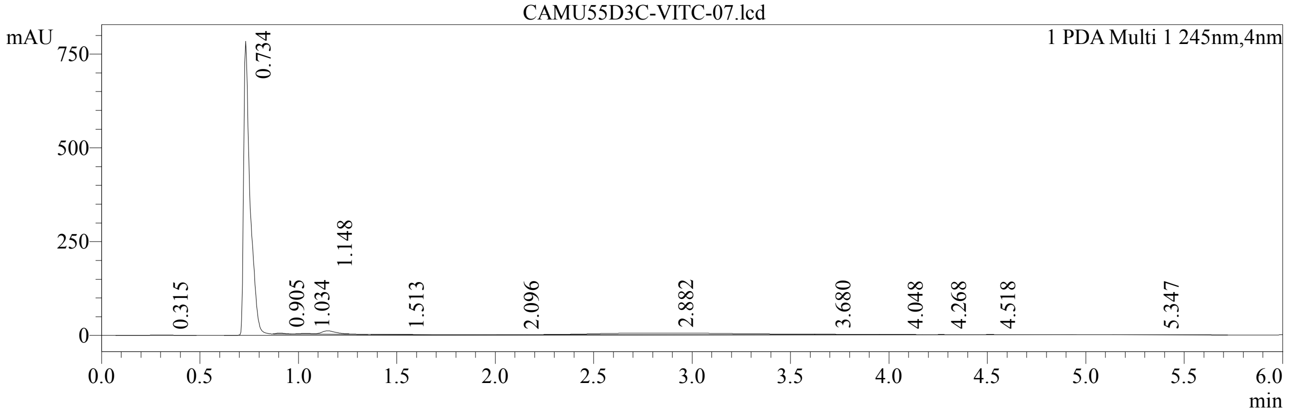
CAMU55D3C-VITC-07.lcd
mAU
1 PDA Multi 1 245nm,4nm
0.734
1.148
0.315
0.905
1.034
1.513
2.096
2.882
3.680
4.048
4.268
4.518
5.347
min

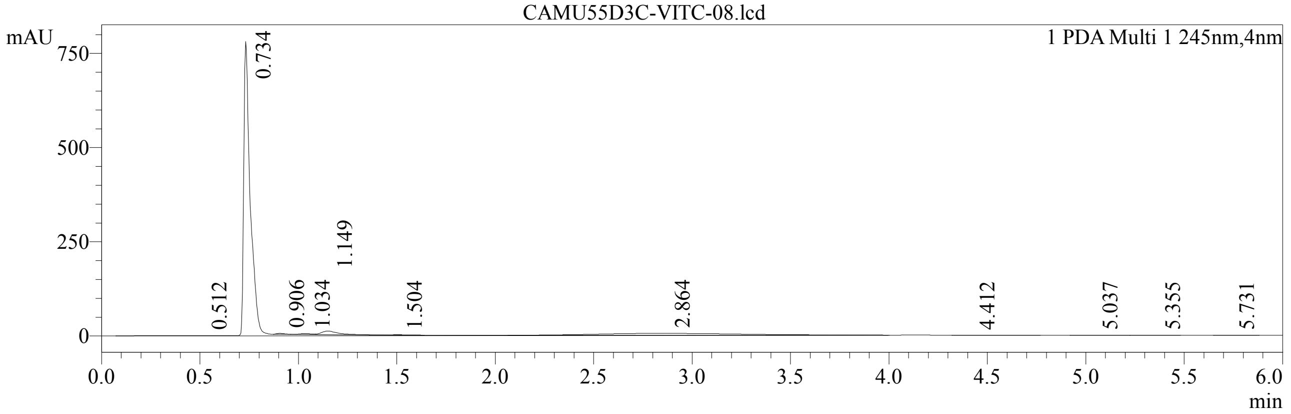
CAMU55D3C-VITC-08.lcd
mAU
1 PDA Multi 1 245nm,4nm
0.734
1.149
0.512
0.906
1.034
1.504
2.864
4.412
5.037
5.355
5.731
min

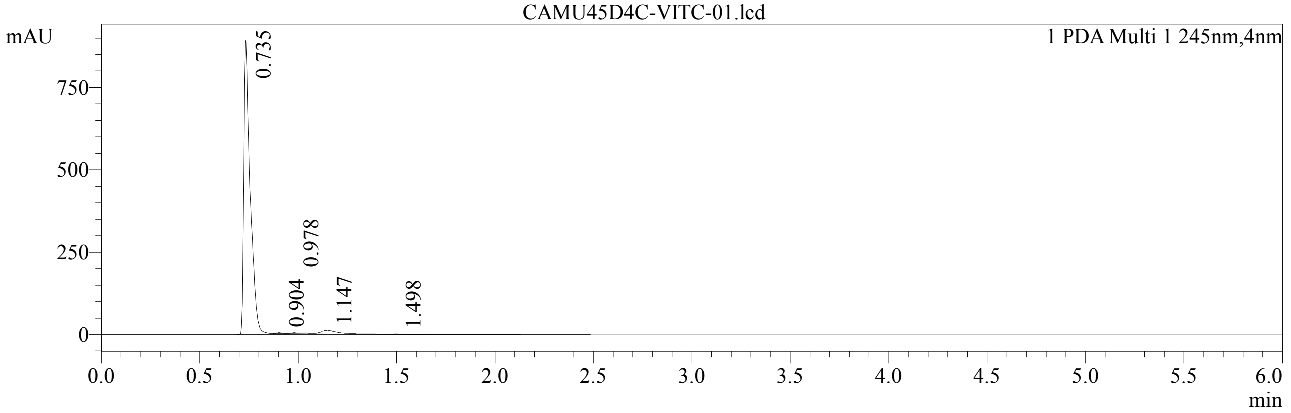
CAMU45D4C-VITC-01.lcd
mAU
1 PDA Multi 1 245nm,4nm
0.735
0.978
0.904
1.147
1.498
min

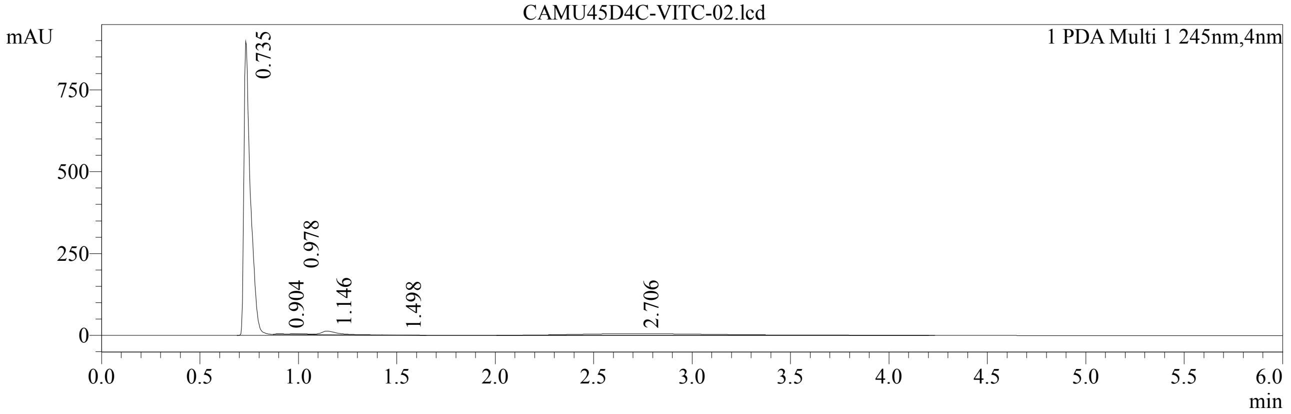
CAMU45D4C-VITC-02.lcd
mAU
1 PDA Multi 1 245nm,4nm
0.735
0.978
0.904
1.146
1.498
2.706
min

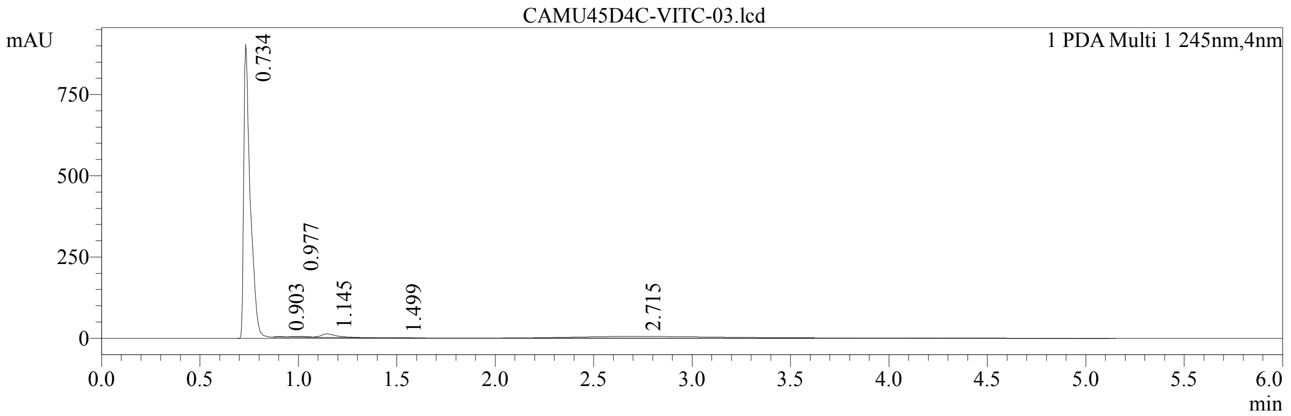
CAMU45D4C-VITC-03.lcd
mAU
1 PDA Multi 1 245nm,4nm
0.734
0.903
0.977
1.145
1.499
2.715
min

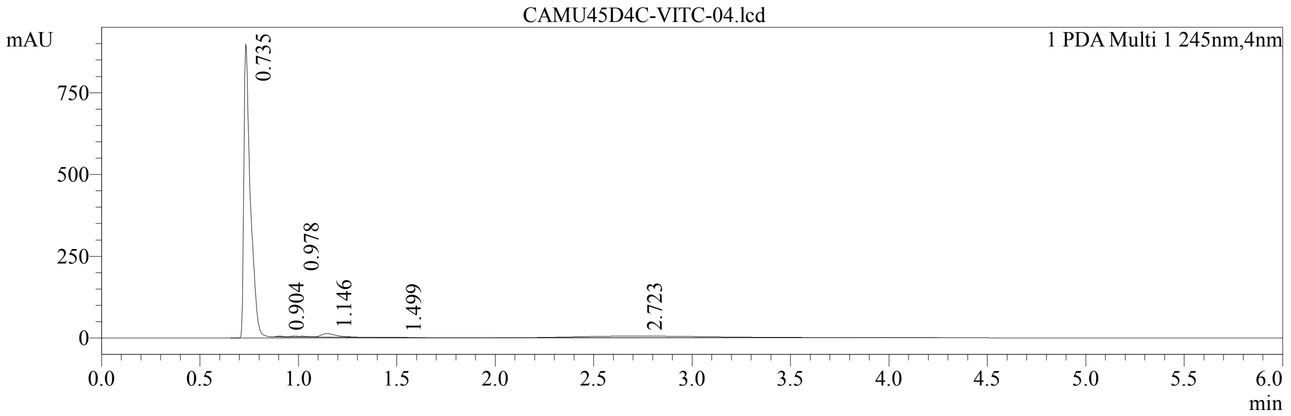
CAMU45D4C-VITC-04.lcd
mAU
1 PDA Multi 1 245nm,4nm
0.735
0.904
0.978
1.146
1.499
2.723
min

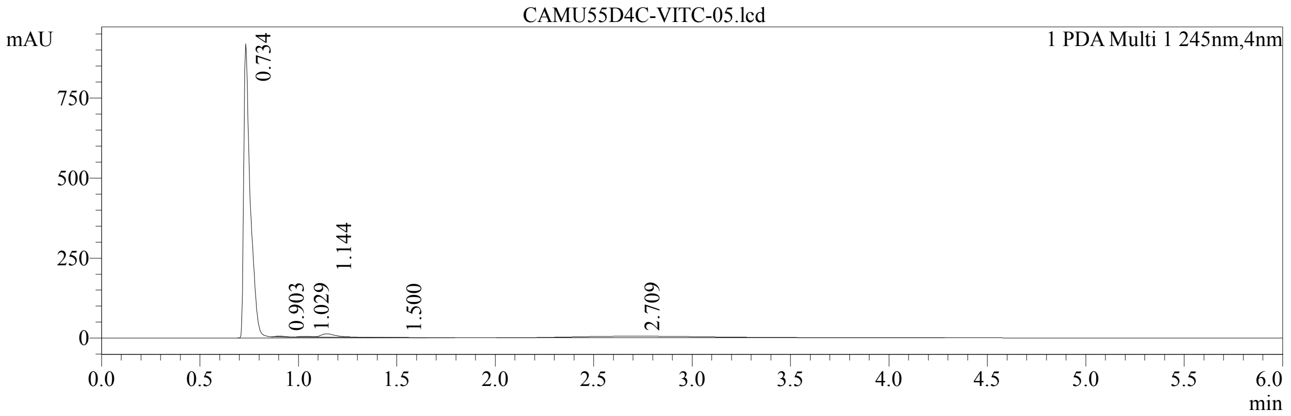
CAMU55D4C-VITC-05.lcd
mAU
1 PDA Multi 1 245nm,4nm
0.734
0.903
1.029
1.144
1.500
2.709
min

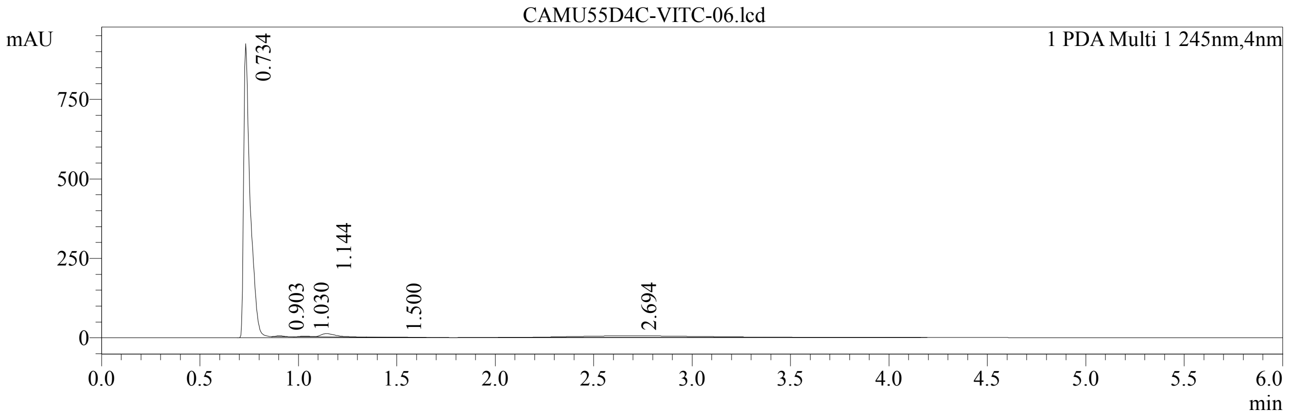
CAMU55D4C-VITC-06.lcd
mAU
1 PDA Multi 1 245nm,4nm
0.734
0.903
1.030
1.144
1.500
2.694
min

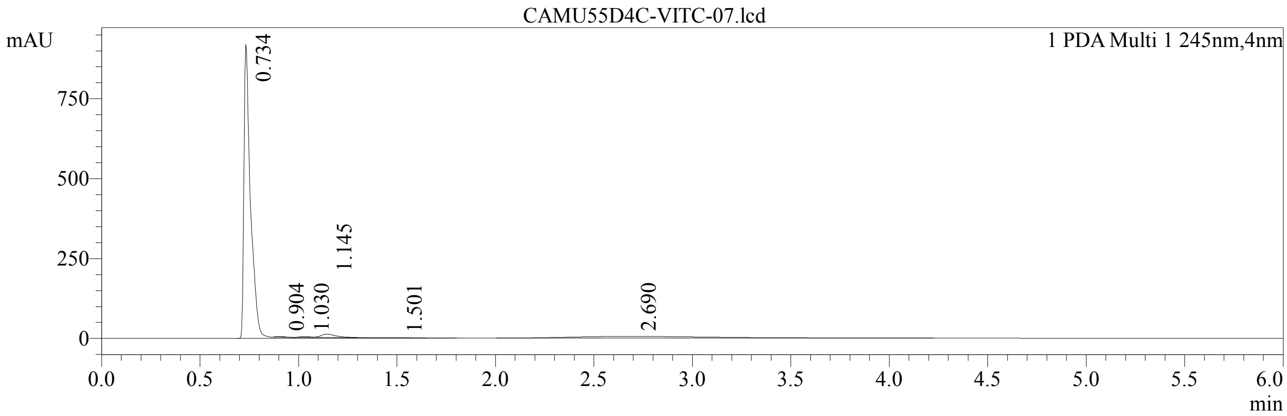
CAMU55D4C-VITC-07.lcd
mAU
1 PDA Multi 1 245nm,4nm
0.734
0.904
1.030
1.145
1.501
2.690
750
500
250
0
0.0 0.5 1.0 1.5 2.0 2.5 3.0 3.5 4.0 4.5 5.0 5.5 6.0
min

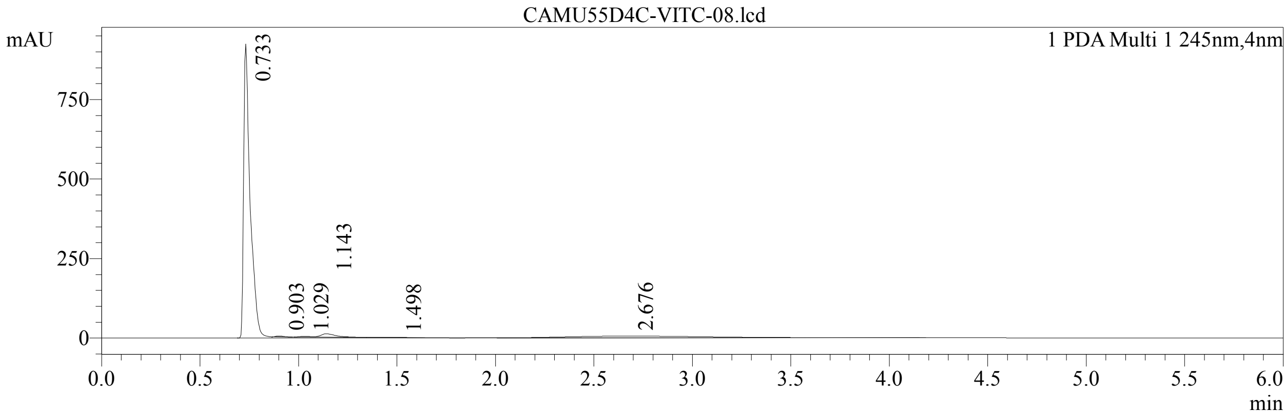
CAMU55D4C-VITC-08.lcd
mAU
1 PDA Multi 1 245nm,4nm
0.733
0.903
1.029
1.143
1.498
2.676
750
500
250
0
0.0 0.5 1.0 1.5 2.0 2.5 3.0 3.5 4.0 4.5 5.0 5.5 6.0
min

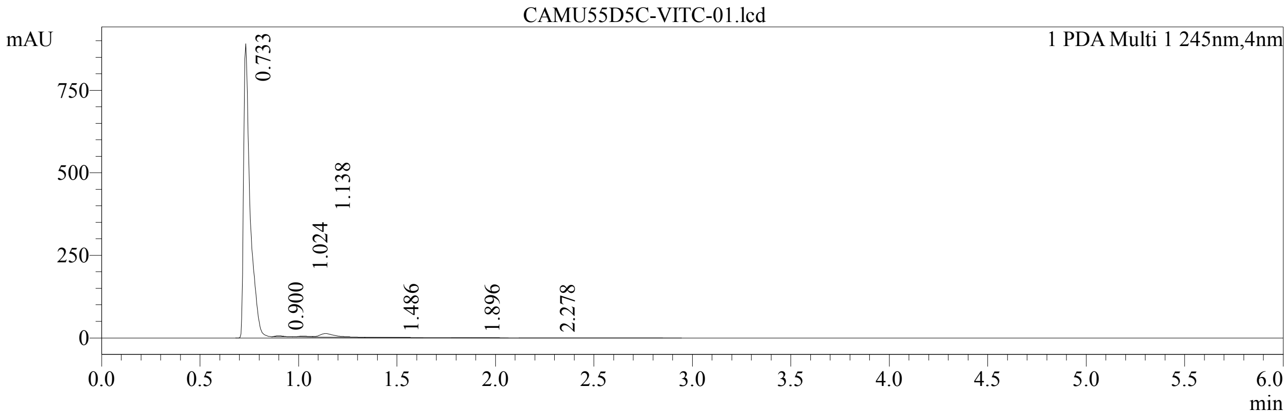
CAMU55D5C-VITC-01.lcd
mAU
1 PDA Multi 1 245nm,4nm
0.733
0.900
1.024
1.138
1.486
1.896
2.278
750
500
250
0
0.0 0.5 1.0 1.5 2.0 2.5 3.0 3.5 4.0 4.5 5.0 5.5 6.0
min

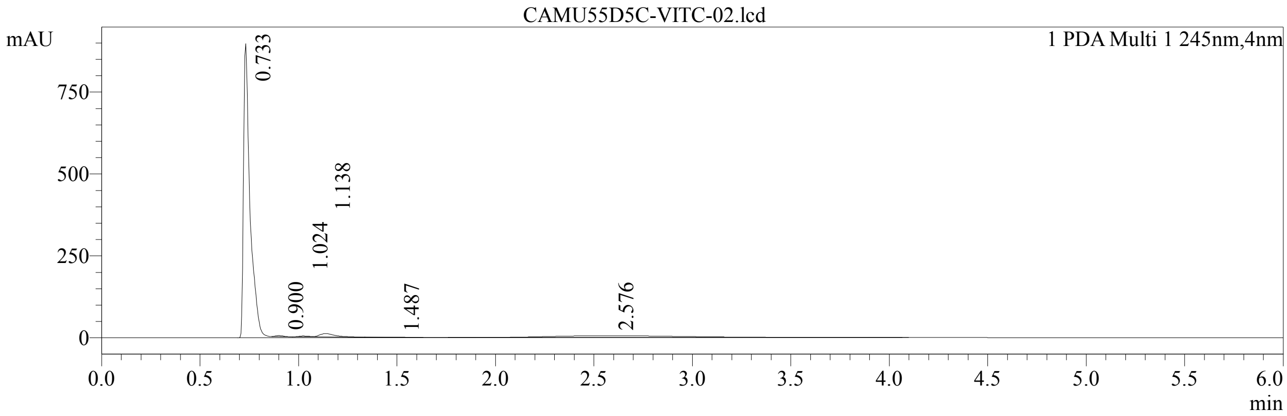
CAMU55D5C-VITC-02.lcd
mAU
1 PDA Multi 1 245nm,4nm
0.733
0.900
1.024
1.138
1.487
2.576
750
500
250
0
0.0 0.5 1.0 1.5 2.0 2.5 3.0 3.5 4.0 4.5 5.0 5.5 6.0
min

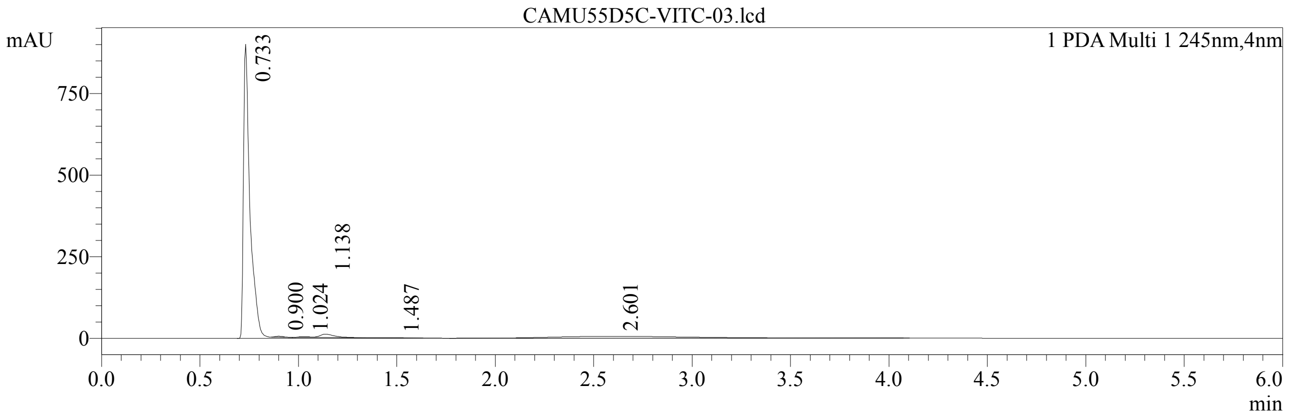
CAMU55D5C-VITC-03.lcd
mAU
1 PDA Multi 1 245nm,4nm
0.733
0.900
1.024
1.138
1.487
2.601
min

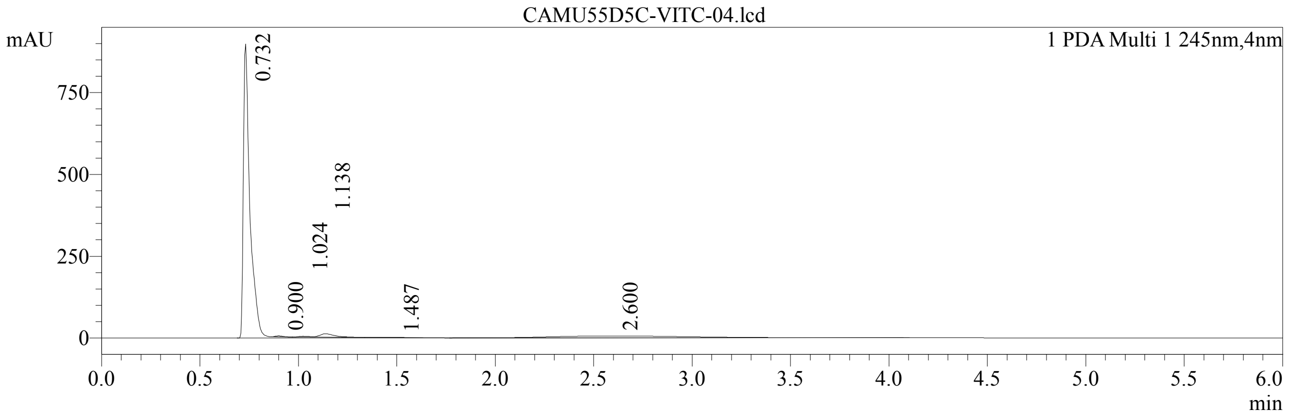
CAMU55D5C-VITC-04.lcd
mAU
1 PDA Multi 1 245nm,4nm
0.732
0.900
1.024
1.138
1.487
2.600
min

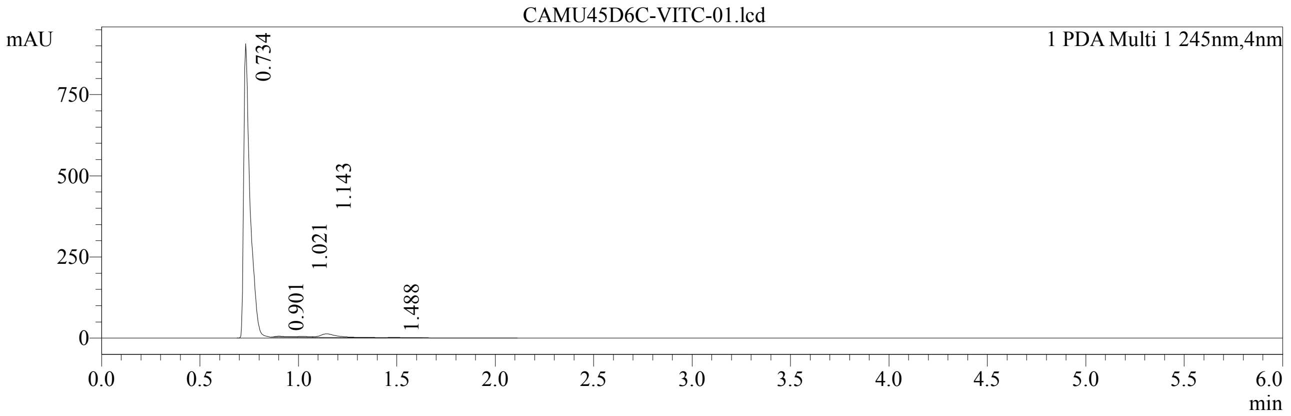
CAMU45D6C-VITC-01.lcd
mAU
1 PDA Multi 1 245nm,4nm
0.734
0.901
1.021
1.143
1.488
min

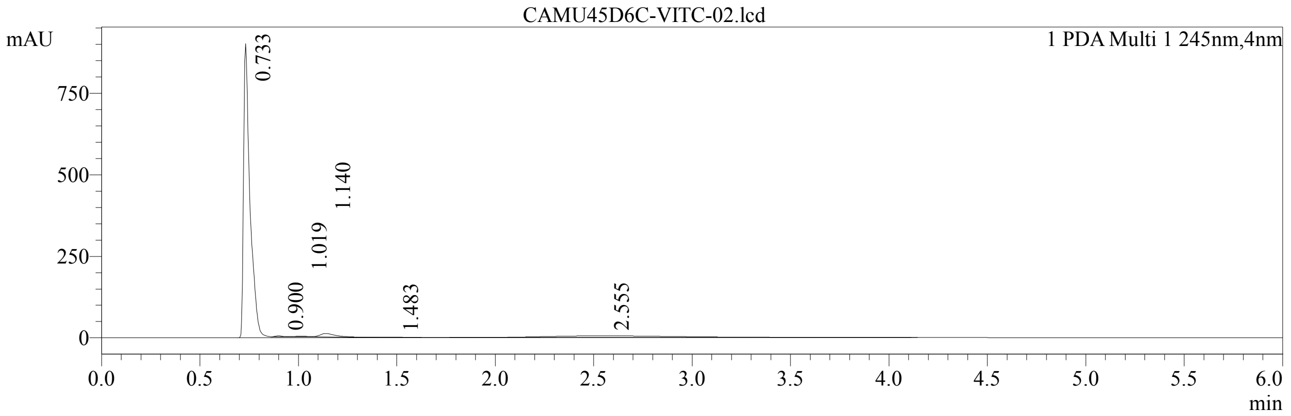
CAMU45D6C-VITC-02.lcd
mAU
1 PDA Multi 1 245nm,4nm
0.733
0.900
1.019
1.140
1.483
2.555
min

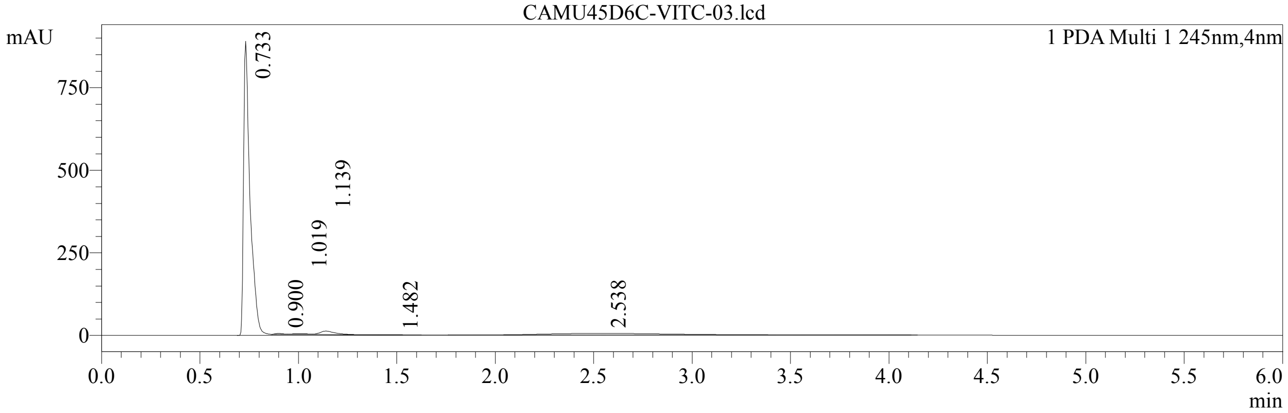
CAMU45D6C-VITC-03.lcd
mAU
1 PDA Multi 1 245nm,4nm
0.733
1.139
1.019
0.900
1.482
2.538
750
500
250
0
0.0 0.5 1.0 1.5 2.0 2.5 3.0 3.5 4.0 4.5 5.0 5.5 6.0
min

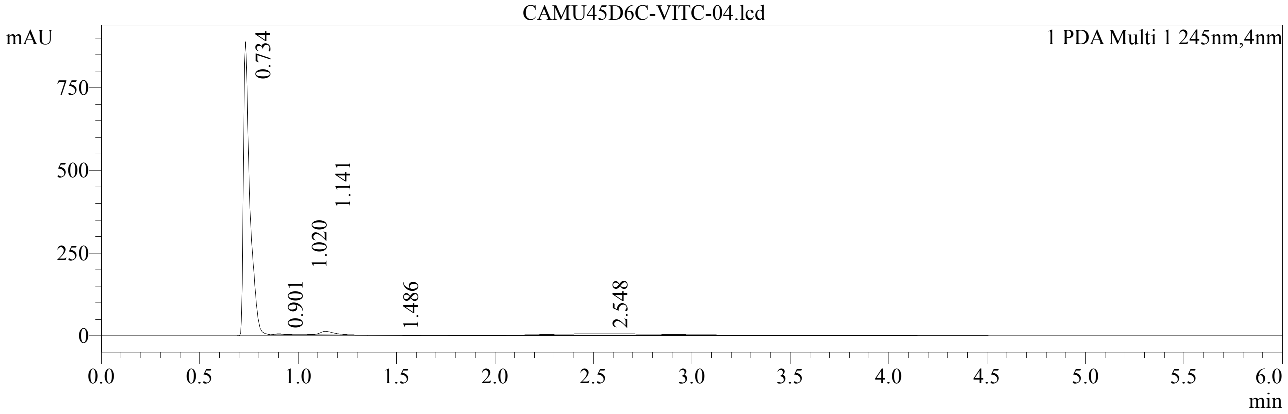
CAMU45D6C-VITC-04.lcd
mAU
1 PDA Multi 1 245nm,4nm
0.734
1.141
1.020
0.901
1.486
2.548
750
500
250
0
0.0 0.5 1.0 1.5 2.0 2.5 3.0 3.5 4.0 4.5 5.0 5.5 6.0
min

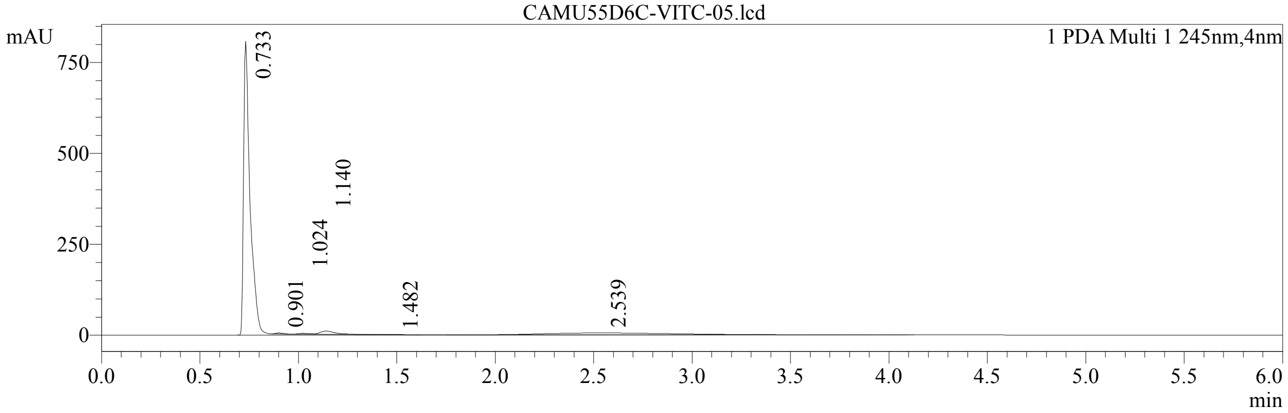
CAMU55D6C-VITC-05.lcd
mAU
1 PDA Multi 1 245nm,4nm
0.733
1.140
1.024
0.901
1.482
2.539
750
500
250
0
0.0 0.5 1.0 1.5 2.0 2.5 3.0 3.5 4.0 4.5 5.0 5.5 6.0
min

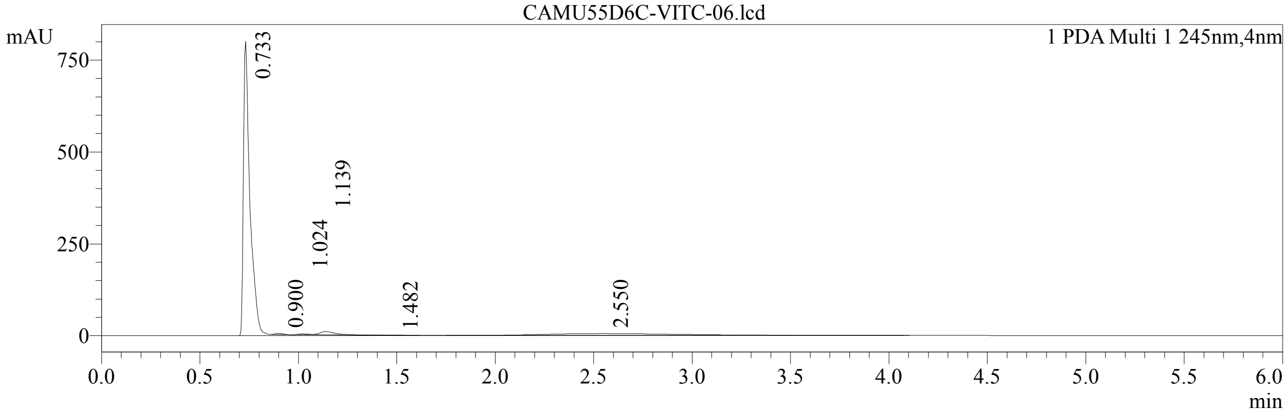
CAMU55D6C-VITC-06.lcd
mAU
1 PDA Multi 1 245nm,4nm
0.733
1.139
1.024
0.900
1.482
2.550
750
500
250
0
0.0 0.5 1.0 1.5 2.0 2.5 3.0 3.5 4.0 4.5 5.0 5.5 6.0
min

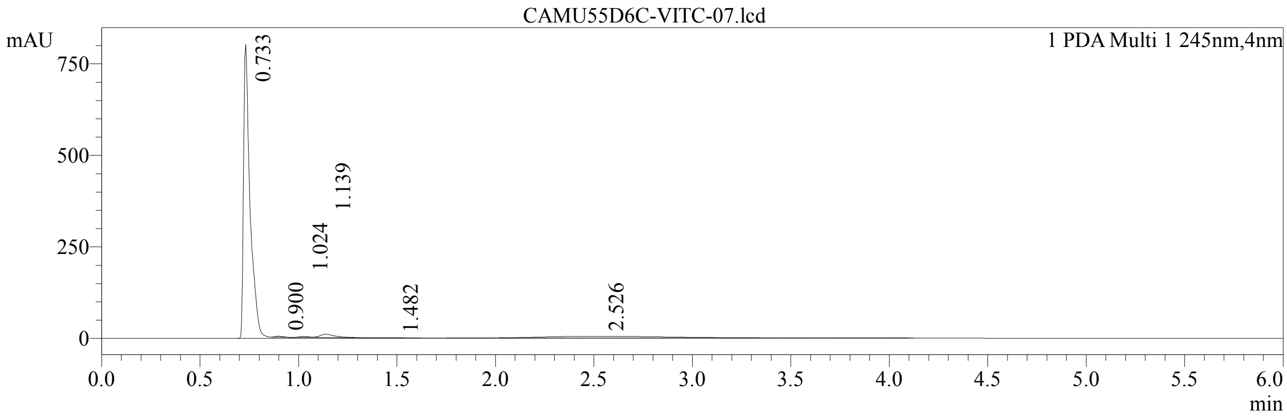
CAMU55D6C-VITC-07.lcd
mAU
1 PDA Multi 1 245nm,4nm
0.733
0.900
1.024
1.139
1.482
2.526
750
500
250
0
0.0 0.5 1.0 1.5 2.0 2.5 3.0 3.5 4.0 4.5 5.0 5.5 6.0
min

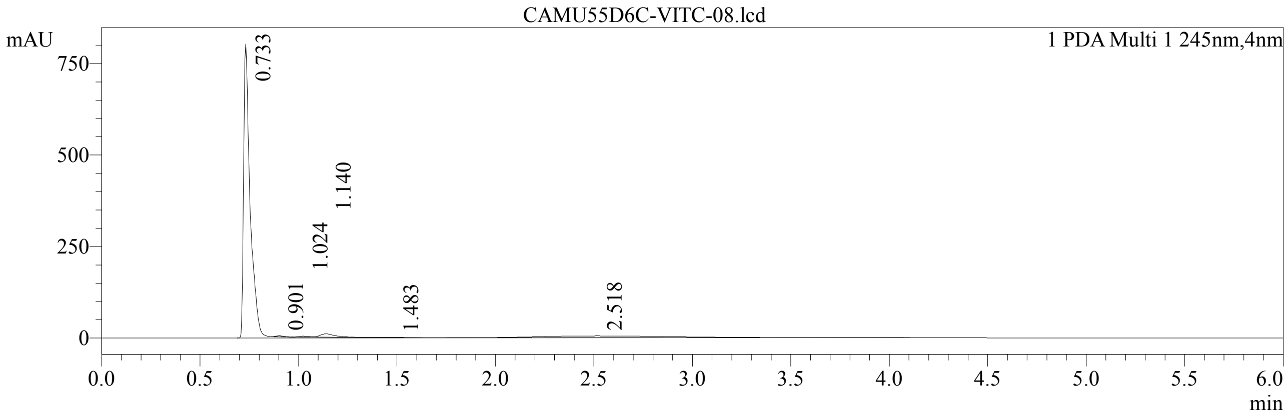
CAMU55D6C-VITC-08.lcd
mAU
1 PDA Multi 1 245nm,4nm
0.733
0.901
1.024
1.140
1.483
2.518
750
500
250
0
0.0 0.5 1.0 1.5 2.0 2.5 3.0 3.5 4.0 4.5 5.0 5.5 6.0
min

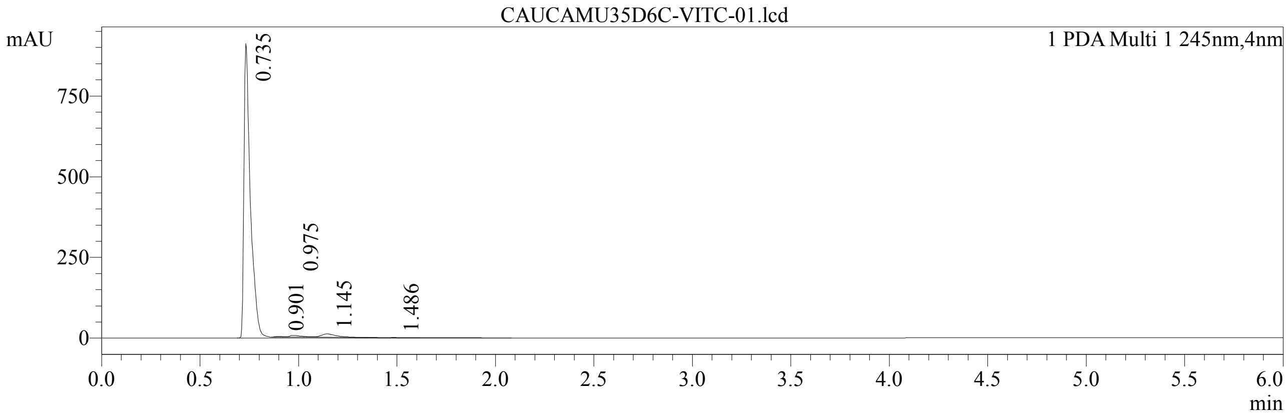
CAUCAMU35D6C-VITC-01.lcd
mAU
1 PDA Multi 1 245nm,4nm
0.735
0.901
0.975
1.145
1.486
750
500
250
0
0.0 0.5 1.0 1.5 2.0 2.5 3.0 3.5 4.0 4.5 5.0 5.5 6.0
min

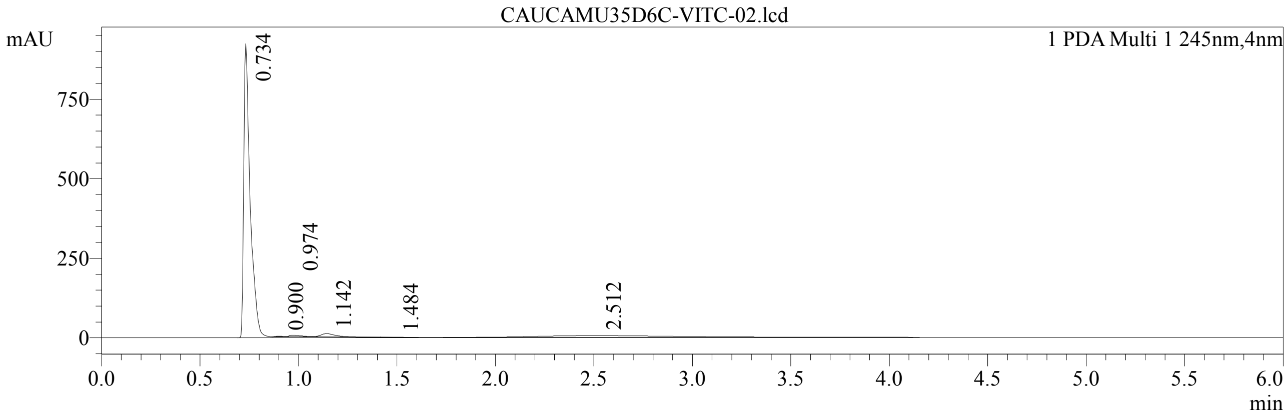
CAUCAMU35D6C-VITC-02.lcd
mAU
1 PDA Multi 1 245nm,4nm
0.734
0.900
0.974
1.142
1.484
2.512
750
500
250
0
0.0 0.5 1.0 1.5 2.0 2.5 3.0 3.5 4.0 4.5 5.0 5.5 6.0
min

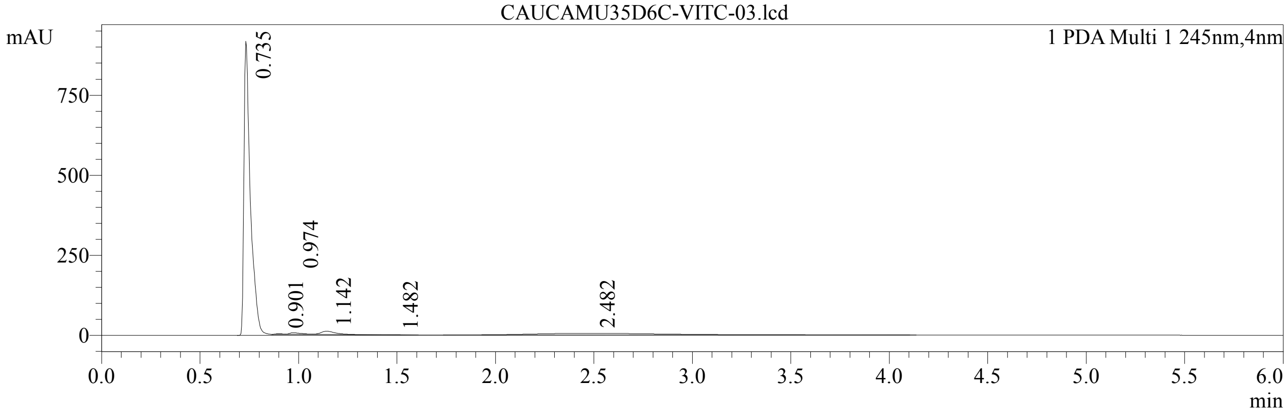
CAUCAMU35D6C-VITC-03.lcd
mAU
1 PDA Multi 1 245nm,4nm
0.735
0.974
0.901
1.142
1.482
2.482
750
500
250
0
0.0
0.5
1.0
1.5
2.0
2.5
3.0
3.5
4.0
4.5
5.0
5.5
6.0
min

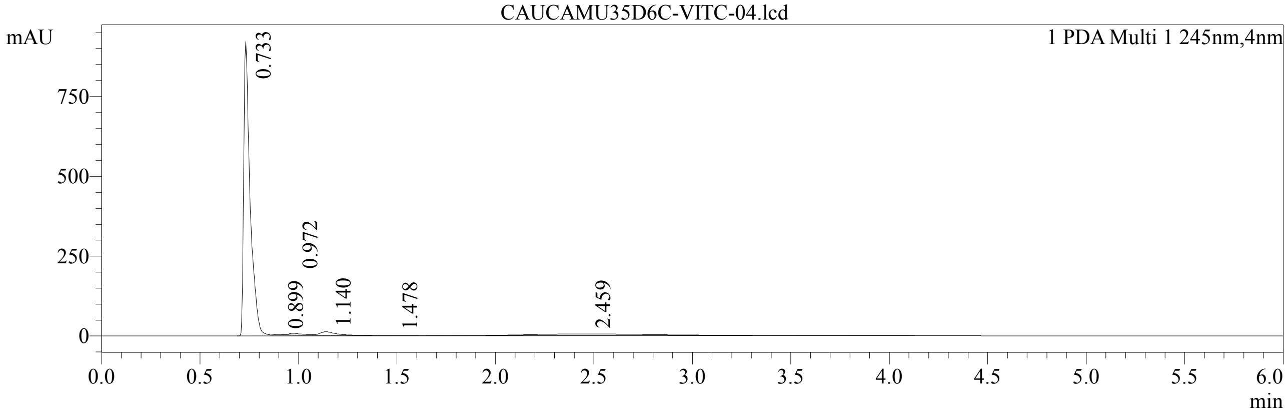
CAUCAMU35D6C-VITC-04.lcd
mAU
1 PDA Multi 1 245nm,4nm
0.733
0.972
0.899
1.140
1.478
2.459
750
500
250
0
0.0
0.5
1.0
1.5
2.0
2.5
3.0
3.5
4.0
4.5
5.0
5.5
6.0
min

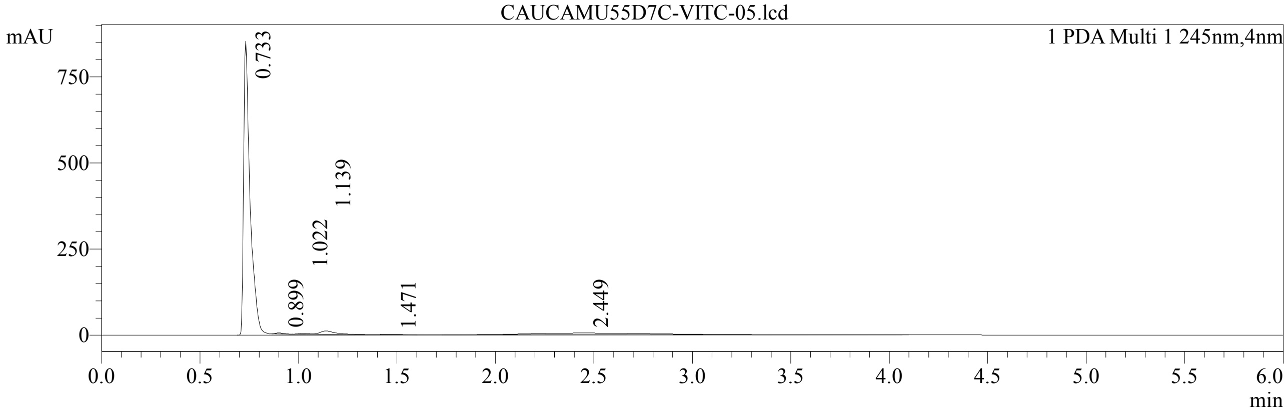
CAUCAMU55D7C-VITC-05.lcd
mAU
1 PDA Multi 1 245nm,4nm
0.733
1.139
1.022
0.899
1.471
2.449
750
500
250
0
0.0
0.5
1.0
1.5
2.0
2.5
3.0
3.5
4.0
4.5
5.0
5.5
6.0
min

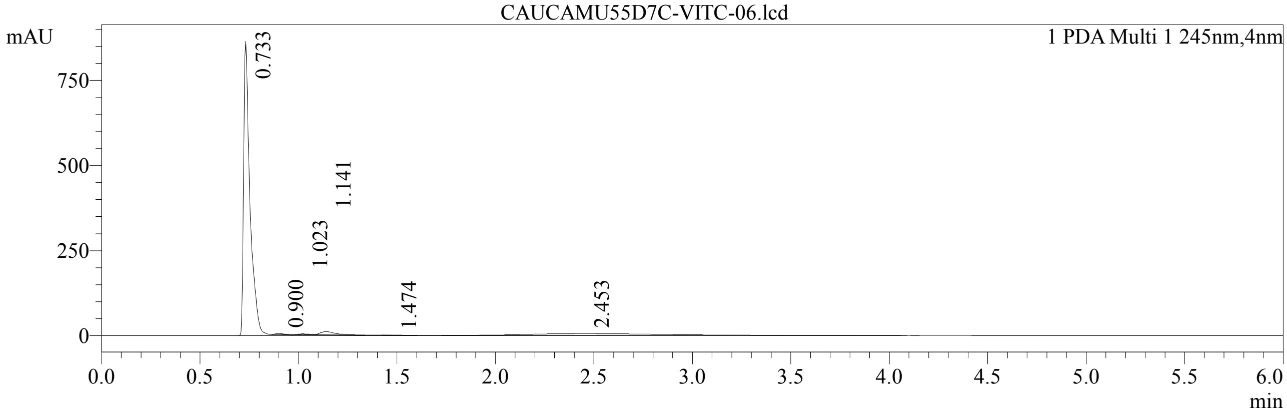
CAUCAMU55D7C-VITC-06.lcd
mAU
1 PDA Multi 1 245nm,4nm
0.733
1.141
1.023
0.900
1.474
2.453
750
500
250
0
0.0
0.5
1.0
1.5
2.0
2.5
3.0
3.5
4.0
4.5
5.0
5.5
6.0
min

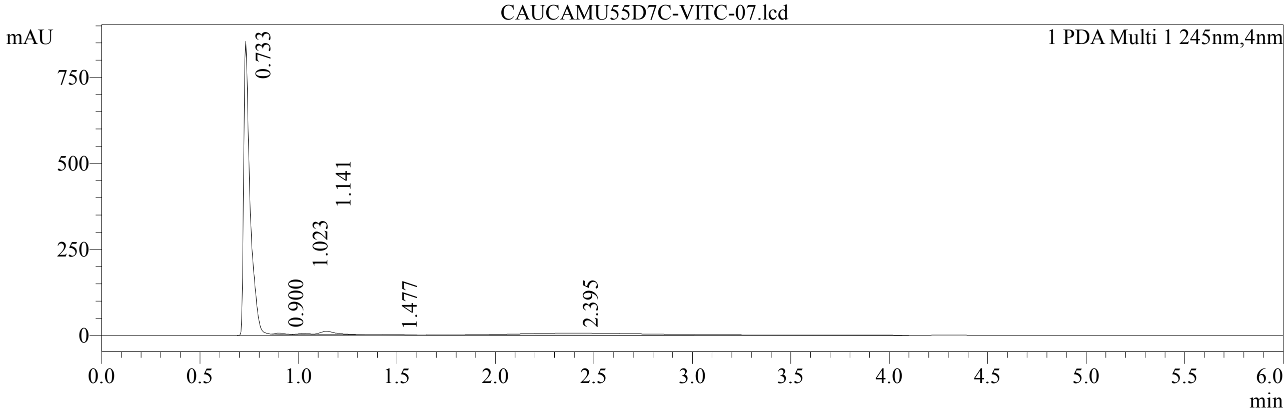
CAUCAMU55D7C-VITC-07.lcd
mAU
1 PDA Multi 1 245nm,4nm
0.733
0.900
1.023
1.141
1.477
2.395
750
500
250
0
0.0
0.5
1.0
1.5
2.0
2.5
3.0
3.5
4.0
4.5
5.0
5.5
6.0
min

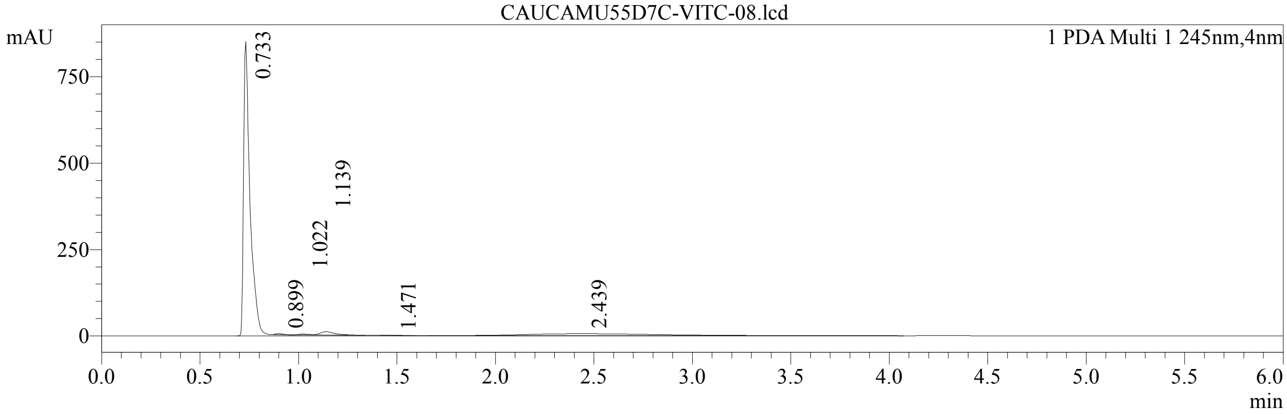
CAUCAMU55D7C-VITC-08.lcd
mAU
1 PDA Multi 1 245nm,4nm
0.733
0.899
1.022
1.139
1.471
2.439
750
500
250
0
0.0
0.5
1.0
1.5
2.0
2.5
3.0
3.5
4.0
4.5
5.0
5.5
6.0
min

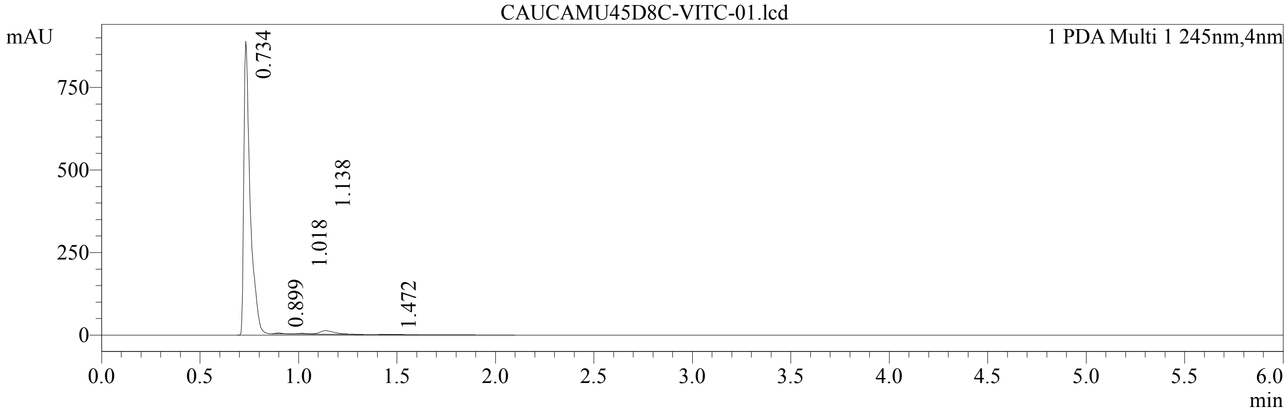
CAUCAMU45D8C-VITC-01.lcd
mAU
1 PDA Multi 1 245nm,4nm
0.734
0.899
1.018
1.138
1.472
750
500
250
0
0.0
0.5
1.0
1.5
2.0
2.5
3.0
3.5
4.0
4.5
5.0
5.5
6.0
min

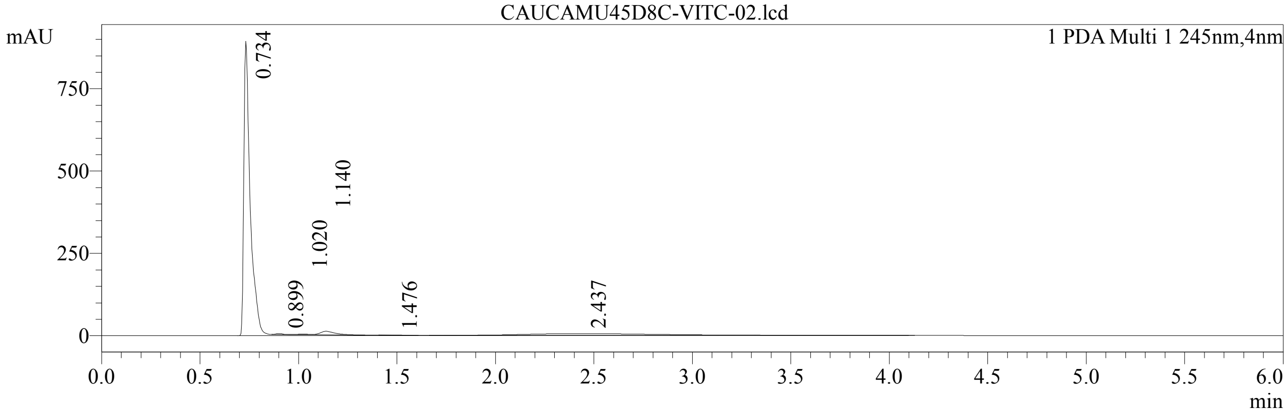
CAUCAMU45D8C-VITC-02.lcd
mAU
1 PDA Multi 1 245nm,4nm
0.734
0.899
1.020
1.140
1.476
2.437
750
500
250
0
0.0
0.5
1.0
1.5
2.0
2.5
3.0
3.5
4.0
4.5
5.0
5.5
6.0
min

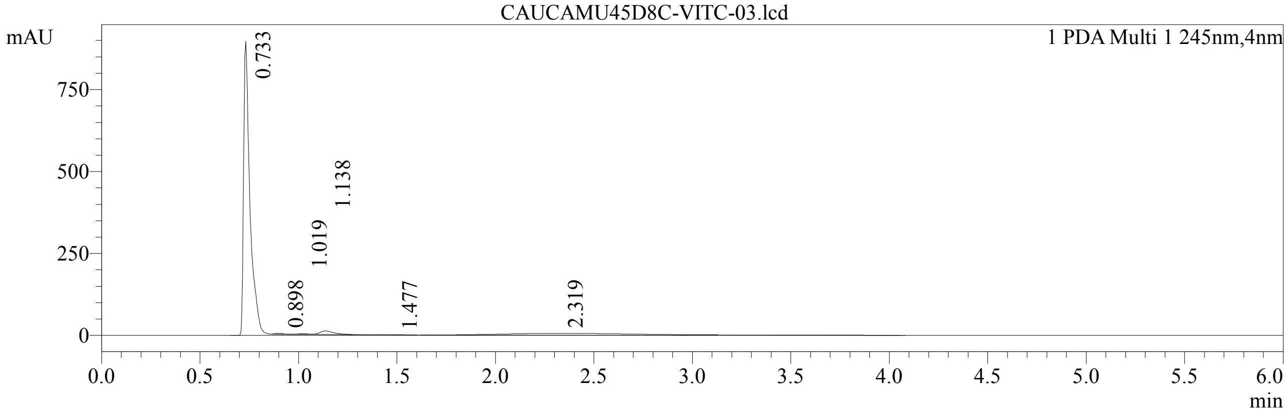
CAUCAMU45D8C-VITC-03.lcd
mAU
1 PDA Multi 1 245nm,4nm
0.733
0.898
1.019
1.138
1.477
2.319
750
500
250
0
0.0
0.5
1.0
1.5
2.0
2.5
3.0
3.5
4.0
4.5
5.0
5.5
6.0
min

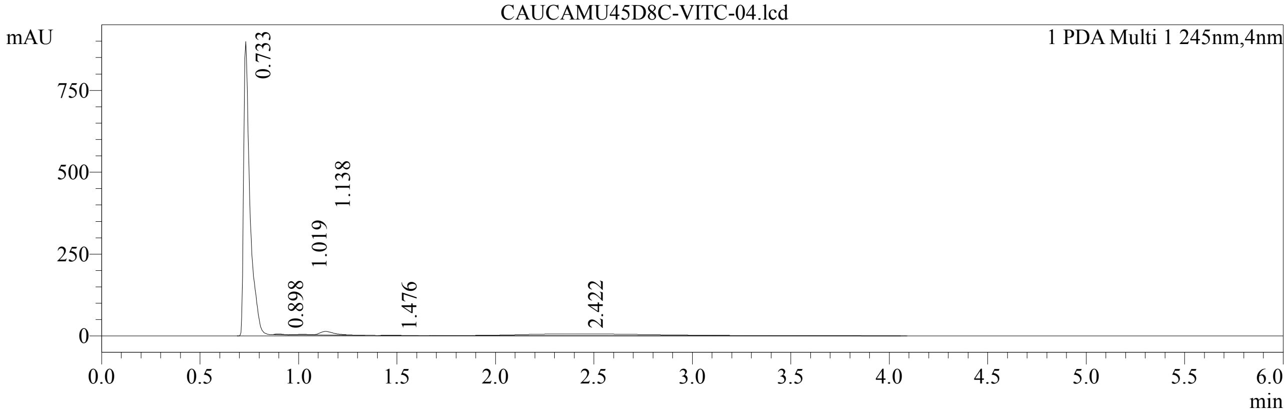
CAUCAMU45D8C-VITC-04.lcd
mAU
1 PDA Multi 1 245nm,4nm
0.733
0.898
1.019
1.138
1.476
2.422
750
500
250
0
0.0
0.5
1.0
1.5
2.0
2.5
3.0
3.5
4.0
4.5
5.0
5.5
6.0
min

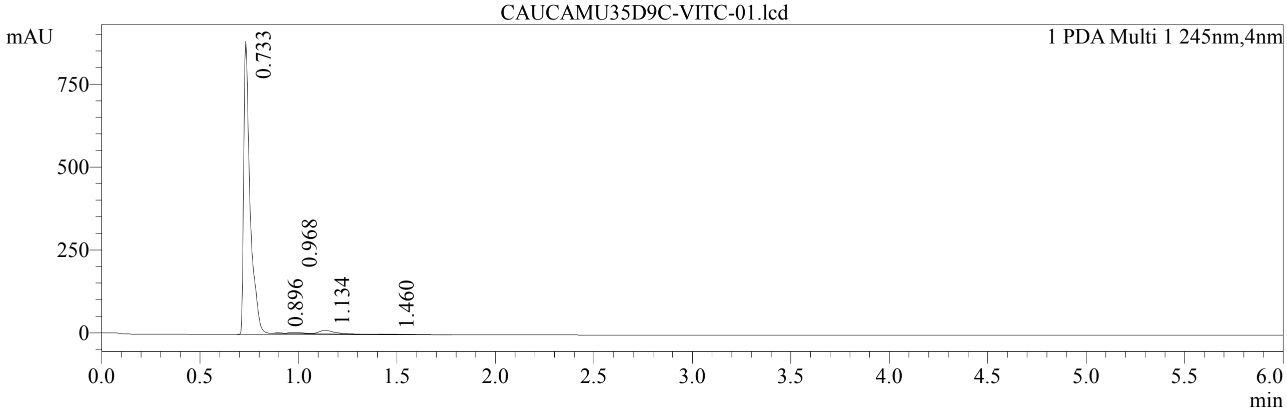
CAUCAMU35D9C-VITC-01.lcd
mAU
1 PDA Multi 1 245nm,4nm
0.733
0.896
0.968
1.134
1.460
750
500
250
0
0.0
0.5
1.0
1.5
2.0
2.5
3.0
3.5
4.0
4.5
5.0
5.5
6.0
min

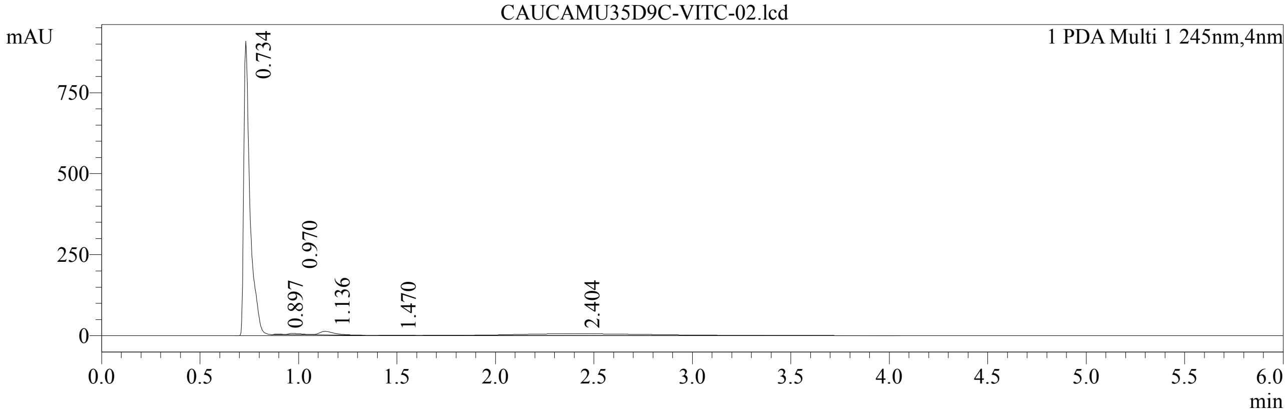
CAUCAMU35D9C-VITC-02.lcd
mAU
1 PDA Multi 1 245nm,4nm
0.734
0.897
0.970
1.136
1.470
2.404
750
500
250
0
0.0
0.5
1.0
1.5
2.0
2.5
3.0
3.5
4.0
4.5
5.0
5.5
6.0
min

CAUCAMU35D9C-VITC-03.lcd

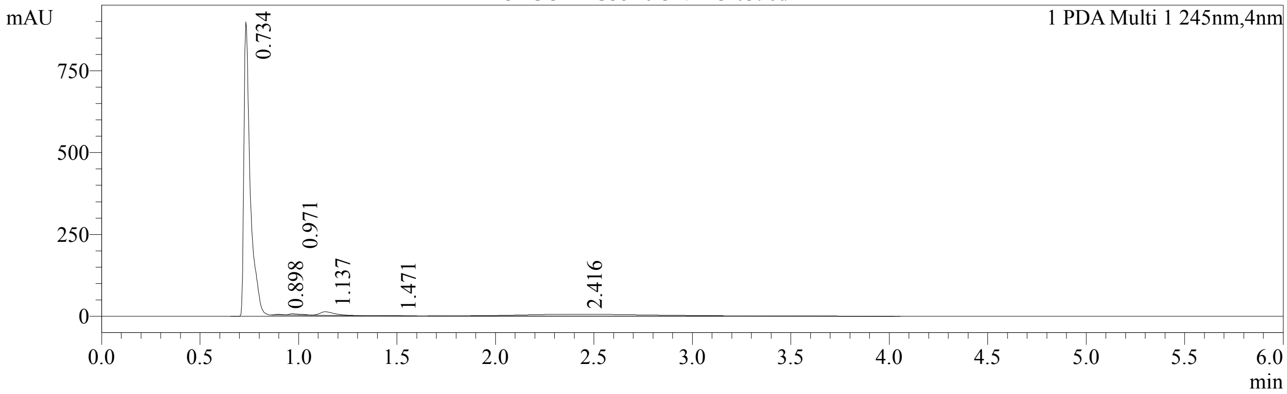

CAUCAMU35D9C-VITC-04.lcd

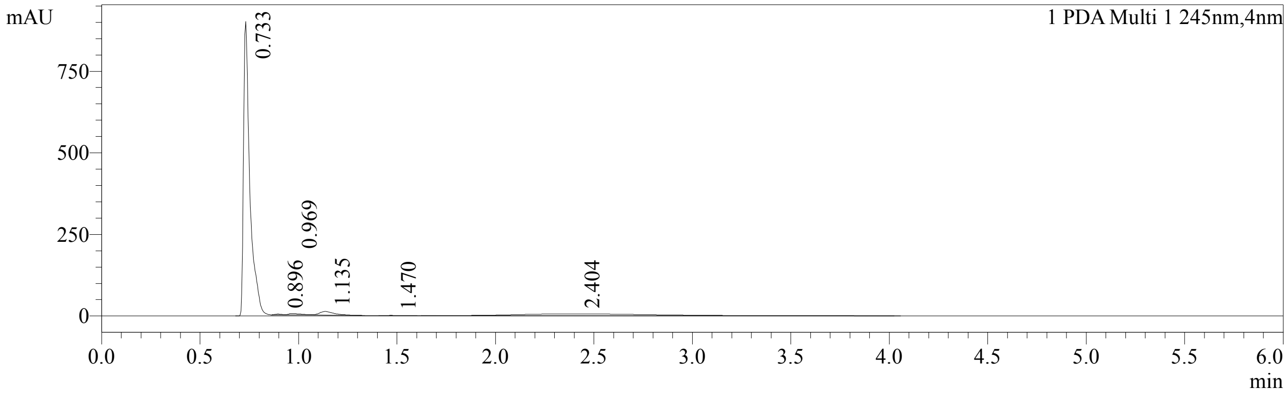

CAUCAMU45D10C-VITC-01.lcd

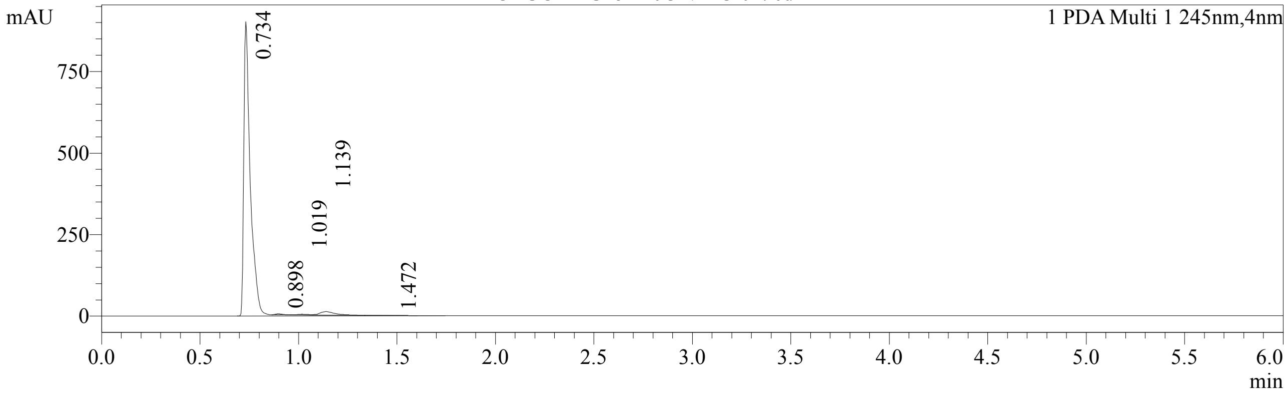

CAUCAMU45D10C-VITC-02.lcd

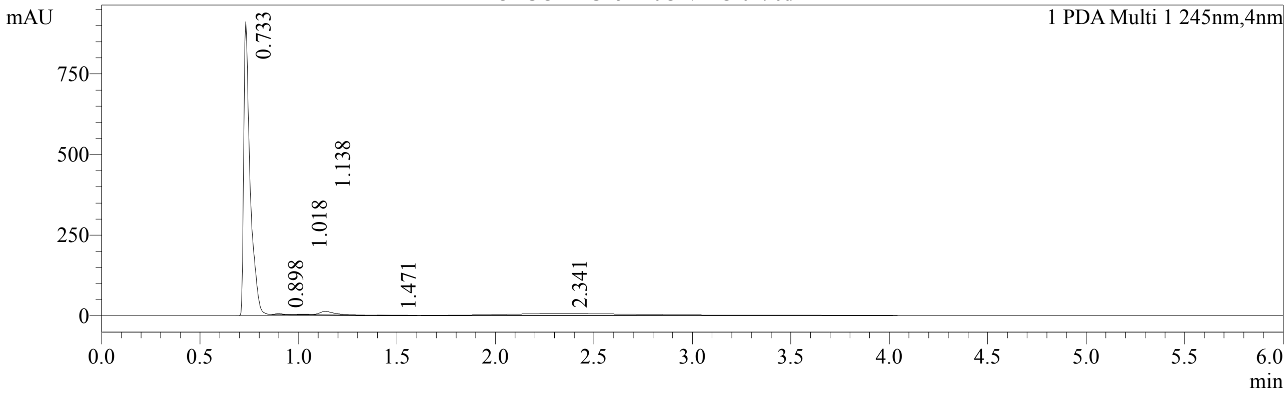

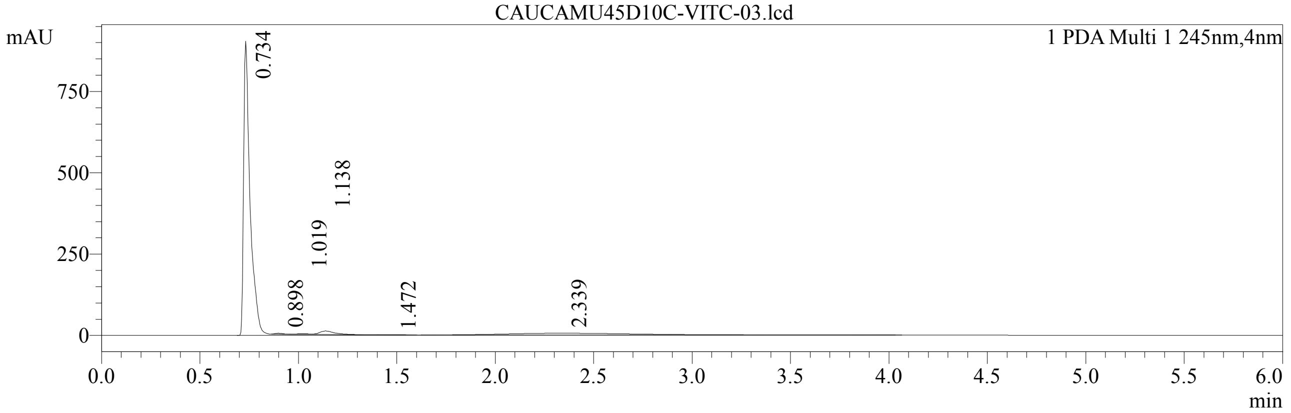
CAUCAMU45D10C-VITC-03.lcd
mAU
1 PDA Multi 1 245nm,4nm
0.734
1.138
1.019
0.898
1.472
2.339
750
500
250
0
0.0
0.5
1.0
1.5
2.0
2.5
3.0
3.5
4.0
4.5
5.0
5.5
6.0
min

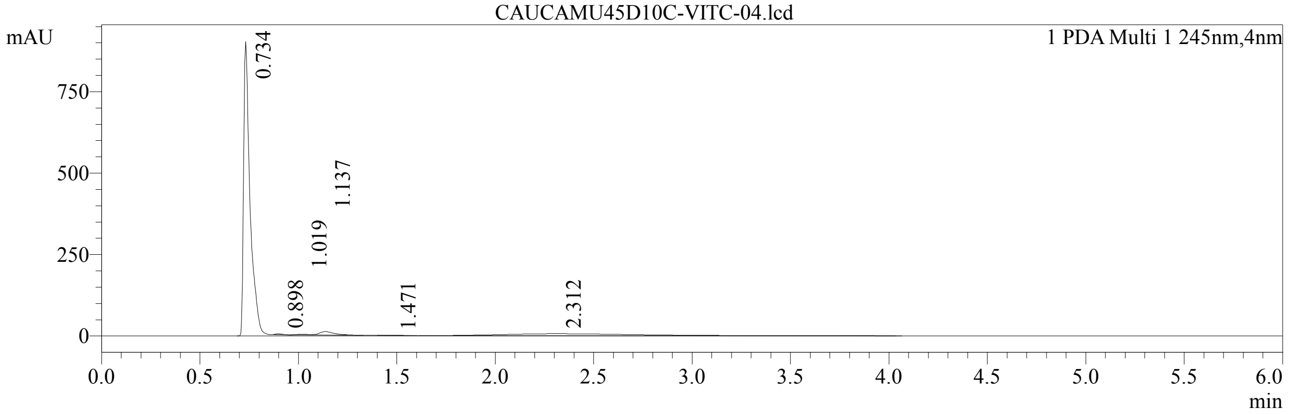
CAUCAMU45D10C-VITC-04.lcd
mAU
1 PDA Multi 1 245nm,4nm
0.734
1.137
1.019
0.898
1.471
2.312
750
500
250
0
0.0
0.5
1.0
1.5
2.0
2.5
3.0
3.5
4.0
4.5
5.0
5.5
6.0
min

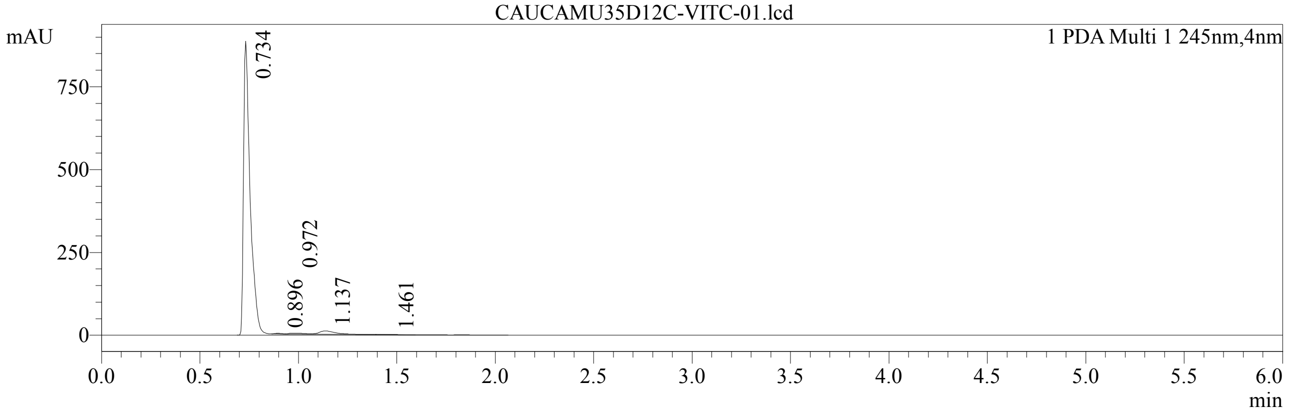
CAUCAMU35D12C-VITC-01.lcd
mAU
1 PDA Multi 1 245nm,4nm
0.734
0.972
0.896
1.137
1.461
750
500
250
0
0.0
0.5
1.0
1.5
2.0
2.5
3.0
3.5
4.0
4.5
5.0
5.5
6.0
min

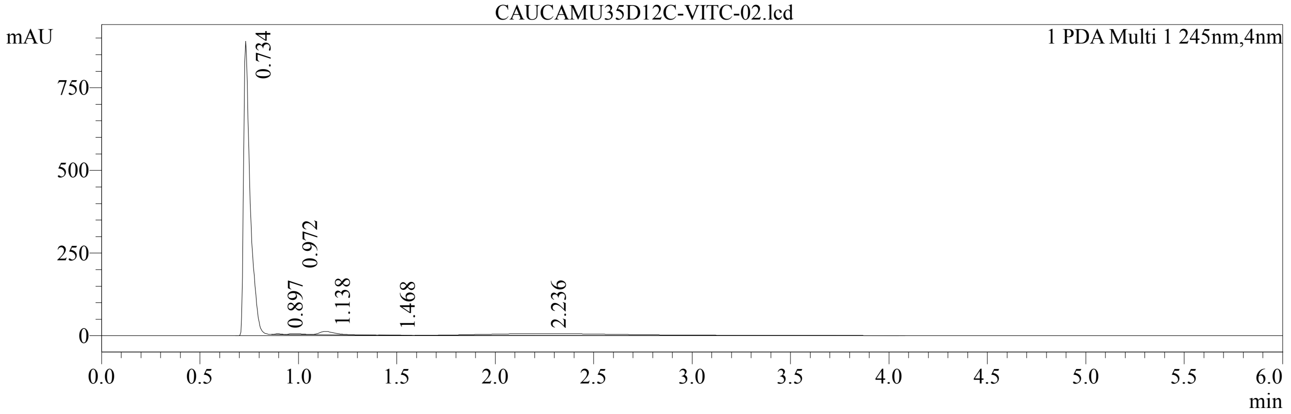
CAUCAMU35D12C-VITC-02.lcd
mAU
1 PDA Multi 1 245nm,4nm
0.734
0.972
0.897
1.138
1.468
2.236
750
500
250
0
0.0
0.5
1.0
1.5
2.0
2.5
3.0
3.5
4.0
4.5
5.0
5.5
6.0
min

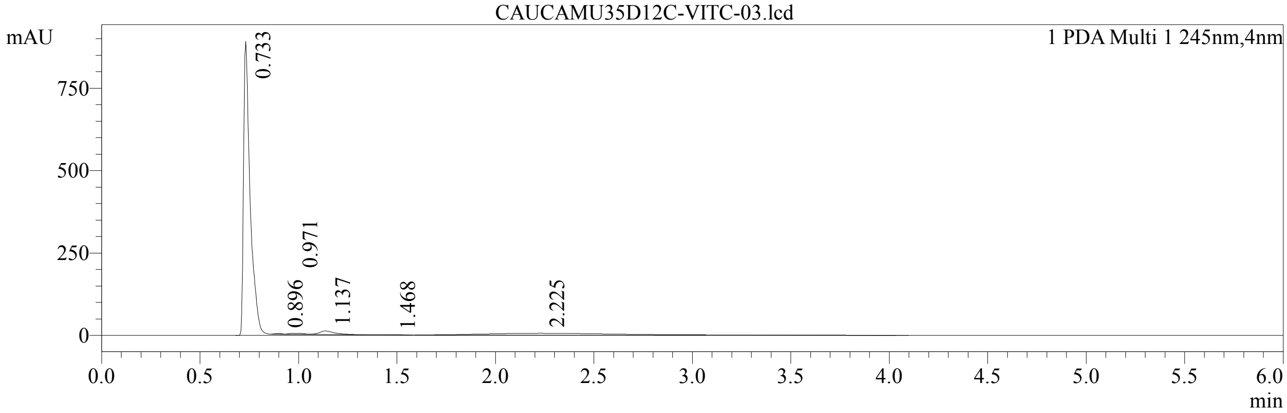
CAUCAMU35D12C-VITC-03.lcd
mAU
1 PDA Multi 1 245nm,4nm
0.733
0.971
0.896
1.137
1.468
2.225
750
500
250
0
0.0
0.5
1.0
1.5
2.0
2.5
3.0
3.5
4.0
4.5
5.0
5.5
6.0
min

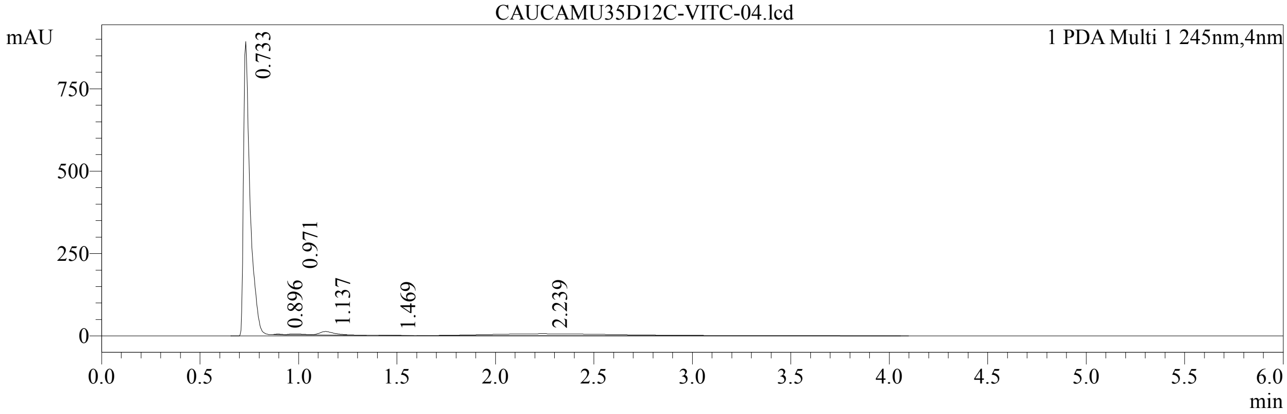
CAUCAMU35D12C-VITC-04.lcd
mAU
1 PDA Multi 1 245nm,4nm
0.733
0.971
0.896
1.137
1.469
2.239
750
500
250
0
0.0
0.5
1.0
1.5
2.0
2.5
3.0
3.5
4.0
4.5
5.0
5.5
6.0
min

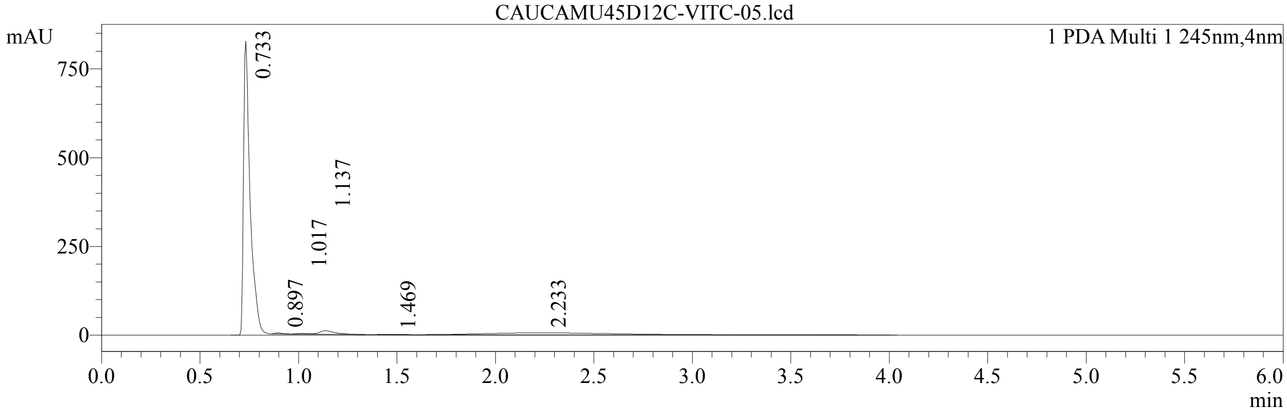
CAUCAMU45D12C-VITC-05.lcd
mAU
1 PDA Multi 1 245nm,4nm
0.733
1.137
1.017
0.897
1.469
2.233
750
500
250
0
0.0
0.5
1.0
1.5
2.0
2.5
3.0
3.5
4.0
4.5
5.0
5.5
6.0
min

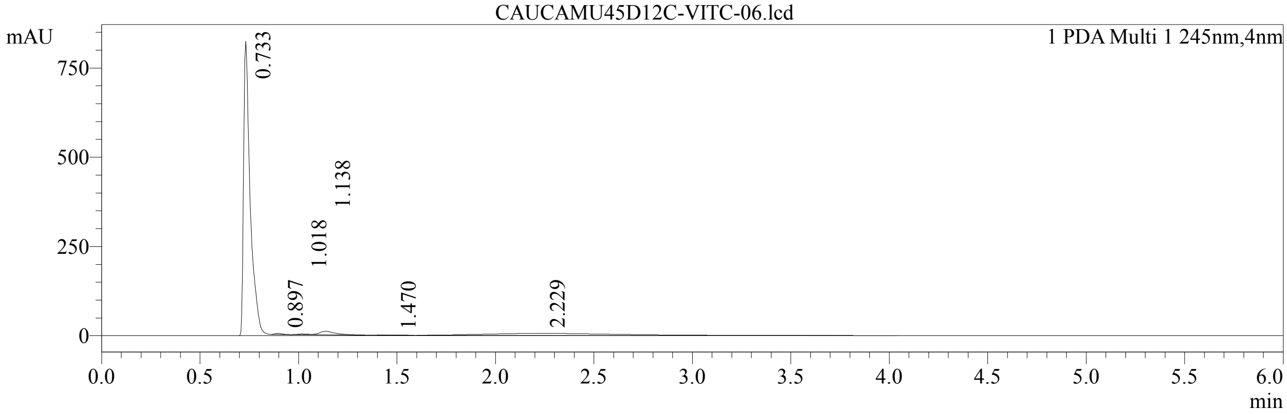
CAUCAMU45D12C-VITC-06.lcd
mAU
1 PDA Multi 1 245nm,4nm
0.733
1.138
1.018
0.897
1.470
2.229
750
500
250
0
0.0
0.5
1.0
1.5
2.0
2.5
3.0
3.5
4.0
4.5
5.0
5.5
6.0
min

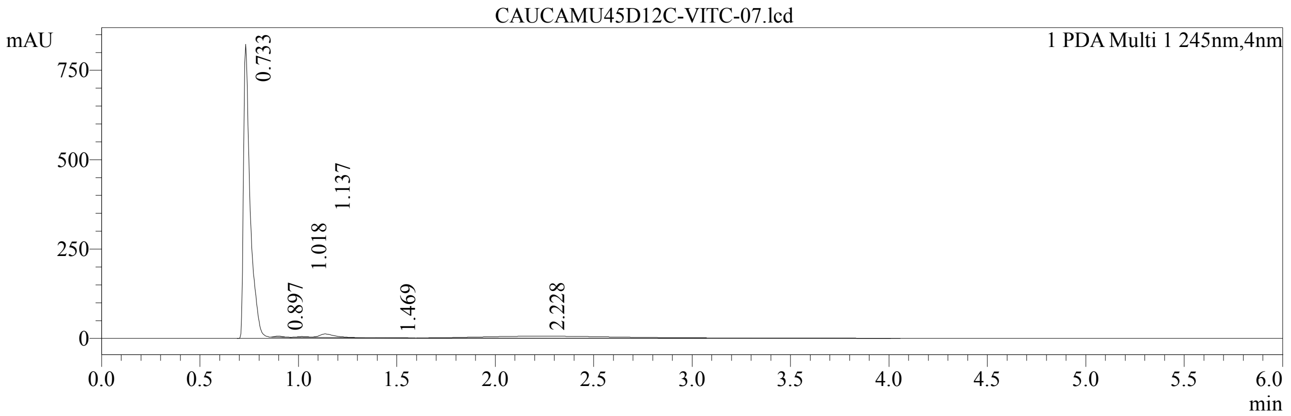
CAUCAMU45D12C-VITC-07.lcd
mAU
1 PDA Multi 1 245nm,4nm
0.733
1.137
1.018
0.897
1.469
2.228
750
500
250
0
0.0
0.5
1.0
1.5
2.0
2.5
3.0
3.5
4.0
4.5
5.0
5.5
6.0
min

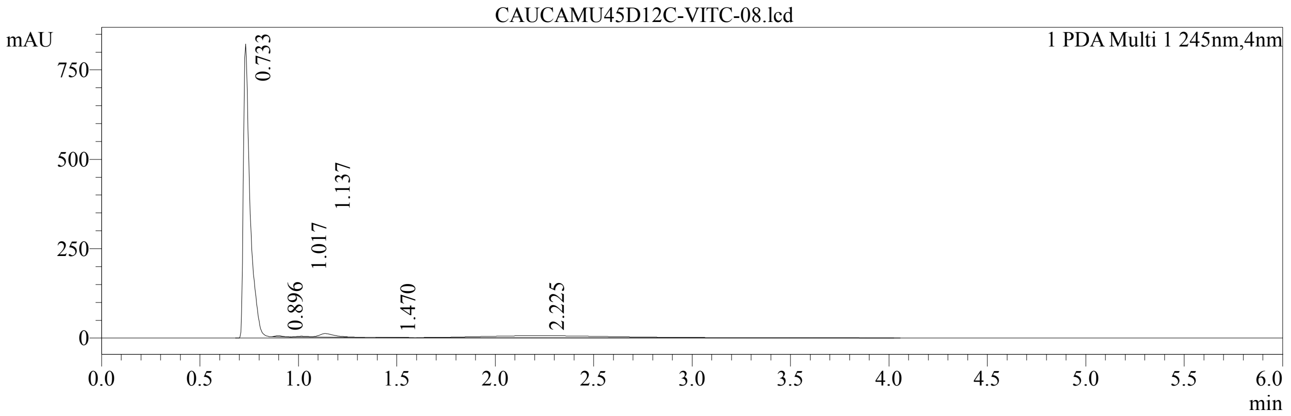
CAUCAMU45D12C-VITC-08.lcd
mAU
1 PDA Multi 1 245nm,4nm
0.733
1.137
1.017
0.896
1.470
2.225
750
500
250
0
0.0
0.5
1.0
1.5
2.0
2.5
3.0
3.5
4.0
4.5
5.0
5.5
6.0
min

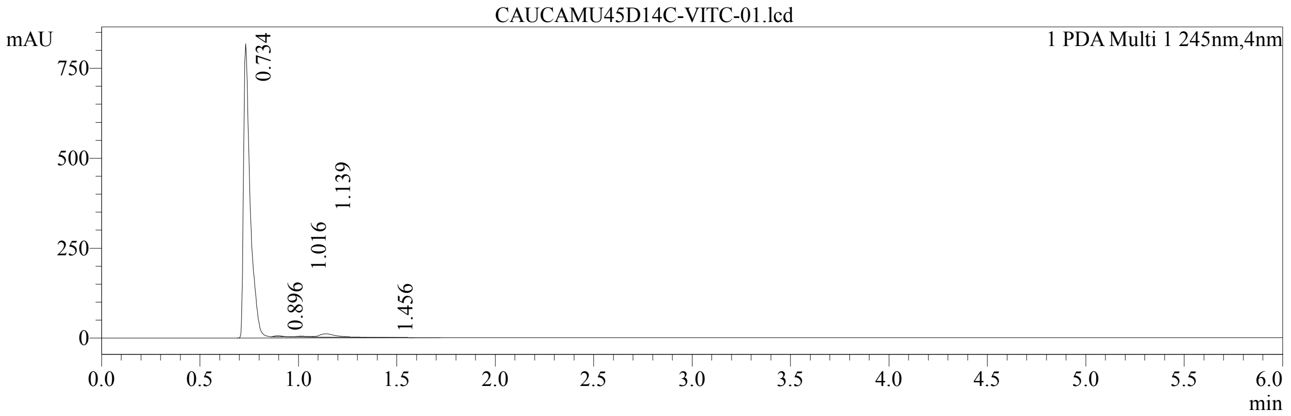
CAUCAMU45D14C-VITC-01.lcd
mAU
1 PDA Multi 1 245nm,4nm
0.734
1.139
1.016
0.896
1.456
750
500
250
0
0.0
0.5
1.0
1.5
2.0
2.5
3.0
3.5
4.0
4.5
5.0
5.5
6.0
min

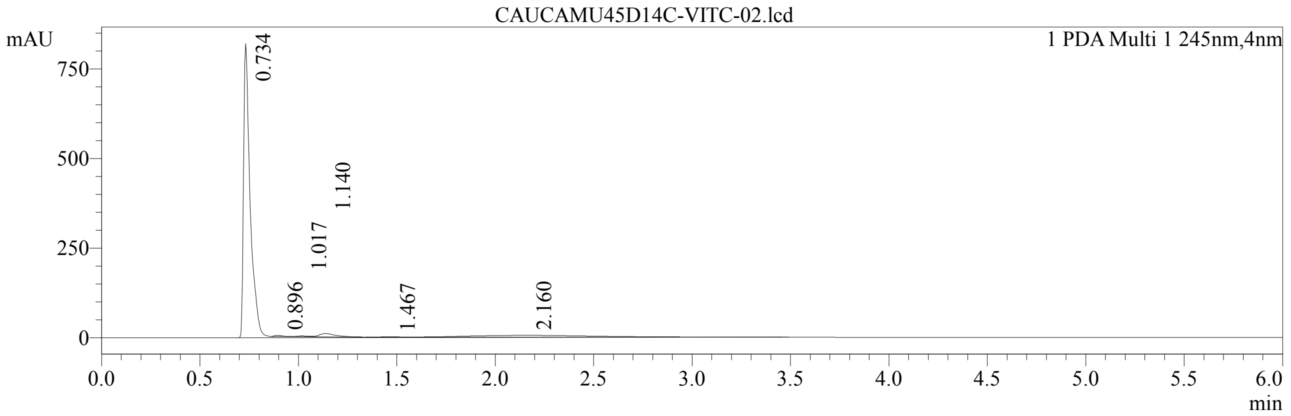
CAUCAMU45D14C-VITC-02.lcd
mAU
1 PDA Multi 1 245nm,4nm
0.734
1.140
1.017
0.896
1.467
2.160
750
500
250
0
0.0
0.5
1.0
1.5
2.0
2.5
3.0
3.5
4.0
4.5
5.0
5.5
6.0
min

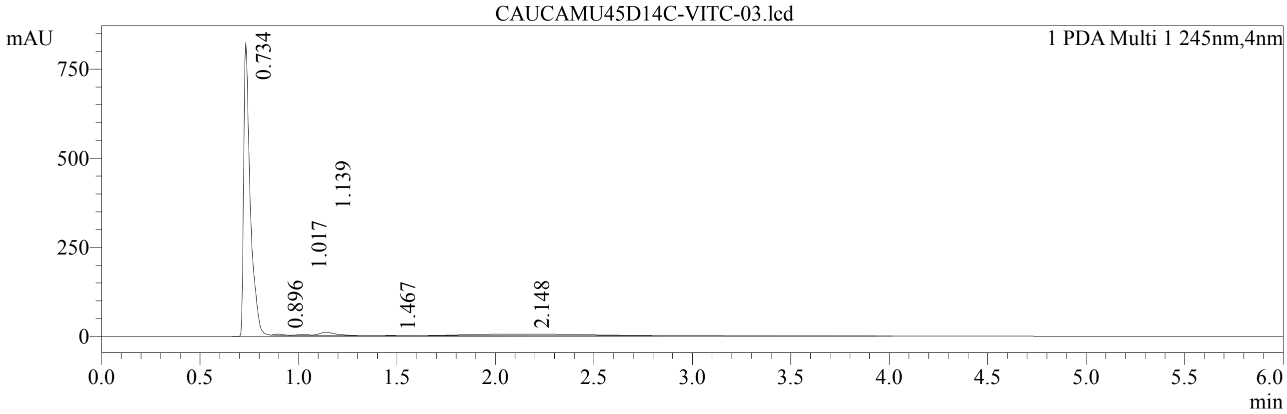
CAUCAMU45D14C-VITC-03.lcd
mAU
1 PDA Multi 1 245nm,4nm
0.734
1.139
1.017
0.896
1.467
2.148
750
500
250
0
0.0 0.5 1.0 1.5 2.0 2.5 3.0 3.5 4.0 4.5 5.0 5.5 6.0
min

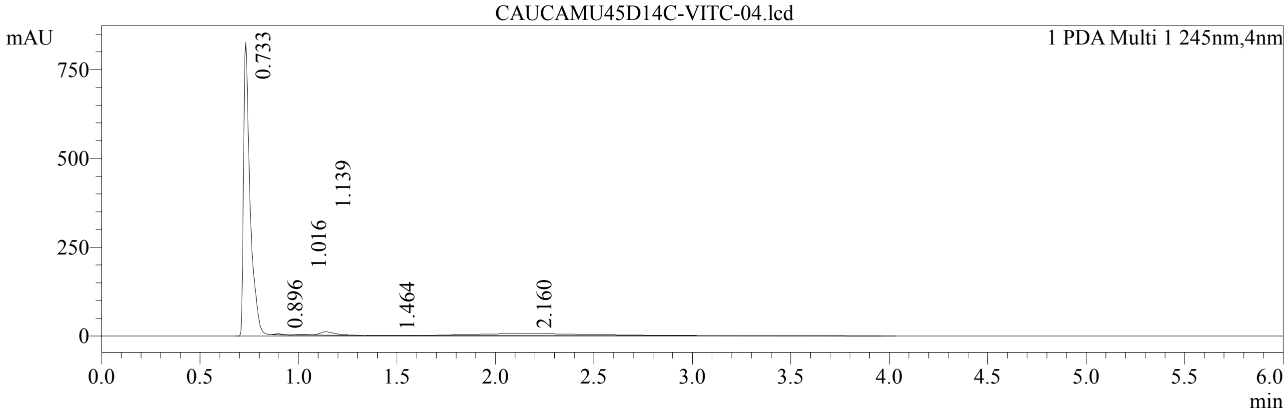
CAUCAMU45D14C-VITC-04.lcd
mAU
1 PDA Multi 1 245nm,4nm
0.733
1.139
1.016
0.896
1.464
2.160
750
500
250
0
0.0 0.5 1.0 1.5 2.0 2.5 3.0 3.5 4.0 4.5 5.0 5.5 6.0
min

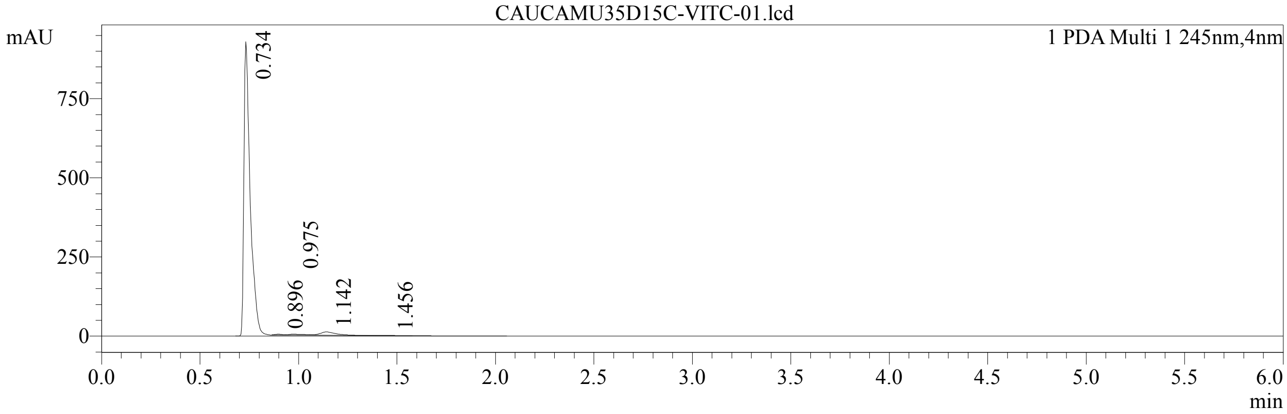
CAUCAMU35D15C-VITC-01.lcd
mAU
1 PDA Multi 1 245nm,4nm
0.734
0.975
0.896
1.142
1.456
750
500
250
0
0.0 0.5 1.0 1.5 2.0 2.5 3.0 3.5 4.0 4.5 5.0 5.5 6.0
min

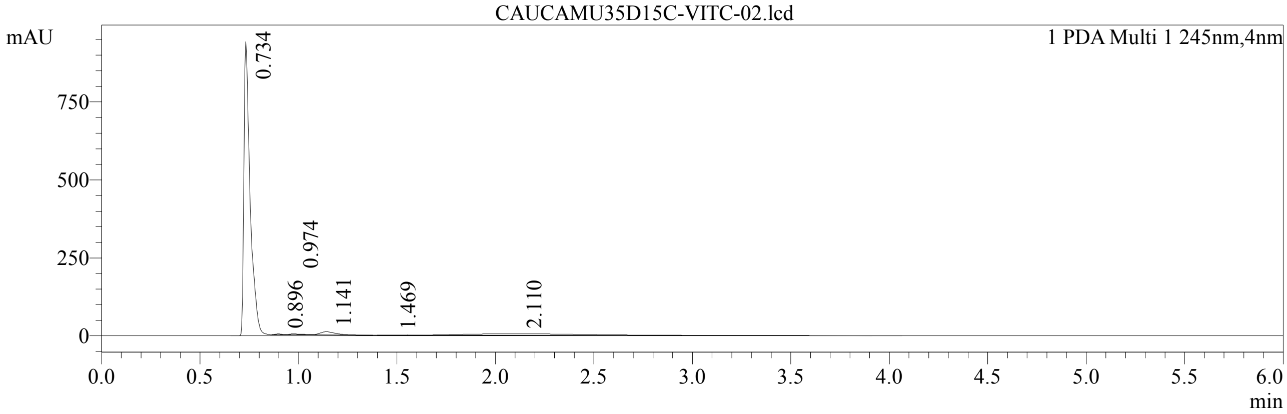
CAUCAMU35D15C-VITC-02.lcd
mAU
1 PDA Multi 1 245nm,4nm
0.734
0.974
0.896
1.141
1.469
2.110
750
500
250
0
0.0 0.5 1.0 1.5 2.0 2.5 3.0 3.5 4.0 4.5 5.0 5.5 6.0
min

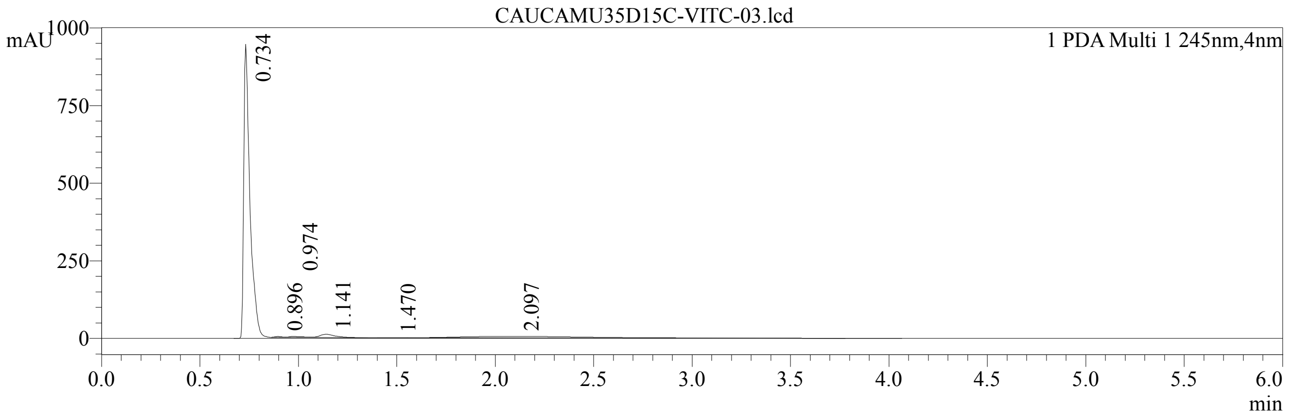
CAUCAMU35D15C-VITC-03.lcd
mAU
1 PDA Multi 1 245nm,4nm
0.734
0.896
0.974
1.141
1.470
2.097
min

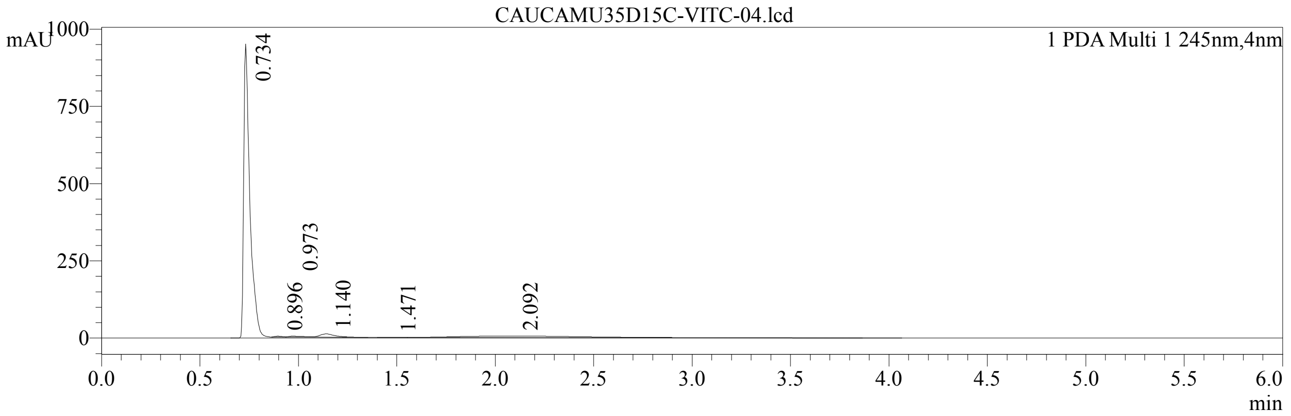
CAUCAMU35D15C-VITC-04.lcd
mAU
1 PDA Multi 1 245nm,4nm
0.734
0.896
0.973
1.140
1.471
2.092
min

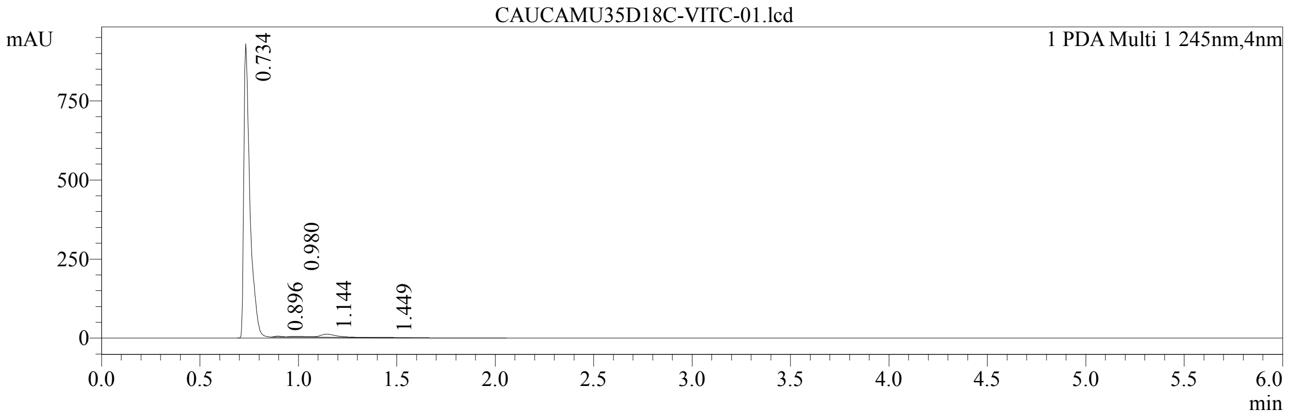
CAUCAMU35D18C-VITC-01.lcd
mAU
1 PDA Multi 1 245nm,4nm
0.734
0.896
0.980
1.144
1.449
min

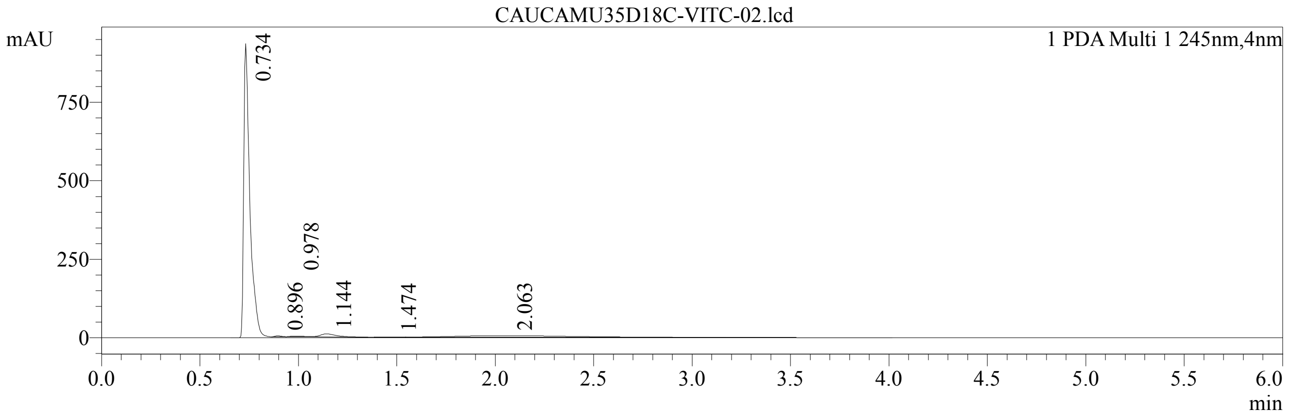
CAUCAMU35D18C-VITC-02.lcd
mAU
1 PDA Multi 1 245nm,4nm
0.734
0.896
0.978
1.144
1.474
2.063
min

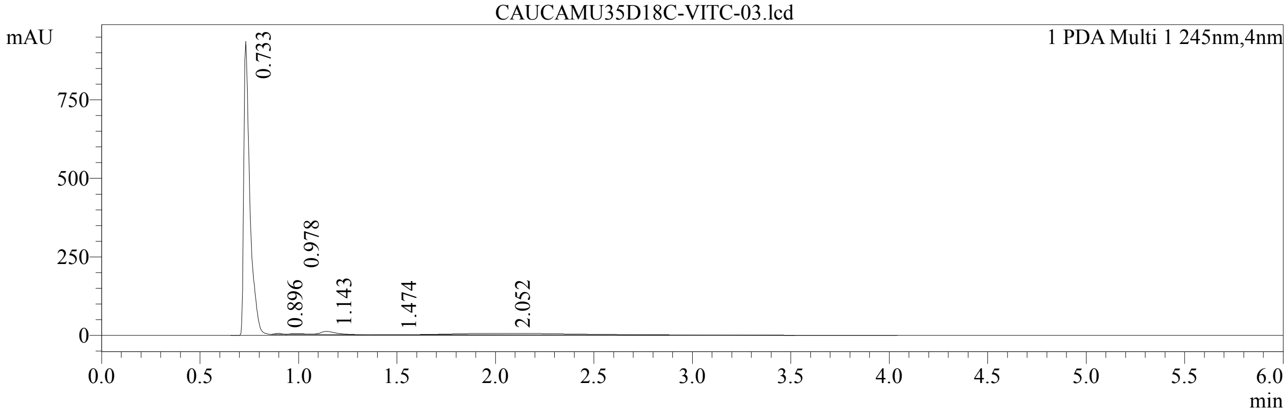
CAUCAMU35D18C-VITC-03.lcd
mAU
1 PDA Multi 1 245nm,4nm
0.733
0.896
0.978
1.143
1.474
2.052
750
500
250
0
0.0
0.5
1.0
1.5
2.0
2.5
3.0
3.5
4.0
4.5
5.0
5.5
6.0
min

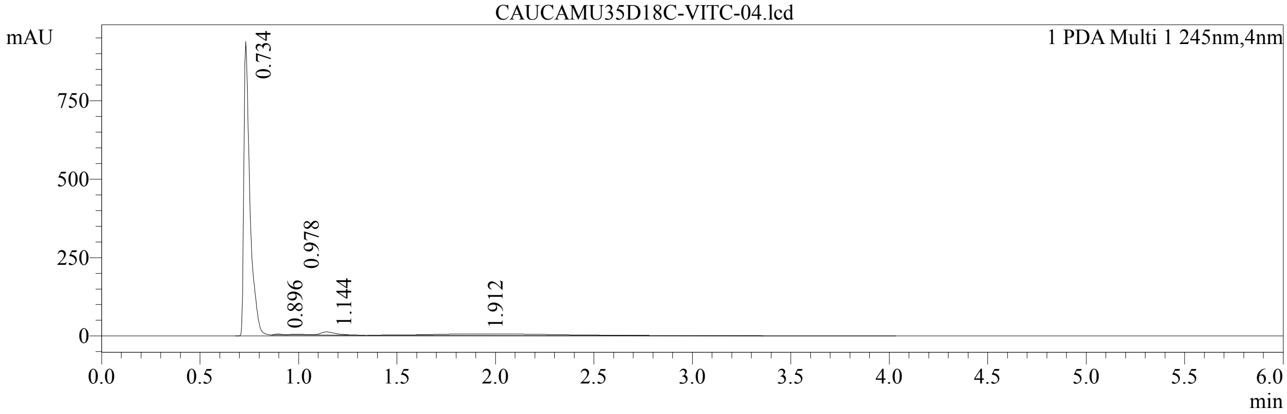
CAUCAMU35D18C-VITC-04.lcd
mAU
1 PDA Multi 1 245nm,4nm
0.734
0.896
0.978
1.144
1.912
750
500
250
0
0.0
0.5
1.0
1.5
2.0
2.5
3.0
3.5
4.0
4.5
5.0
5.5
6.0
min

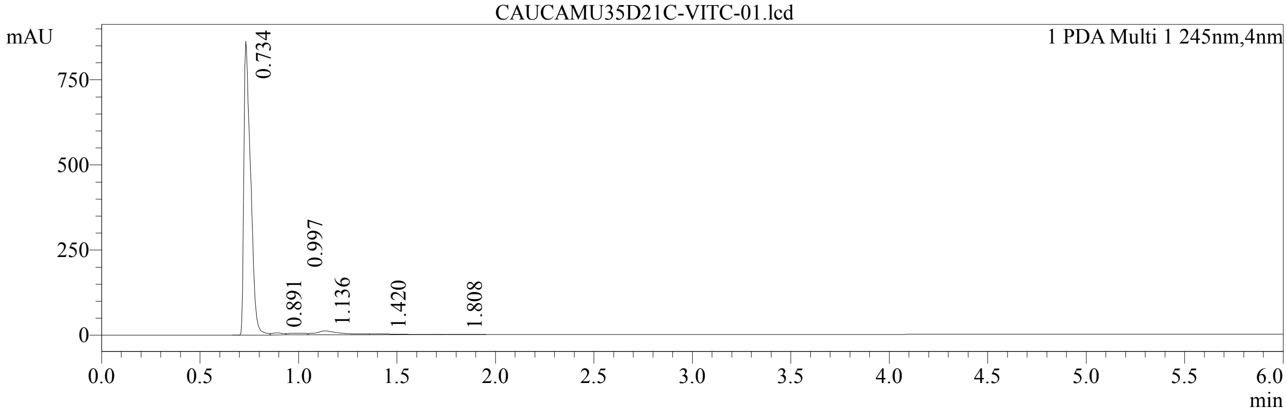
CAUCAMU35D21C-VITC-01.lcd
mAU
1 PDA Multi 1 245nm,4nm
0.734
0.891
0.997
1.136
1.420
1.808
750
500
250
0
0.0
0.5
1.0
1.5
2.0
2.5
3.0
3.5
4.0
4.5
5.0
5.5
6.0
min

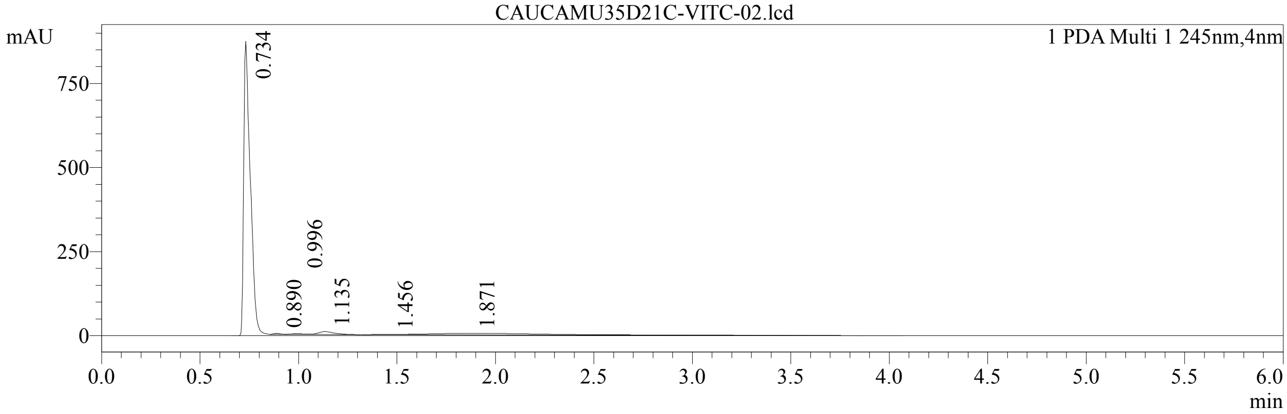
CAUCAMU35D21C-VITC-02.lcd
mAU
1 PDA Multi 1 245nm,4nm
0.734
0.890
0.996
1.135
1.456
1.871
750
500
250
0
0.0
0.5
1.0
1.5
2.0
2.5
3.0
3.5
4.0
4.5
5.0
5.5
6.0
min

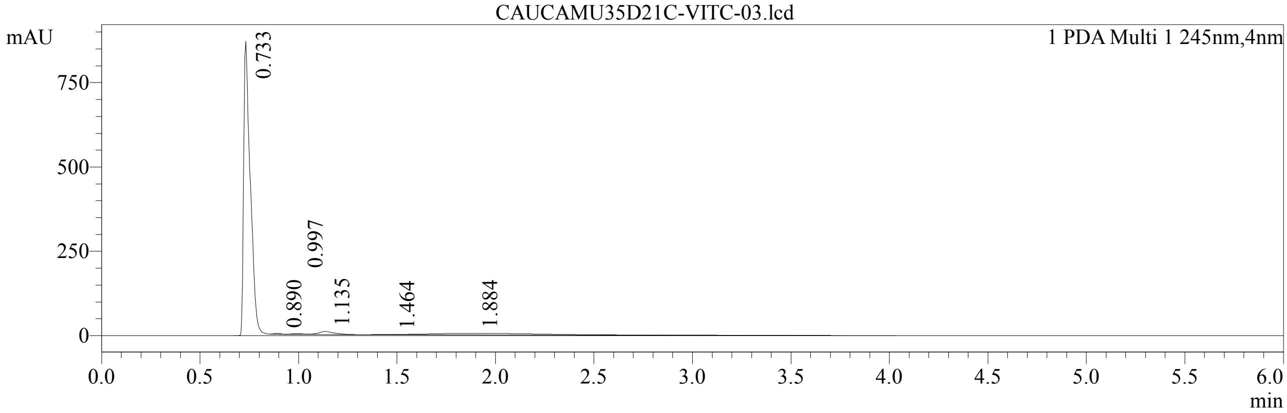

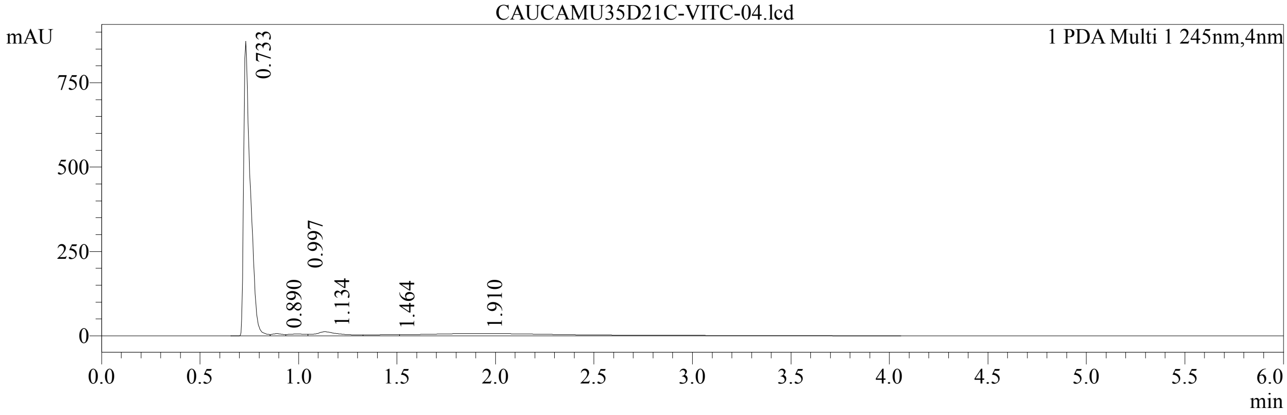

<< PDA >>

ID#1 Compound Name: VIT C

Title	Sample Name	Sample ID	Ret. Time	Area	Height	Conc.
CAMU55-D1-VITC-01.lcd	PROT-VITC-01	CAMU55-D1-VITC-01	0.741	8691605	3822055	3458.379
CAMU55-D1-VITC-02.lcd	PROT-VITC-02	CAMU55-D1-VITC-02	0.736	2739828	1037148	1090.174
CAMU55-D1-VITC-03.lcd	PROT-VITC-03	CAMU55-D1-VITC-03	0.733	2399901	1025360	954.918
CAMU55-D1D-VITC-01.lcd	PROT-VITC-01	CAMU55-D1D-VITC-01	0.729	2601840	1022059	1035.269
CAMU55-D1D-VITC-02.lcd	PROT-VITC-02	CAMU55-D1D-VITC-02	0.734	2459312	1045500	978.557
CAMU55-D1D-VITC-03.lcd	PROT-VITC-03	CAMU55-D1D-VITC-03	0.736	2388783	1033620	950.494
CAMU45D2C-VITC-01.lcd	PROT-VITC-01	CAMU45D2C-VITC-01	0.735	2209050	963900	878.978
CAMU45D2C-VITC-02.lcd	PROT-VITC-02	CAMU45D2C-VITC-02	0.736	2243399	948093	892.646
CAMU45D2C-VITC-03.lcd	PROT-VITC-03	CAMU45D2C-VITC-03	0.735	2209300	948172	879.078
CAMU45D2C-VITC-04.lcd	PROT-VITC-04	CAMU45D2C-VITC-04	0.736	2492205	952089	991.645
CAMU55D2C-VITC-05.lcd	PROT-VITC-05	CAMU55D2C-VITC-05	0.733	2357513	960764	938.051
CAMU55D2C-VITC-06.lcd	PROT-VITC-06	CAMU55D2C-VITC-06	0.735	2392741	969831	952.069
CAMU55D2C-VITC-07.lcd	PROT-VITC-07	CAMU55D2C-VITC-07	0.734	2339566	962690	930.910
CAMU55D2C-VITC-08.lcd	PROT-VITC-08	CAMU55D2C-VITC-08	0.737	2881653	955985	1146.606
CAMU35D3C-VITC-01.lcd	PROT-VITC-01	CAMU35D3C-VITC-01	0.735	2317266	928595	922.037
CAMU35D3C-VITC-02.lcd	PROT-VITC-02	CAMU35D3C-VITC-02	0.734	2332918	933143	928.265
CAMU35D3C-VITC-03.lcd	PROT-VITC-03	CAMU35D3C-VITC-03	0.735	2299396	922493	914.927
CAMU35D3C-VITC-04.lcd	PROT-VITC-04	CAMU35D3C-VITC-04	0.735	2304678	925415	917.028
CAMU55D3C-VITC-05.lcd	PROT-VITC-05	CAMU55D3C-VITC-05	0.734	1915622	771697	762.224
CAMU55D3C-VITC-06.lcd	PROT-VITC-06	CAMU55D3C-VITC-06	0.735	1963114	768751	781.120
CAMU55D3C-VITC-07.lcd	PROT-VITC-07	CAMU55D3C-VITC-07	0.734	1993550	765852	793.231
CAMU55D3C-VITC-08.lcd	PROT-VITC-08	CAMU55D3C-VITC-08	0.734	2006075	769460	798.215
CAMU45D4C-VITC-01.lcd	PROT-VITC-01	CAMU45D4C-VITC-01	0.735	2174111	890231	865.076
CAMU45D4C-VITC-02.lcd	PROT-VITC-02	CAMU45D4C-VITC-02	0.735	2203513	893517	876.775
CAMU45D4C-VITC-03.lcd	PROT-VITC-03	CAMU45D4C-VITC-03	0.734	2263471	889986	900.632
CAMU45D4C-VITC-04.lcd	PROT-VITC-04	CAMU45D4C-VITC-04	0.735	2213827	891776	880.879

Title	Sample Name	Sample ID	Ret. Time	Area	Height	Conc.
CAMU55D4C-VITC-05.lcd	PROT-VITC-05	CAMU55D4C-VITC-05	0.734	2226648	904813	885.981
CAMU55D4C-VITC-06.lcd	PROT-VITC-06	CAMU55D4C-VITC-06	0.734	2287431	907380	910.166
CAMU55D4C-VITC-07.lcd	PROT-VITC-07	CAMU55D4C-VITC-07	0.734	2230143	911545	887.371
CAMU55D4C-VITC-08.lcd	PROT-VITC-08	CAMU55D4C-VITC-08	0.733	2272246	901032	904.124
CAMU55D5C-VITC-01.lcd	PROT-VITC-01	CAMU55D5C-VITC-01	0.733	2186316	867102	869.932
CAMU55D5C-VITC-02.lcd	PROT-VITC-02	CAMU55D5C-VITC-02	0.733	2229429	870326	887.087
CAMU55D5C-VITC-03.lcd	PROT-VITC-03	CAMU55D5C-VITC-03	0.733	2230205	876961	887.396
CAMU55D5C-VITC-04.lcd	PROT-VITC-04	CAMU55D5C-VITC-04	0.732	2228304	870139	886.639
CAMU45D6C-VITC-01.lcd	PROT-VITC-01	CAMU45D6C-VITC-01	0.734	2154916	888105	857.438
CAMU45D6C-VITC-02.lcd	PROT-VITC-02	CAMU45D6C-VITC-02	0.733	2199235	877201	875.073
CAMU45D6C-VITC-03.lcd	PROT-VITC-03	CAMU45D6C-VITC-03	0.733	2192078	868812	872.225
CAMU45D6C-VITC-04.lcd	PROT-VITC-04	CAMU45D6C-VITC-04	0.734	2191958	870909	872.177
CAMU55D6C-VITC-05.lcd	PROT-VITC-05	CAMU55D6C-VITC-05	0.733	2000658	790986	796.059
CAMU55D6C-VITC-06.lcd	PROT-VITC-06	CAMU55D6C-VITC-06	0.733	1989000	776902	791.421
CAMU55D6C-VITC-07.lcd	PROT-VITC-07	CAMU55D6C-VITC-07	0.733	1998354	778860	795.143
CAMU55D6C-VITC-08.lcd	PROT-VITC-08	CAMU55D6C-VITC-08	0.733	1995024	782563	793.817
CAUCAMU35D6C-VITC-01.lcd	PROT-VITC-01	CAUCAMU35D6C-VITC-01	0.735	2185773	904341	869.716
CAUCAMU35D6C-VITC-02.lcd	PROT-VITC-02	CAUCAMU35D6C-VITC-0	0.734	2248610	904849	894.719
CAUCAMU35D6C-VITC-03.lcd	PROT-VITC-03	CAUCAMU35D6C-VITC-03	0.735	2250572	910374	895.500
CAUCAMU35D6C-VITC-04.lcd	PROT-VITC-04	CAUCAMU35D6C-VITC-04	0.733	2246508	900672	893.883
CAUCAMU55D7C-VITC-05.lcd	PROT-VITC-05	CAUCAMU55D7C-VITC-05	0.733	2121540	831387	844.158
CAUCAMU55D7C-VITC-06.lcd	PROT-VITC-06	CAUCAMU55D7C-VITC-06	0.733	2110863	838995	839.910
CAUCAMU55D7C-VITC-07.lcd	PROT-VITC-07	CAUCAMU55D7C-VITC-07	0.733	2119465	832912	843.332
CAUCAMU55D7C-VITC-08.lcd	PROT-VITC-08	CAUCAMU55D7C-VITC-08	0.733	2114330	827544	841.289
CAUCAMU45D8C-VITC-01.lcd	PROT-VITC-01	CAUCAMU145D6C-VITC-01	0.734	2127773	876124	846.638
CAUCAMU45D8C-VITC-02.lcd	PROT-VITC-02	CAUCAMU45D6C-VITC-02	0.734	2192139	880791	872.249
CAUCAMU45D8C-VITC-03.lcd	PROT-VITC-03	CAUCAMU45D6C-VITC-03	0.733	2206687	871940	878.038
CAUCAMU45D8C-VITC-04.lcd	PROT-VITC-04	CAUCAMU45D6C-VITC-04	0.733	2197643	874627	874.439
CAUCAMU35D9C-VITC-01.lcd	PROT-VITC-01	CAUCAMU135D9C-VITC-01	0.733	2097015	866478	834.400
CAUCAMU35D9C-VITC-02.lcd	PROT-VITC-02	CAUCAMU35D9C-VITC-02	0.734	2185168	890238	869.476
CAUCAMU35D9C-VITC-03.lcd	PROT-VITC-03	CAUCAMU35D9C-VITC-03	0.734	2202706	891164	876.454
CAUCAMU35D9C-VITC-04.lcd	PROT-VITC-04	CAUCAMU35D9C-VITC-04	0.733	2196156	874852	873.848
CAUCAMU45D10C-VITC-01.lcd	PROT-VITC-01	CAUCAMU45D10C-VITC-01	0.734	2164633	889007	861.305
CAUCAMU45D10C-VITC-02.lcd	PROT-VITC-02	CAUCAMU45D10C-VITC-02	0.733	2262184	891050	900.120
CAUCAMU45D10C-VITC-03.lcd	PROT-VITC-03	CAUCAMU45D10C-VITC-03	0.734	2264295	886755	900.960
CAUCAMU45D10C-VITC-04.lcd	PROT-VITC-04	CAUCAMU45D10C-VITC-04	0.734	2268002	885590	902.435
CAUCAMU35D12C-VITC-01.lcd	PROT-VITC-01	CAUCAMU35D12C-VITC-01	0.734	2189537	870540	871.214
CAUCAMU35D12C-VITC-02.lcd	PROT-VITC-02	CAUCAMU35D12C-VITC-02	0.734	2286733	873421	909.888
CAUCAMU35D12C-VITC-03.lcd	PROT-VITC-03	CAUCAMU35D12C-VITC-03	0.733	2297252	870003	914.074
CAUCAMU35D12C-VITC-04.lcd	PROT-VITC-04	CAUCAMU35D12C-VITC-04	0.733	2292062	869419	912.009
CAUCAMU45D12C-VITC-05.lcd	PROT-VITC-05	CAUCAMU45D12C-VITC-05	0.733	2158693	808865	858.941
CAUCAMU45D12C-VITC-06.lcd	PROT-VITC-06	CAUCAMU45D12C-VITC-06	0.733	2153013	807710	856.681
CAUCAMU45D12C-VITC-07.lcd	PROT-VITC-07	CAUCAMU45D12C-VITC-07	0.733	2146836	806282	854.223
CAUCAMU45D12C-VITC-08.lcd	PROT-VITC-08	CAUCAMU45D12C-VITC-08	0.733	2155514	802202	857.676
CAUCAMU45D14C-VITC-01.lcd	PROT-VITC-01	CAUCAMU45D14C-VITC-01	0.734	2013801	802504	801.289
CAUCAMU45D14C-VITC-02.lcd	PROT-VITC-02	CAUCAMU45D14C-VITC-02	0.734	2121638	805181	844.197
CAUCAMU45D14C-VITC-03.lcd	PROT-VITC-03	CAUCAMU45D14C-VITC-03	0.734	2176463	809551	866.012
CAUCAMU45D14C-VITC-04.lcd	PROT-VITC-04	CAUCAMU45D14C-VITC-04	0.733	2166600	805550	862.087
CAUCAMU35D15C-VITC-01.lcd	PROT-VITC-01	CAUCAMU35D15C-VITC-01	0.734	2187270	916230	870.312
CAUCAMU35D15C-VITC-02.lcd	PROT-VITC-02	CAUCAMU35D15C-VITC-02	0.734	2349066	925500	934.690
CAUCAMU35D15C-VITC-03.lcd	PROT-VITC-03	CAUCAMU35D15C-VITC-03	0.734	2346345	926162	933.608
CAUCAMU35D15C-VITC-04.lcd	PROT-VITC-04	CAUCAMU35D15C-VITC-04	0.734	2347715	927998	934.153
CAUCAMU35D18C-VITC-01.lcd	PROT-VITC-01	CAUCAMU35D18C-VITC-01	0.734	2141188	913029	851.976
CAUCAMU35D18C-VITC-02.lcd	PROT-VITC-02	CAUCAMU35D18C-VITC-02	0.734	2303317	914358	916.487
CAUCAMU35D18C-VITC-03.lcd	PROT-VITC-03	CAUCAMU35D18C-VITC-03	0.733	2300593	910039	915.403
CAUCAMU35D18C-VITC-04.lcd	PROT-VITC-04	CAUCAMU35D18C-VITC-04	0.734	2321049	915513	923.543
CAUCAMU35D21C-VITC-01.lcd	PROT-VITC-01	CAUCAMU35D21C-VITC-01	0.734	2049234	850877	815.388
CAUCAMU35D21C-VITC-02.lcd	PROT-VITC-02	CAUCAMU35D21C-VITC-02	0.734	2394031	854775	952.582
CAUCAMU35D21C-VITC-03.lcd	PROT-VITC-03	CAUCAMU35D21C-VITC-03	0.733	2373293	849223	944.330
CAUCAMU35D21C-VITC-04.lcd	PROT-VITC-04	CAUCAMU35D21C-VITC-04	0.733	2044242	850293	813.401
Average			0.734	2303299	918102	916.480
%RSD			0.179	30.978	35.184	30.978
Maximum			0.741	8691605	3822055	3458.379
Minimum			0.729	1915622	765852	762.224
Standard Deviation			0.001	713520	323026	283.909

ID# : 1
Name : VIT C
Quantitative Method : External Standard
Function : f(x)=2513.20*x+0
Rr1=0.9983722 Rr2=0.9967470 RSS=1.779444e+010
MeanRF: 2.712798e+003 RFSD: 4.273329e+002 RFRSD: 15.752473
FitType : Linear
ZeroThrough : Through
Weighted Regression : None
Detector Name : PDA

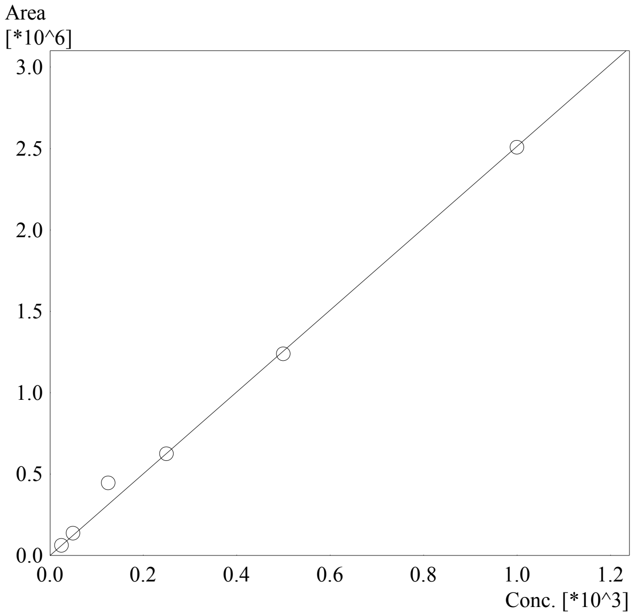

#	Conc.(Ratio)	MeanArea	Area
1	25	62703	62703
2	50	136316	136316
3	125	445542	445542
4	250	623948	623948
5	500	1237707	1237707
6	1000	2506784	2506784

Summary(Conc.)

STD-VITC-01.lcd

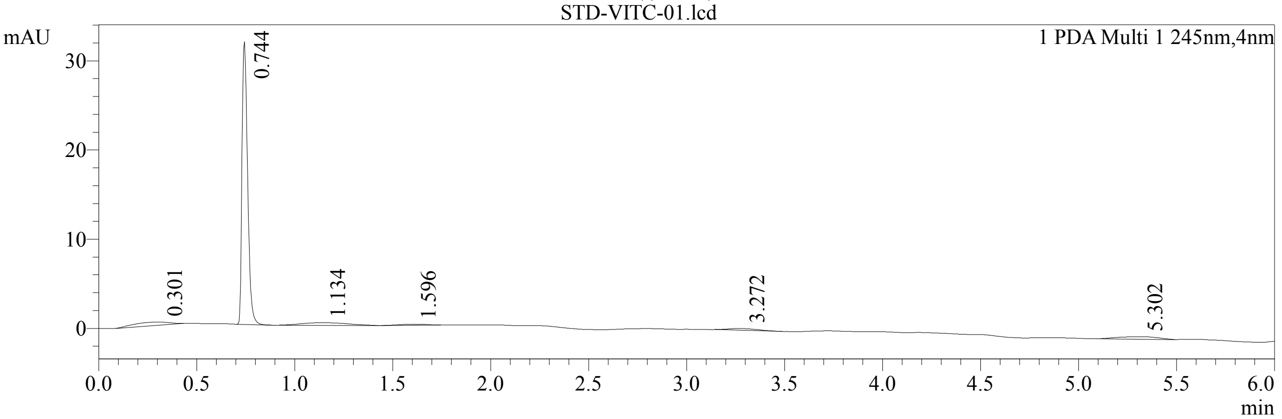

STD-VITC-02.lcd

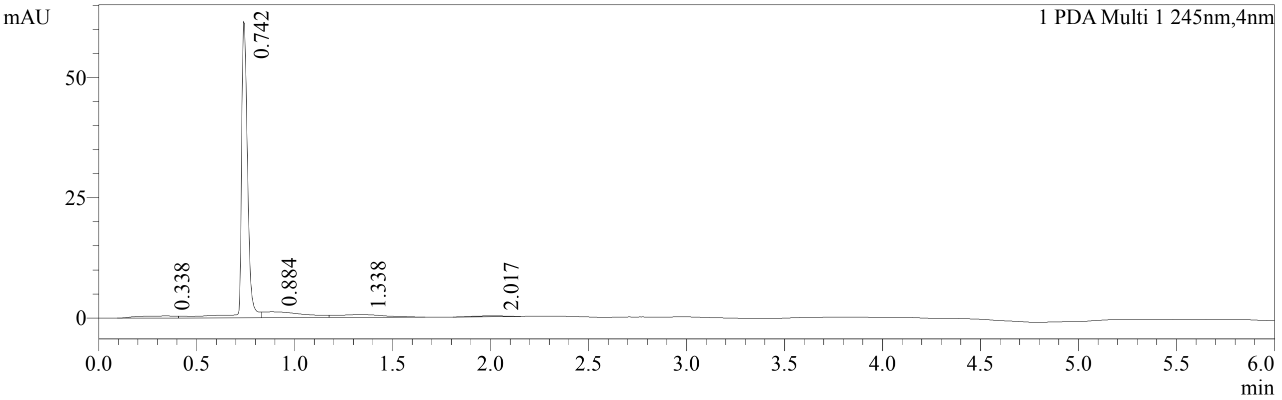

STD-VITC-03.lcd

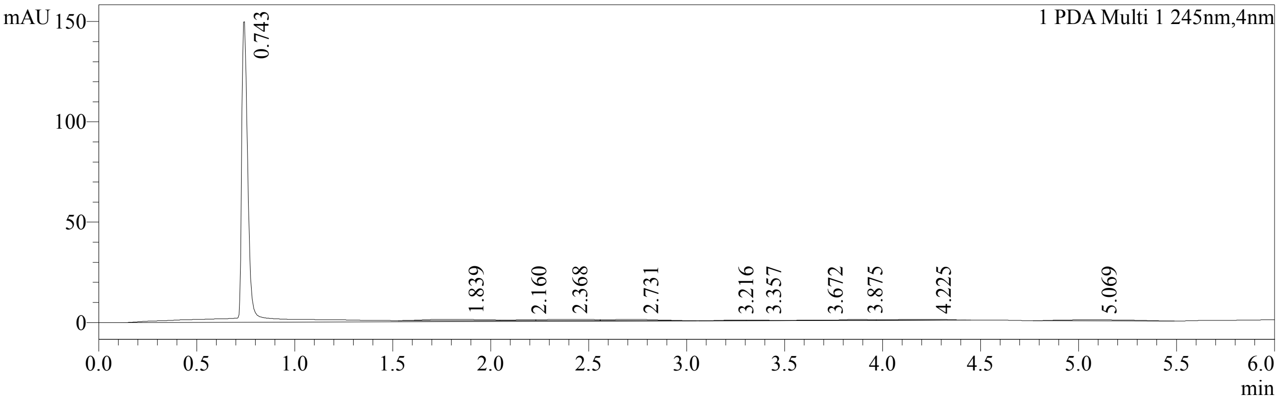

STD-VITC-04.lcd

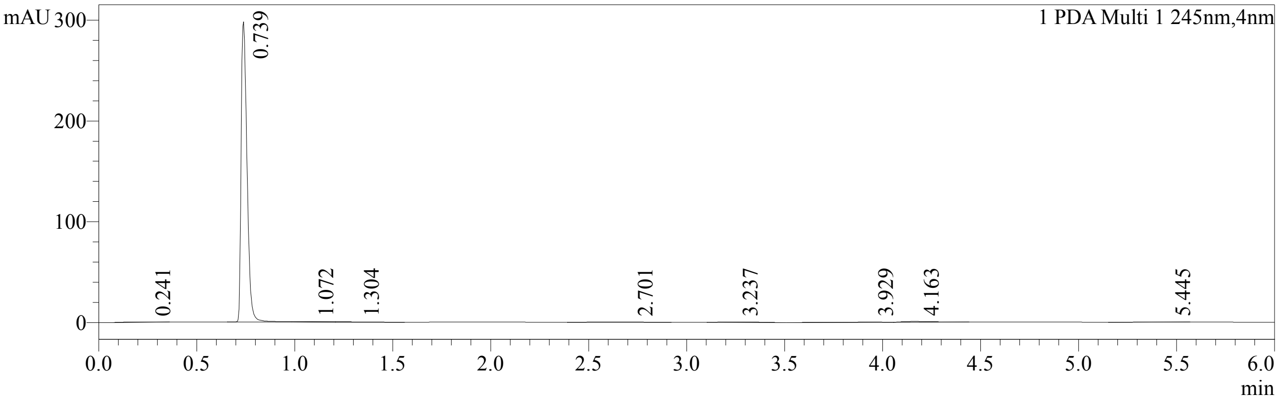

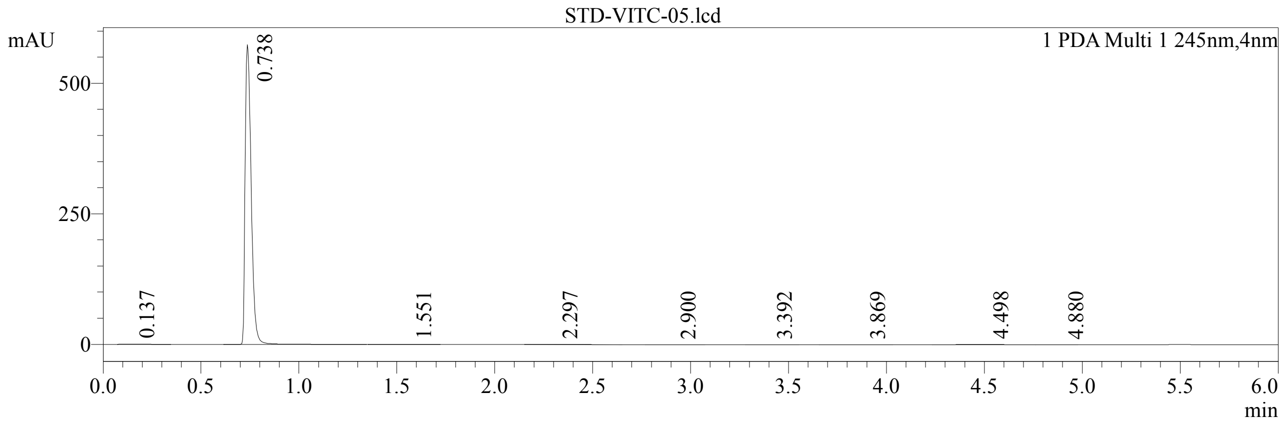

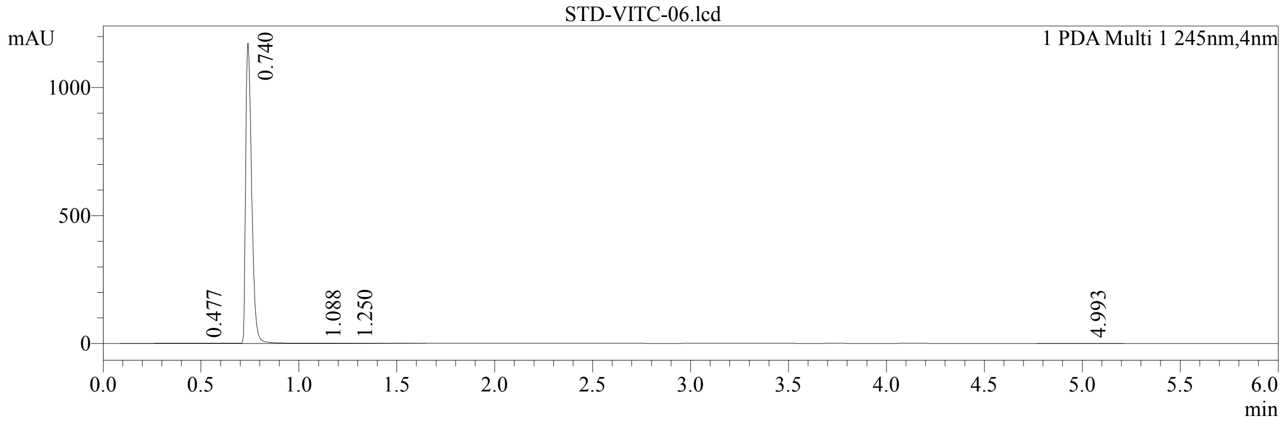

<< PDA >>

Title	Sample Name	Sample ID	VIT C
STD-VITC-01.lcd	STD-VITC-01	STD-VITC-01	24.950
STD-VITC-02.lcd	STD-VITC-02	STD-VITC-02	54.240
STD-VITC-03.lcd	STD-VITC-03	STD-VITC-03	177.281
STD-VITC-04.lcd	STD-VITC-04	STD-VITC-04	248.268
STD-VITC-05.lcd	STD-VITC-05	STD-VITC-05	492.482
STD-VITC-06.lcd	STD-VITC-06	STD-VITC-06	997.446
Average			332.444
%RSD			110.190
Maximum			997.446
Minimum			24.950
Standard Deviation			366.320
