# Supplementary material for: Kinetic Modeling of Vitamin C Degradation for Predicting Shelf Life in Tropical Juices Made from Camu Camu and Naranjilla Under Accelerated Storage Conditions
Source: Foods. 2026 May 14;15(10):1722. doi: 10.3390/foods15101722 (PMC13205238; doi:10.3390/foods15101722)
Supplement: Supplementary file 1 [file foods-15-01722-s001.zip › File S2. Report HPLC chromatogram of obtained from Naranjilla juice extract and ascorbic acid standard solution used for compound identification and retention time determination.PDF]

Shimadzu LabSolutions Report
PEDRO RUIZ GALLO NATIONAL UNIVERSITY-FIQIA
VITAMIN C CHROMATOGRAM FOR NARANJILLA JUICE

Summary(Compound)

NARAN55-D1D-VITC-07.lcd

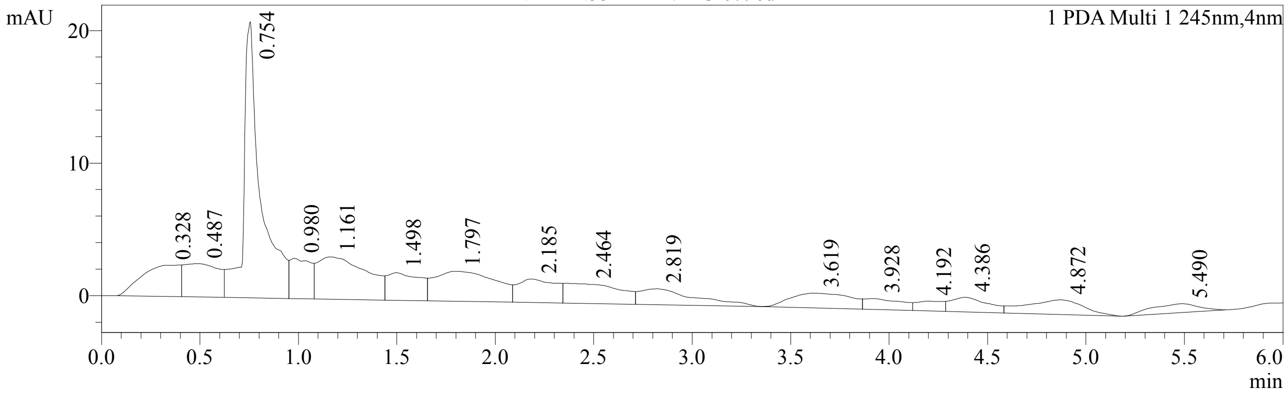

NARAN55-D1D-VITC-08.lcd

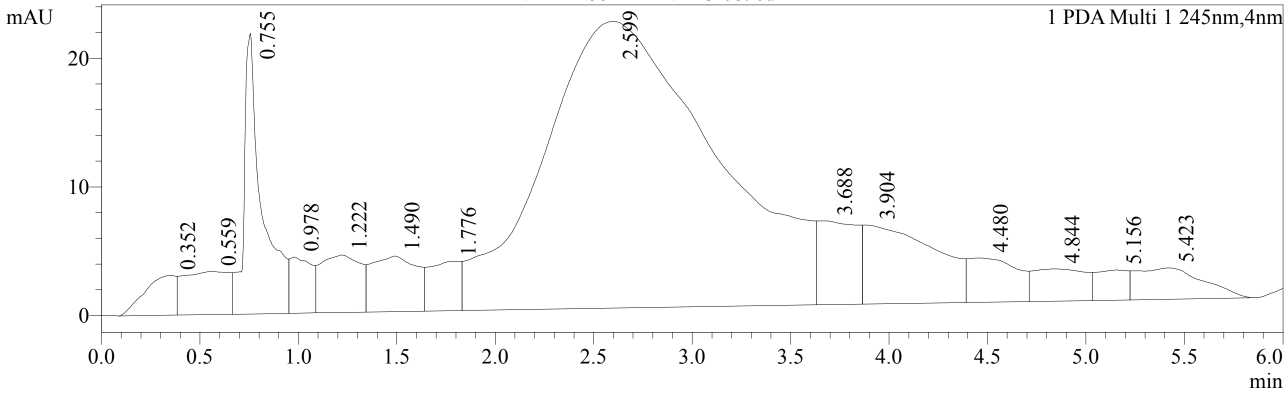

NARAN55-D1D-VITC-09.lcd

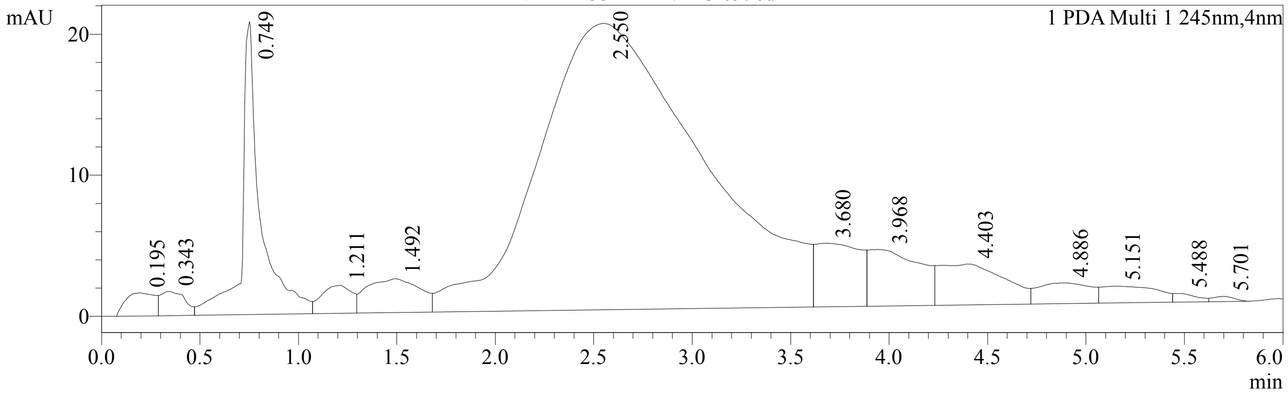

NARAN45D2C-VITC-01.lcd

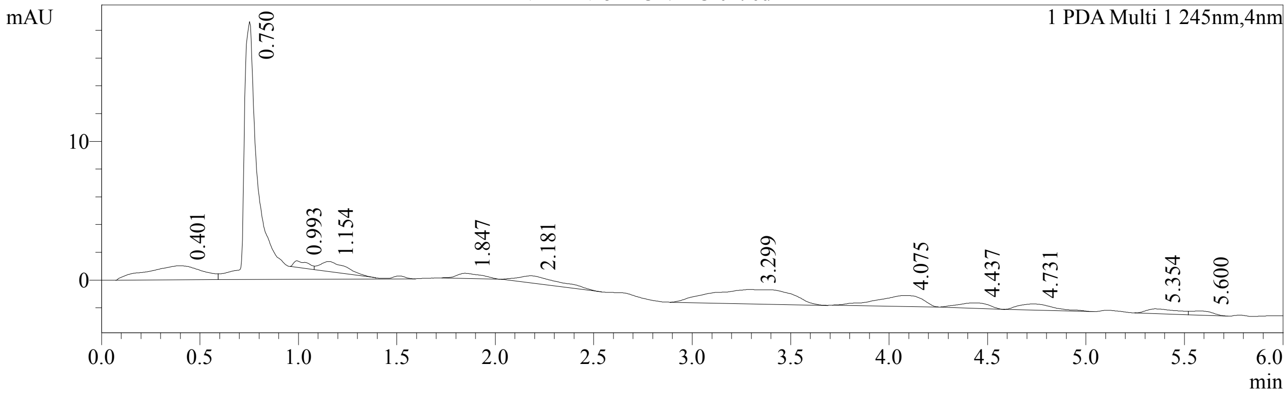

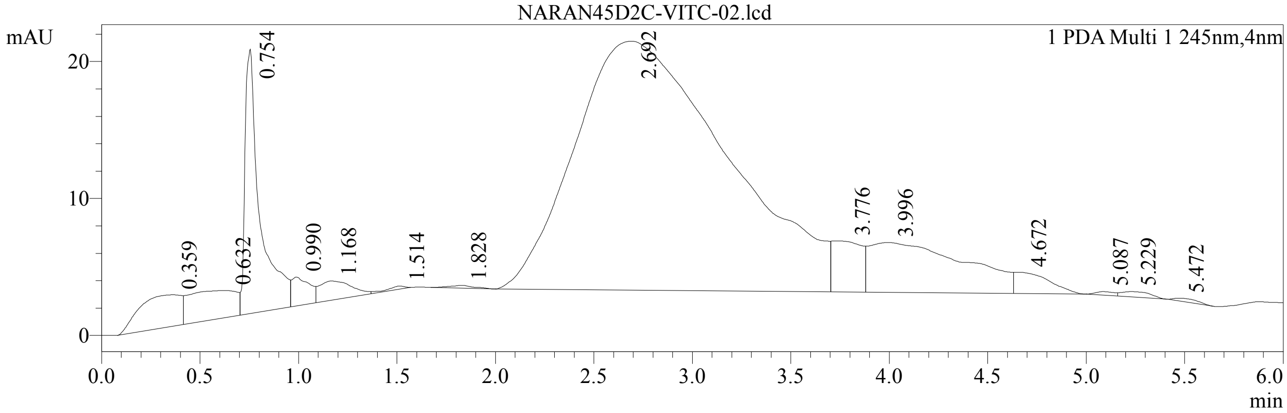
NARAN45D2C-VITC-02.lcd
mAU
1 PDA Multi 1 245nm,4nm
0.359
0.632
0.754
0.990
1.168
1.514
1.828
2.692
3.776
3.996
4.672
5.087
5.229
5.472
min

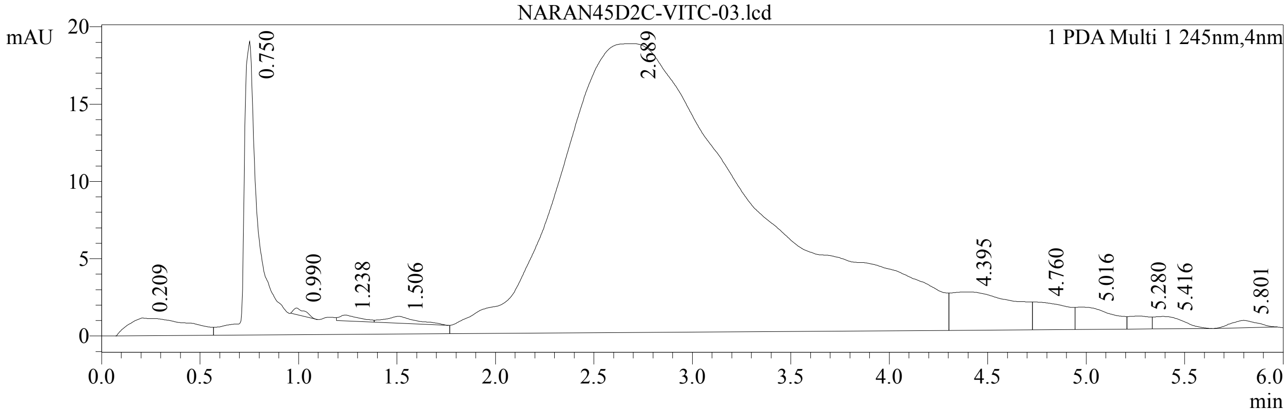
NARAN45D2C-VITC-03.lcd
mAU
1 PDA Multi 1 245nm,4nm
0.209
0.750
0.990
1.238
1.506
2.689
4.395
4.760
5.016
5.280
5.416
5.801
min

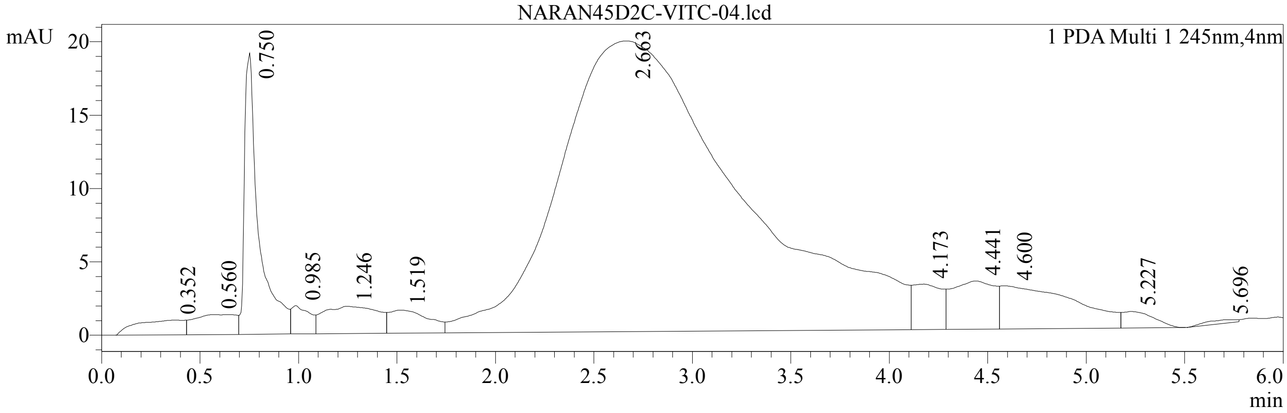
NARAN45D2C-VITC-04.lcd
mAU
1 PDA Multi 1 245nm,4nm
0.352
0.560
0.750
0.985
1.246
1.519
2.663
4.173
4.441
4.600
5.227
5.696
min

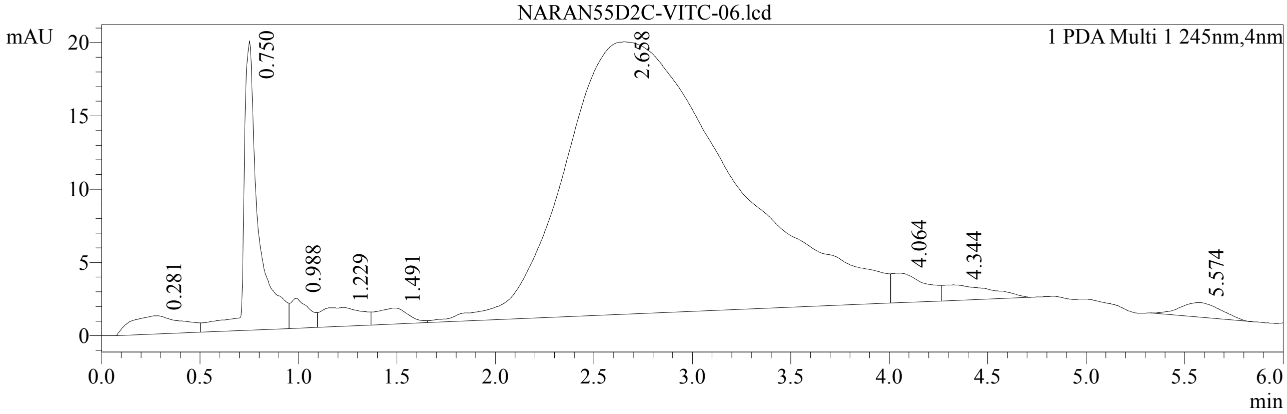
NARAN55D2C-VITC-06.lcd
mAU
1 PDA Multi 1 245nm,4nm
0.281
0.750
0.988
1.229
1.491
2.658
4.064
4.344
5.574
min

NARAN55D2C-VITC-07.lcd

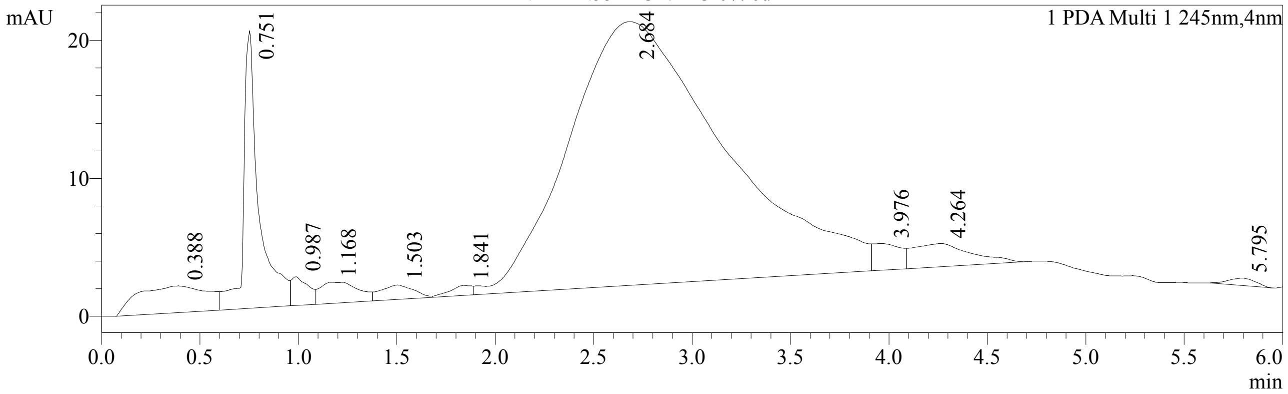

NARAN55D2C-VITC-08.lcd

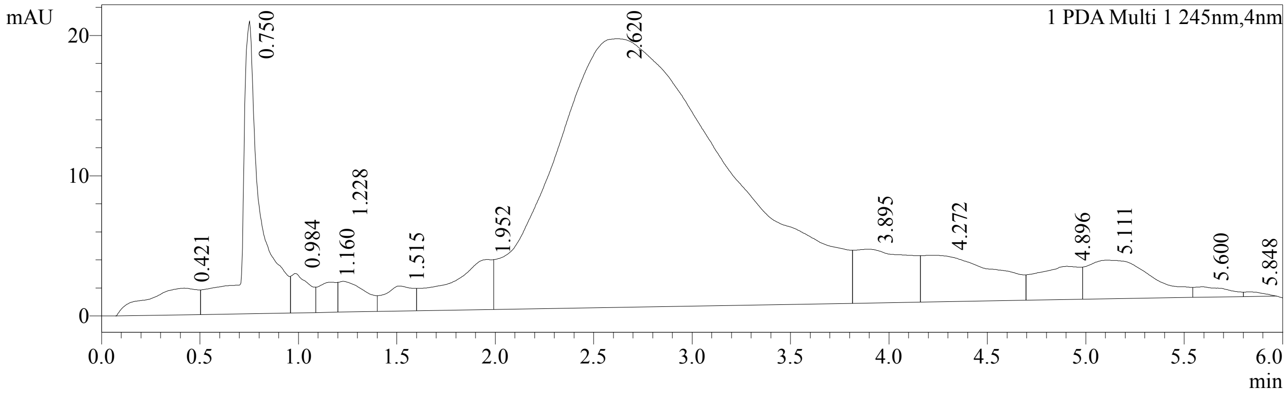

NARAN55D2C-VITC-05.lcd

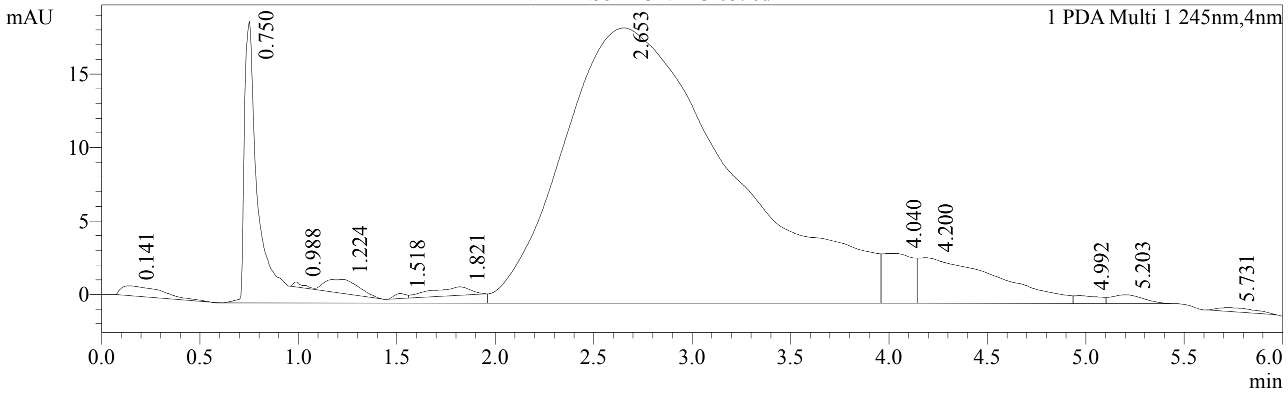

NARAN35D3C-VITC-01.lcd

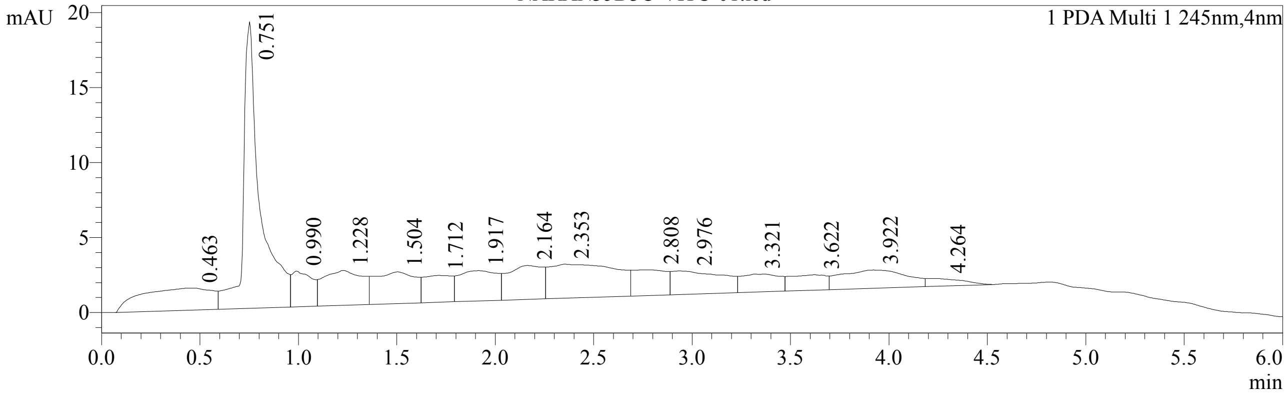

NARAN35D3C-VITC-02.lcd

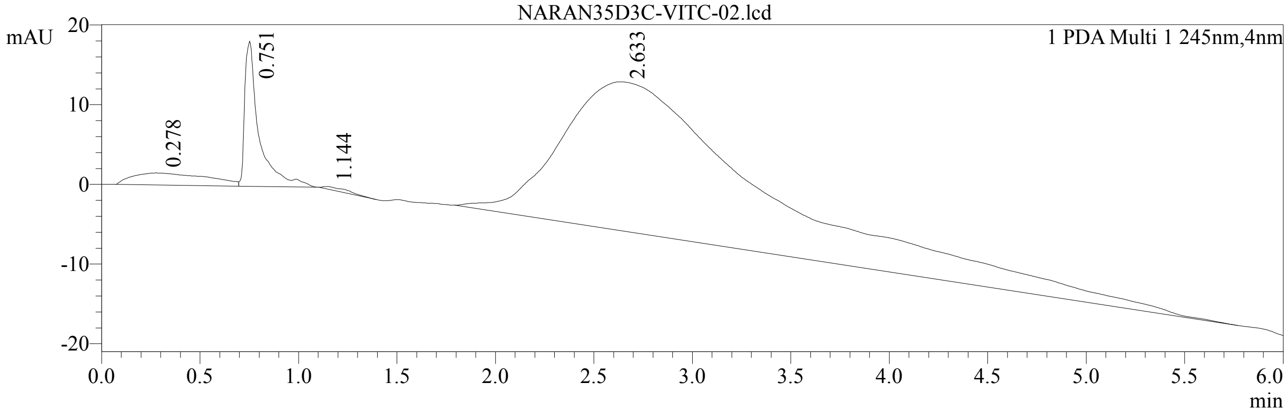

NARAN35D3C-VITC-03.lcd

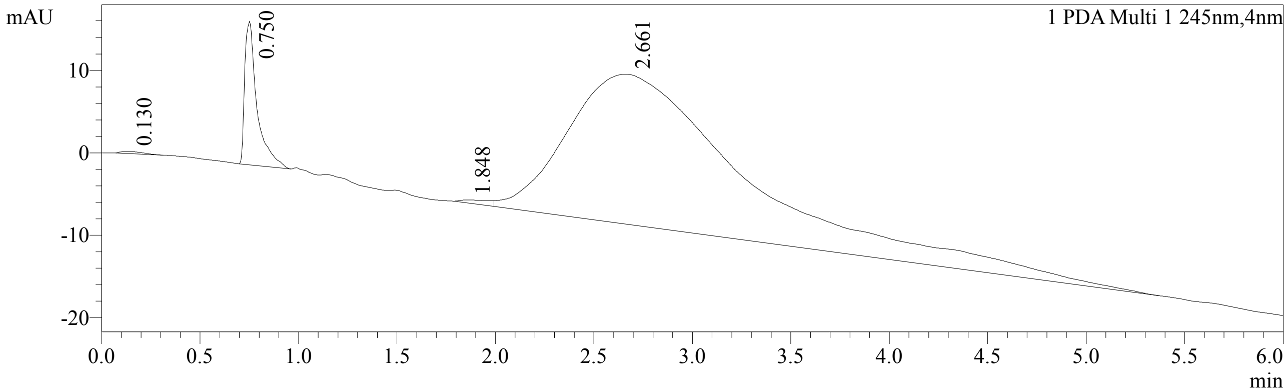

NARAN35D3C-VITC-04.lcd

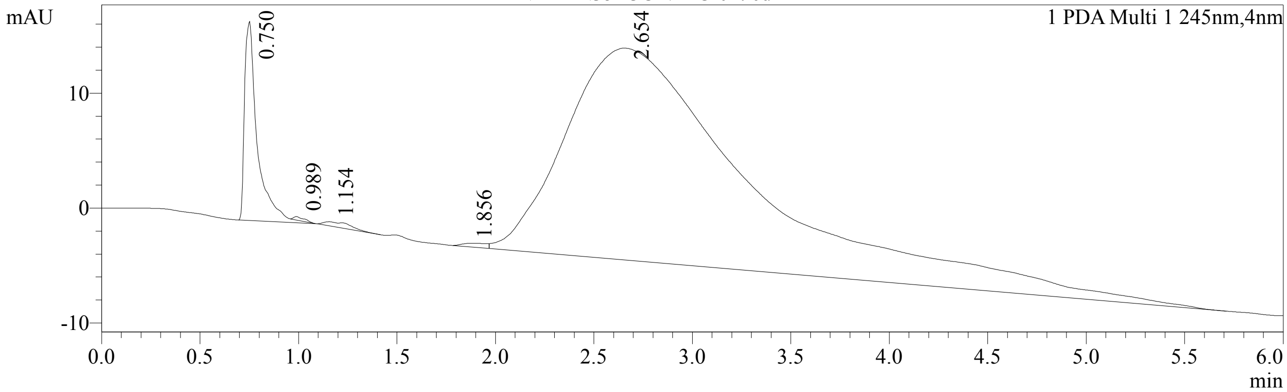

NARAN55D3C-VITC-05.lcd

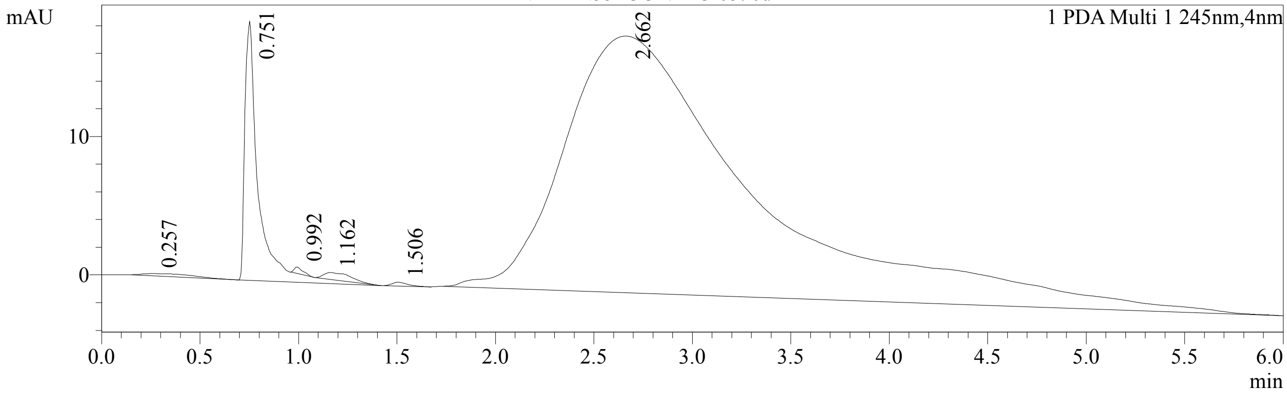

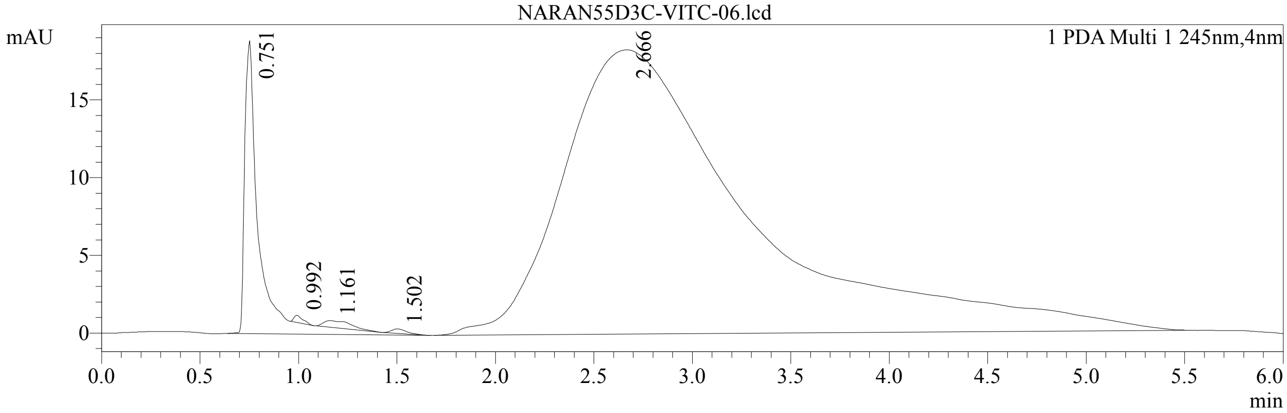
NARAN55D3C-VITC-06.lcd
mAU
1 PDA Multi 1 245nm,4nm
0.751
0.992
1.161
1.502
2.666
0 5 10 15
0.0 0.5 1.0 1.5 2.0 2.5 3.0 3.5 4.0 4.5 5.0 5.5 6.0
min

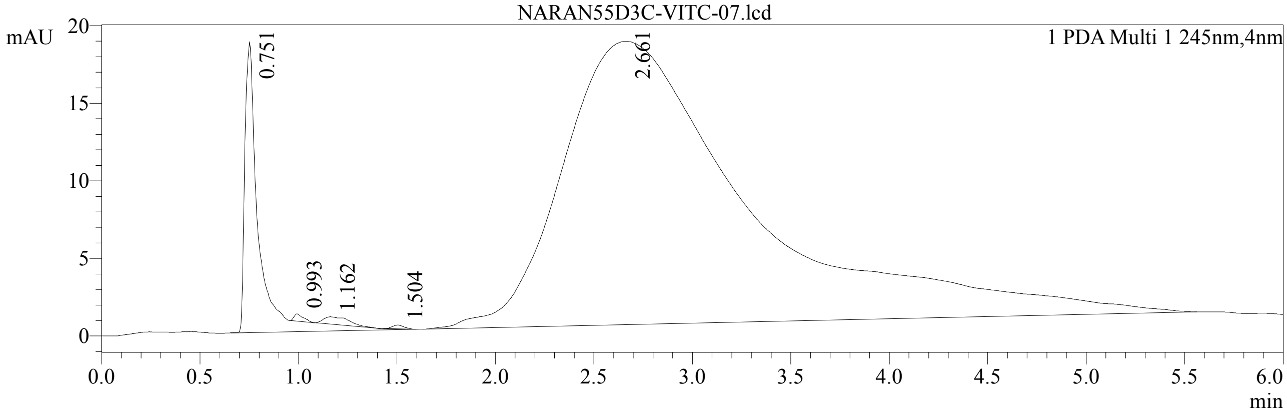
NARAN55D3C-VITC-07.lcd
mAU
1 PDA Multi 1 245nm,4nm
0.751
0.993
1.162
1.504
2.661
0 5 10 15 20
0.0 0.5 1.0 1.5 2.0 2.5 3.0 3.5 4.0 4.5 5.0 5.5 6.0
min

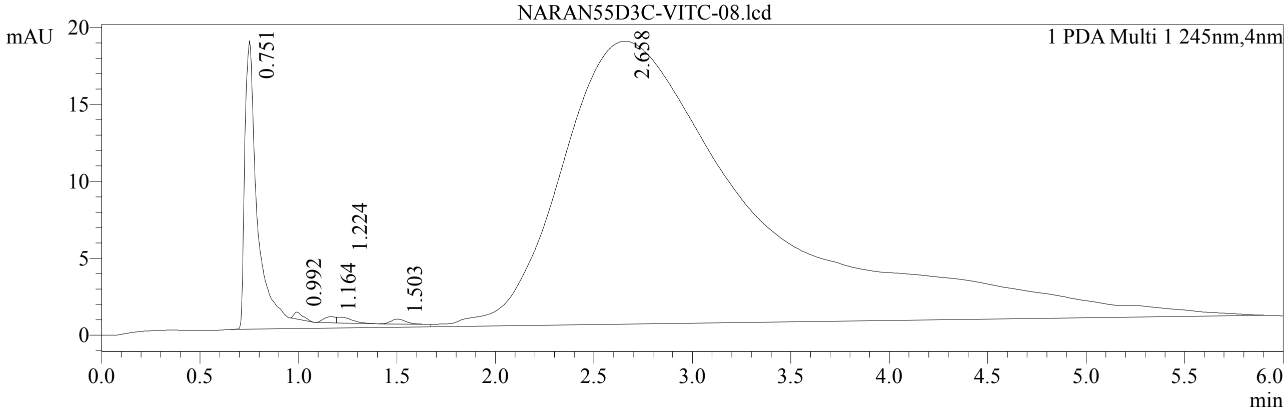
NARAN55D3C-VITC-08.lcd
mAU
1 PDA Multi 1 245nm,4nm
0.751
0.992
1.164
1.224
1.503
2.658
0 5 10 15 20
0.0 0.5 1.0 1.5 2.0 2.5 3.0 3.5 4.0 4.5 5.0 5.5 6.0
min

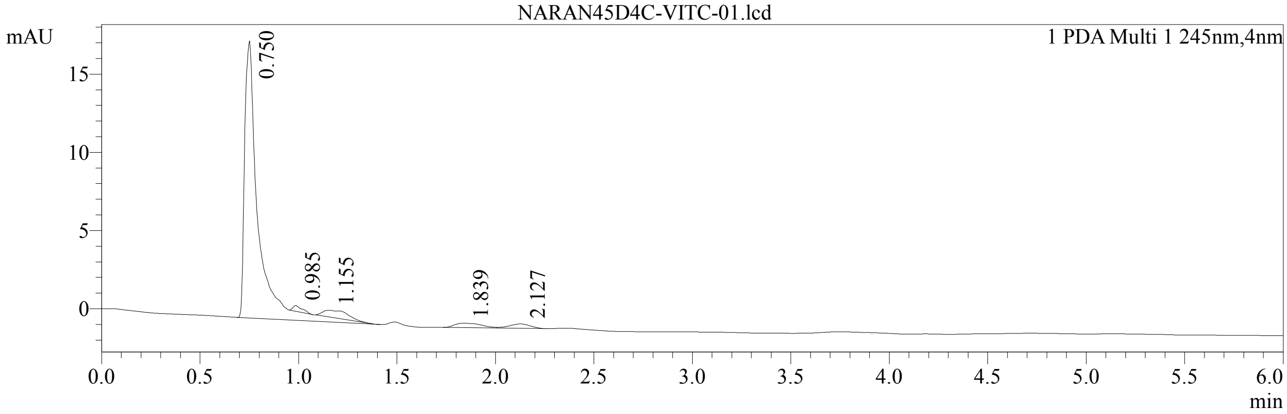
NARAN45D4C-VITC-01.lcd
mAU
1 PDA Multi 1 245nm,4nm
0.750
0.985
1.155
1.839
2.127
0 5 10 15
0.0 0.5 1.0 1.5 2.0 2.5 3.0 3.5 4.0 4.5 5.0 5.5 6.0
min

NARAN45D4C-VITC-02.lcd

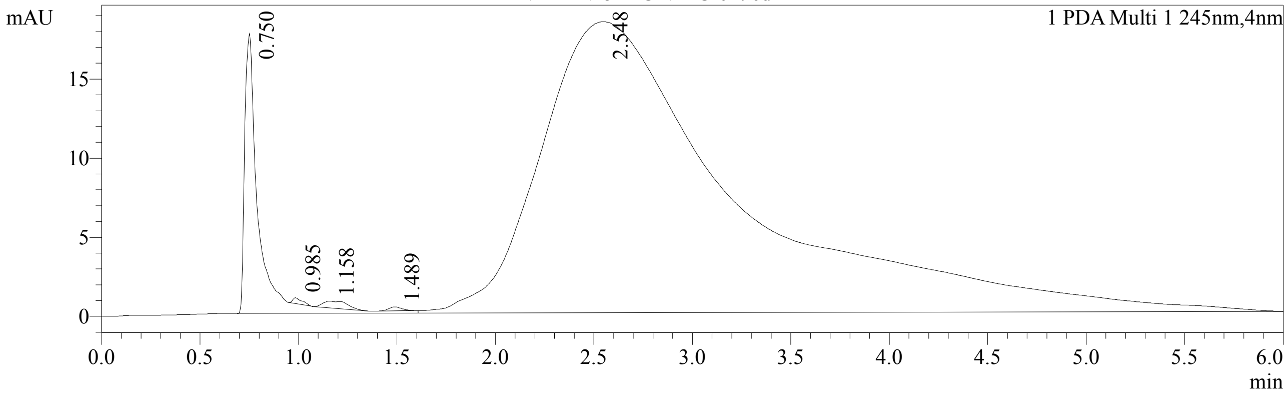

NARAN45D4C-VITC-03.lcd

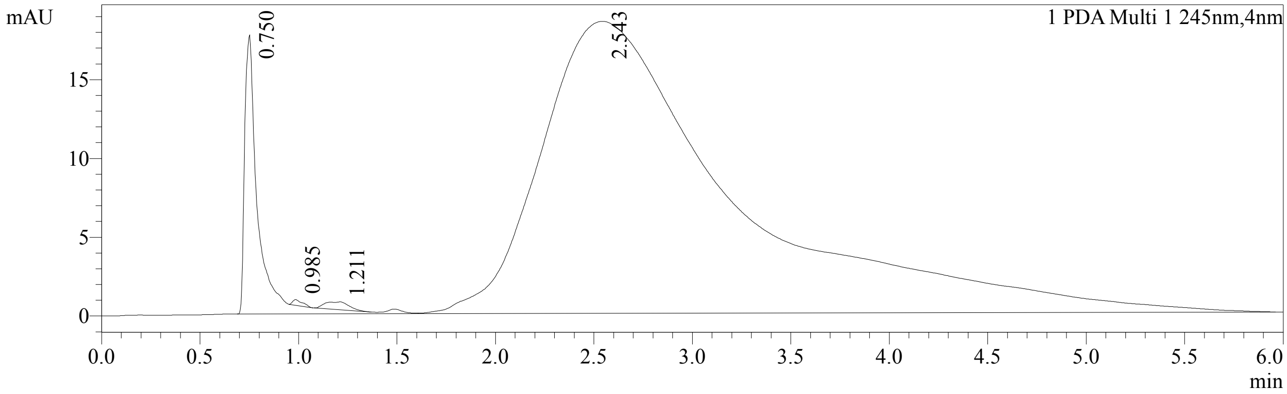

NARAN45D4C-VITC-04.lcd

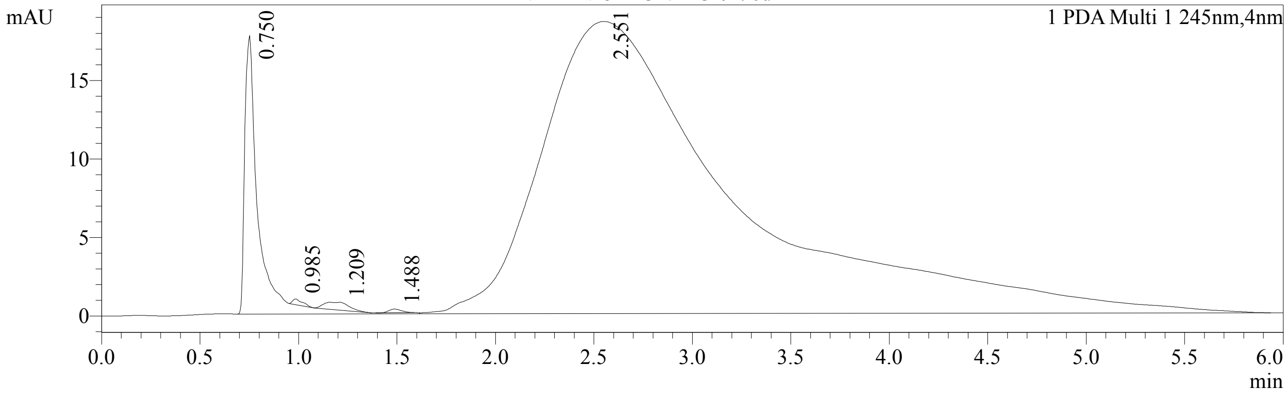

NARAN55D4C-VITC-05.lcd

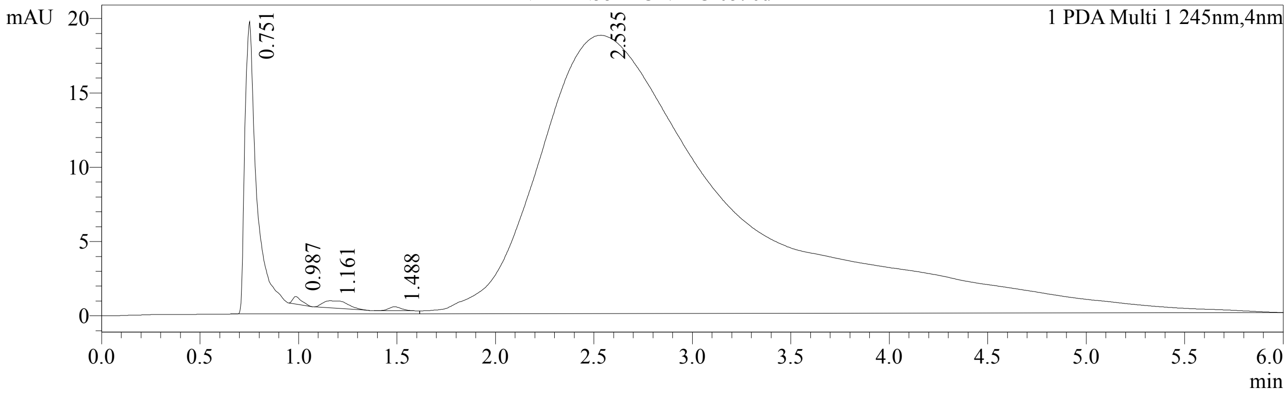

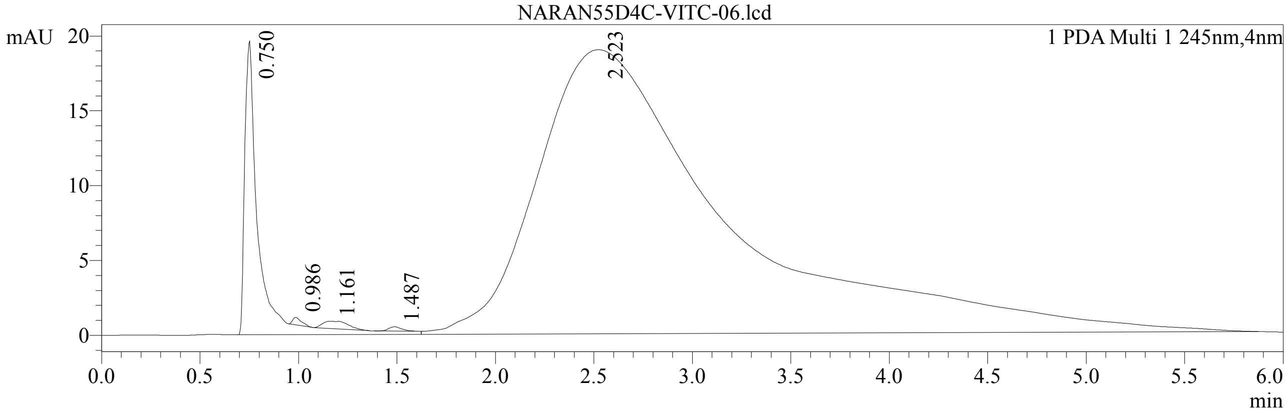
NARAN55D4C-VITC-06.lcd
mAU
1 PDA Multi 1 245nm,4nm
0.750
0.986
1.161
1.487
2.523
min

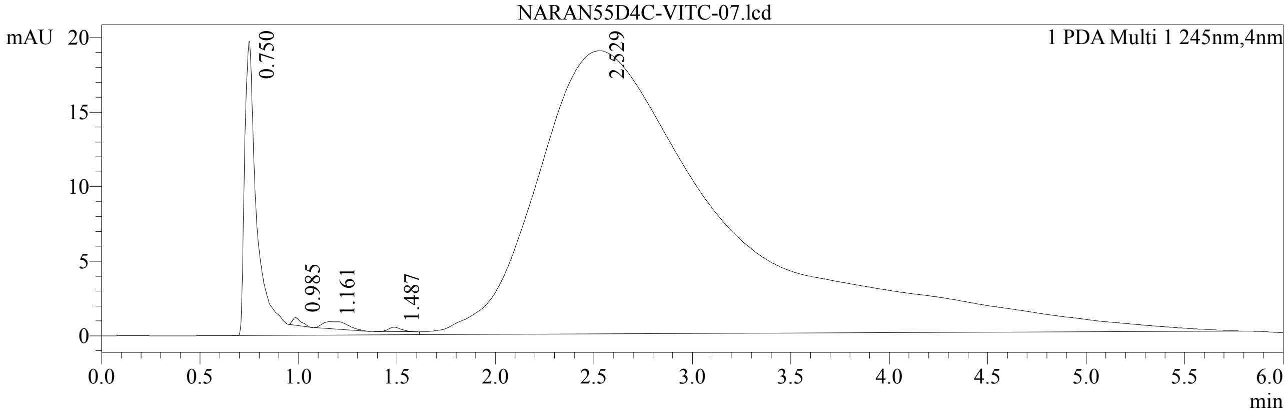
NARAN55D4C-VITC-07.lcd
mAU
1 PDA Multi 1 245nm,4nm
0.750
0.985
1.161
1.487
2.529
min

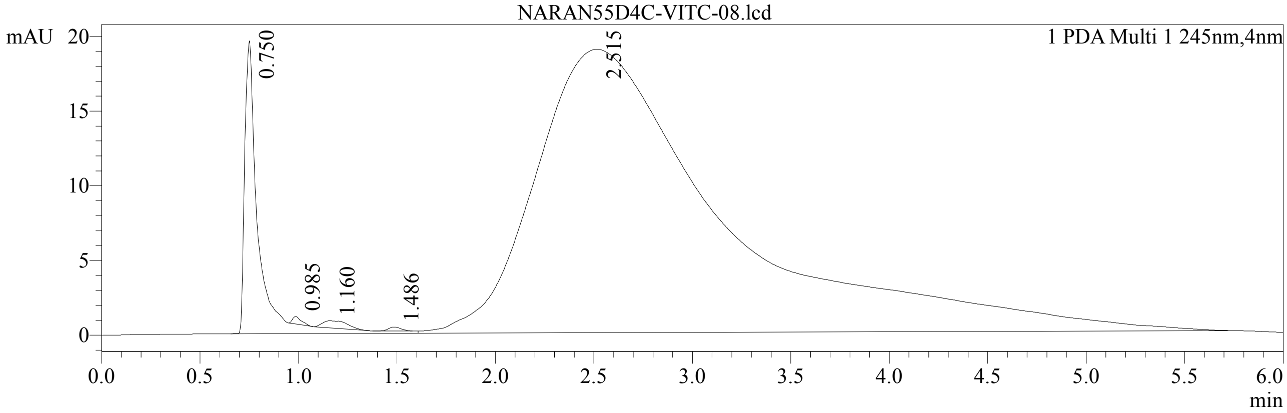
NARAN55D4C-VITC-08.lcd
mAU
1 PDA Multi 1 245nm,4nm
0.750
0.985
1.160
1.486
2.515
min

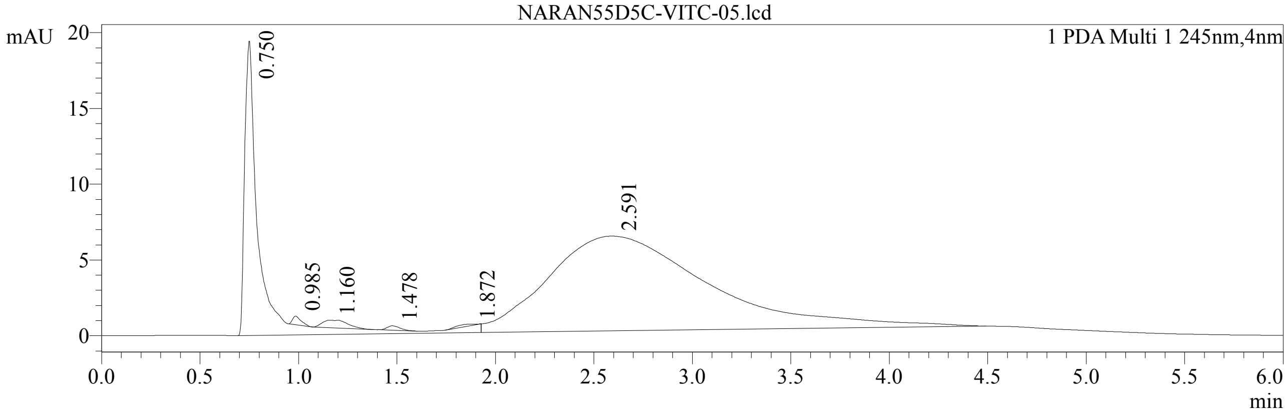
NARAN55D5C-VITC-05.lcd
mAU
1 PDA Multi 1 245nm,4nm
0.750
0.985
1.160
1.478
1.872
2.591
min

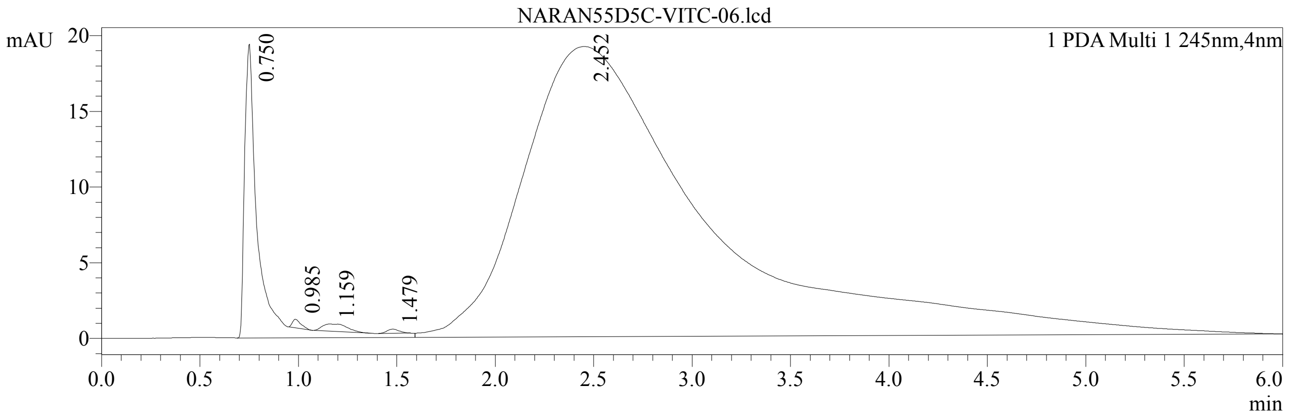
NARAN55D5C-VITC-06.lcd
mAU
1 PDA Multi 1 245nm,4nm
0.750
0.985
1.159
1.479
2.452
min

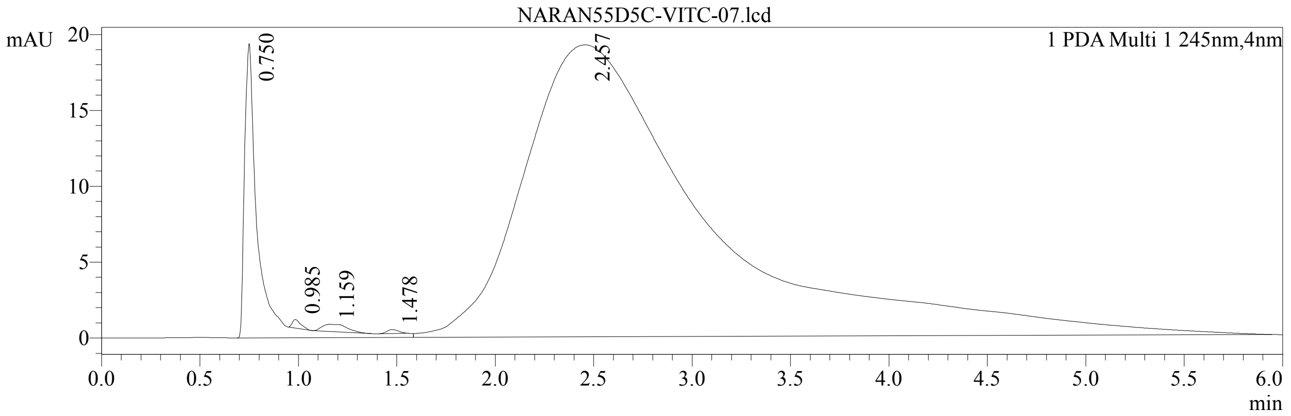
NARAN55D5C-VITC-07.lcd
mAU
1 PDA Multi 1 245nm,4nm
0.750
0.985
1.159
1.478
2.457
min

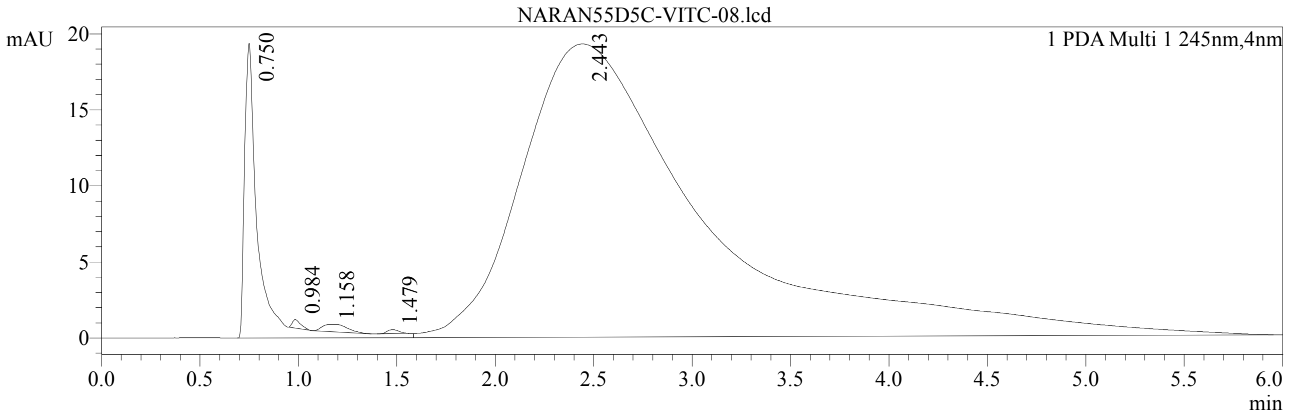
NARAN55D5C-VITC-08.lcd
mAU
1 PDA Multi 1 245nm,4nm
0.750
0.984
1.158
1.479
2.443
min

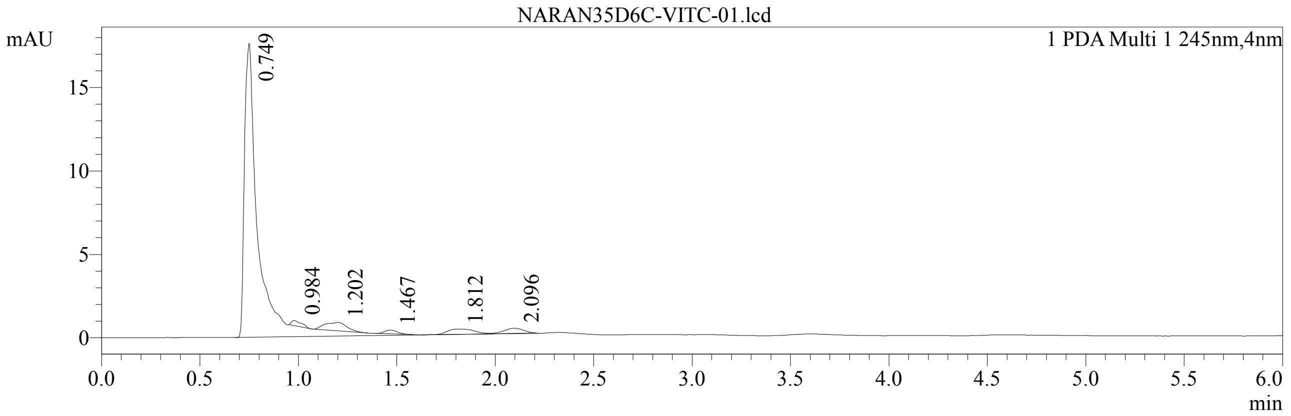
NARAN35D6C-VITC-01.lcd
mAU
1 PDA Multi 1 245nm,4nm
0.749
0.984
1.202
1.467
1.812
2.096
min

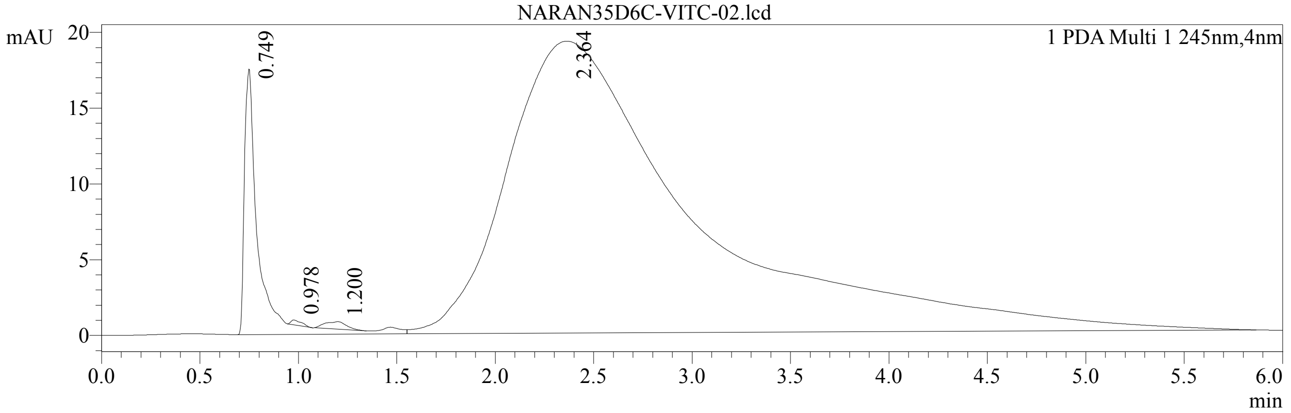
NARAN35D6C-VITC-02.lcd
mAU
1 PDA Multi 1 245nm,4nm
0.749
0.978
1.200
2.364
min

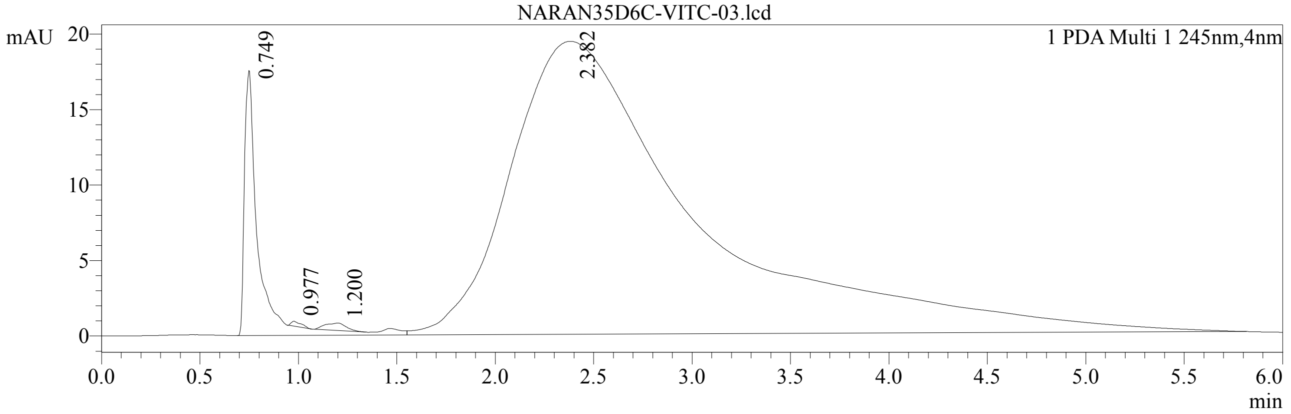
NARAN35D6C-VITC-03.lcd
mAU
1 PDA Multi 1 245nm,4nm
0.749
0.977
1.200
2.382
min

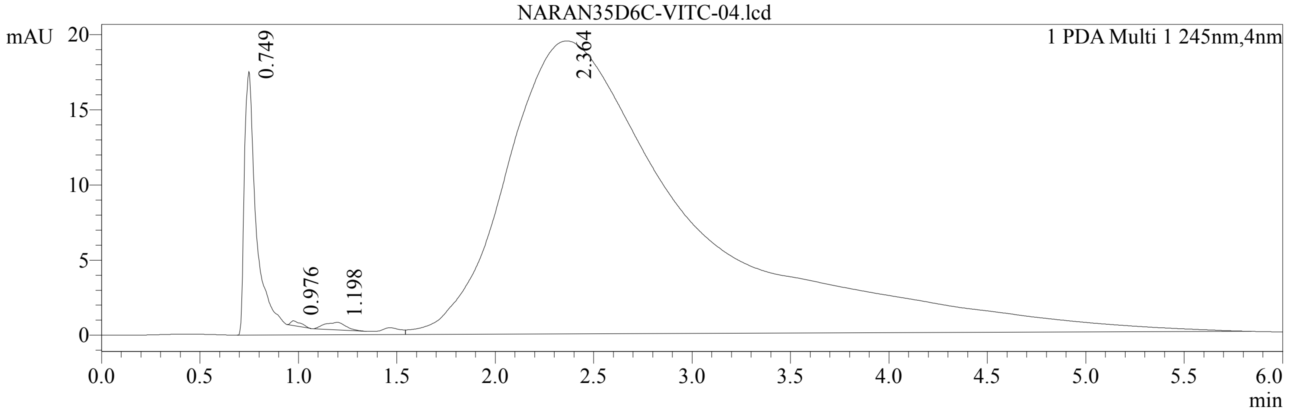
NARAN35D6C-VITC-04.lcd
mAU
1 PDA Multi 1 245nm,4nm
0.749
0.976
1.198
2.364
min

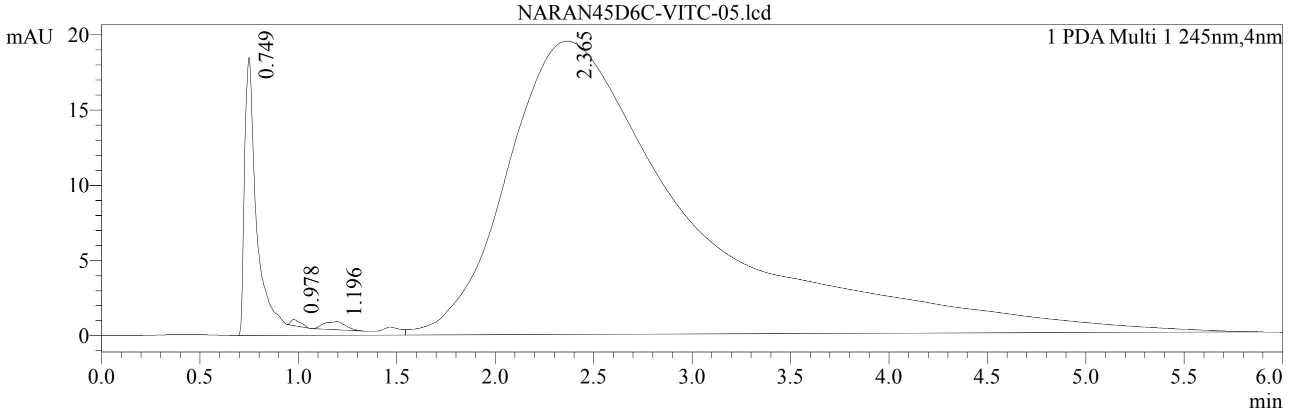
NARAN45D6C-VITC-05.lcd
mAU
1 PDA Multi 1 245nm,4nm
0.749
0.978
1.196
2.365
min

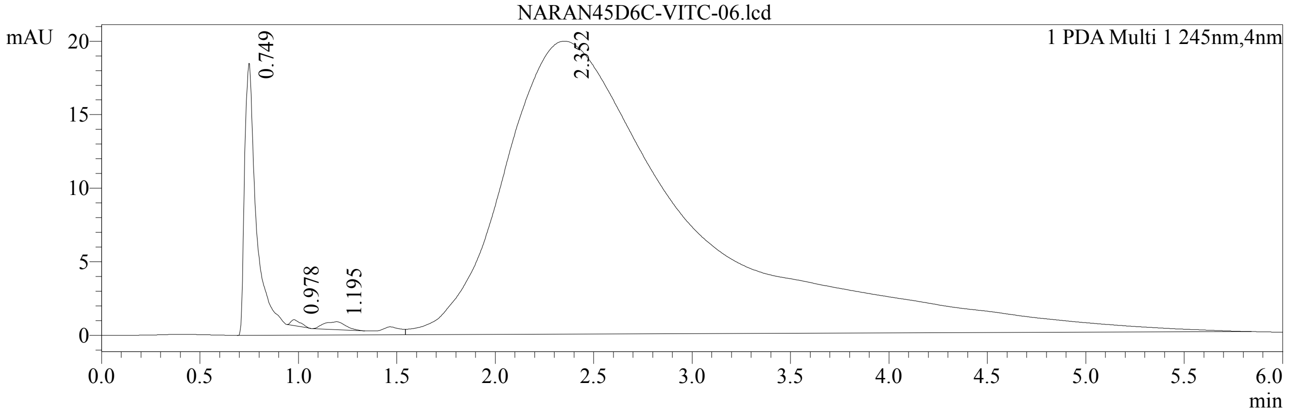
NARAN45D6C-VITC-06.lcd
mAU
1 PDA Multi 1 245nm,4nm
0.749
0.978
1.195
2.352
0 5 10 15 20
0.0 0.5 1.0 1.5 2.0 2.5 3.0 3.5 4.0 4.5 5.0 5.5 6.0
min

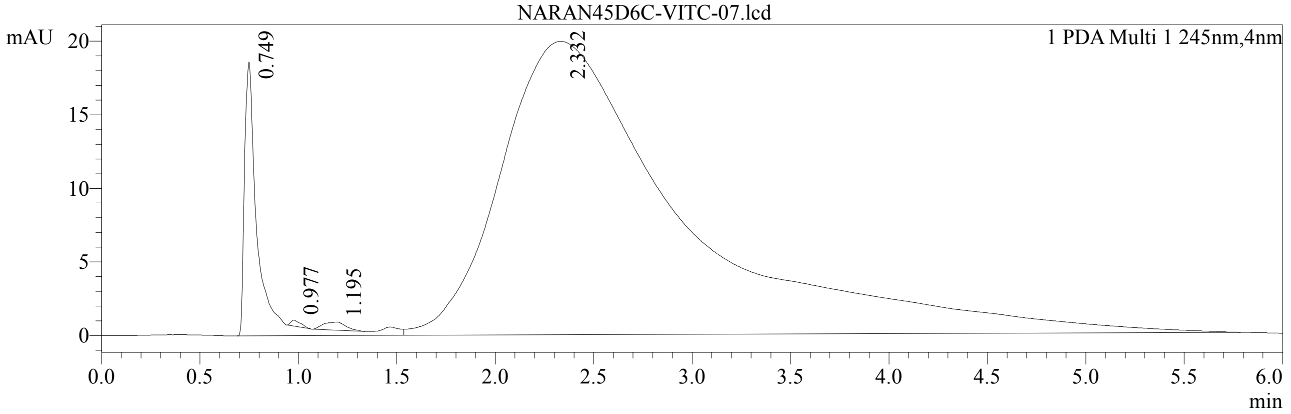
NARAN45D6C-VITC-07.lcd
mAU
1 PDA Multi 1 245nm,4nm
0.749
0.977
1.195
2.332
0 5 10 15 20
0.0 0.5 1.0 1.5 2.0 2.5 3.0 3.5 4.0 4.5 5.0 5.5 6.0
min

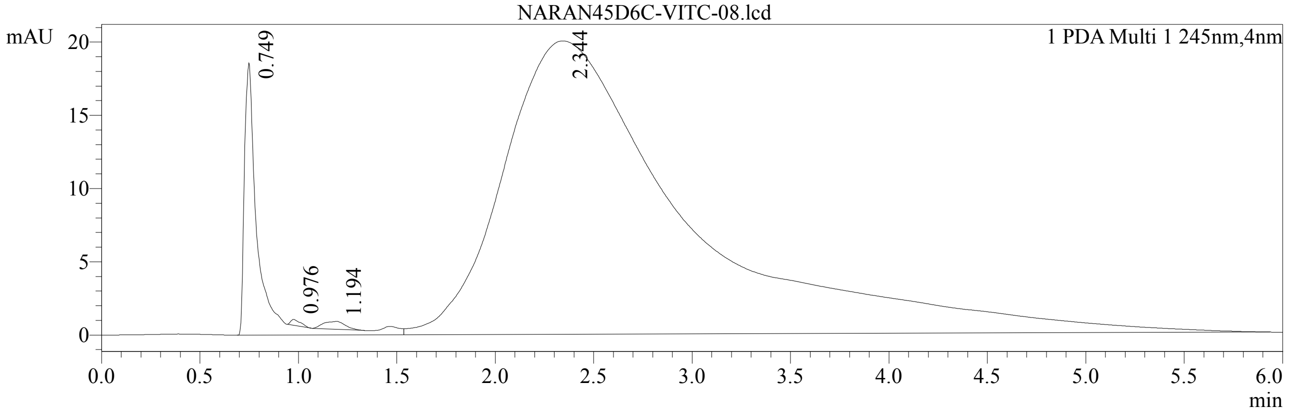
NARAN45D6C-VITC-08.lcd
mAU
1 PDA Multi 1 245nm,4nm
0.749
0.976
1.194
2.344
0 5 10 15 20
0.0 0.5 1.0 1.5 2.0 2.5 3.0 3.5 4.0 4.5 5.0 5.5 6.0
min

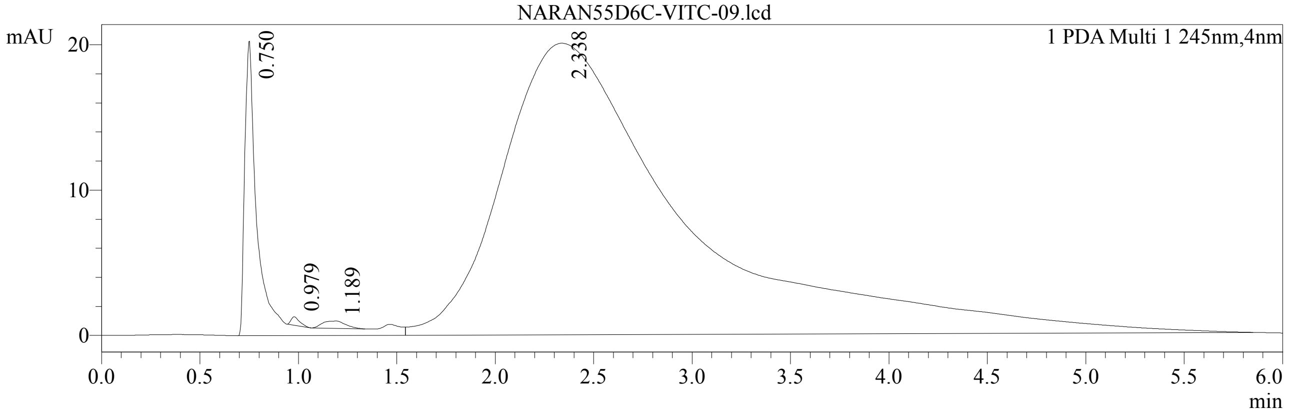
NARAN55D6C-VITC-09.lcd
mAU
1 PDA Multi 1 245nm,4nm
0.750
0.979
1.189
2.338
0 10 20
0.0 0.5 1.0 1.5 2.0 2.5 3.0 3.5 4.0 4.5 5.0 5.5 6.0
min

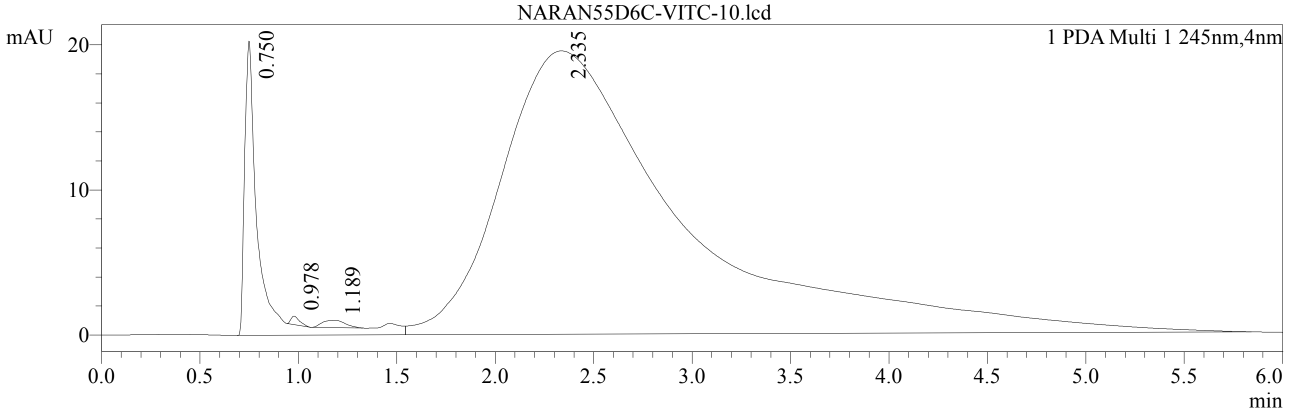
NARAN55D6C-VITC-10.lcd
mAU
1 PDA Multi 1 245nm,4nm
0.750
0.978
1.189
2.335
min

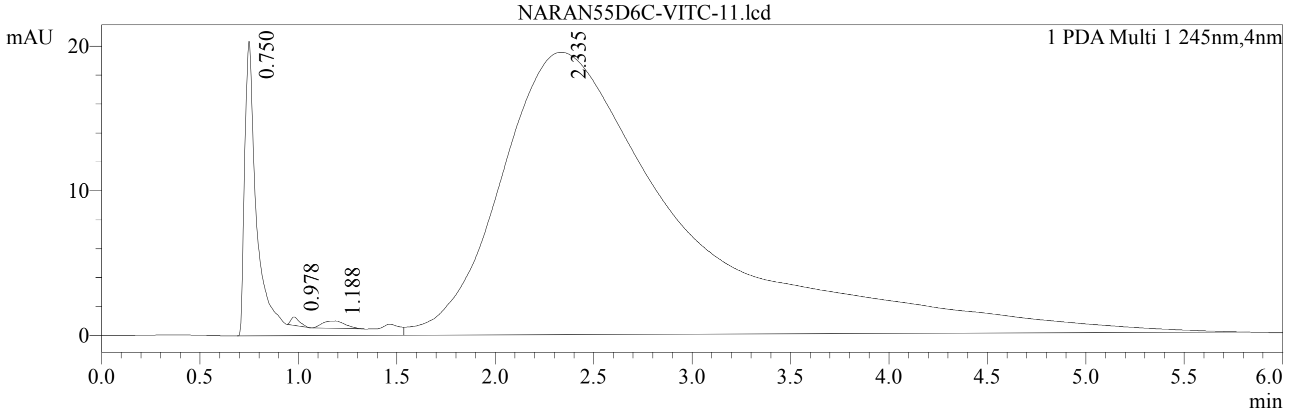
NARAN55D6C-VITC-11.lcd
mAU
1 PDA Multi 1 245nm,4nm
0.750
0.978
1.188
2.335
min

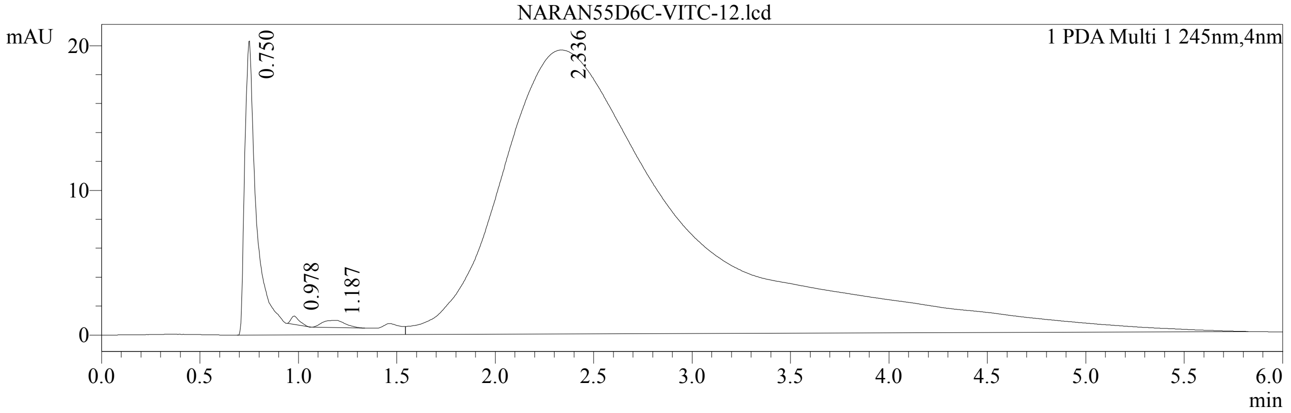
NARAN55D6C-VITC-12.lcd
mAU
1 PDA Multi 1 245nm,4nm
0.750
0.978
1.187
2.336
min

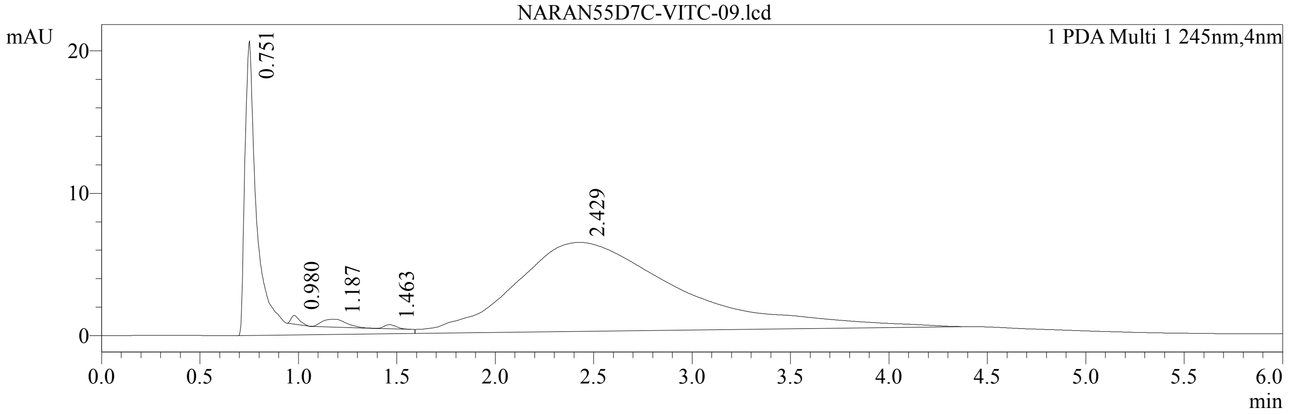
NARAN55D7C-VITC-09.lcd
mAU
1 PDA Multi 1 245nm,4nm
0.751
0.980
1.187
1.463
2.429
min

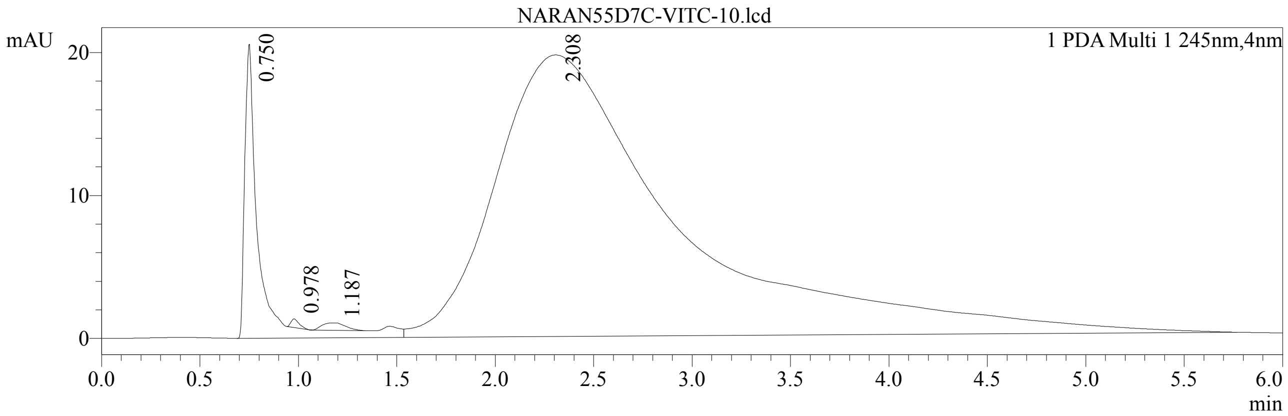
NARAN55D7C-VITC-10.lcd
mAU
1 PDA Multi 1 245nm,4nm
0.750
0.978
1.187
2.308
min

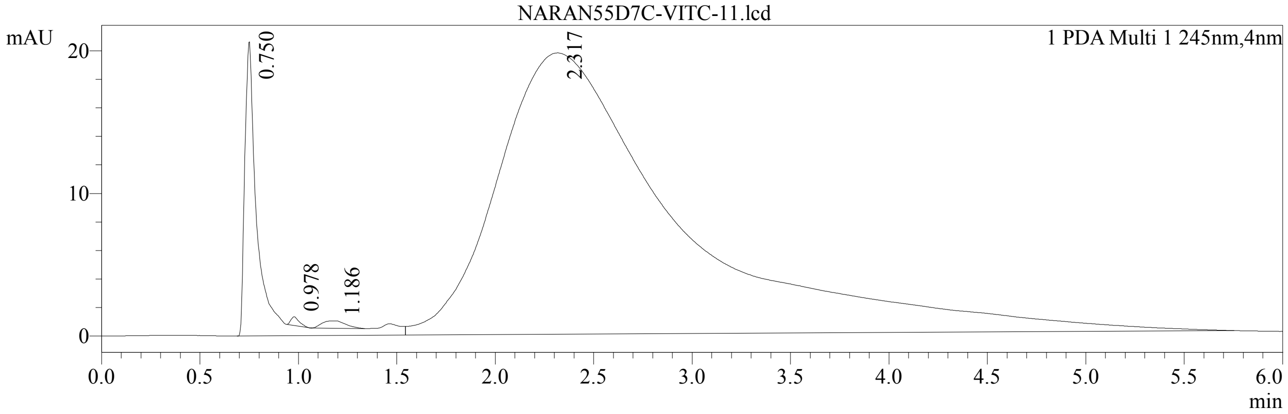
NARAN55D7C-VITC-11.lcd
mAU
1 PDA Multi 1 245nm,4nm
0.750
0.978
1.186
2.317
min

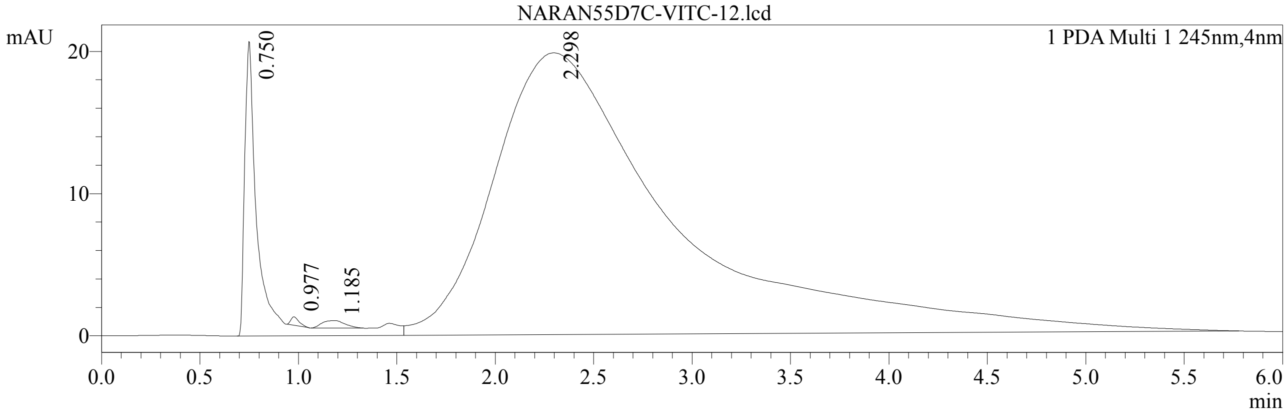
NARAN55D7C-VITC-12.lcd
mAU
1 PDA Multi 1 245nm,4nm
0.750
0.977
1.185
2.298
min

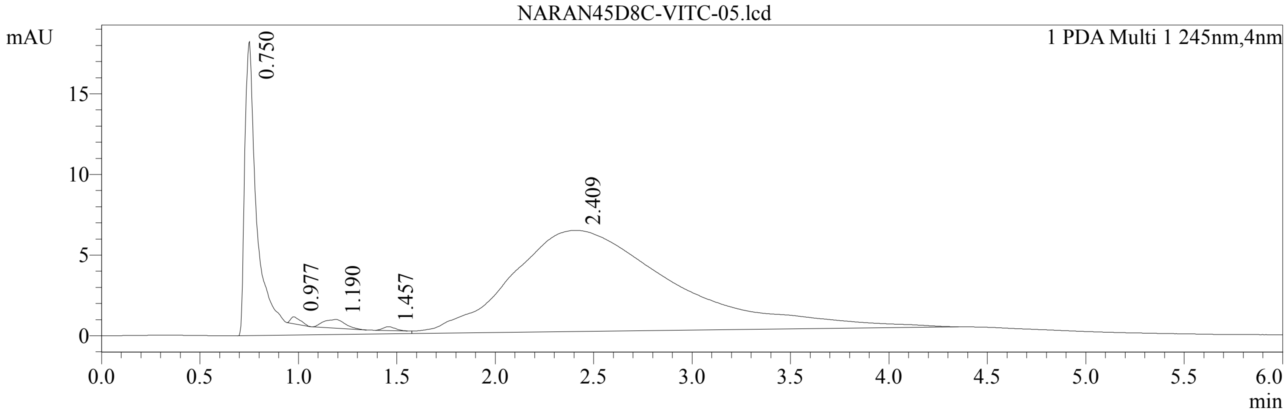
NARAN45D8C-VITC-05.lcd
mAU
1 PDA Multi 1 245nm,4nm
0.750
0.977
1.190
1.457
2.409
min

NARAN45D8C-VITC-06.lcd

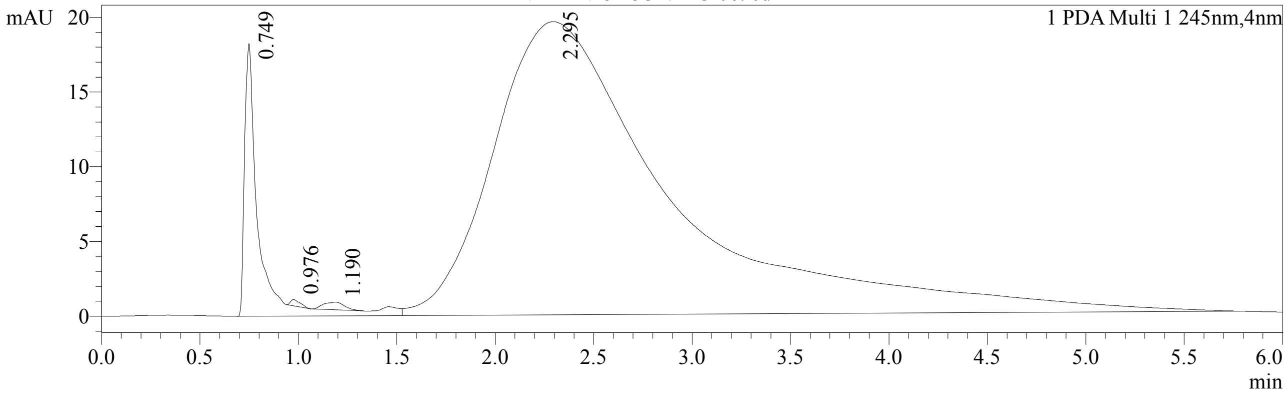

NARAN45D8C-VITC-07.lcd

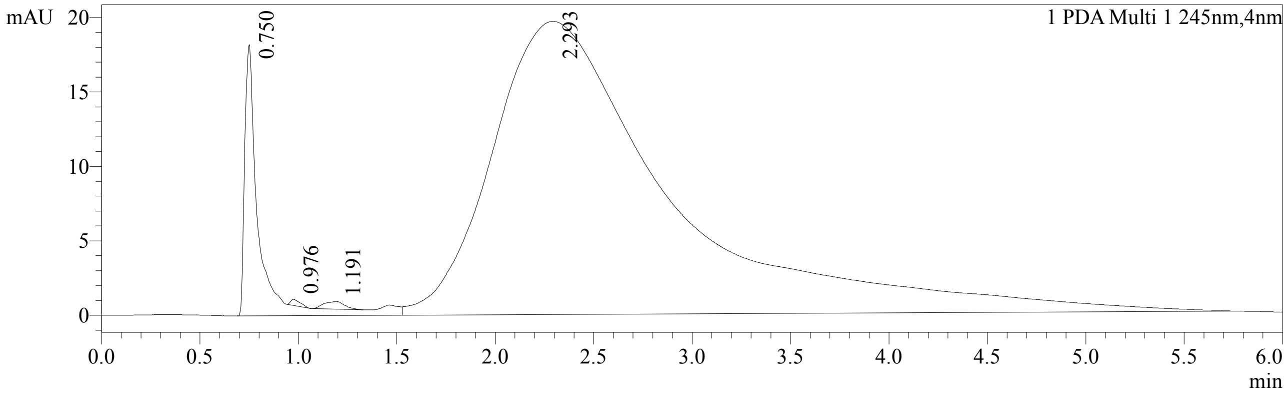

NARAN45D8C-VITC-08.lcd

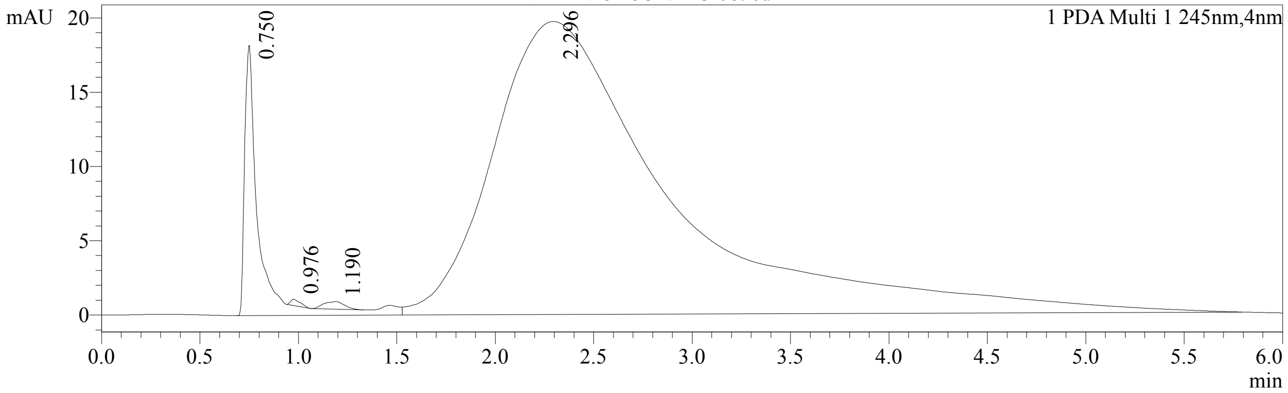

NARAN35D9C-VITC-05.lcd

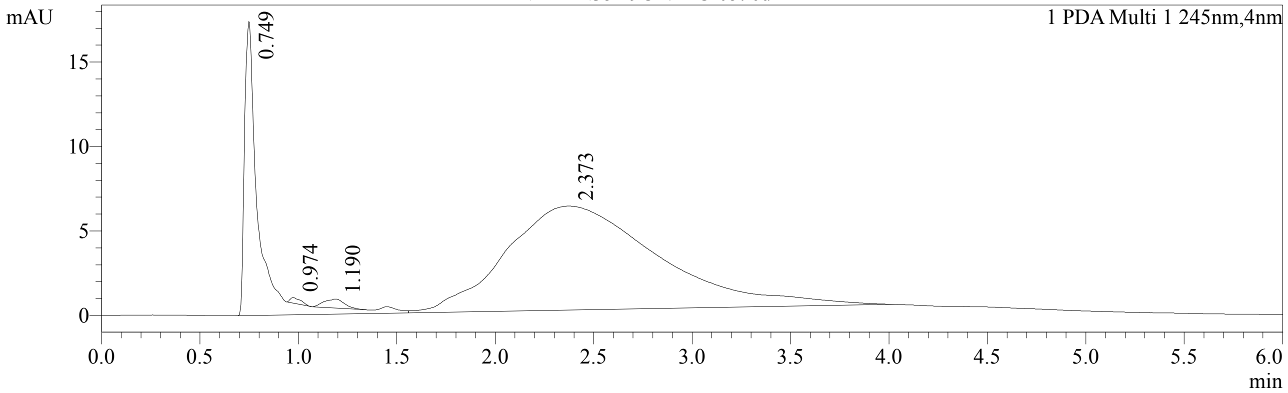

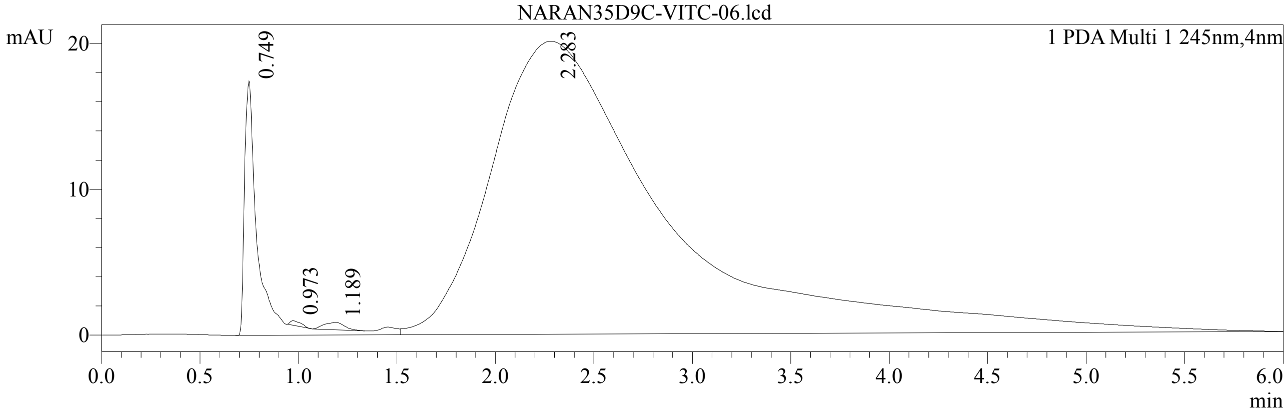
NARAN35D9C-VITC-06.lcd
mAU
1 PDA Multi 1 245nm,4nm
0.749
0.973
1.189
2.283
0 10 20
0.0 0.5 1.0 1.5 2.0 2.5 3.0 3.5 4.0 4.5 5.0 5.5 6.0
min

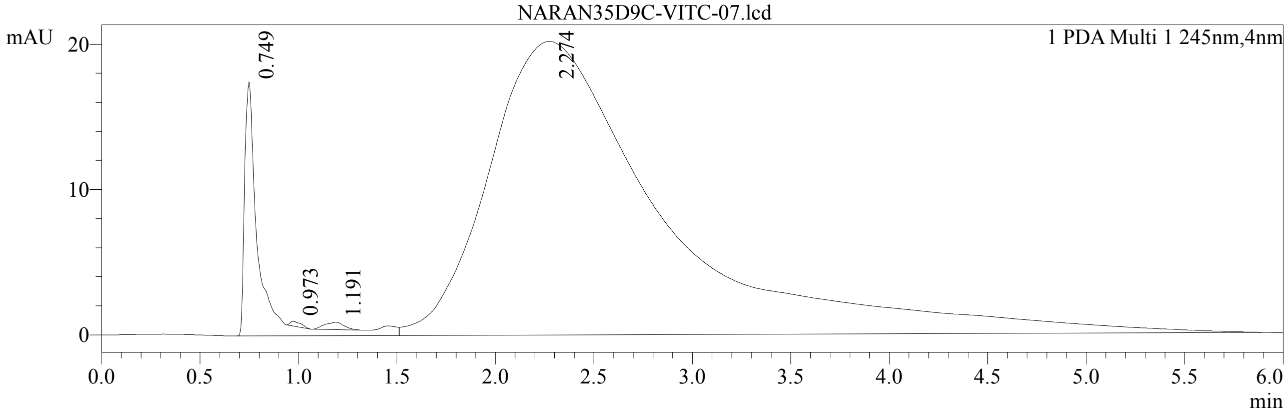
NARAN35D9C-VITC-07.lcd
mAU
1 PDA Multi 1 245nm,4nm
0.749
0.973
1.191
2.274
0 10 20
0.0 0.5 1.0 1.5 2.0 2.5 3.0 3.5 4.0 4.5 5.0 5.5 6.0
min

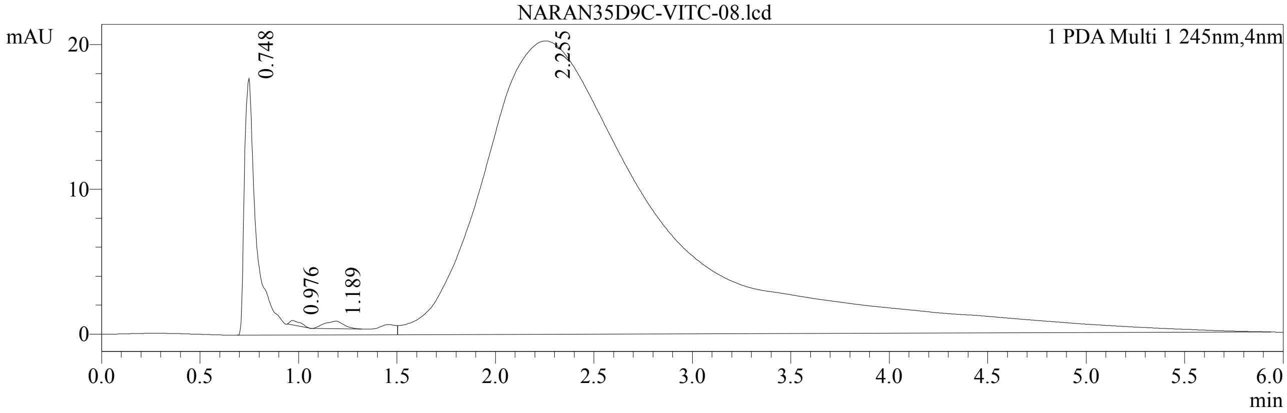
NARAN35D9C-VITC-08.lcd
mAU
1 PDA Multi 1 245nm,4nm
0.748
0.976
1.189
2.255
0 10 20
0.0 0.5 1.0 1.5 2.0 2.5 3.0 3.5 4.0 4.5 5.0 5.5 6.0
min

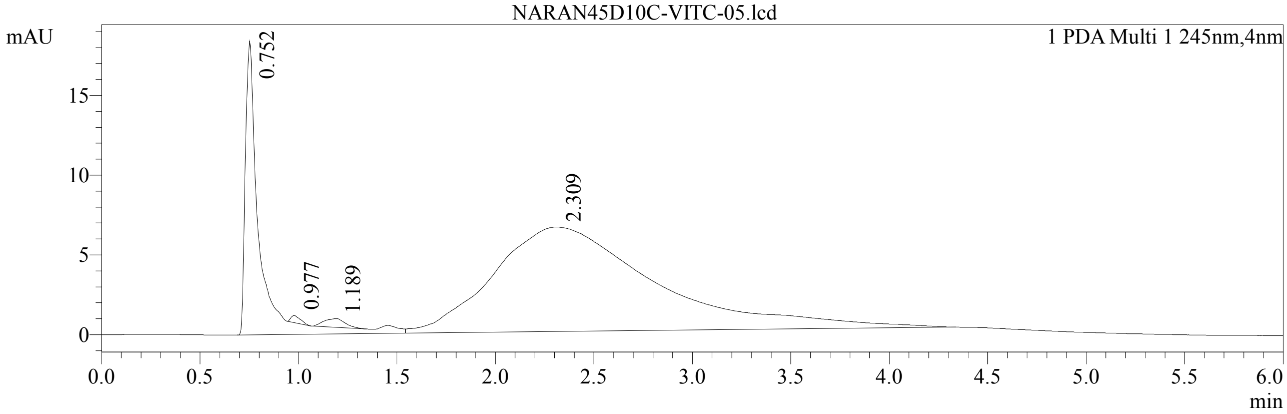
NARAN45D10C-VITC-05.lcd
mAU
1 PDA Multi 1 245nm,4nm
0.752
0.977
1.189
2.309
0 5 10 15
0.0 0.5 1.0 1.5 2.0 2.5 3.0 3.5 4.0 4.5 5.0 5.5 6.0
min

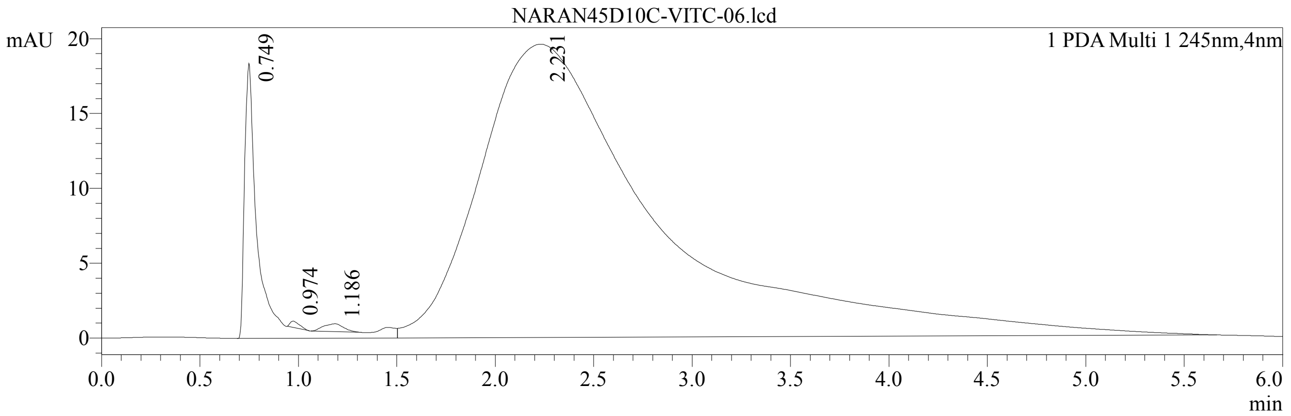
NARAN45D10C-VITC-06.lcd
mAU
1 PDA Multi 1 245nm,4nm
0.749
0.974
1.186
2.231
0 5 10 15 20
0.0 0.5 1.0 1.5 2.0 2.5 3.0 3.5 4.0 4.5 5.0 5.5 6.0
min

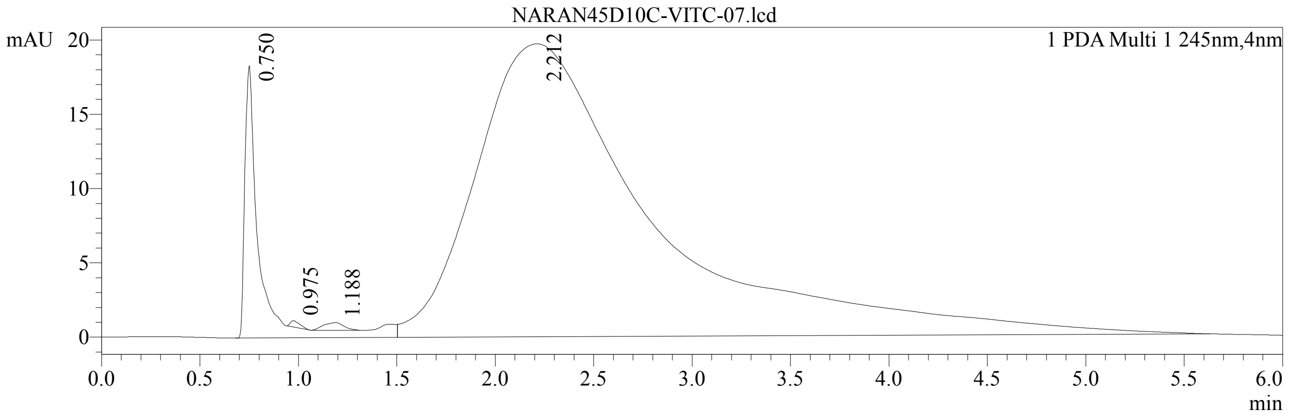
NARAN45D10C-VITC-07.lcd
mAU
1 PDA Multi 1 245nm,4nm
0.750
0.975
1.188
2.212
0 5 10 15 20
0.0 0.5 1.0 1.5 2.0 2.5 3.0 3.5 4.0 4.5 5.0 5.5 6.0
min

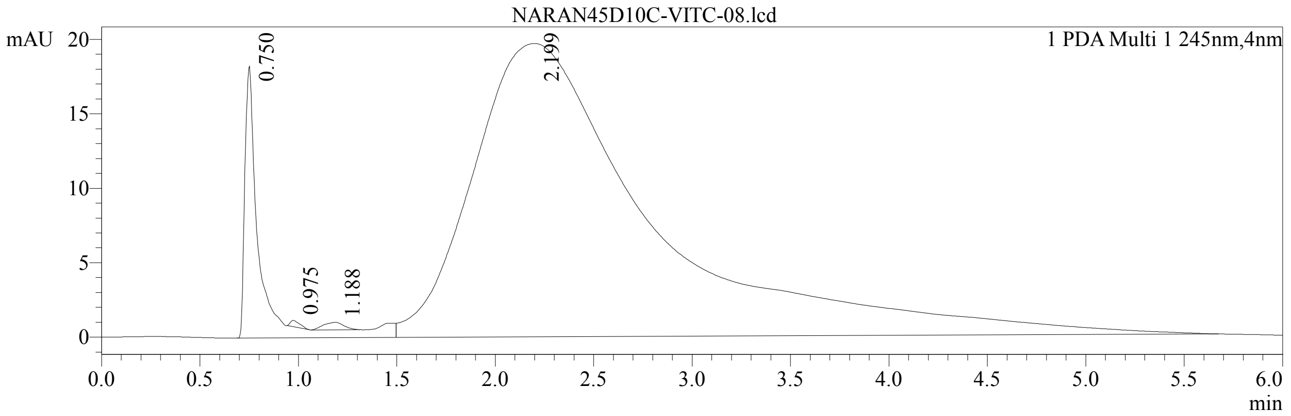
NARAN45D10C-VITC-08.lcd
mAU
1 PDA Multi 1 245nm,4nm
0.750
0.975
1.188
2.199
0 5 10 15 20
0.0 0.5 1.0 1.5 2.0 2.5 3.0 3.5 4.0 4.5 5.0 5.5 6.0
min

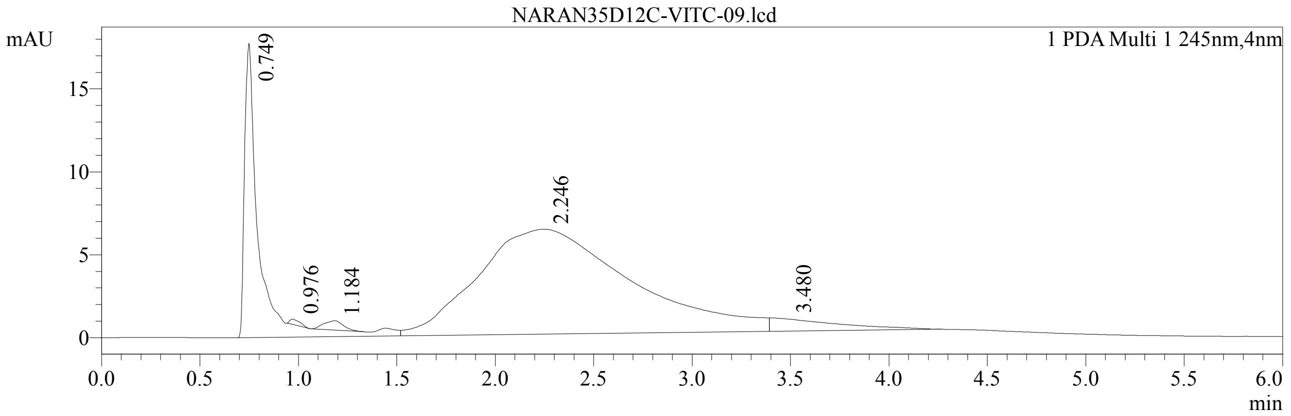
NARAN35D12C-VITC-09.lcd
mAU
1 PDA Multi 1 245nm,4nm
0.749
0.976
1.184
2.246
3.480
0 5 10 15
0.0 0.5 1.0 1.5 2.0 2.5 3.0 3.5 4.0 4.5 5.0 5.5 6.0
min

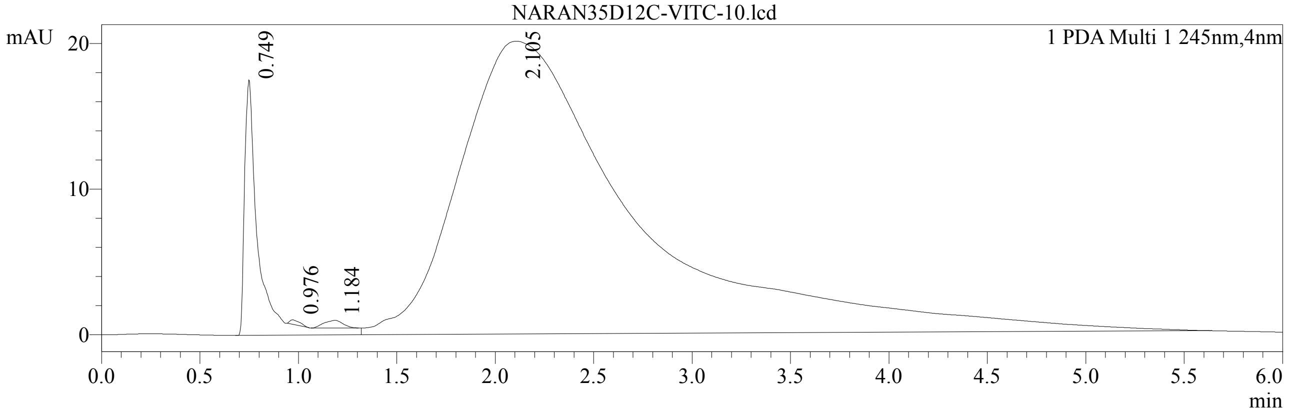
NARAN35D12C-VITC-10.lcd
mAU
1 PDA Multi 1 245nm,4nm
0.749
2.105
0.976
1.184
min

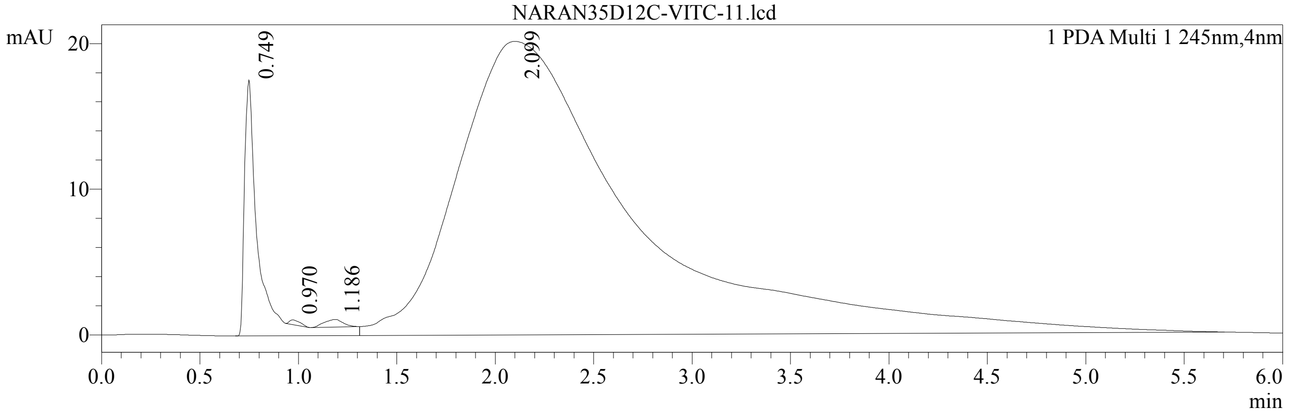
NARAN35D12C-VITC-11.lcd
mAU
1 PDA Multi 1 245nm,4nm
0.749
2.099
0.970
1.186
min

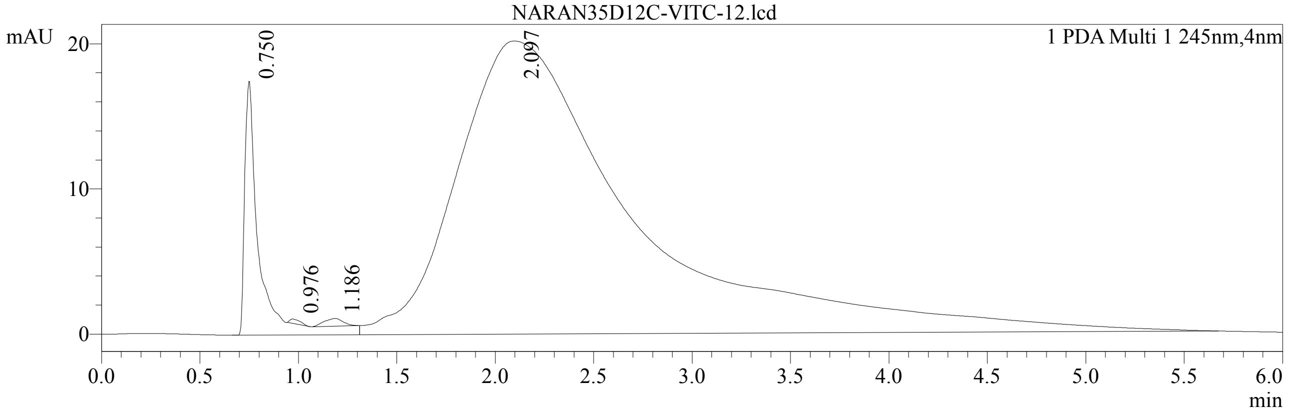
NARAN35D12C-VITC-12.lcd
mAU
1 PDA Multi 1 245nm,4nm
0.750
2.097
0.976
1.186
min

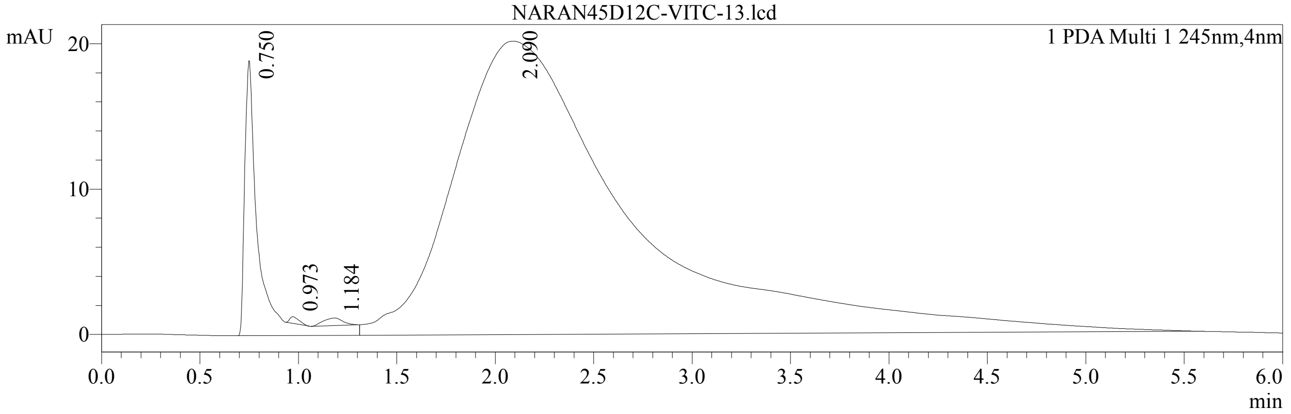
NARAN45D12C-VITC-13.lcd
mAU
1 PDA Multi 1 245nm,4nm
0.750
2.090
0.973
1.184
min

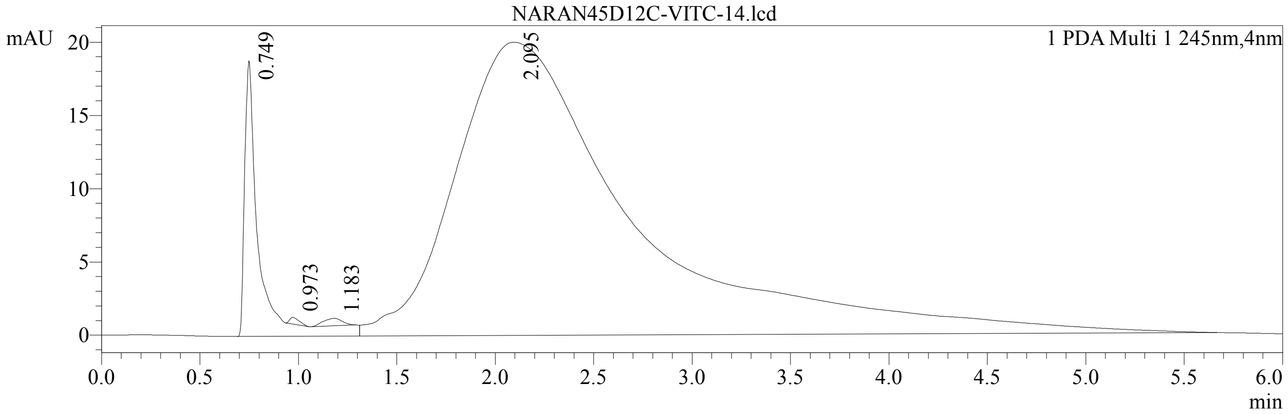
NARAN45D12C-VITC-14.lcd
mAU
1 PDA Multi 1 245nm,4nm
0.749
0.973
1.183
2.095
min

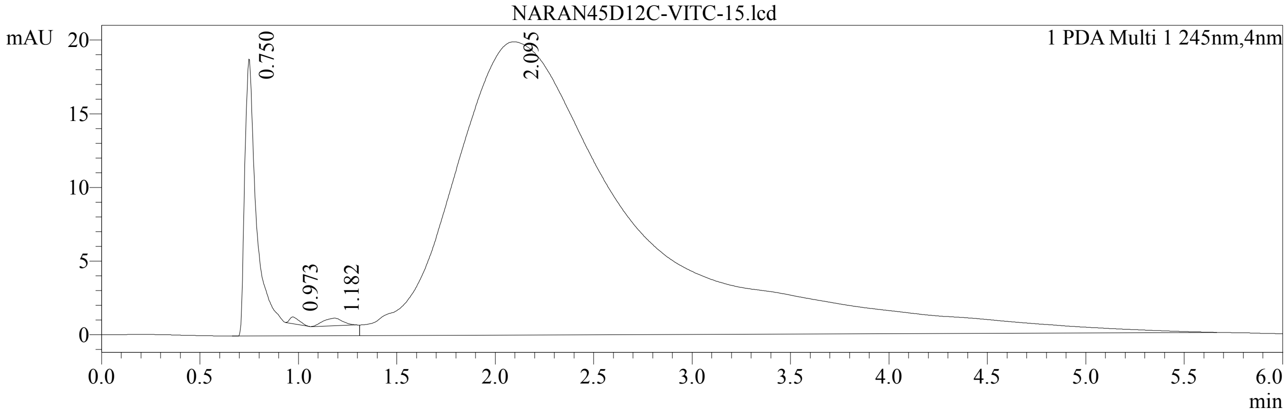
NARAN45D12C-VITC-15.lcd
mAU
1 PDA Multi 1 245nm,4nm
0.750
0.973
1.182
2.095
min

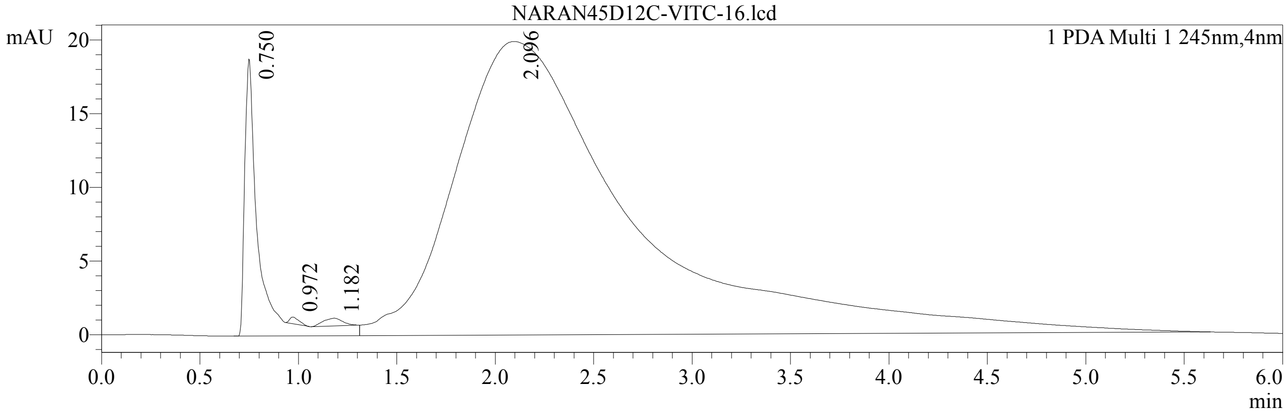
NARAN45D12C-VITC-16.lcd
mAU
1 PDA Multi 1 245nm,4nm
0.750
0.972
1.182
2.096
min

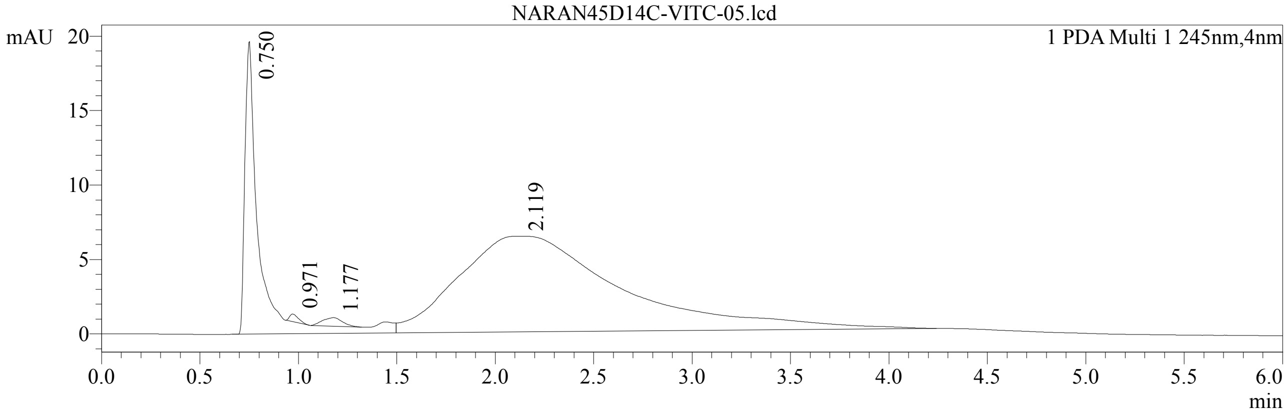
NARAN45D14C-VITC-05.lcd
mAU
1 PDA Multi 1 245nm,4nm
0.750
0.971
1.177
2.119
min

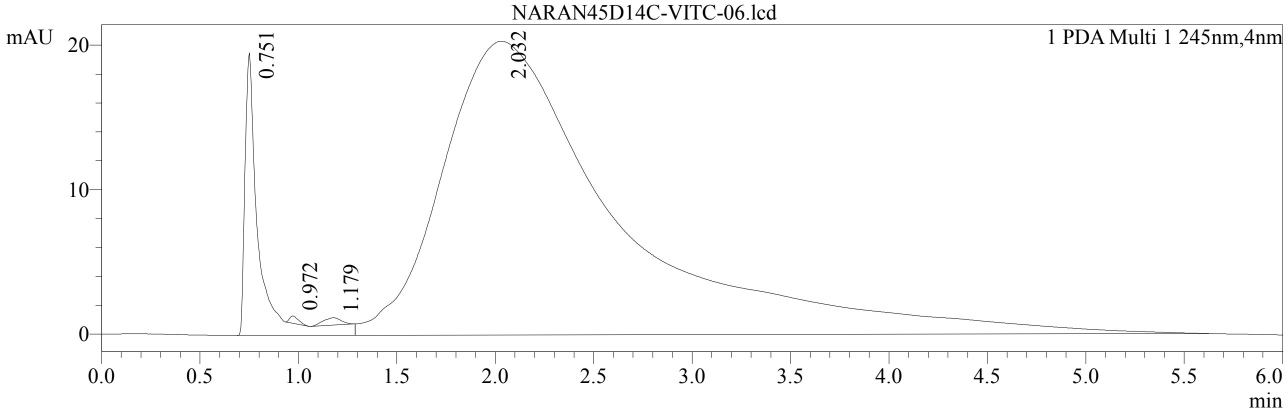
NARAN45D14C-VITC-06.lcd
mAU
1 PDA Multi 1 245nm,4nm
0.751
0.972
1.179
2.032
min

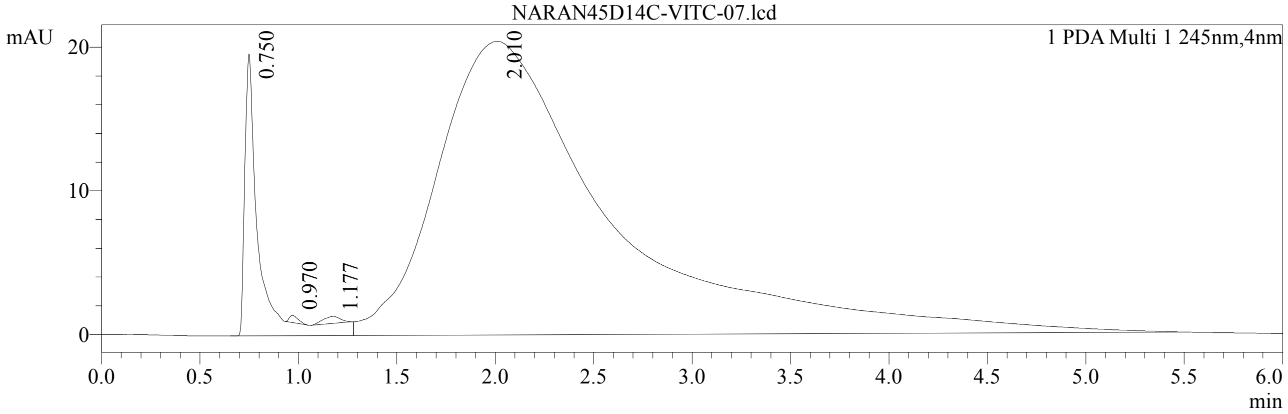
NARAN45D14C-VITC-07.lcd
mAU
1 PDA Multi 1 245nm,4nm
0.750
0.970
1.177
2.010
min

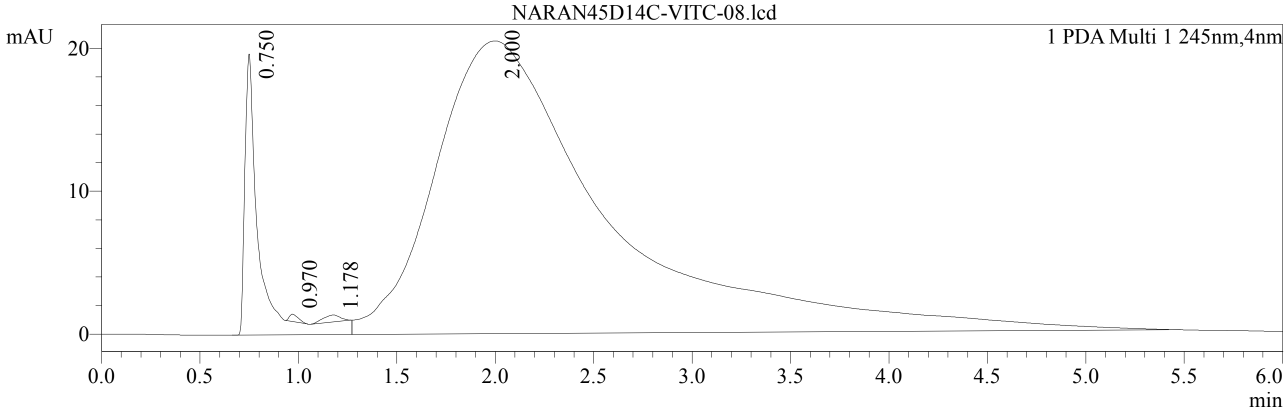
NARAN45D14C-VITC-08.lcd
mAU
1 PDA Multi 1 245nm,4nm
0.750
0.970
1.178
2.000
min

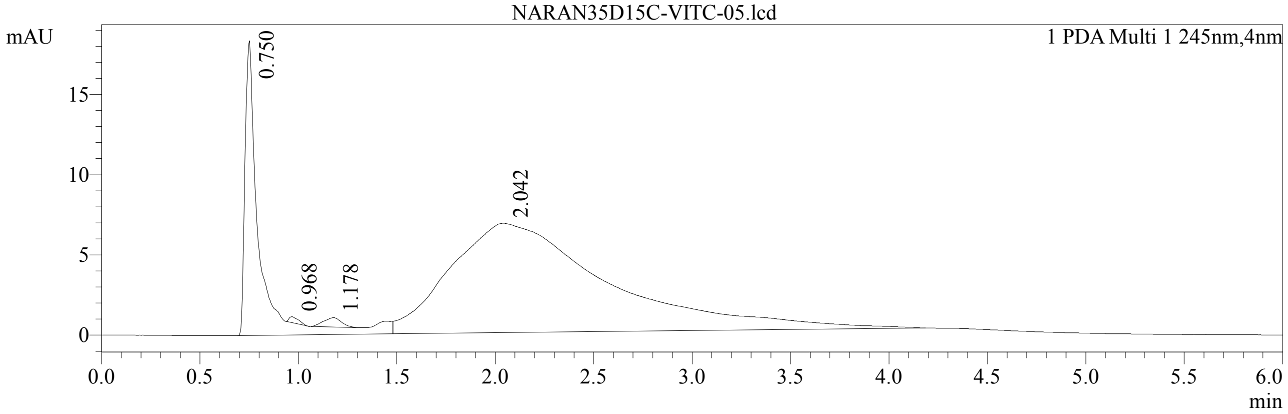
NARAN35D15C-VITC-05.lcd
mAU
1 PDA Multi 1 245nm,4nm
0.750
0.968
1.178
2.042
min

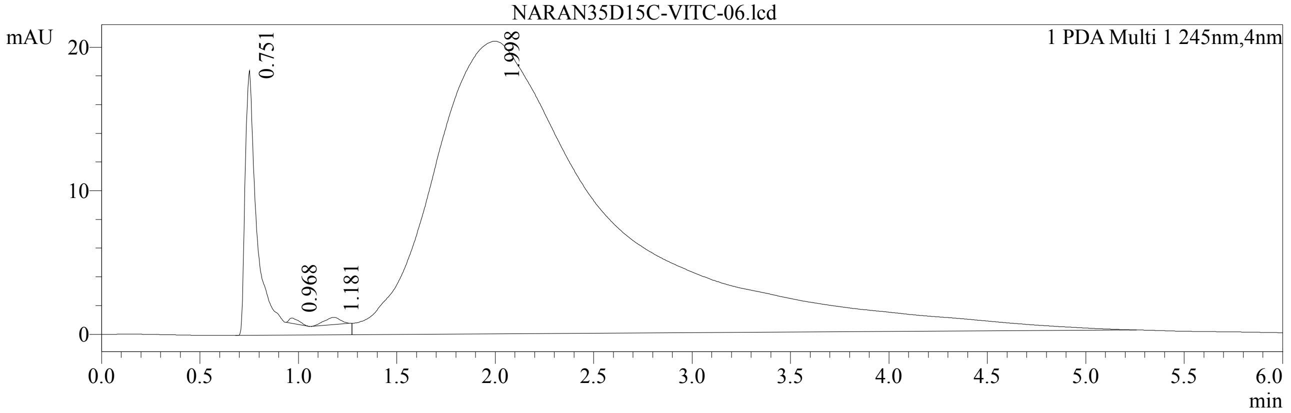
NARAN35D15C-VITC-06.lcd
mAU
1 PDA Multi 1 245nm,4nm
0.751
0.968
1.181
1.998
min

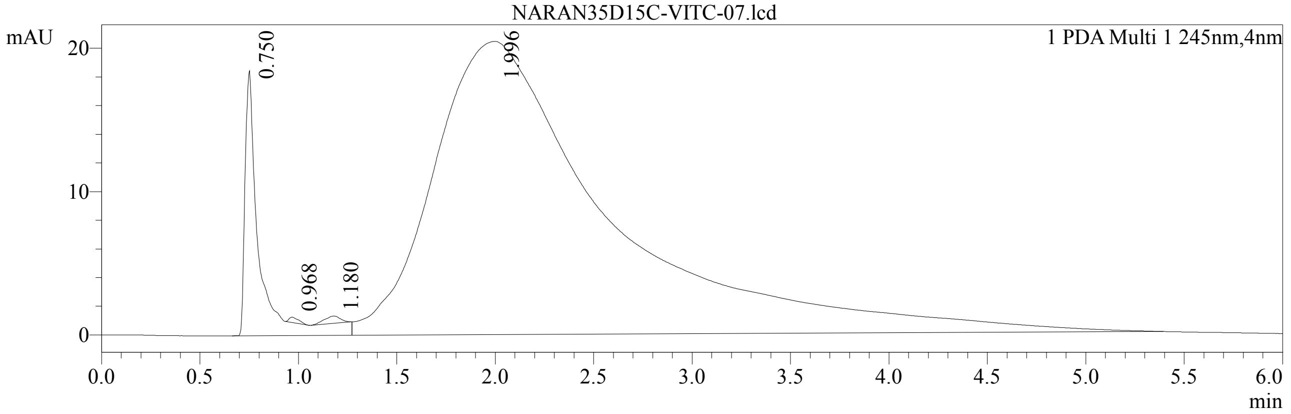
NARAN35D15C-VITC-07.lcd
mAU
1 PDA Multi 1 245nm,4nm
0.750
0.968
1.180
1.996
min

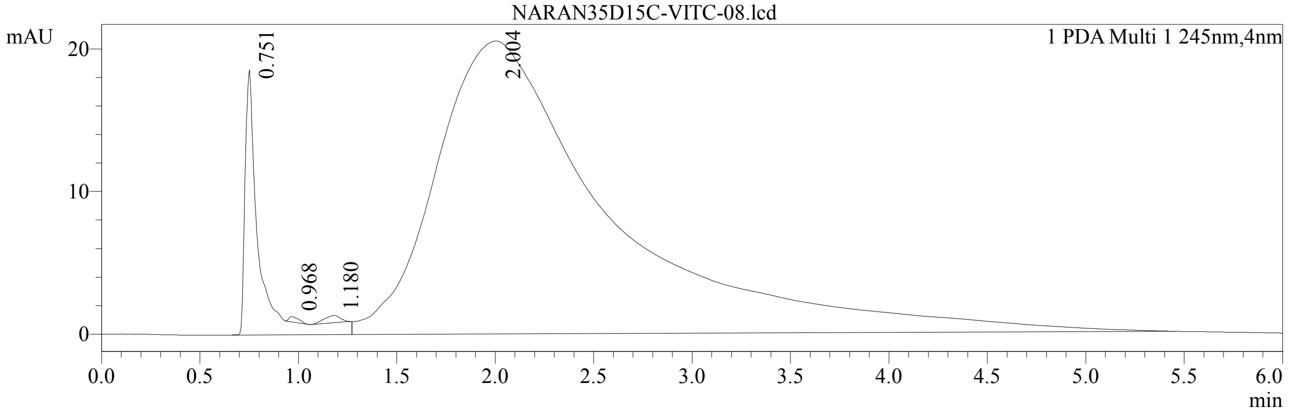
NARAN35D15C-VITC-08.lcd
mAU
1 PDA Multi 1 245nm,4nm
0.751
0.968
1.180
2.004
min

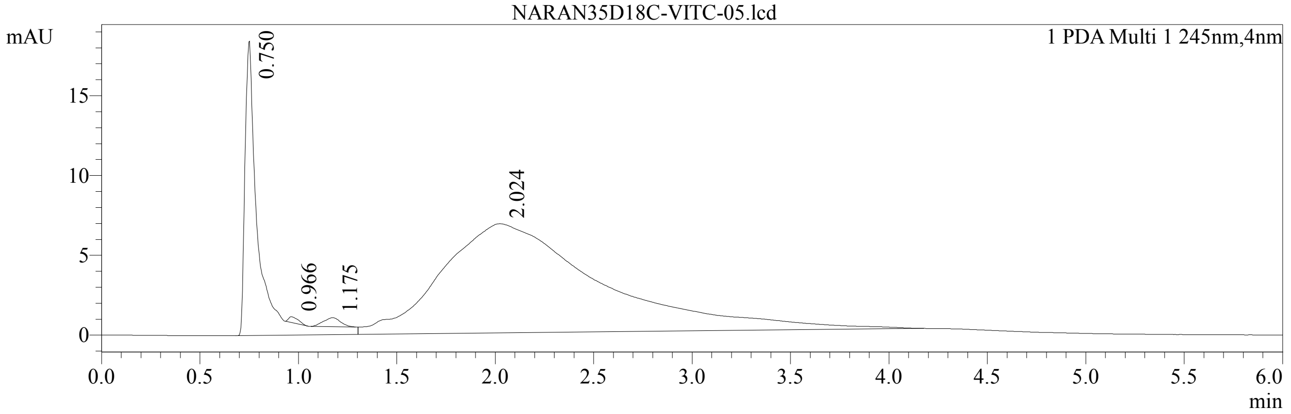
NARAN35D18C-VITC-05.lcd
mAU
1 PDA Multi 1 245nm,4nm
0.750
0.966
1.175
2.024
min

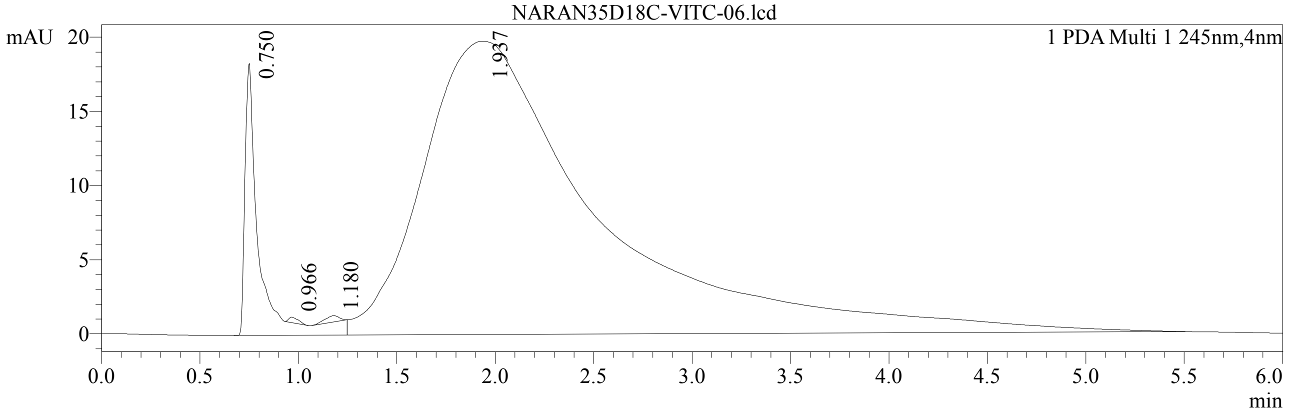
NARAN35D18C-VITC-06.lcd
mAU
1 PDA Multi 1 245nm,4nm
0.750
0.966
1.180
1.937
min

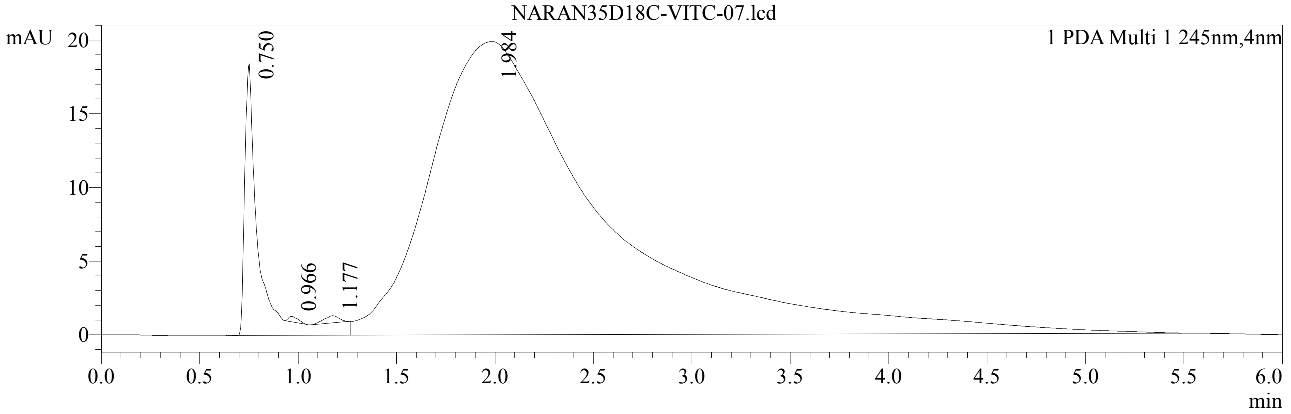
NARAN35D18C-VITC-07.lcd
mAU
1 PDA Multi 1 245nm,4nm
0.750
0.966
1.177
1.984
min

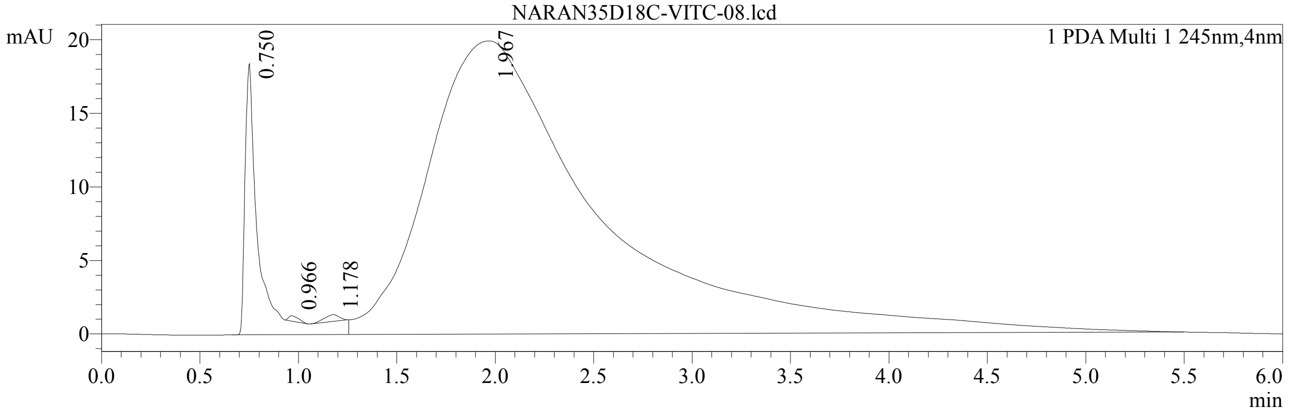
NARAN35D18C-VITC-08.lcd
mAU
1 PDA Multi 1 245nm,4nm
0.750
0.966
1.178
1.967
min

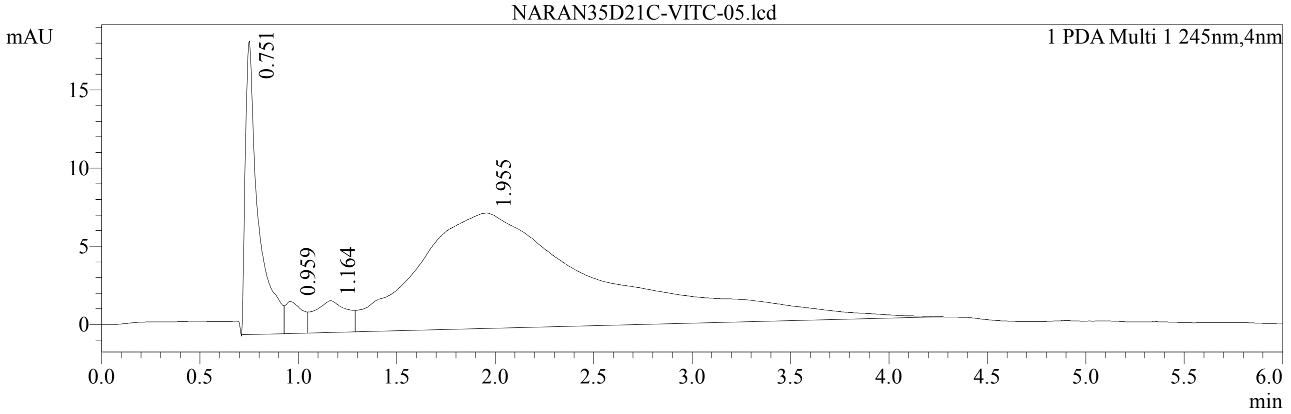
NARAN35D21C-VITC-05.lcd
mAU
1 PDA Multi 1 245nm,4nm
0.751
0.959
1.164
1.955
min

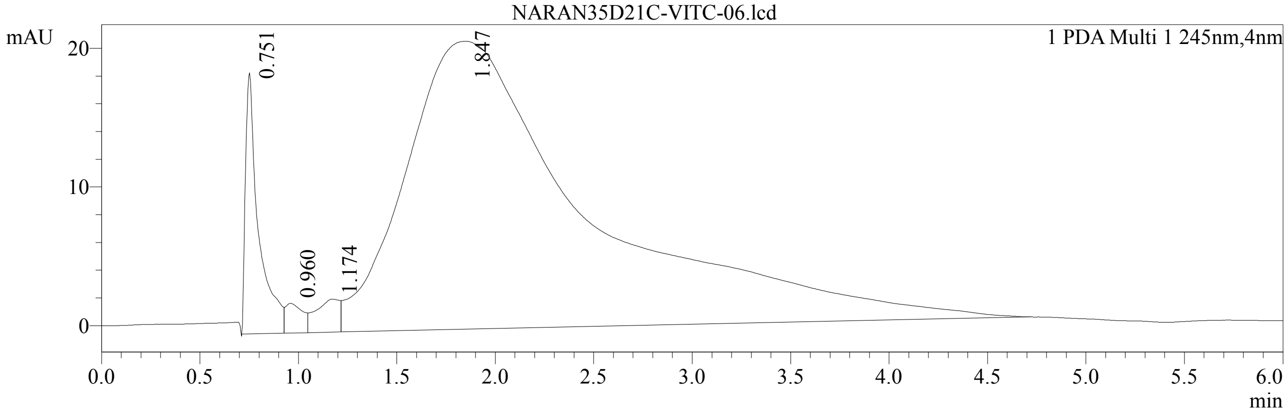

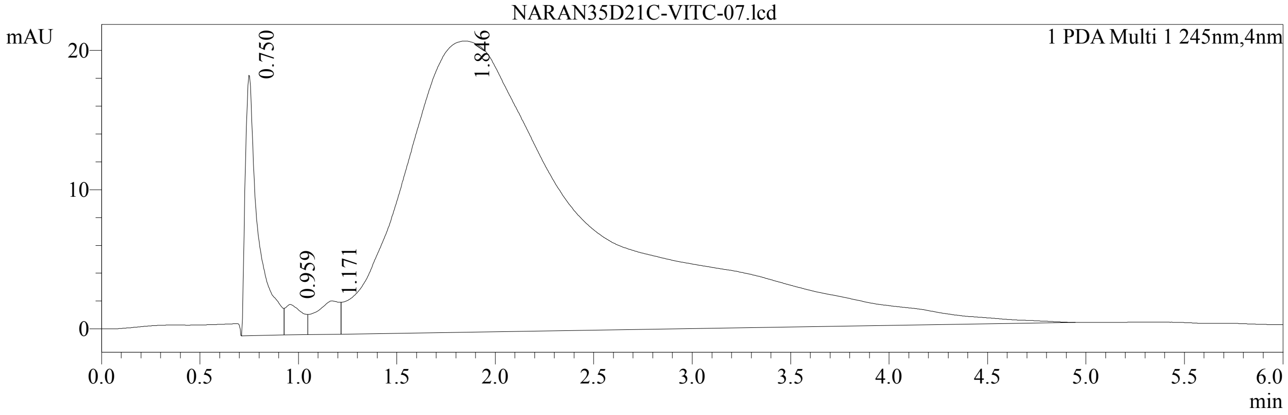

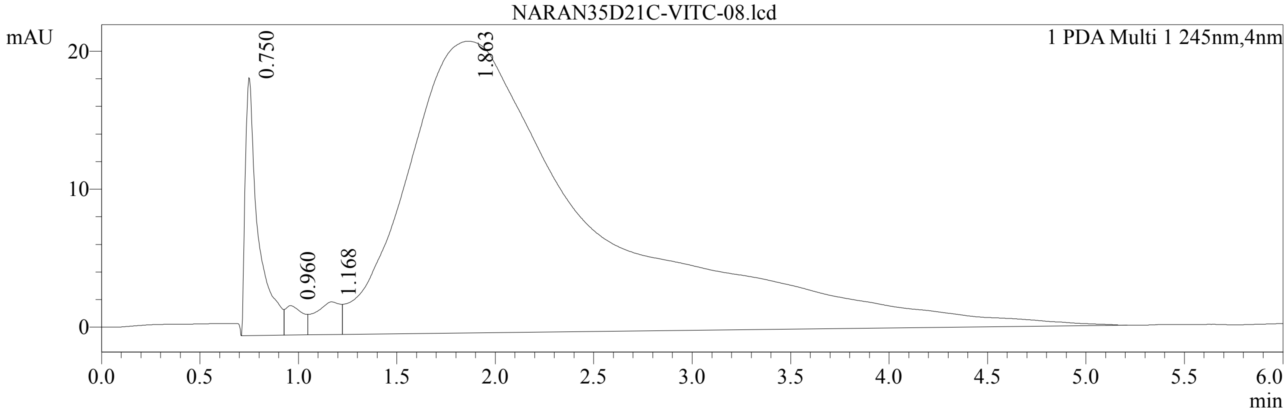

<< PDA >>

ID#1 Compound Name: VIT C

Title	Sample Name	Sample ID	Ret. Time	Area	Height	Conc.
NARAN55-D1D-VITC-07.lcd	PROT-VITC-07	NARAN55-D1D-VITC-07	0.754	125853	20537	50.077
NARAN55-D1D-VITC-08.lcd	PROT-VITC-08	NARAN55-D1D-VITC-08	0.755	139597	21567	55.546
NARAN55-D1D-VITC-09.lcd	PROT-VITC-09	NARAN55-D1D-VITC-09	0.749	134555	20666	53.539
NARAN45D2C-VITC-01.lcd	PROT-VITC-01	NARAN45D2C-VITC-01	0.750	104921	18456	41.748
NARAN45D2C-VITC-02.lcd	PROT-VITC-02	NARAN45D2C-VITC-02	0.754	102224	19016	40.675
NARAN45D2C-VITC-03.lcd	PROT-VITC-03	NARAN45D2C-VITC-03	0.750	138690	18882	55.185
NARAN45D2C-VITC-04.lcd	PROT-VITC-04	NARAN45D2C-VITC-04	0.750	97145	19038	38.654
NARAN55D2C-VITC-06.lcd	PROT-VITC-06	NARAN55D2C-VITC-06	0.750	105071	19604	41.807
NARAN55D2C-VITC-07.lcd	PROT-VITC-07	NARAN55D2C-VITC-07	0.751	109817	19927	43.696
NARAN55D2C-VITC-08.lcd	PROT-VITC-08	NARAN55D2C-VITC-08	0.750	139057	20737	55.330
NARAN55D2C-VITC-05.lcd	PROT-VITC-05	NARAN55D2C-VITC-05	0.750	124295	19101	49.457
NARAN35D3C-VITC-01.lcd	PROT-VITC-01	NARAN35D3C-VITC-01	0.751	113452	18924	45.142
NARAN35D3C-VITC-02.lcd	PROT-VITC-02	NARAN35D3C-VITC-02	0.751	91484	18072	36.401
NARAN35D3C-VITC-03.lcd	PROT-VITC-03	NARAN35D3C-VITC-03	0.750	75578	17319	30.073
NARAN35D3C-VITC-04.lcd	PROT-VITC-04	NARAN35D3C-VITC-04	0.750	78219	17265	31.123
NARAN55D3C-VITC-05.lcd	PROT-VITC-05	NARAN55D3C-VITC-05	0.751	91660	18527	36.471
NARAN55D3C-VITC-06.lcd	PROT-VITC-06	NARAN55D3C-VITC-06	0.751	99433	18700	39.564
NARAN55D3C-VITC-07.lcd	PROT-VITC-07	NARAN55D3C-VITC-07	0.751	95507	18539	38.002
NARAN55D3C-VITC-08.lcd	PROT-VITC-08	NARAN55D3C-VITC-08	0.751	97282	18593	38.708
NARAN45D4C-VITC-01.lcd	PROT-VITC-01	NARAN45D4C-VITC-01	0.750	86341	17629	34.355
NARAN45D4C-VITC-02.lcd	PROT-VITC-02	NARAN45D4C-VITC-02	0.750	89976	17615	35.801
NARAN45D4C-VITC-03.lcd	PROT-VITC-03	NARAN45D4C-VITC-03	0.750	87680	17633	34.888
NARAN45D4C-VITC-04.lcd	PROT-VITC-04	NARAN45D4C-VITC-04	0.750	87841	17600	34.952

Title	Sample Name	Sample ID	Ret. Time	Area	Height	Conc.
NARAN55D4C-VITC-05.lcd	PROT-VITC-05	NARAN55D4C-VITC-05	0.751	99815	19554	39.716
NARAN55D4C-VITC-06.lcd	PROT-VITC-06	NARAN55D4C-VITC-06	0.750	99884	19553	39.744
NARAN55D4C-VITC-07.lcd	PROT-VITC-07	NARAN55D4C-VITC-07	0.750	100601	19716	40.029
NARAN55D4C-VITC-08.lcd	PROT-VITC-08	NARAN55D4C-VITC-08	0.750	97546	19527	38.813
NARAN55D5C-VITC-05.lcd	PROT-VITC-05	NARAN55D5C-VITC-05	0.750	104911	19412	41.744
NARAN55D5C-VITC-06.lcd	PROT-VITC-06	NARAN55D5C-VITC-06	0.750	99327	19377	39.522
NARAN55D5C-VITC-07.lcd	PROT-VITC-07	NARAN55D5C-VITC-07	0.750	98623	19379	39.242
NARAN55D5C-VITC-08.lcd	PROT-VITC-08	NARAN55D5C-VITC-08	0.750	98689	19368	39.268
NARAN35D6C-VITC-01.lcd	PROT-VITC-01	NARAN35D6C-VITC-01	0.749	88599	17597	35.253
NARAN35D6C-VITC-02.lcd	PROT-VITC-02	NARAN35D6C-VITC-02	0.749	90341	17499	35.946
NARAN35D6C-VITC-03.lcd	PROT-VITC-03	NARAN35D6C-VITC-03	0.749	89433	17539	35.585
NARAN35D6C-VITC-04.lcd	PROT-VITC-04	NARAN35D6C-VITC-04	0.749	89978	17453	35.802
NARAN45D6C-VITC-05.lcd	PROT-VITC-05	NARAN45D6C-VITC-05	0.749	94202	18460	37.483
NARAN45D6C-VITC-06.lcd	PROT-VITC-06	NARAN45D6C-VITC-06	0.749	94759	18474	37.704
NARAN45D6C-VITC-07.lcd	PROT-VITC-07	NARAN45D6C-VITC-07	0.749	95323	18525	37.929
NARAN45D6C-VITC-08.lcd	PROT-VITC-08	NARAN45D6C-VITC-08	0.749	95503	18498	38.001
NARAN55D6C-VITC-09.lcd	PROT-VITC-09	NARAN55D6C-VITC-09	0.750	105345	20261	41.917
NARAN55D6C-VITC-10.lcd	PROT-VITC-10	NARAN55D6C-VITC-10	0.750	106762	20266	42.481
NARAN55D6C-VITC-11.lcd	PROT-VITC-11	NARAN55D6C-VITC-11	0.750	105839	20348	42.113
NARAN55D6C-VITC-12.lcd	PROT-VITC-12	NARAN55D6C-VITC-12	0.750	106017	20324	42.184
NARAN55D7C-VITC-09.lcd	PROT-VITC-09	NARAN55D7C-VITC-09	0.751	106855	20585	42.517
NARAN55D7C-VITC-10.lcd	PROT-VITC-10	NARAN55D7C-VITC-10	0.750	106735	20559	42.470
NARAN55D7C-VITC-11.lcd	PROT-VITC-11	NARAN55D7C-VITC-11	0.750	107452	20620	42.755
NARAN55D7C-VITC-12.lcd	PROT-VITC-12	NARAN55D7C-VITC-12	0.750	108420	20693	43.140
NARAN45D8C-VITC-05.lcd	PROT-VITC-09	NARAN45D7C-VITC-05	0.750	93567	18197	37.230
NARAN45D8C-VITC-06.lcd	PROT-VITC-10	NARAN45D7C-VITC-06	0.749	94319	18176	37.529
NARAN45D8C-VITC-07.lcd	PROT-VITC-11	NARAN45D7C-VITC-07	0.750	95726	18189	38.089
NARAN45D8C-VITC-08.lcd	PROT-VITC-12	NARAN45D7C-VITC-08	0.750	95153	18188	37.861
NARAN35D9C-VITC-05.lcd	PROT-VITC-05	NARAN35D9C-VITC-05	0.749	89642	17367	35.668
NARAN35D9C-VITC-06.lcd	PROT-VITC-06	NARAN35D9C-VITC-06	0.749	90931	17360	36.181
NARAN35D9C-VITC-07.lcd	PROT-VITC-07	NARAN35D9C-VITC-07	0.749	92882	17402	36.958
NARAN35D9C-VITC-08.lcd	PROT-VITC-08	NARAN35D9C-VITC-08	0.748	93675	17561	37.273
NARAN45D10C-VITC-05.lcd	PROT-VITC-05	NARAN45D10C-VITC-05	0.752	95227	18179	37.891
NARAN45D10C-VITC-06.lcd	PROT-VITC-06	NARAN45D10C-VITC-06	0.749	96337	18280	38.332
NARAN45D10C-VITC-07.lcd	PROT-VITC-07	NARAN45D10C-VITC-07	0.750	99288	18270	39.507
NARAN45D10C-VITC-08.lcd	PROT-VITC-08	NARAN45D10C-VITC-08	0.750	99929	18216	39.761
NARAN35D12C-VITC-09.lcd	PROT-VITC-09	NARAN35D12C-VITC-09	0.749	93187	17662	37.079
NARAN35D12C-VITC-10.lcd	PROT-VITC-10	NARAN35D12C-VITC-10	0.749	90470	17488	35.998
NARAN35D12C-VITC-11.lcd	PROT-VITC-11	NARAN35D12C-VITC-11	0.749	92408	17473	36.769
NARAN35D12C-VITC-12.lcd	PROT-VITC-12	NARAN35D12C-VITC-12	0.750	92574	17492	36.835
NARAN45D12C-VITC-13.lcd	PROT-VITC-13	NARAN45D12C-VITC-13	0.750	98313	18889	39.119
NARAN45D12C-VITC-14.lcd	PROT-VITC-14	NARAN45D12C-VITC-14	0.749	98397	18719	39.152
NARAN45D12C-VITC-15.lcd	PROT-VITC-15	NARAN45D12C-VITC-15	0.750	98119	18760	39.041
NARAN45D12C-VITC-16.lcd	PROT-VITC-16	NARAN45D12C-VITC-16	0.750	97772	18732	38.903
NARAN45D14C-VITC-05.lcd	PROT-VITC-05	NARAN45D14C-VITC-05	0.750	102361	19625	40.729
NARAN45D14C-VITC-06.lcd	PROT-VITC-06	NARAN45D14C-VITC-06	0.751	99057	19452	39.415
NARAN45D14C-VITC-07.lcd	PROT-VITC-07	NARAN45D14C-VITC-07	0.750	101665	19557	40.452
NARAN45D14C-VITC-08.lcd	PROT-VITC-08	NARAN45D14C-VITC-08	0.750	102303	19651	40.706
NARAN35D15C-VITC-05.lcd	PROT-VITC-05	NARAN35D15C-VITC-05	0.750	96020	18279	38.206
NARAN35D15C-VITC-06.lcd	PROT-VITC-06	NARAN35D15C-VITC-06	0.751	93140	18317	37.060
NARAN35D15C-VITC-07.lcd	PROT-VITC-07	NARAN35D15C-VITC-07	0.750	96145	18392	38.256
NARAN35D15C-VITC-08.lcd	PROT-VITC-08	NARAN35D15C-VITC-08	0.751	95830	18423	38.131
NARAN35D18C-VITC-05.lcd	PROT-VITC-05	NARAN35D18C-VITC-05	0.750	90204	18420	35.892
NARAN35D18C-VITC-06.lcd	PROT-VITC-06	NARAN35D18C-VITC-06	0.750	93121	18299	37.053
NARAN35D18C-VITC-07.lcd	PROT-VITC-07	NARAN35D18C-VITC-07	0.750	94987	18359	37.795
NARAN35D18C-VITC-08.lcd	PROT-VITC-08	NARAN35D18C-VITC-08	0.750	95162	18370	37.865
NARAN35D21C-VITC-05.lcd	PROT-VITC-05	NARAN35D21C-VITC-05	0.751	87048	18737	34.636
NARAN35D21C-VITC-06.lcd	PROT-VITC-06	NARAN35D21C-VITC-06	0.751	87594	18717	34.853
NARAN35D21C-VITC-07.lcd	PROT-VITC-07	NARAN35D21C-VITC-07	0.750	88089	18682	35.051
NARAN35D21C-VITC-08.lcd	PROT-VITC-08	NARAN35D21C-VITC-08	0.750	87510	18632	34.820
Average			0.750	98973	18772	39.381
%RSD			0.144	12.057	5.419	12.057
Maximum			0.755	139597	21567	55.546
Minimum			0.748	75578	17265	30.073
Standard Deviation			0.001	11933	1017	4.748

ID# : 1
Name : VIT C
Quantitative Method : External Standard
Function : f(x)=2513.20*x+0
Rr1=0.9983722 Rr2=0.9967470 RSS=1.779444e+010
MeanRF: 2.712798e+003 RFSD: 4.273329e+002 RFRSD: 15.752473
FitType : Linear
ZeroThrough : Through
Weighted Regression : None
Detector Name : PDA

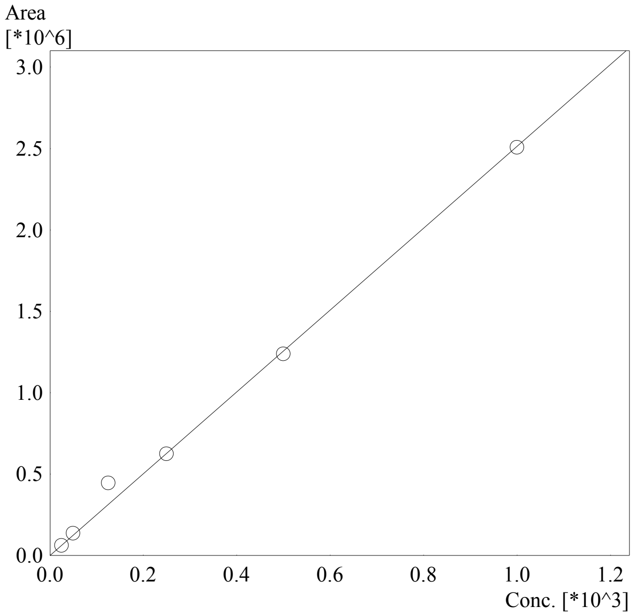

#	Conc.(Ratio)	MeanArea	Area
1	25	62703	62703
2	50	136316	136316
3	125	445542	445542
4	250	623948	623948
5	500	1237707	1237707
6	1000	2506784	2506784

Summary(Conc.)

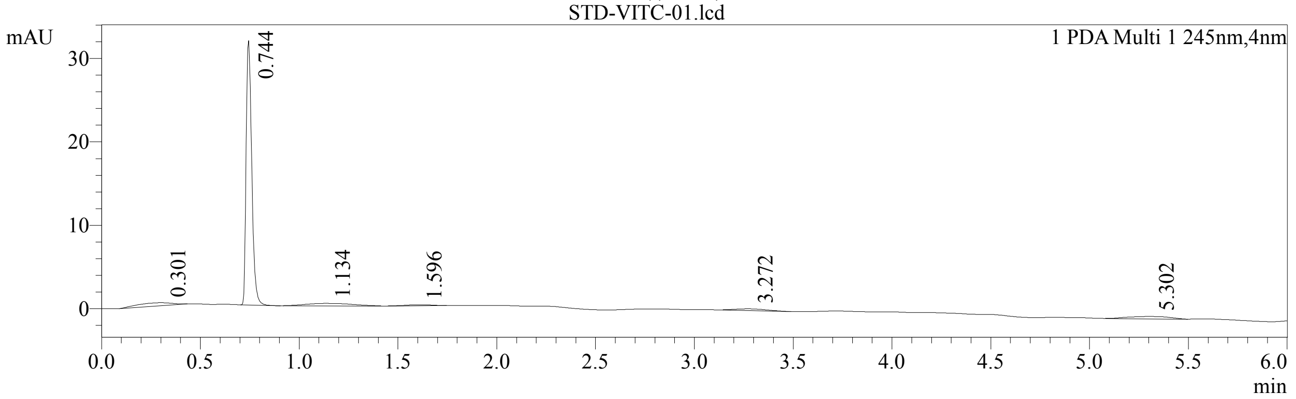

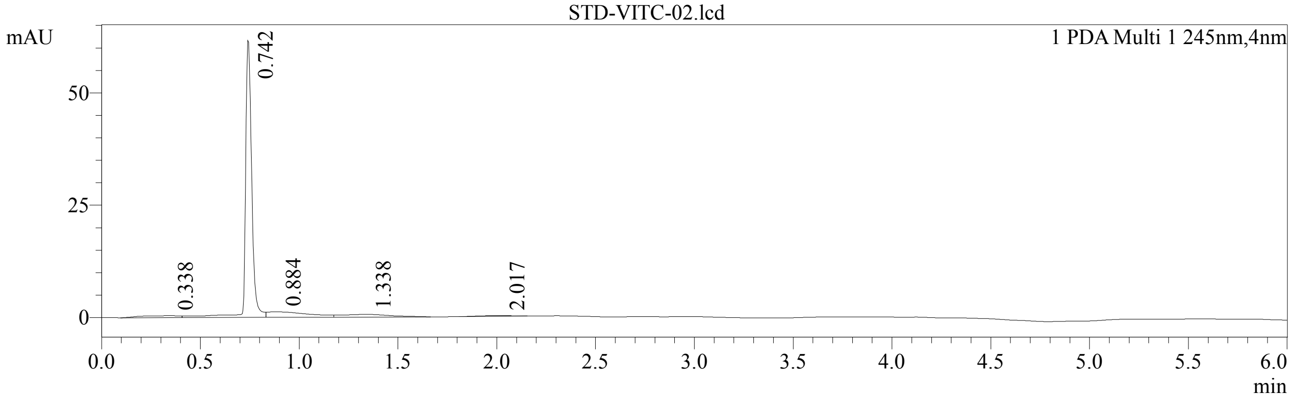

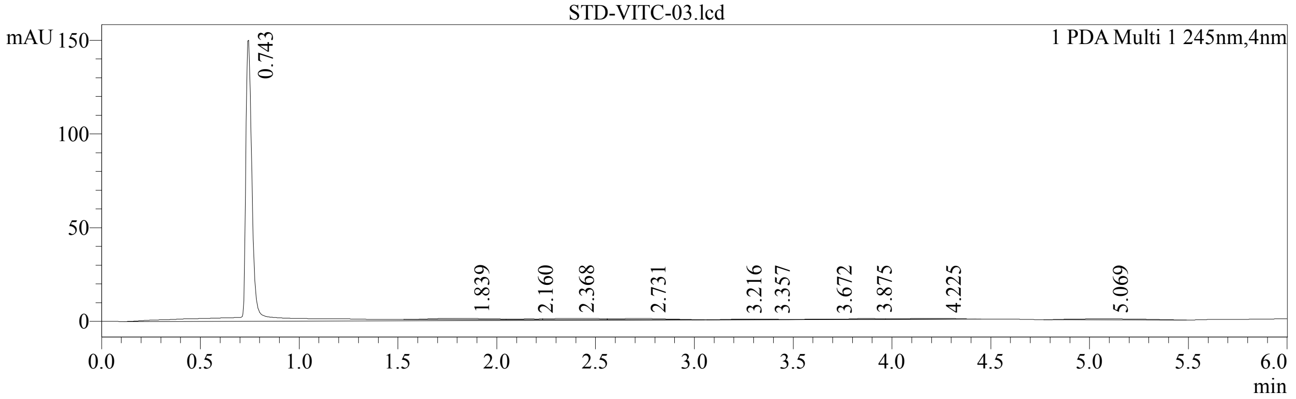

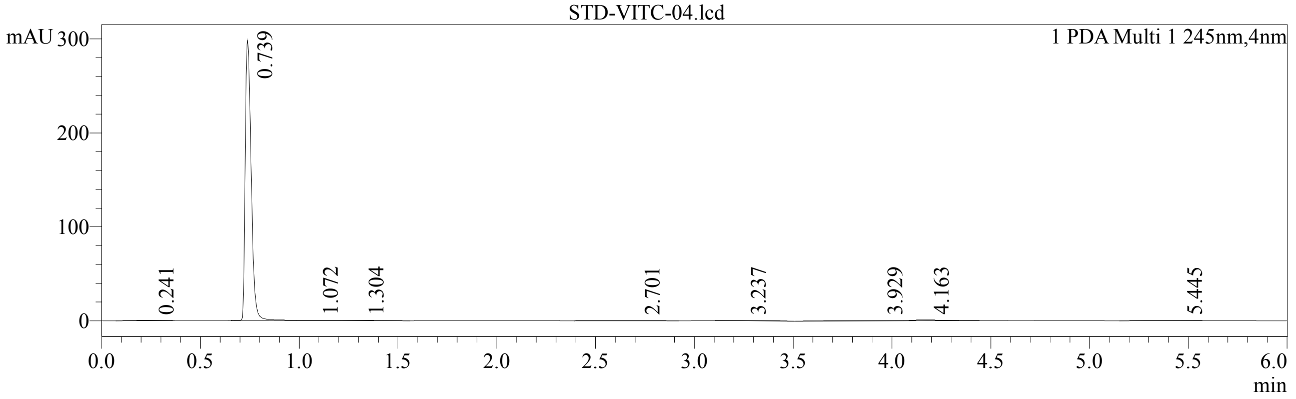

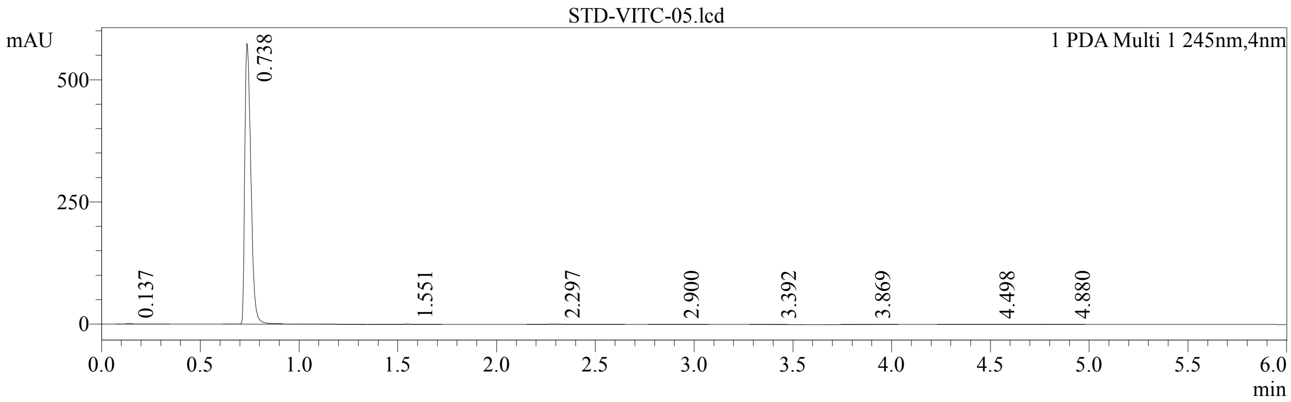

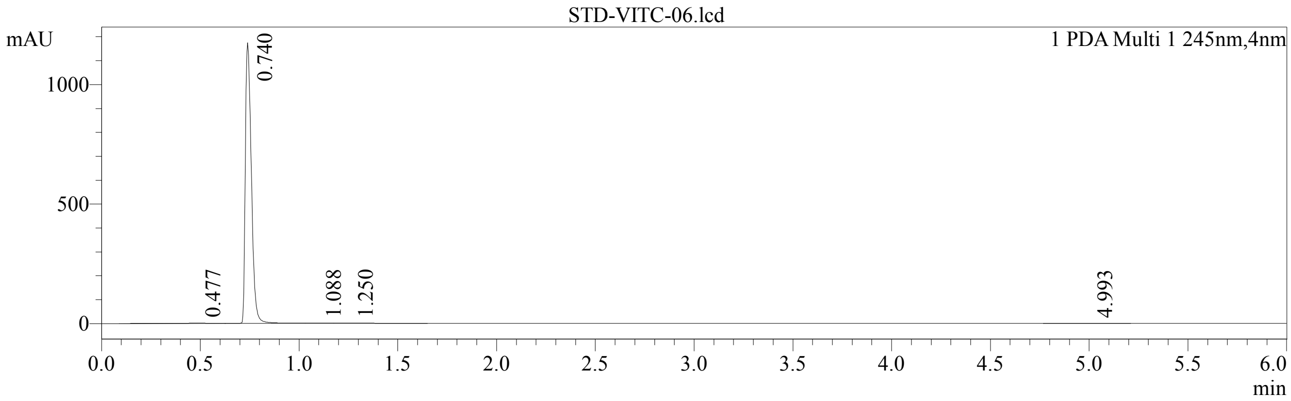

<< PDA >>

Title	Sample Name	Sample ID	VIT C
STD-VITC-01.lcd	STD-VITC-01	STD-VITC-01	24.950
STD-VITC-02.lcd	STD-VITC-02	STD-VITC-02	54.240
STD-VITC-03.lcd	STD-VITC-03	STD-VITC-03	177.281
STD-VITC-04.lcd	STD-VITC-04	STD-VITC-04	248.268
STD-VITC-05.lcd	STD-VITC-05	STD-VITC-05	492.482
STD-VITC-06.lcd	STD-VITC-06	STD-VITC-06	997.446
Average			332.444
%RSD			110.190
Maximum			997.446
Minimum			24.950
Standard Deviation			366.320

Summary(Conc.)
NARAN55-D1D-VITC-07.lcd

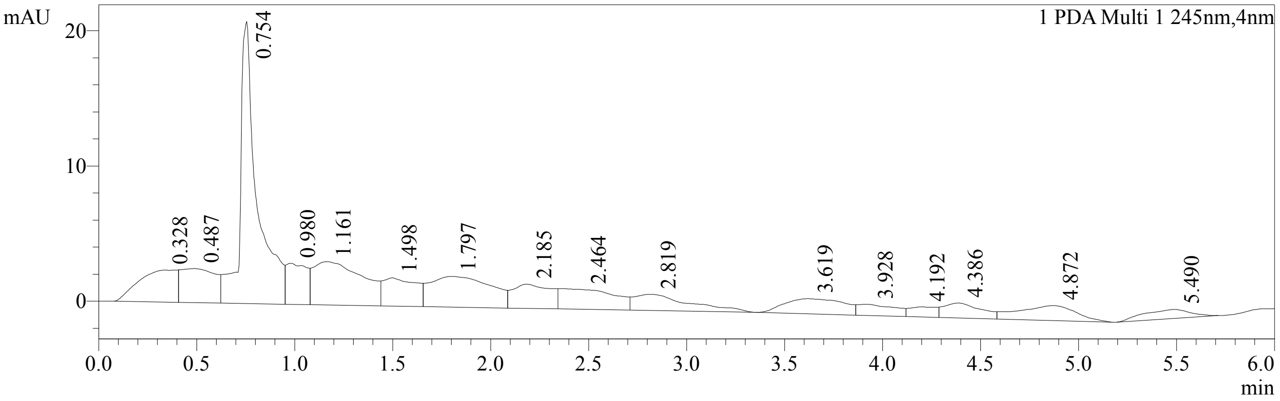

NARAN55-D1D-VITC-08.lcd

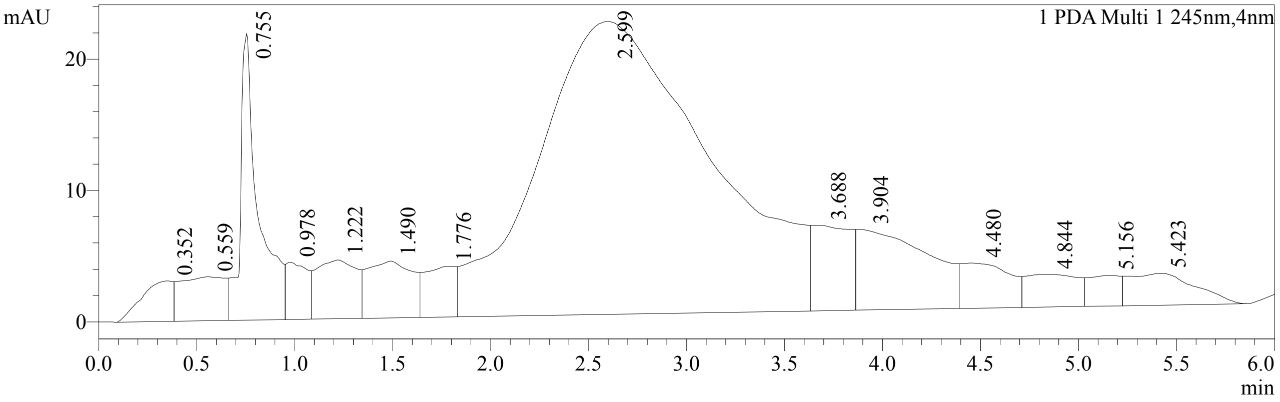

NARAN55-D1D-VITC-09.lcd

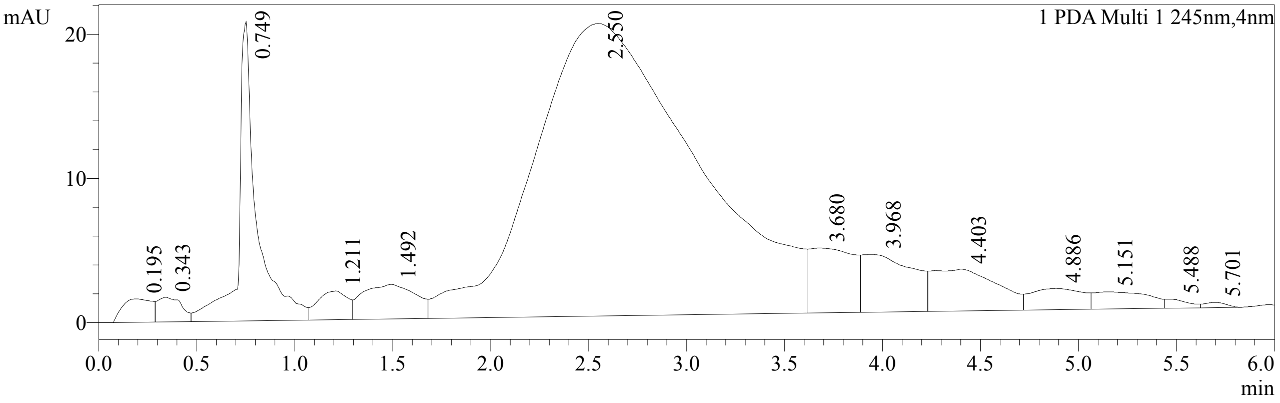

NARAN45D2C-VITC-01.lcd

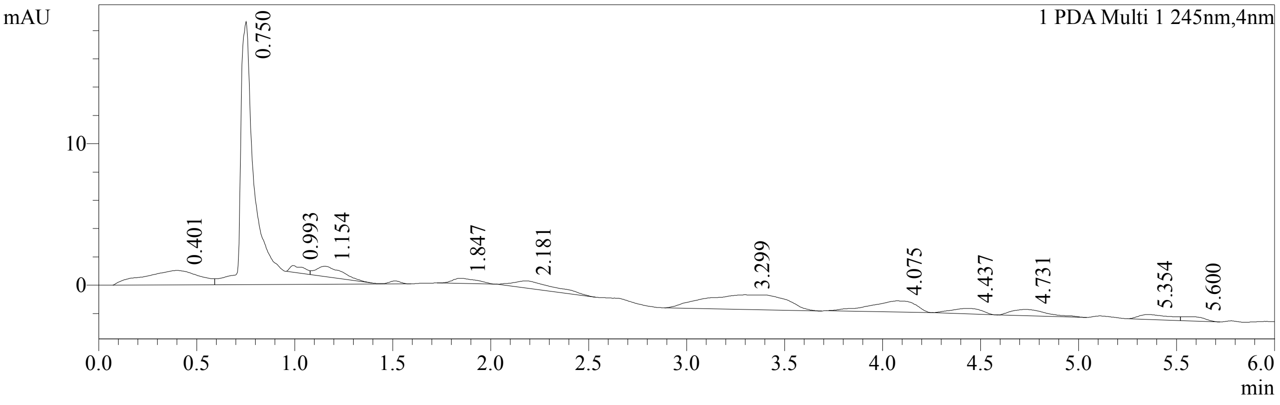

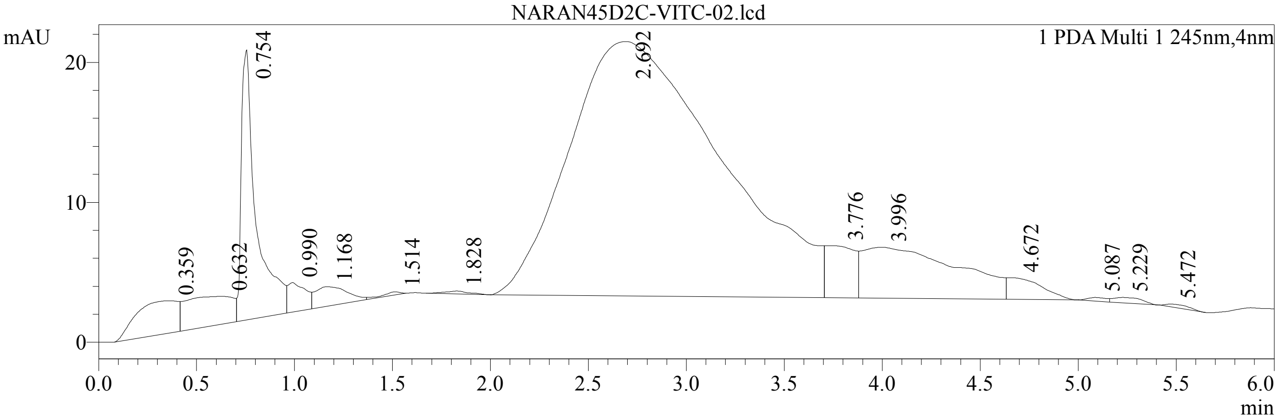
NARAN45D2C-VITC-02.lcd
mAU
1 PDA Multi 1 245nm,4nm
0.359
0.632
0.754
0.990
1.168
1.514
1.828
2.692
3.776
3.996
4.672
5.087
5.229
5.472
min

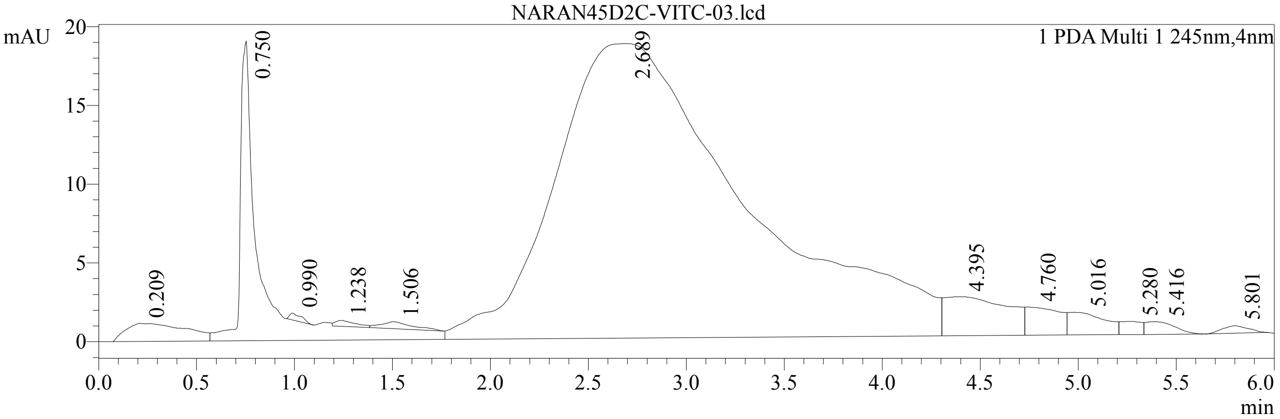
NARAN45D2C-VITC-03.lcd
mAU
1 PDA Multi 1 245nm,4nm
0.209
0.750
0.990
1.238
1.506
2.689
4.395
4.760
5.016
5.280
5.416
5.801
min

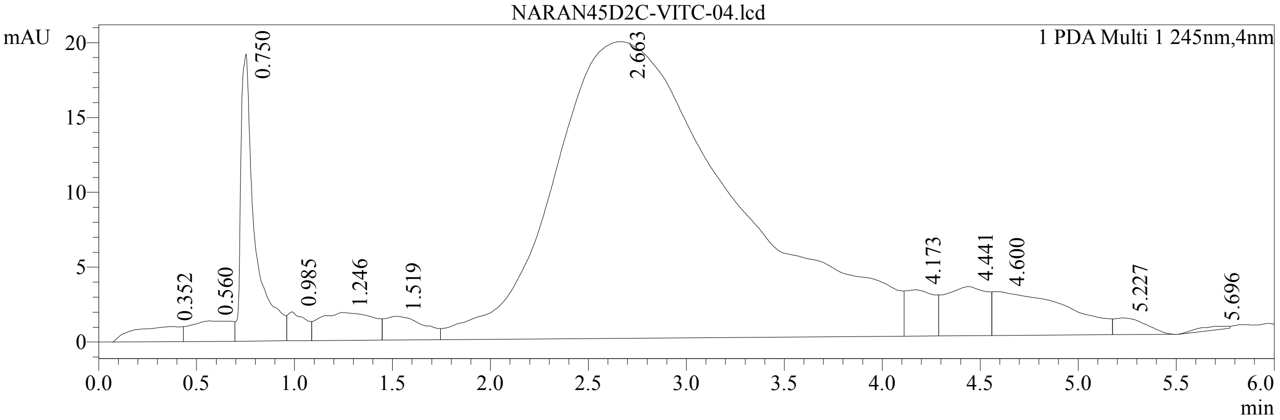
NARAN45D2C-VITC-04.lcd
mAU
1 PDA Multi 1 245nm,4nm
0.352
0.560
0.750
0.985
1.246
1.519
2.663
4.173
4.441
4.600
5.227
5.696
min

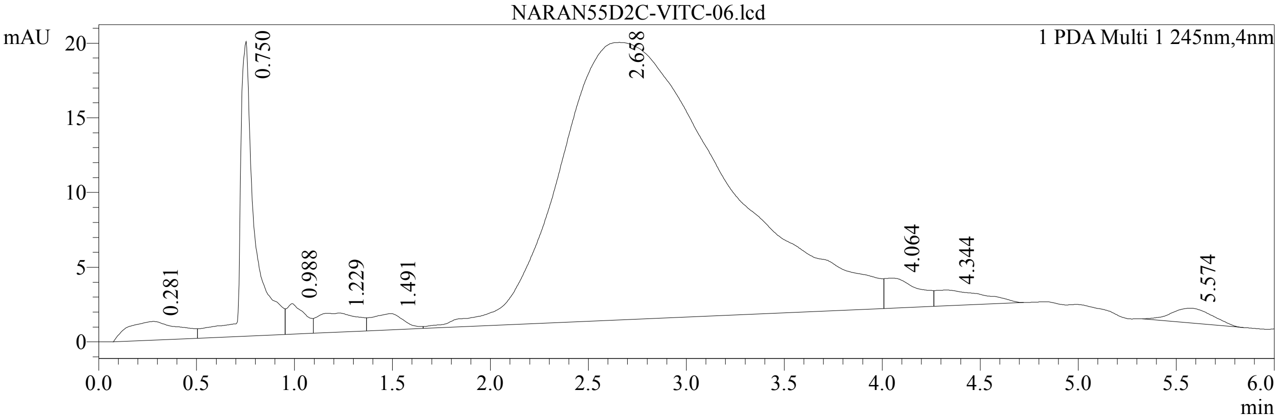
NARAN55D2C-VITC-06.lcd
mAU
1 PDA Multi 1 245nm,4nm
0.281
0.750
0.988
1.229
1.491
2.658
4.064
4.344
5.574
min

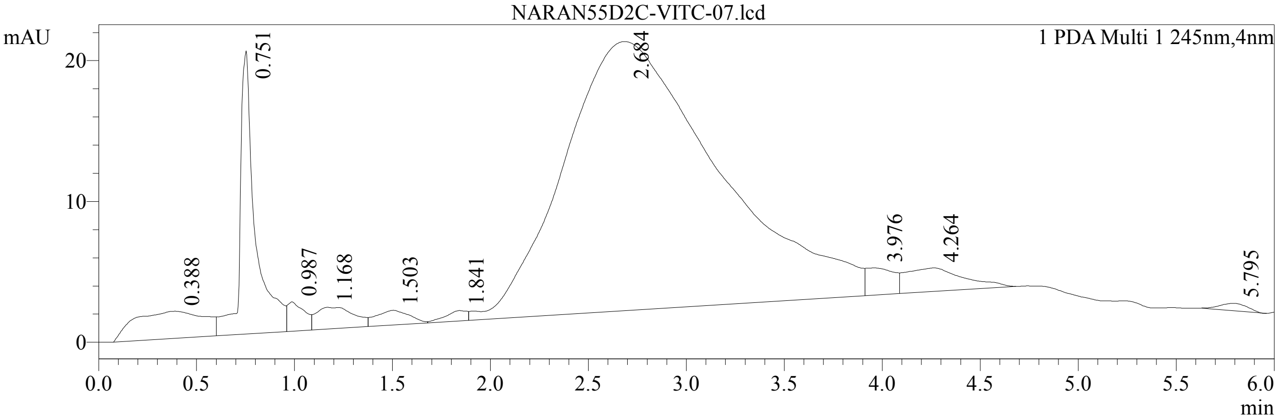
NARAN55D2C-VITC-07.lcd
mAU
1 PDA Multi 1 245nm,4nm
0.388
0.751
0.987
1.168
1.503
1.841
2.684
3.976
4.264
5.795
min

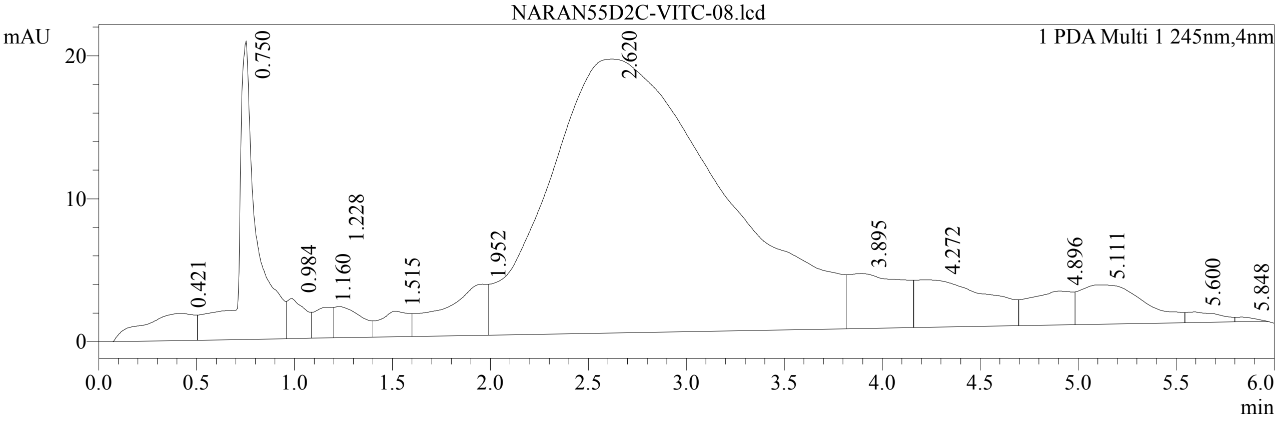
NARAN55D2C-VITC-08.lcd
mAU
1 PDA Multi 1 245nm,4nm
0.421
0.750
0.984
1.160
1.228
1.515
1.952
2.620
3.895
4.272
4.896
5.111
5.600
5.848
min

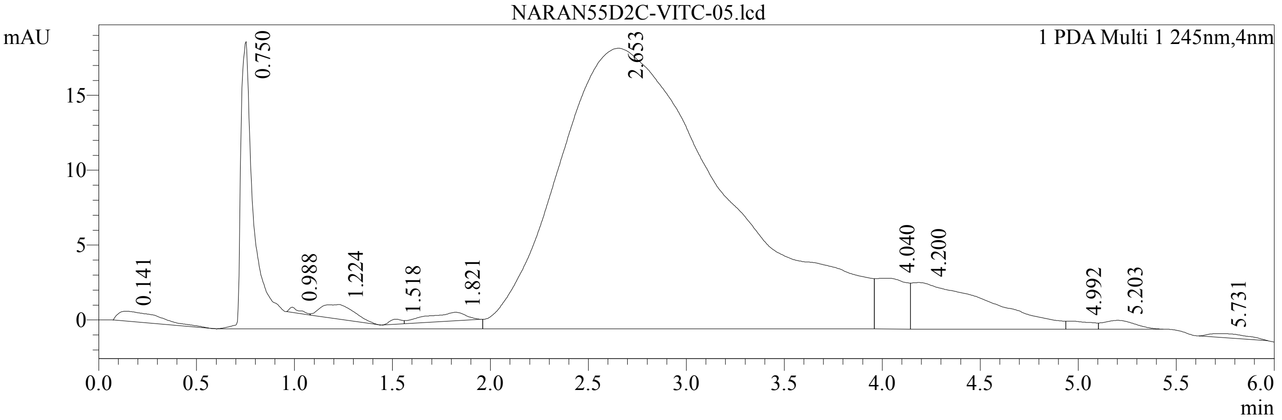
NARAN55D2C-VITC-05.lcd
mAU
1 PDA Multi 1 245nm,4nm
0.141
0.750
0.988
1.224
1.518
1.821
2.653
4.040
4.200
4.992
5.203
5.731
min

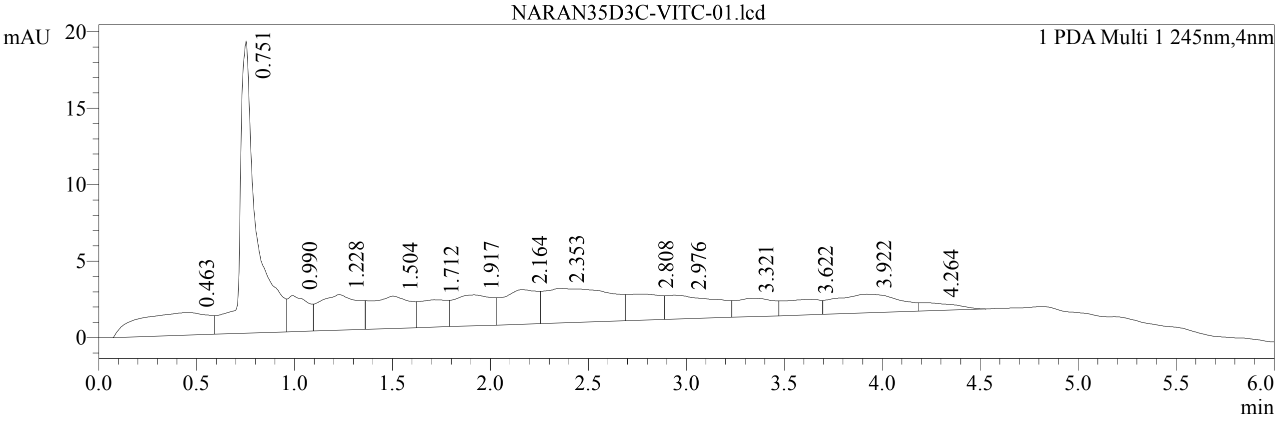
NARAN35D3C-VITC-01.lcd
mAU
1 PDA Multi 1 245nm,4nm
0.463
0.751
0.990
1.228
1.504
1.712
1.917
2.164
2.353
2.808
2.976
3.321
3.622
3.922
4.264
min

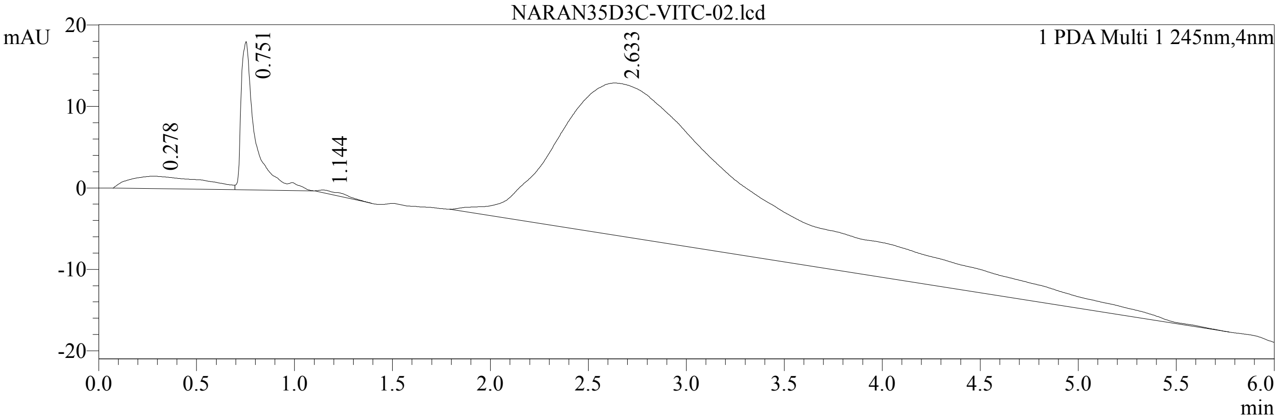
NARAN35D3C-VITC-02.lcd
mAU
1 PDA Multi 1 245nm,4nm
0.278
0.751
1.144
2.633
min

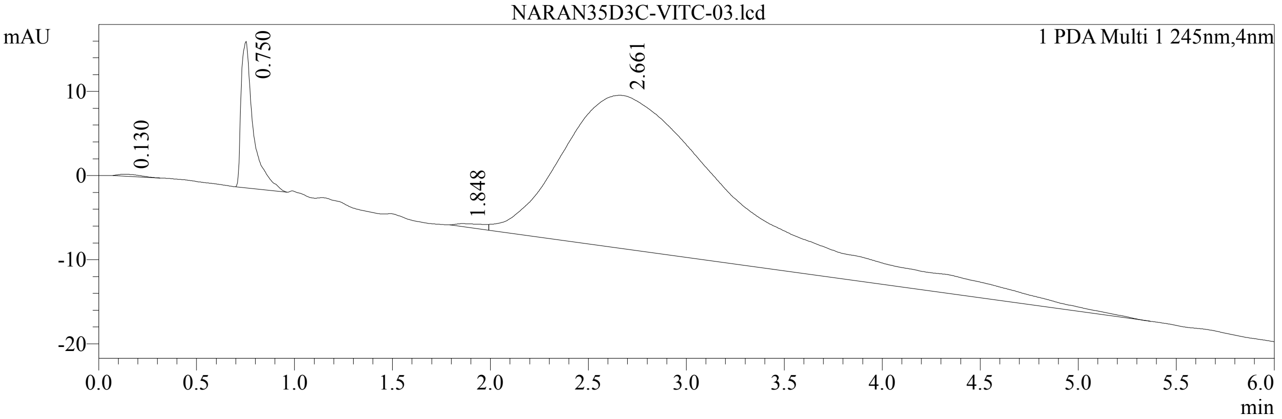
NARAN35D3C-VITC-03.lcd
mAU
1 PDA Multi 1 245nm,4nm
0.130
0.750
1.848
2.661
min

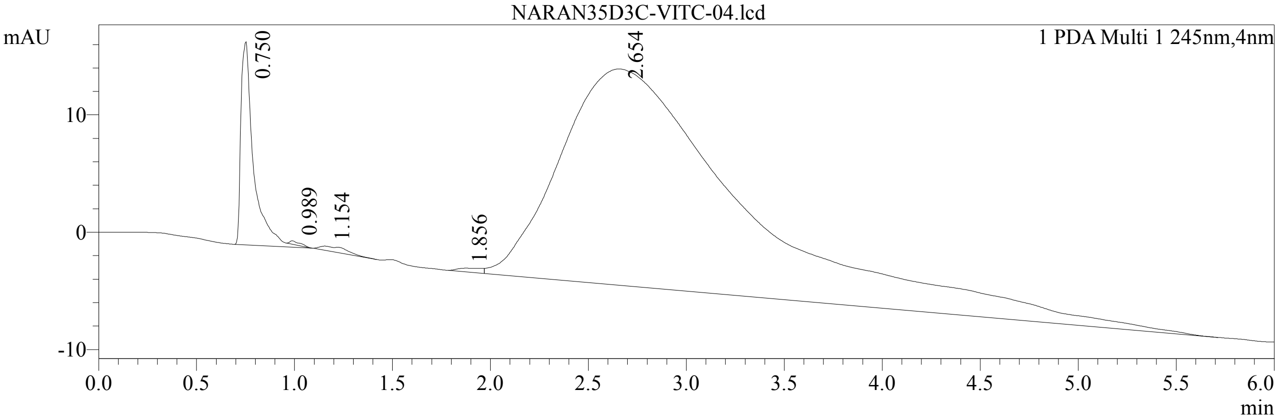
NARAN35D3C-VITC-04.lcd
mAU
1 PDA Multi 1 245nm,4nm
0.750
0.989
1.154
1.856
2.654
min

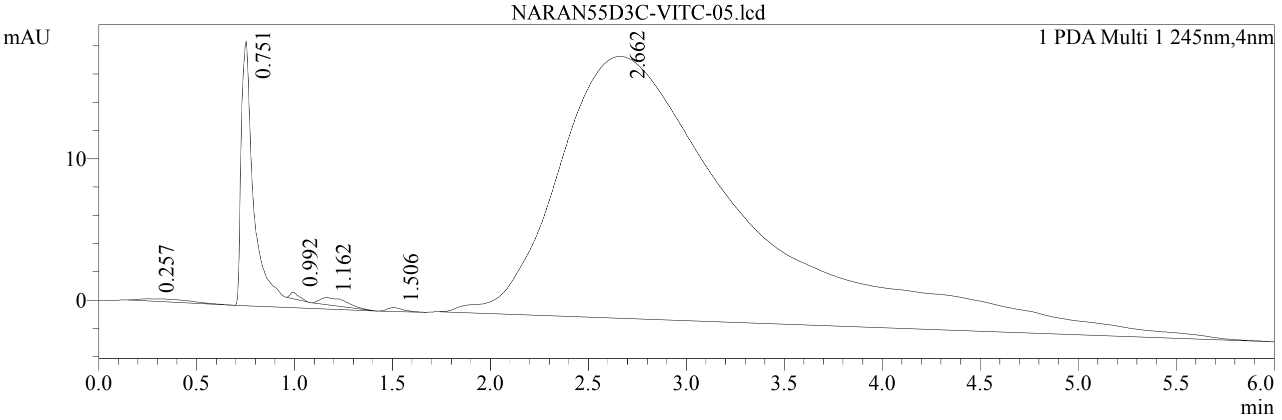
NARAN55D3C-VITC-05.lcd
mAU
1 PDA Multi 1 245nm,4nm
0.257
0.751
0.992
1.162
1.506
2.662
min

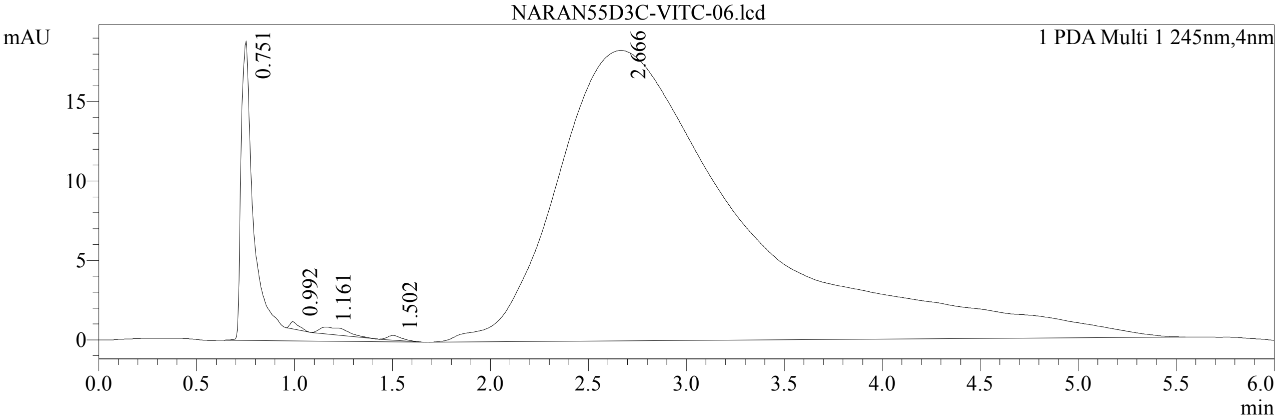
NARAN55D3C-VITC-06.lcd
mAU
1 PDA Multi 1 245nm,4nm
0.751
0.992
1.161
1.502
2.666
min

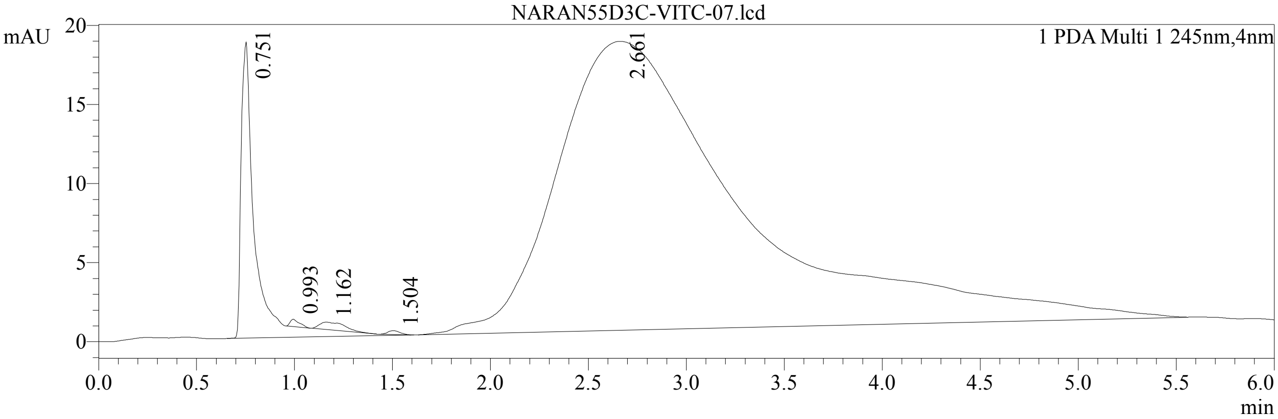
NARAN55D3C-VITC-07.lcd
mAU
1 PDA Multi 1 245nm,4nm
0.751
0.993
1.162
1.504
2.661
min

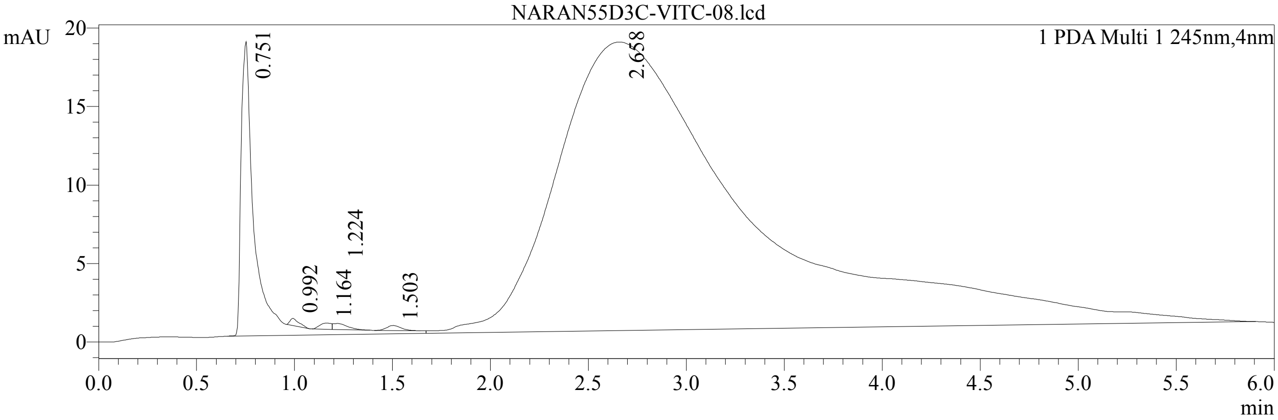
NARAN55D3C-VITC-08.lcd
mAU
1 PDA Multi 1 245nm,4nm
0.751
0.992
1.164
1.224
1.503
2.658
min

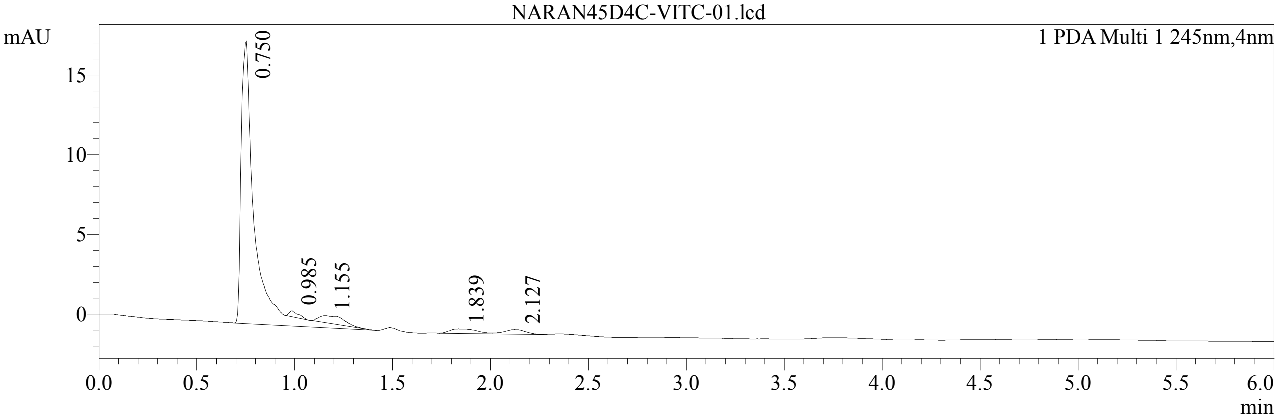
NARAN45D4C-VITC-01.lcd
mAU
1 PDA Multi 1 245nm,4nm
0.750
0.985
1.155
1.839
2.127
min

NARAN45D4C-VITC-02.lcd

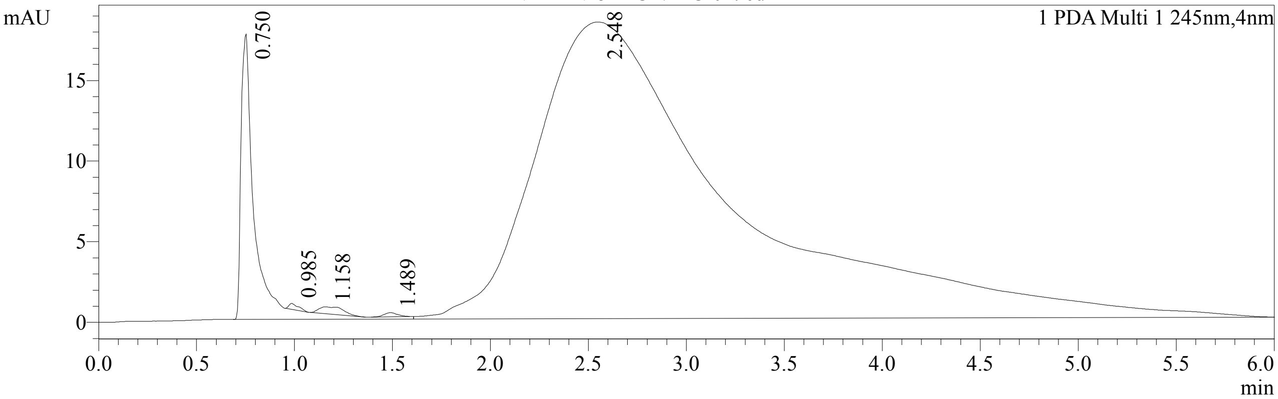

NARAN45D4C-VITC-03.lcd

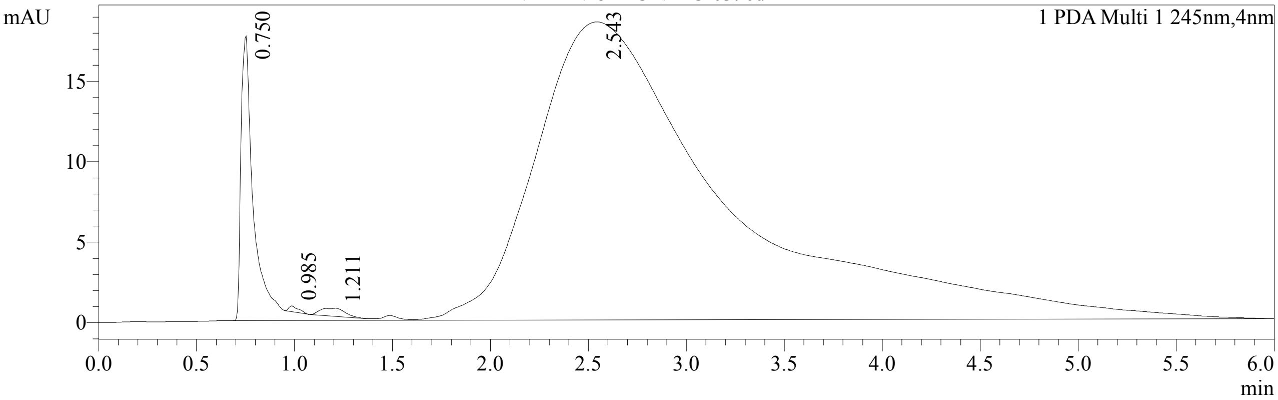

NARAN45D4C-VITC-04.lcd

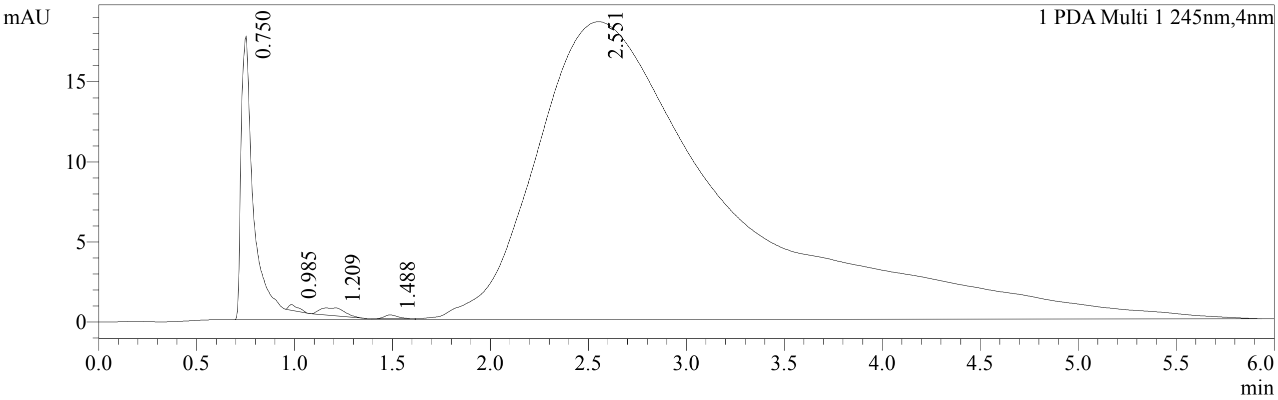

NARAN55D4C-VITC-05.lcd

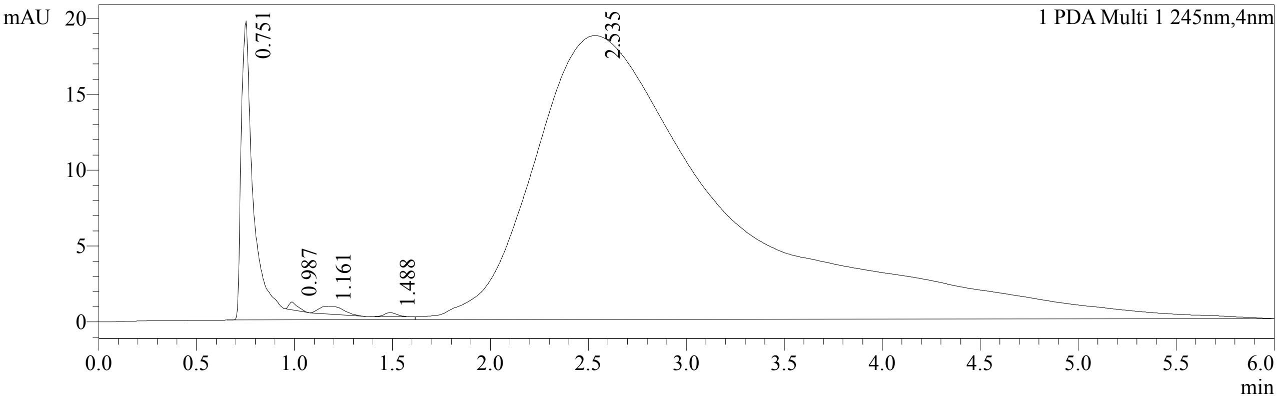

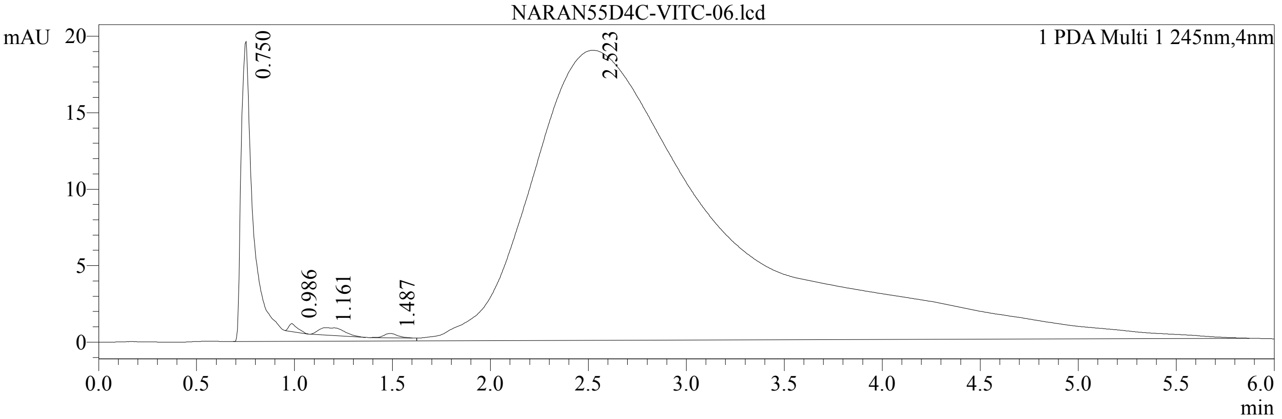
NARAN55D4C-VITC-06.lcd
mAU
1 PDA Multi 1 245nm,4nm
0.750
0.986
1.161
1.487
2.523
20
15
10
5
0
0.0 0.5 1.0 1.5 2.0 2.5 3.0 3.5 4.0 4.5 5.0 5.5 6.0
min

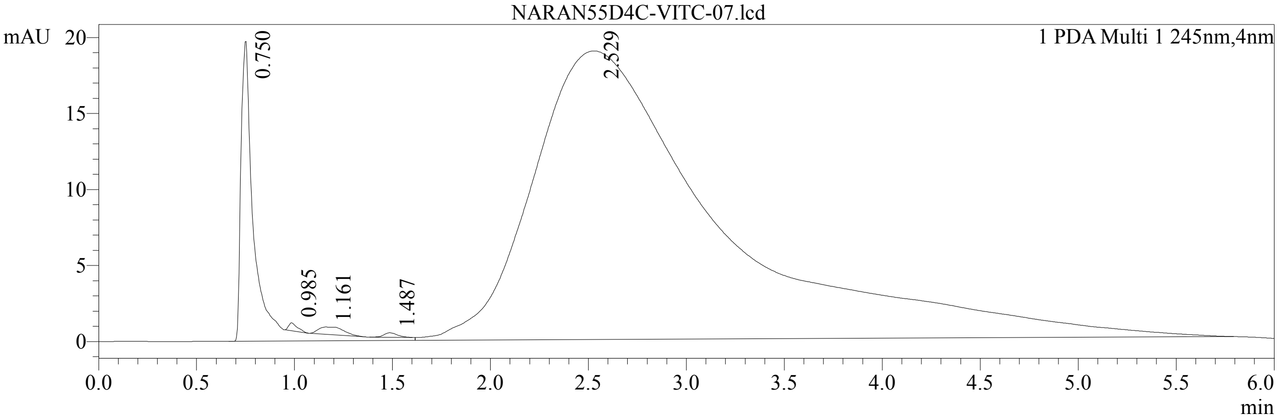
NARAN55D4C-VITC-07.lcd
mAU
1 PDA Multi 1 245nm,4nm
0.750
0.985
1.161
1.487
2.529
20
15
10
5
0
0.0 0.5 1.0 1.5 2.0 2.5 3.0 3.5 4.0 4.5 5.0 5.5 6.0
min

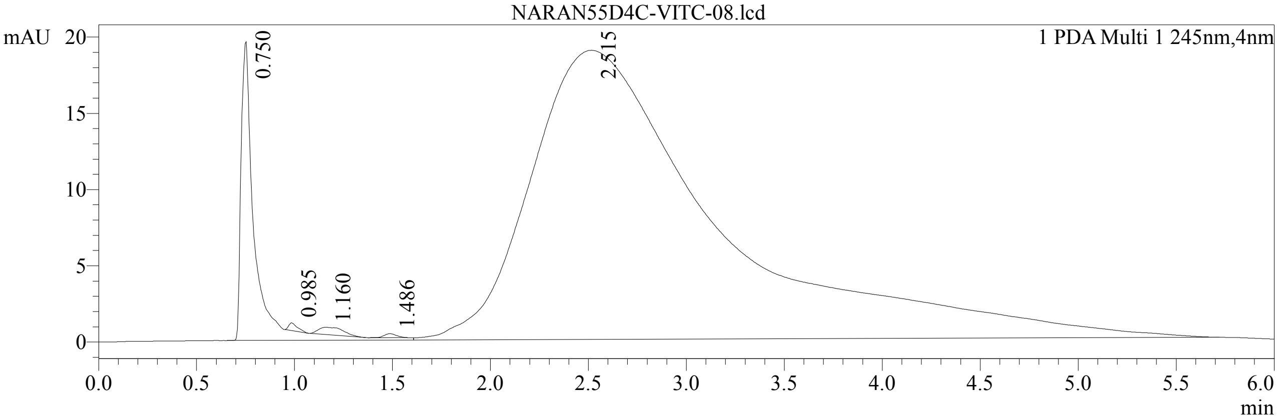
NARAN55D4C-VITC-08.lcd
mAU
1 PDA Multi 1 245nm,4nm
0.750
0.985
1.160
1.486
2.515
20
15
10
5
0
0.0 0.5 1.0 1.5 2.0 2.5 3.0 3.5 4.0 4.5 5.0 5.5 6.0
min

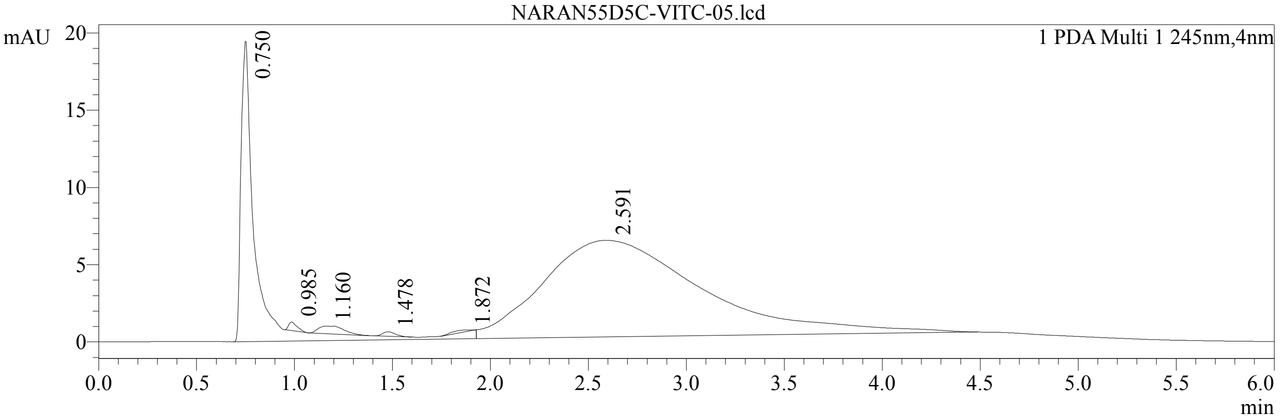
NARAN55D5C-VITC-05.lcd
mAU
1 PDA Multi 1 245nm,4nm
0.750
0.985
1.160
1.478
1.872
2.591
20
15
10
5
0
0.0 0.5 1.0 1.5 2.0 2.5 3.0 3.5 4.0 4.5 5.0 5.5 6.0
min

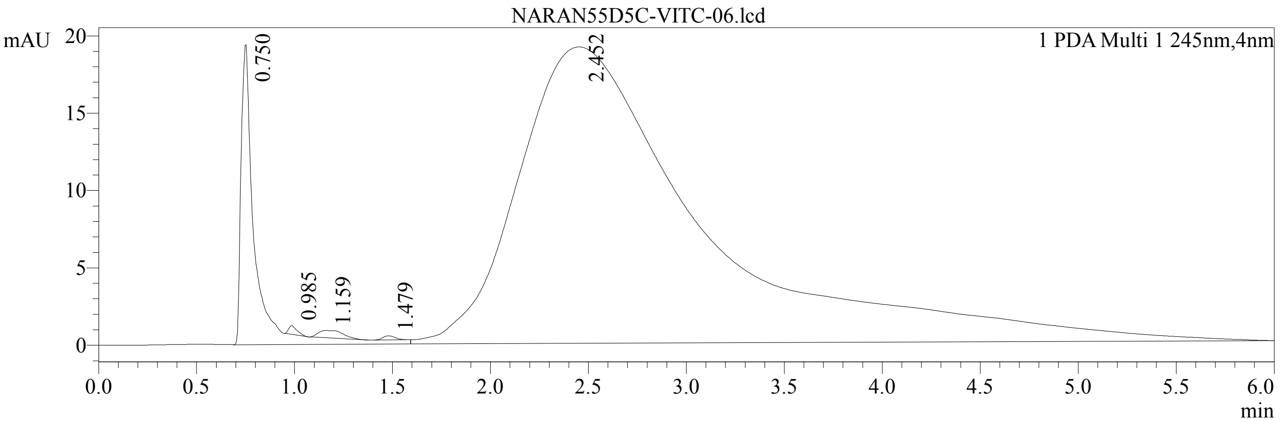
NARAN55D5C-VITC-06.lcd
mAU
1 PDA Multi 1 245nm,4nm
0.750
0.985
1.159
1.479
2.452
min

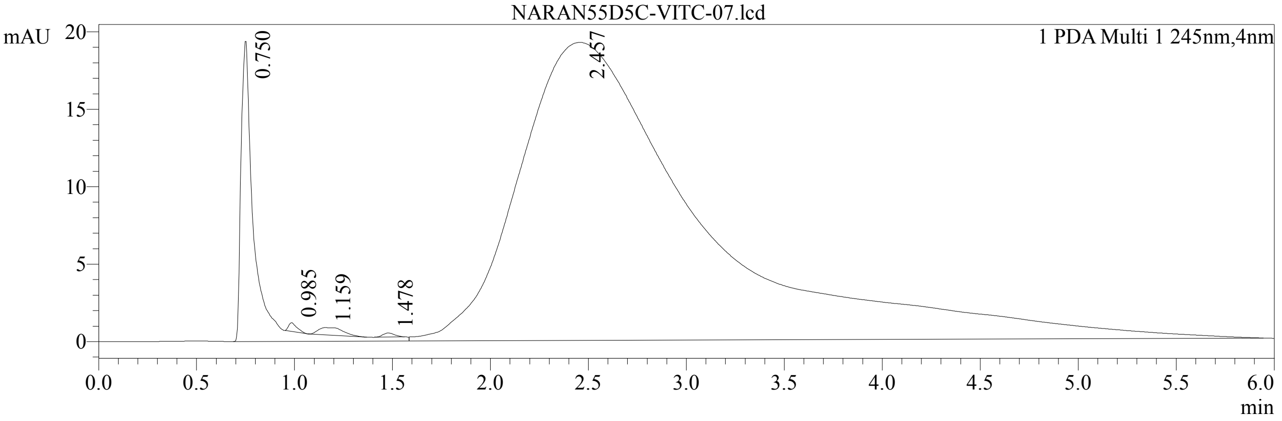
NARAN55D5C-VITC-07.lcd
mAU
1 PDA Multi 1 245nm,4nm
0.750
0.985
1.159
1.478
2.457
min

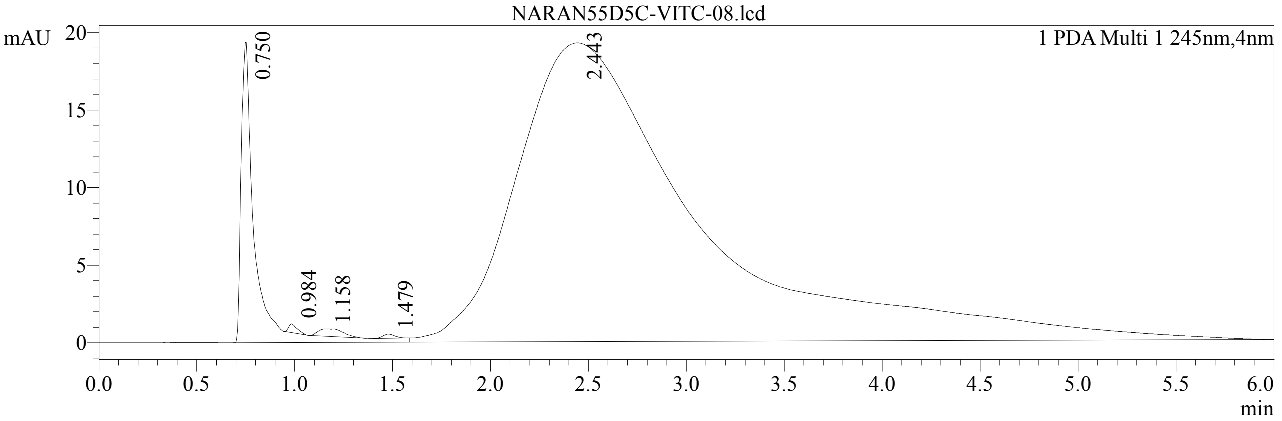
NARAN55D5C-VITC-08.lcd
mAU
1 PDA Multi 1 245nm,4nm
0.750
0.984
1.158
1.479
2.443
min

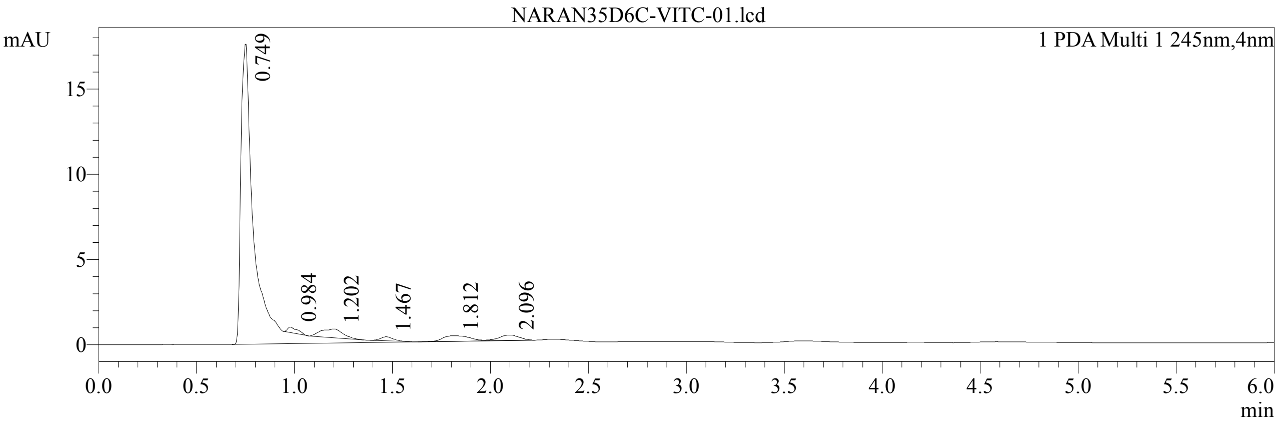
NARAN35D6C-VITC-01.lcd
mAU
1 PDA Multi 1 245nm,4nm
0.749
0.984
1.202
1.467
1.812
2.096
min

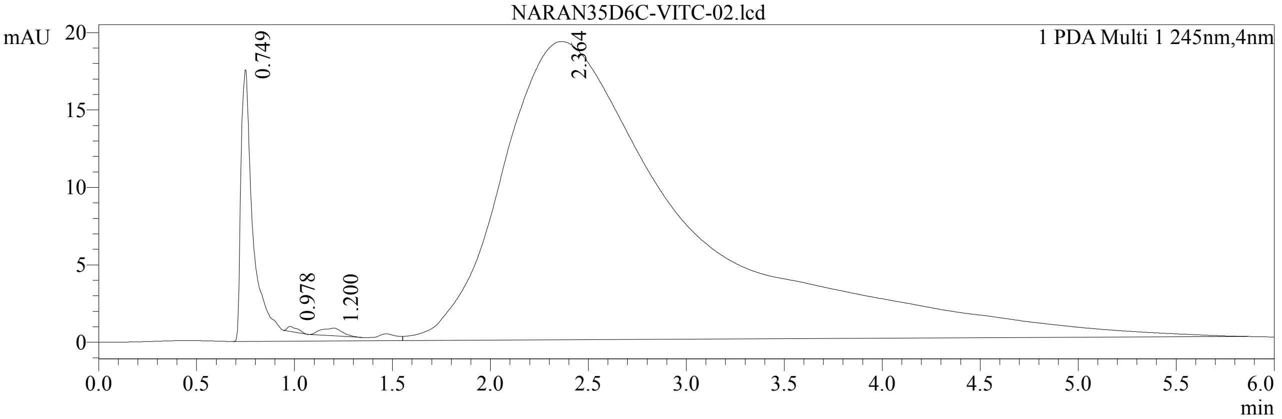
NARAN35D6C-VITC-02.lcd
mAU
1 PDA Multi 1 245nm,4nm
0.749
0.978
1.200
2.364
20
15
10
5
0
0.0
0.5
1.0
1.5
2.0
2.5
3.0
3.5
4.0
4.5
5.0
5.5
6.0
min

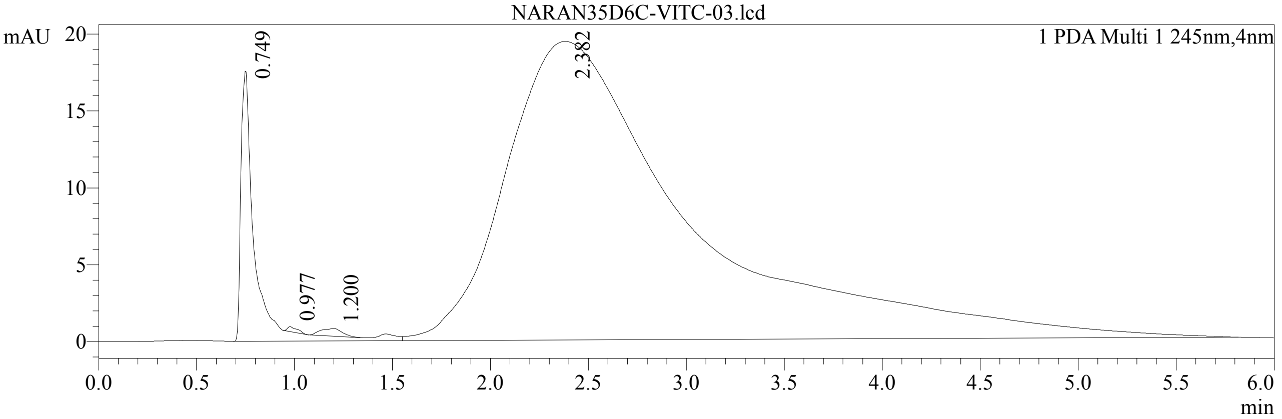
NARAN35D6C-VITC-03.lcd
mAU
1 PDA Multi 1 245nm,4nm
0.749
0.977
1.200
2.382
20
15
10
5
0
0.0
0.5
1.0
1.5
2.0
2.5
3.0
3.5
4.0
4.5
5.0
5.5
6.0
min

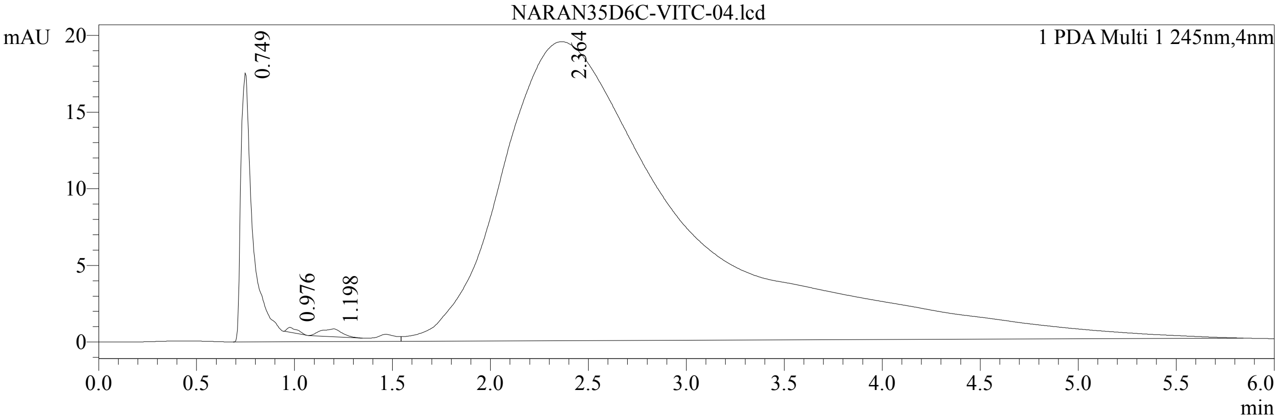
NARAN35D6C-VITC-04.lcd
mAU
1 PDA Multi 1 245nm,4nm
0.749
0.976
1.198
2.364
20
15
10
5
0
0.0
0.5
1.0
1.5
2.0
2.5
3.0
3.5
4.0
4.5
5.0
5.5
6.0
min

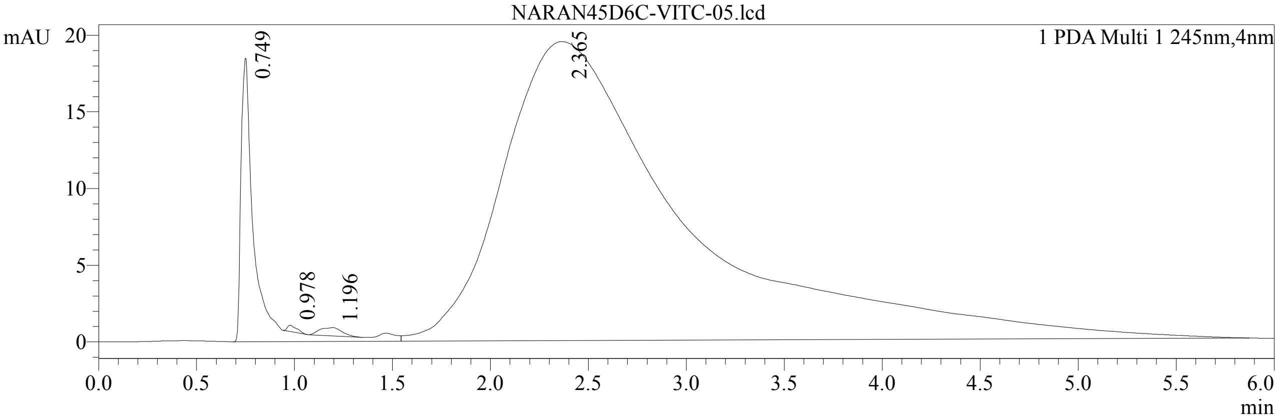
NARAN45D6C-VITC-05.lcd
mAU
1 PDA Multi 1 245nm,4nm
0.749
0.978
1.196
2.365
20
15
10
5
0
0.0
0.5
1.0
1.5
2.0
2.5
3.0
3.5
4.0
4.5
5.0
5.5
6.0
min

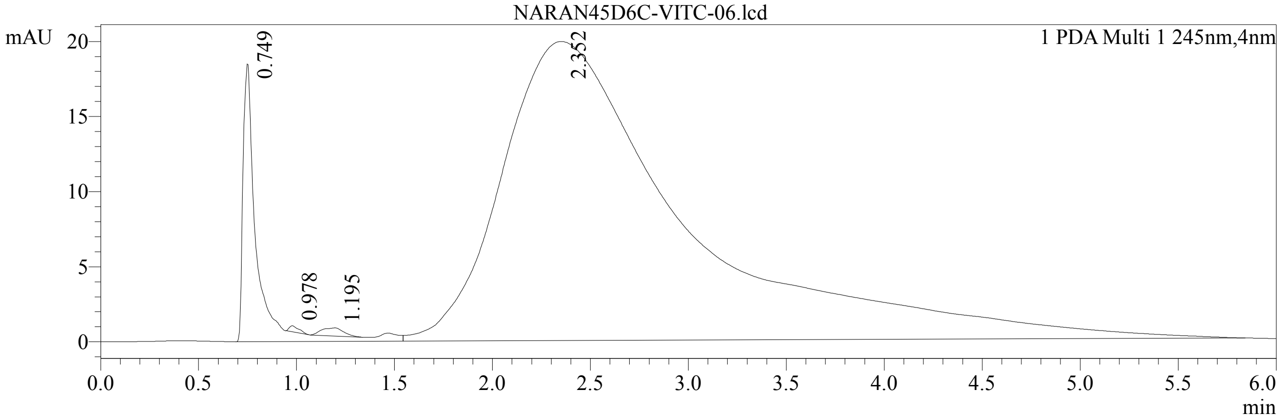
NARAN45D6C-VITC-06.lcd
mAU
1 PDA Multi 1 245nm,4nm
0.749
0.978
1.195
2.352
0 5 10 15 20
0.0 0.5 1.0 1.5 2.0 2.5 3.0 3.5 4.0 4.5 5.0 5.5 6.0
min

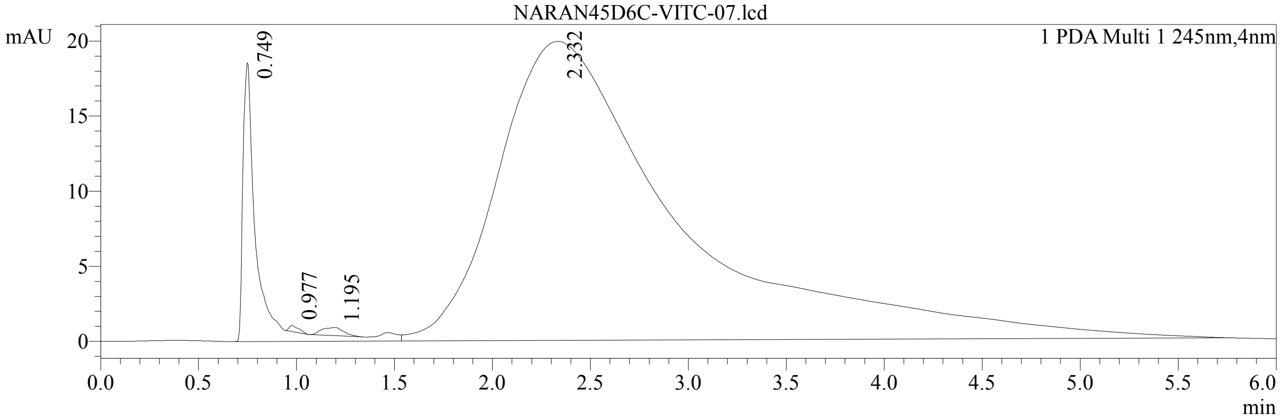
NARAN45D6C-VITC-07.lcd
mAU
1 PDA Multi 1 245nm,4nm
0.749
0.977
1.195
2.332
0 5 10 15 20
0.0 0.5 1.0 1.5 2.0 2.5 3.0 3.5 4.0 4.5 5.0 5.5 6.0
min

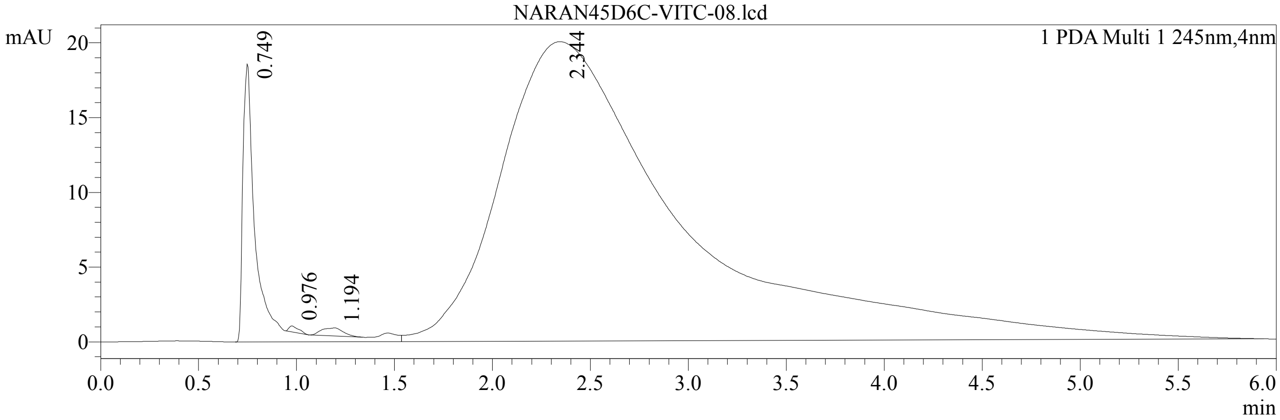
NARAN45D6C-VITC-08.lcd
mAU
1 PDA Multi 1 245nm,4nm
0.749
0.976
1.194
2.344
0 5 10 15 20
0.0 0.5 1.0 1.5 2.0 2.5 3.0 3.5 4.0 4.5 5.0 5.5 6.0
min

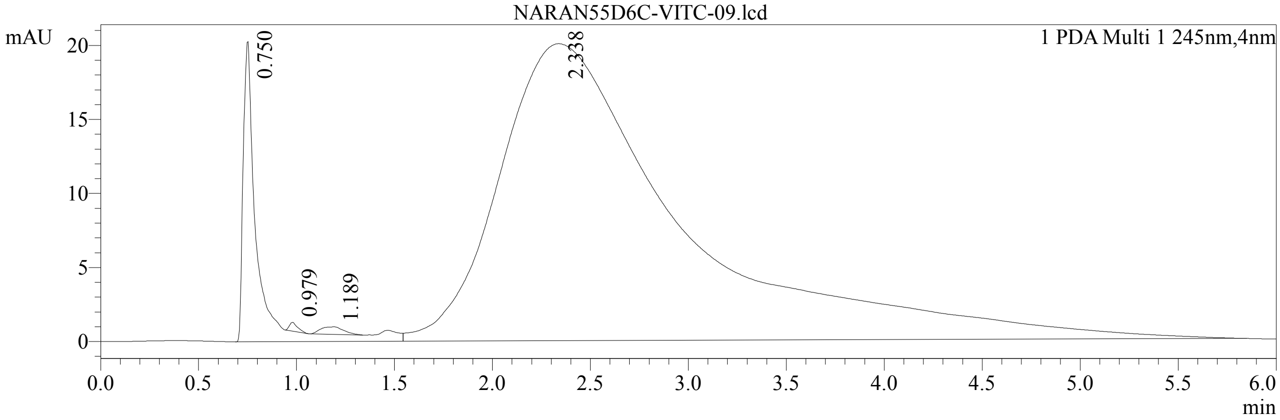
NARAN55D6C-VITC-09.lcd
mAU
1 PDA Multi 1 245nm,4nm
0.750
0.979
1.189
2.338
0 5 10 15 20
0.0 0.5 1.0 1.5 2.0 2.5 3.0 3.5 4.0 4.5 5.0 5.5 6.0
min

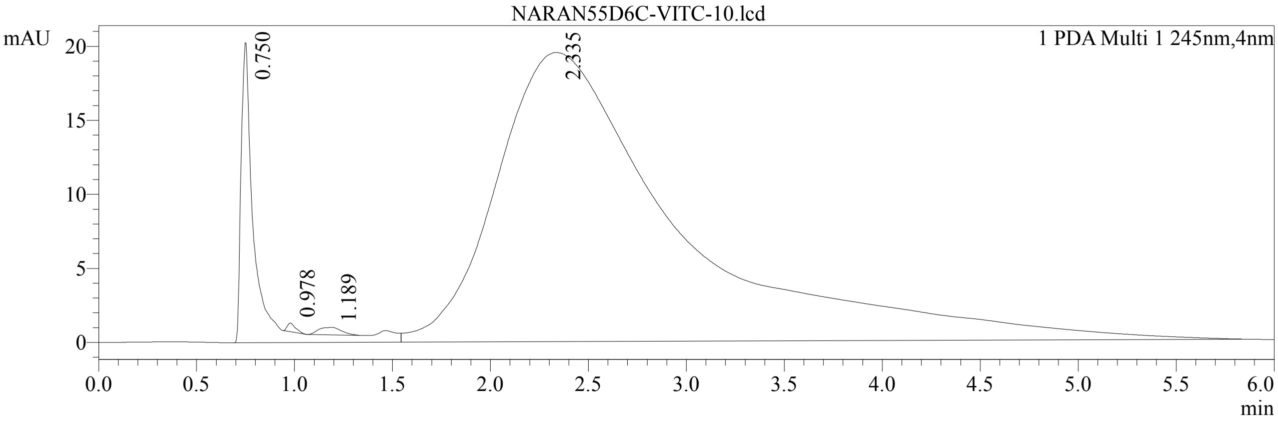
NARAN55D6C-VITC-10.lcd
mAU
1 PDA Multi 1 245nm,4nm
0.750
0.978
1.189
2.335
min

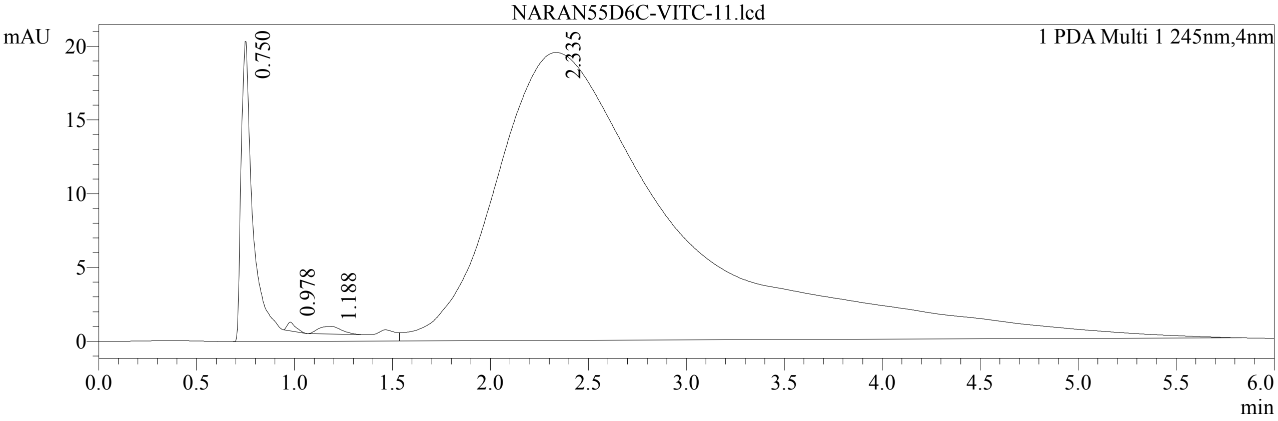
NARAN55D6C-VITC-11.lcd
mAU
1 PDA Multi 1 245nm,4nm
0.750
0.978
1.188
2.335
min

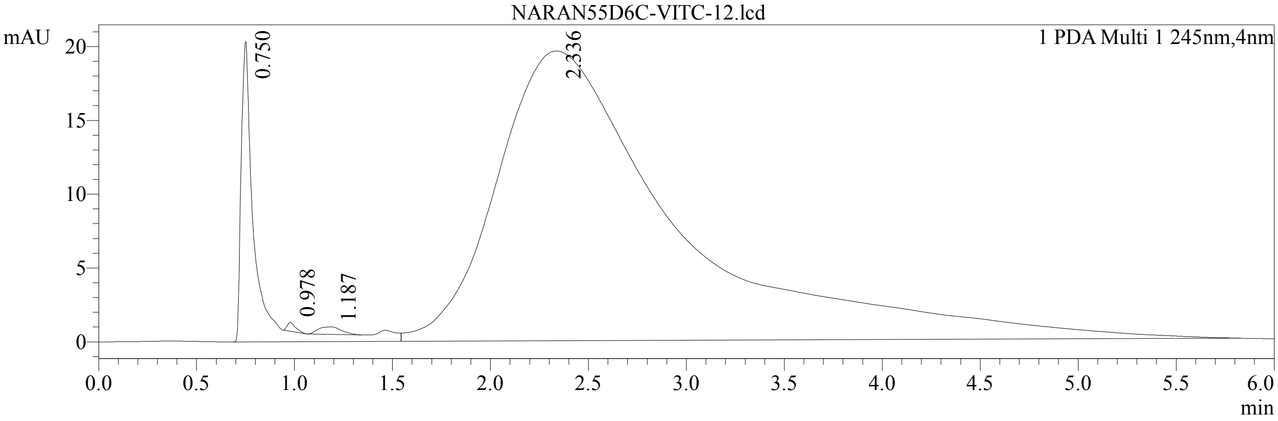
NARAN55D6C-VITC-12.lcd
mAU
1 PDA Multi 1 245nm,4nm
0.750
0.978
1.187
2.336
min

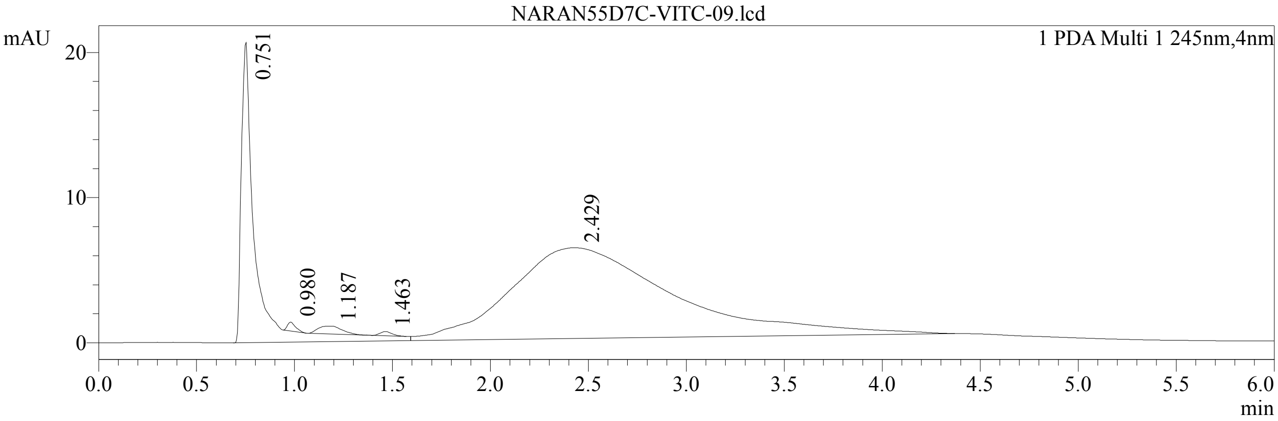
NARAN55D7C-VITC-09.lcd
mAU
1 PDA Multi 1 245nm,4nm
0.751
0.980
1.187
1.463
2.429
min

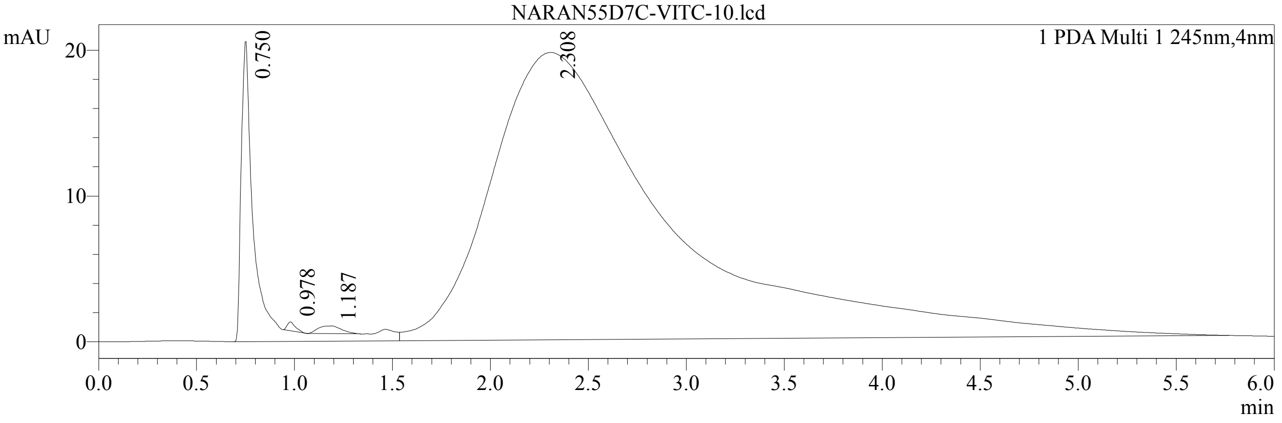
NARAN55D7C-VITC-10.lcd
mAU
1 PDA Multi 1 245nm,4nm
0.750
0.978
1.187
2.308
0
10
20
0.0
0.5
1.0
1.5
2.0
2.5
3.0
3.5
4.0
4.5
5.0
5.5
6.0
min

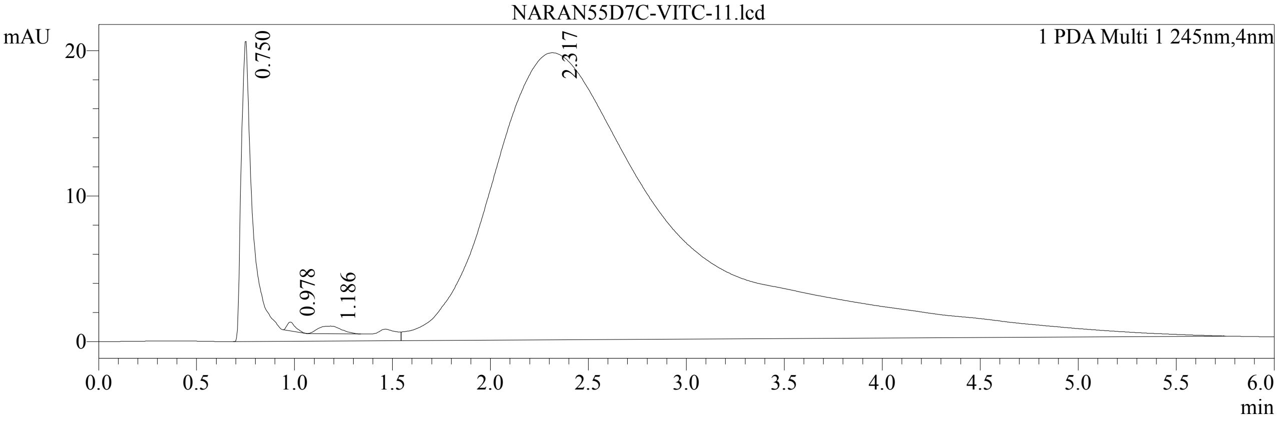
NARAN55D7C-VITC-11.lcd
mAU
1 PDA Multi 1 245nm,4nm
0.750
0.978
1.186
2.317
0
10
20
0.0
0.5
1.0
1.5
2.0
2.5
3.0
3.5
4.0
4.5
5.0
5.5
6.0
min

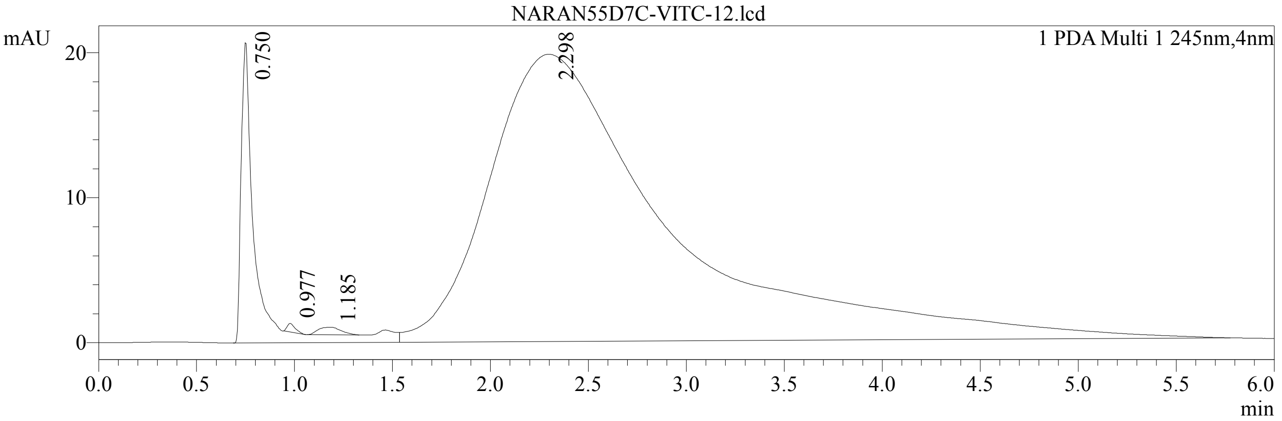
NARAN55D7C-VITC-12.lcd
mAU
1 PDA Multi 1 245nm,4nm
0.750
0.977
1.185
2.298
0
10
20
0.0
0.5
1.0
1.5
2.0
2.5
3.0
3.5
4.0
4.5
5.0
5.5
6.0
min

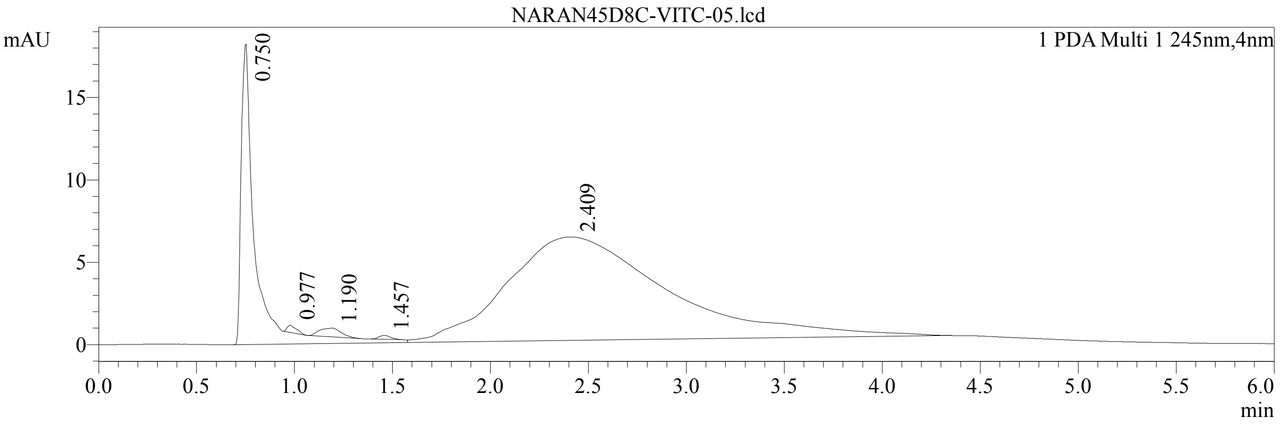
NARAN45D8C-VITC-05.lcd
mAU
1 PDA Multi 1 245nm,4nm
0.750
0.977
1.190
1.457
2.409
0
5
10
15
0.0
0.5
1.0
1.5
2.0
2.5
3.0
3.5
4.0
4.5
5.0
5.5
6.0
min

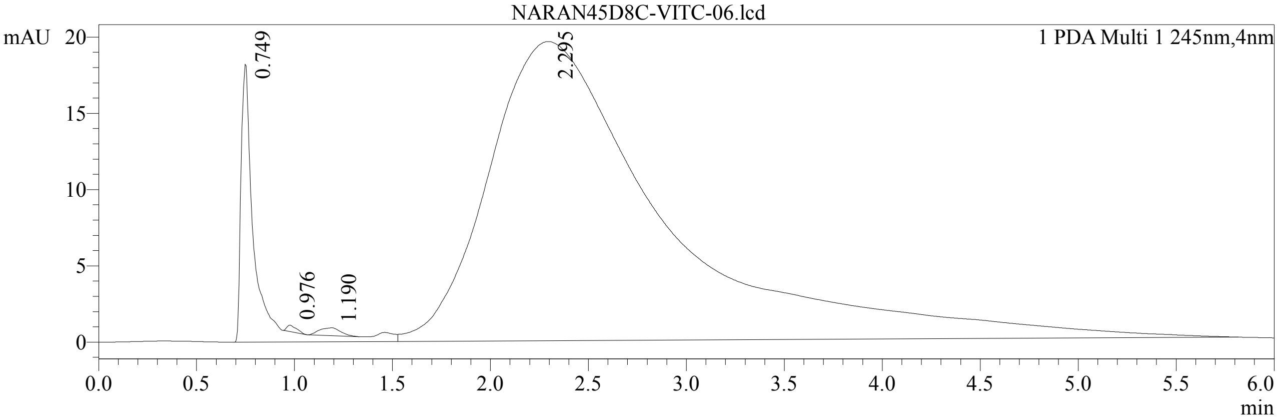
NARAN45D8C-VITC-06.lcd
mAU
1 PDA Multi 1 245nm,4nm
0.749
0.976
1.190
2.295
min

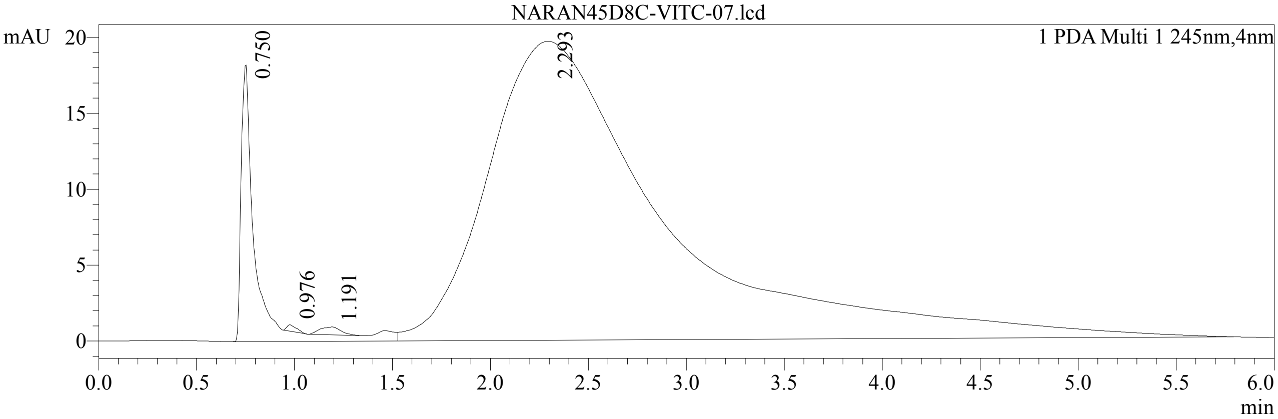
NARAN45D8C-VITC-07.lcd
mAU
1 PDA Multi 1 245nm,4nm
0.750
0.976
1.191
2.293
min

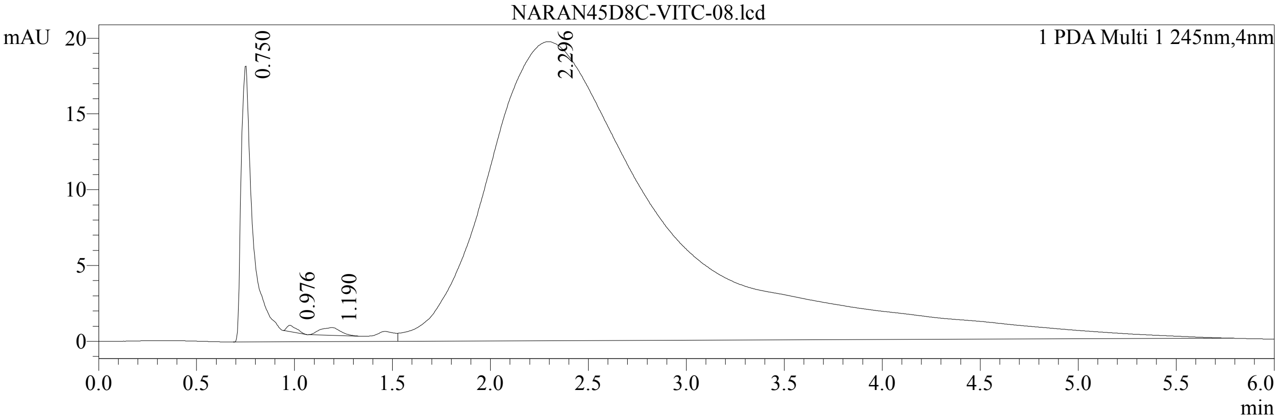
NARAN45D8C-VITC-08.lcd
mAU
1 PDA Multi 1 245nm,4nm
0.750
0.976
1.190
2.296
min

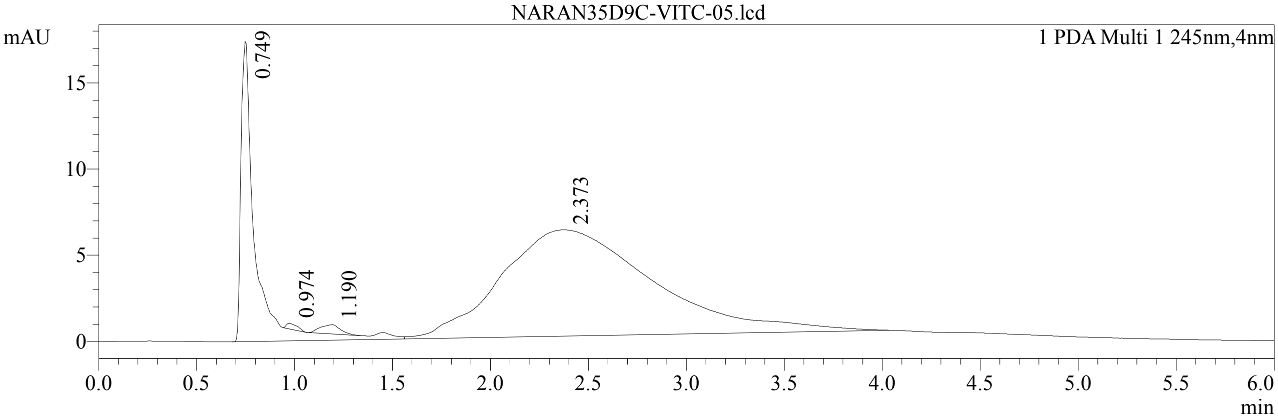
NARAN35D9C-VITC-05.lcd
mAU
1 PDA Multi 1 245nm,4nm
0.749
0.974
1.190
2.373
min

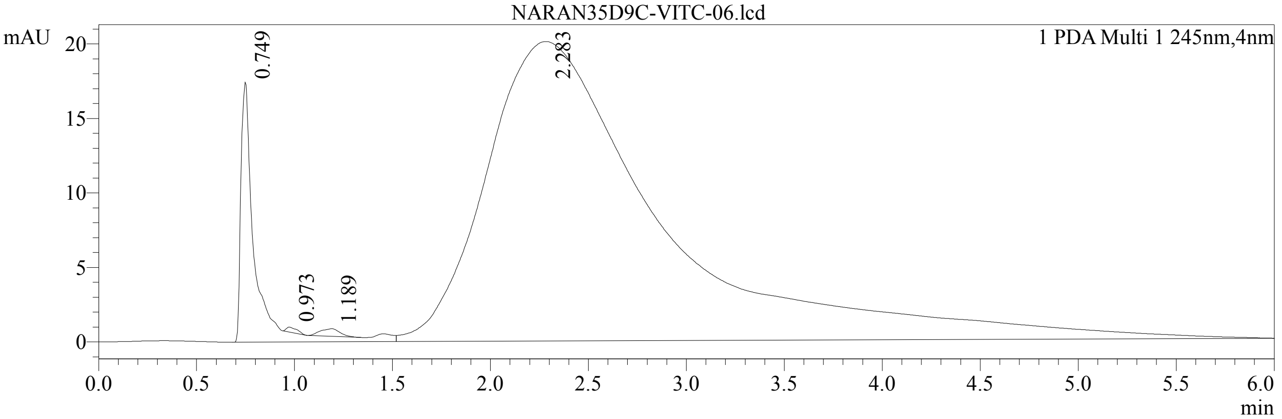
NARAN35D9C-VITC-06.lcd
mAU
1 PDA Multi 1 245nm,4nm
0.749
0.973
1.189
2.283
0
5
10
15
20
0.0
0.5
1.0
1.5
2.0
2.5
3.0
3.5
4.0
4.5
5.0
5.5
6.0
min

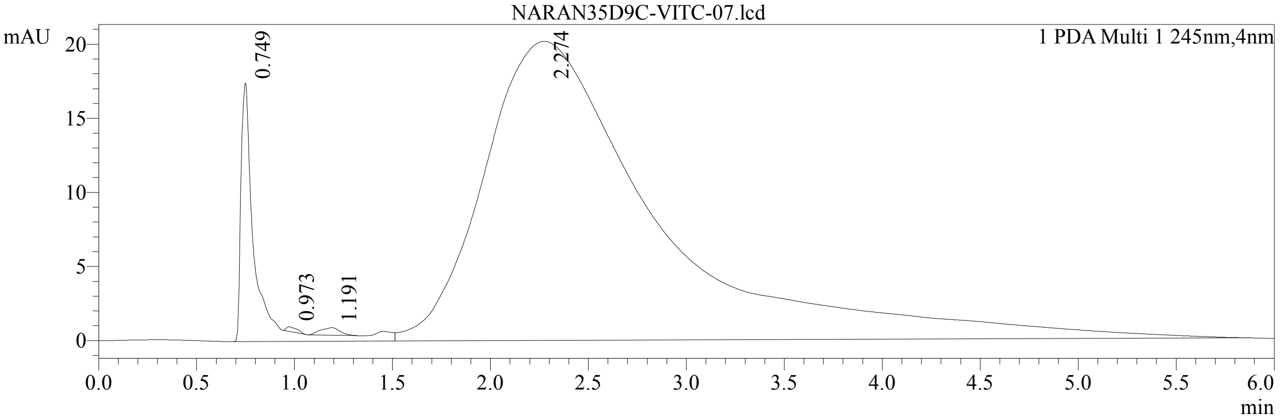
NARAN35D9C-VITC-07.lcd
mAU
1 PDA Multi 1 245nm,4nm
0.749
0.973
1.191
2.274
0
5
10
15
20
0.0
0.5
1.0
1.5
2.0
2.5
3.0
3.5
4.0
4.5
5.0
5.5
6.0
min

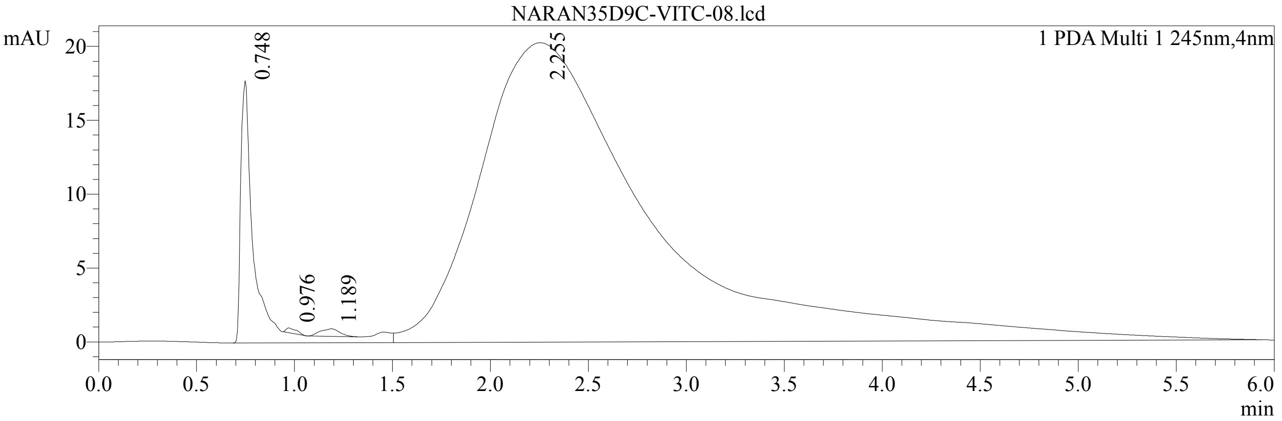
NARAN35D9C-VITC-08.lcd
mAU
1 PDA Multi 1 245nm,4nm
0.748
0.976
1.189
2.255
0
5
10
15
20
0.0
0.5
1.0
1.5
2.0
2.5
3.0
3.5
4.0
4.5
5.0
5.5
6.0
min

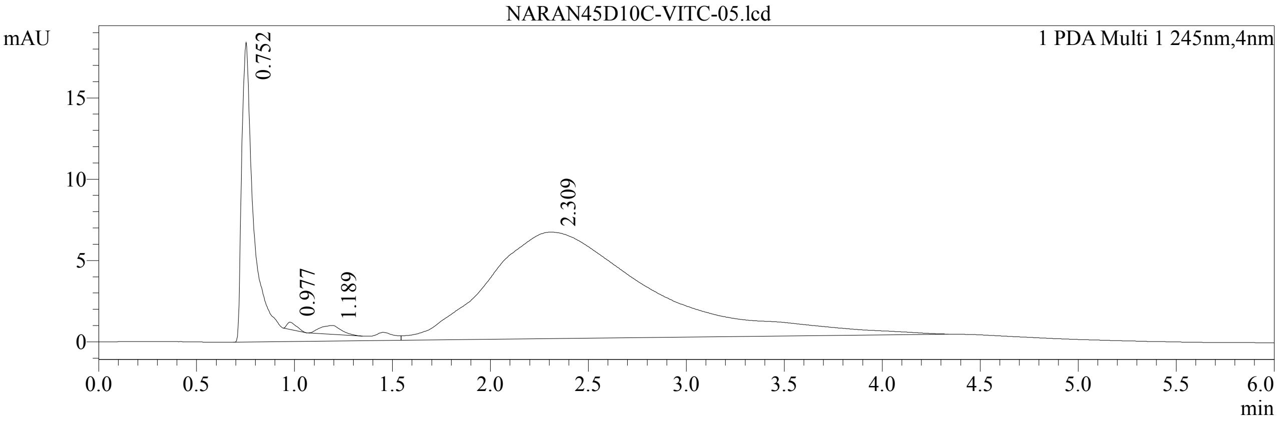
NARAN45D10C-VITC-05.lcd
mAU
1 PDA Multi 1 245nm,4nm
0.752
0.977
1.189
2.309
0
5
10
15
0.0
0.5
1.0
1.5
2.0
2.5
3.0
3.5
4.0
4.5
5.0
5.5
6.0
min

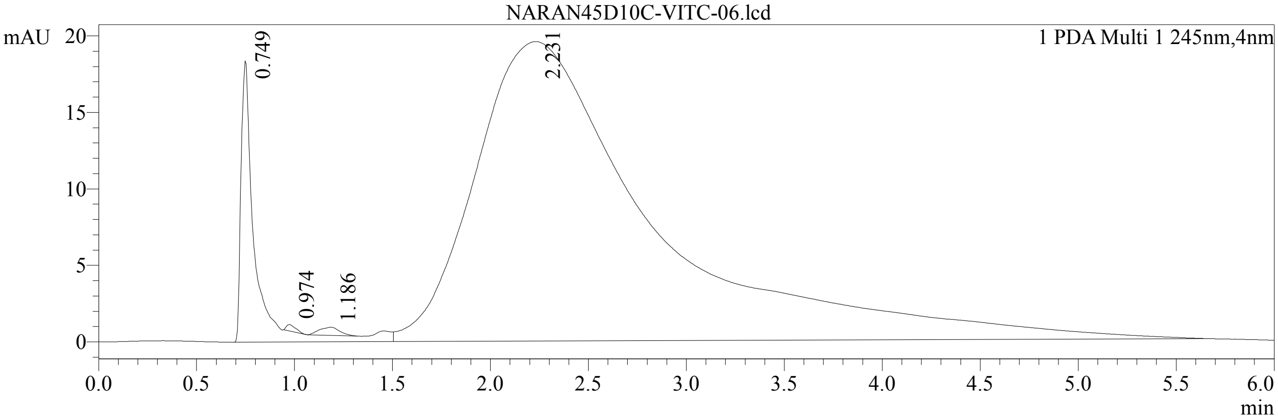
NARAN45D10C-VITC-06.lcd
mAU
1 PDA Multi 1 245nm,4nm
0.749
0.974
1.186
2.231
min

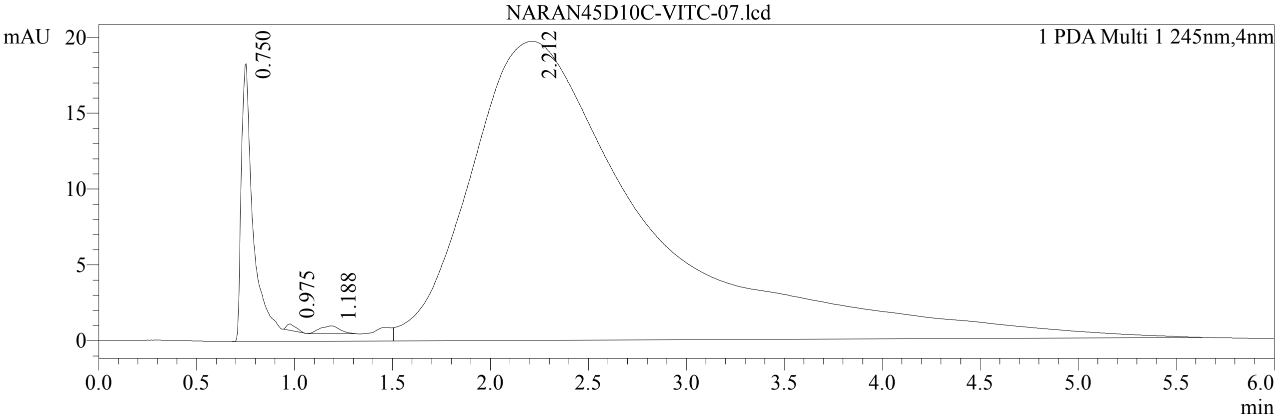
NARAN45D10C-VITC-07.lcd
mAU
1 PDA Multi 1 245nm,4nm
0.750
0.975
1.188
2.212
min

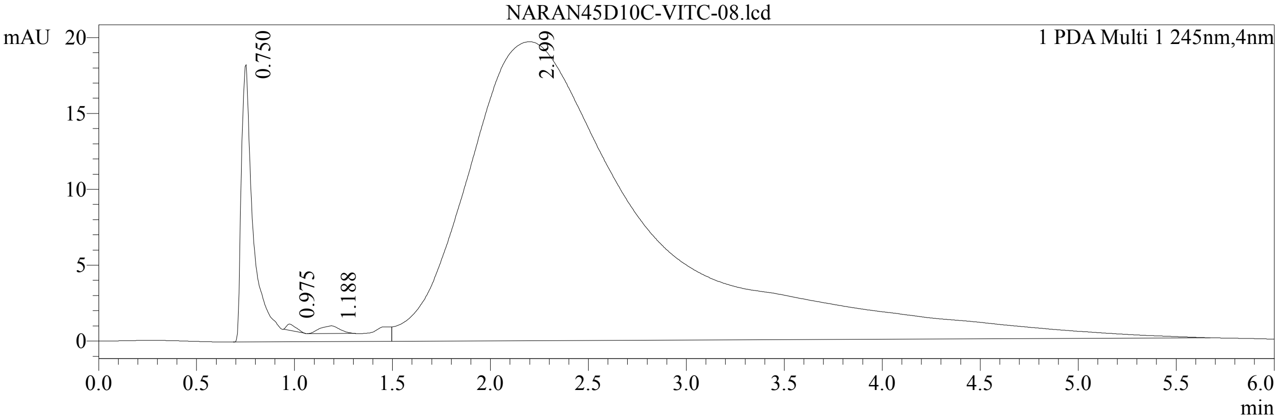
NARAN45D10C-VITC-08.lcd
mAU
1 PDA Multi 1 245nm,4nm
0.750
0.975
1.188
2.199
min

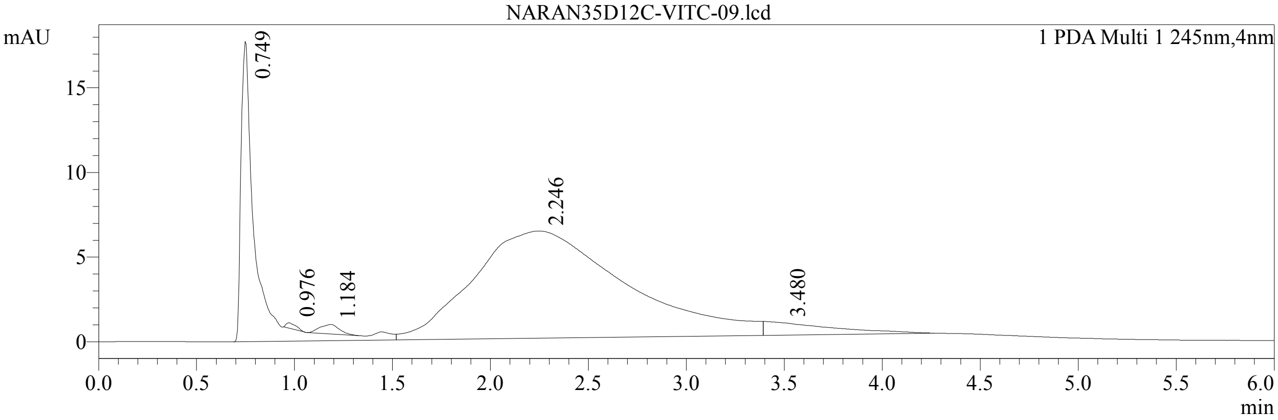
NARAN35D12C-VITC-09.lcd
mAU
1 PDA Multi 1 245nm,4nm
0.749
0.976
1.184
2.246
3.480
min

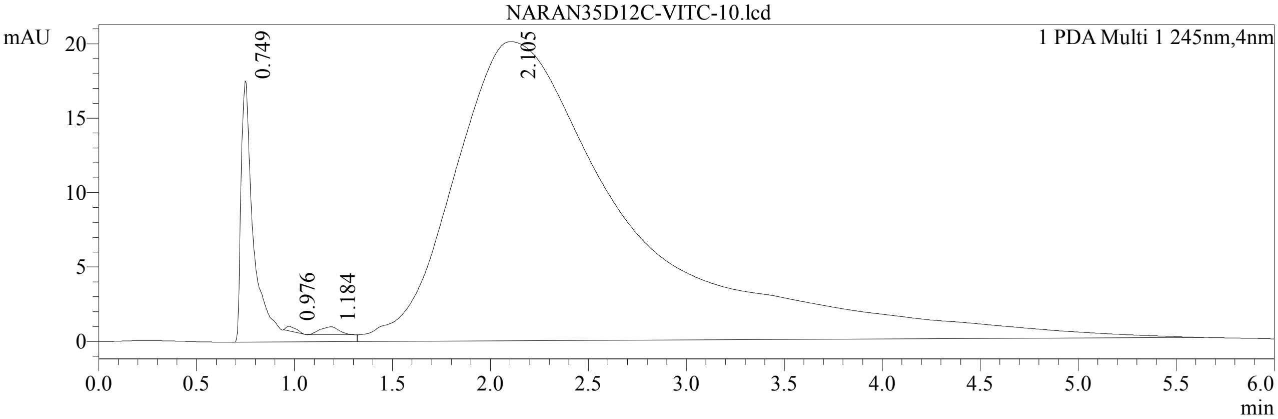
NARAN35D12C-VITC-10.lcd
mAU
1 PDA Multi 1 245nm,4nm
0.749
0.976
1.184
2.105
min

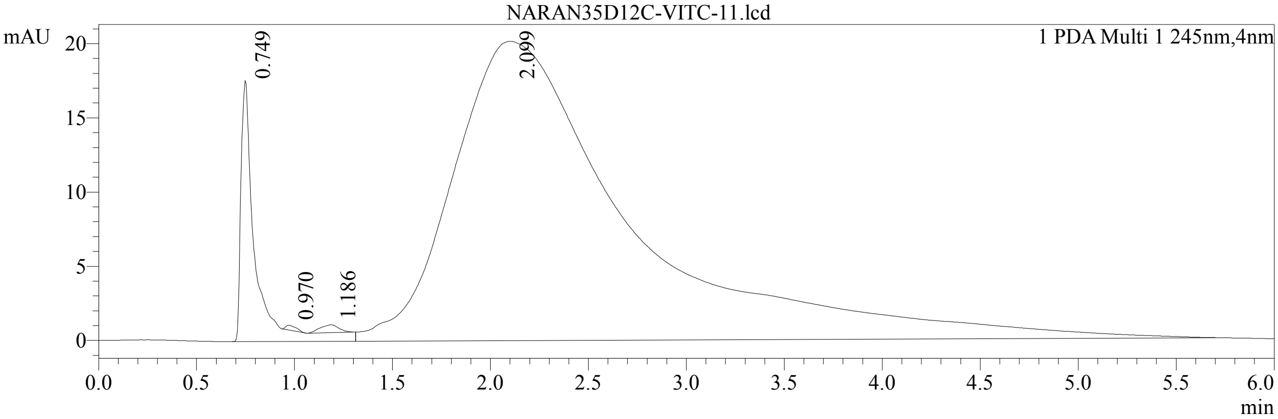
NARAN35D12C-VITC-11.lcd
mAU
1 PDA Multi 1 245nm,4nm
0.749
0.970
1.186
2.099
min

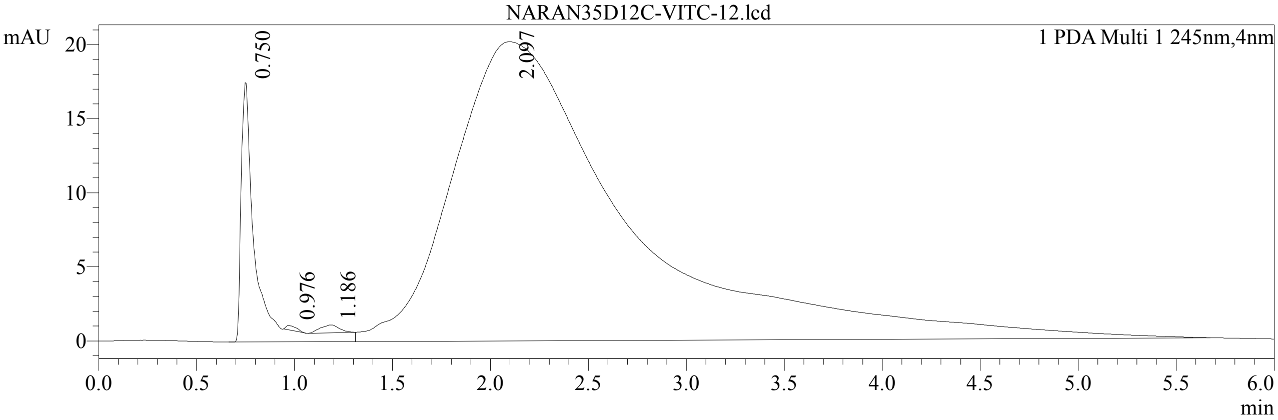
NARAN35D12C-VITC-12.lcd
mAU
1 PDA Multi 1 245nm,4nm
0.750
0.976
1.186
2.097
min

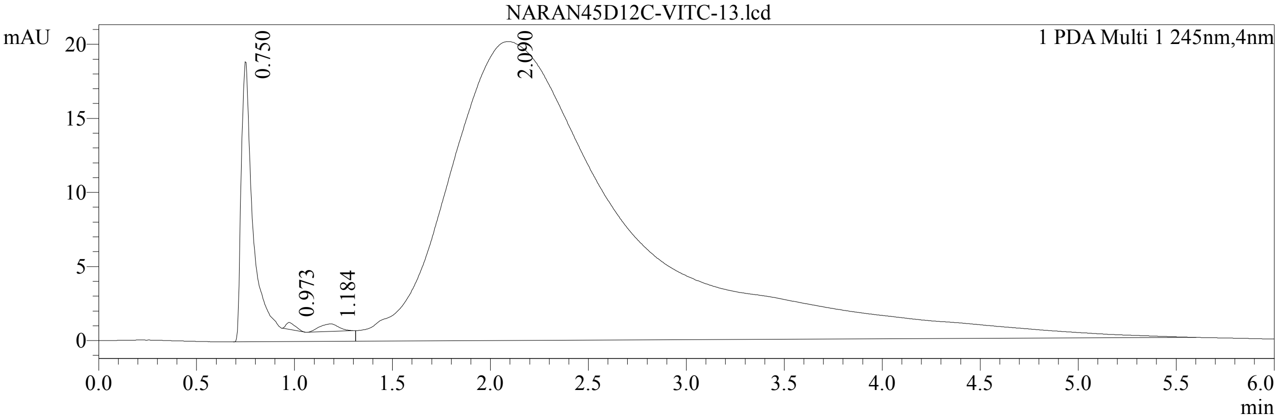
NARAN45D12C-VITC-13.lcd
mAU
1 PDA Multi 1 245nm,4nm
0.750
0.973
1.184
2.090
min

NARAN45D12C-VITC-14.lcd

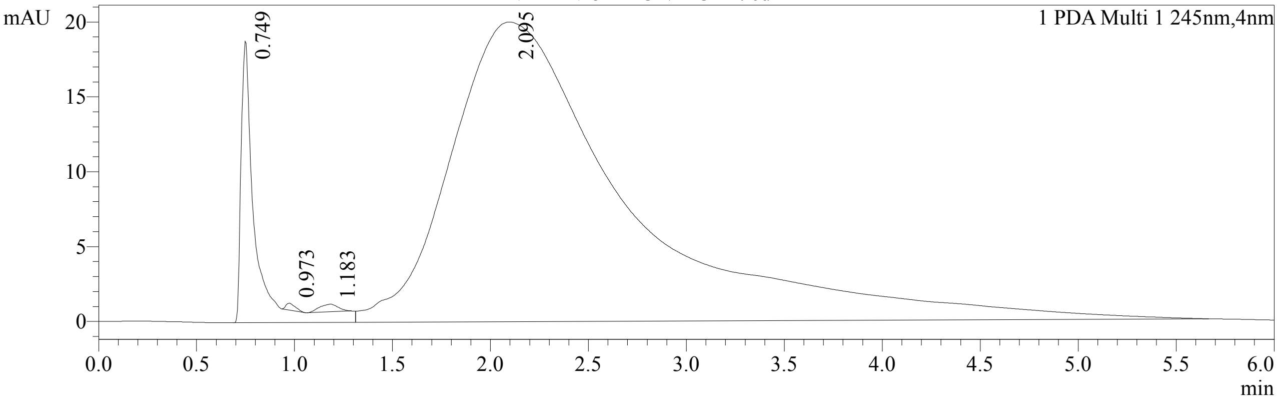

NARAN45D12C-VITC-15.lcd

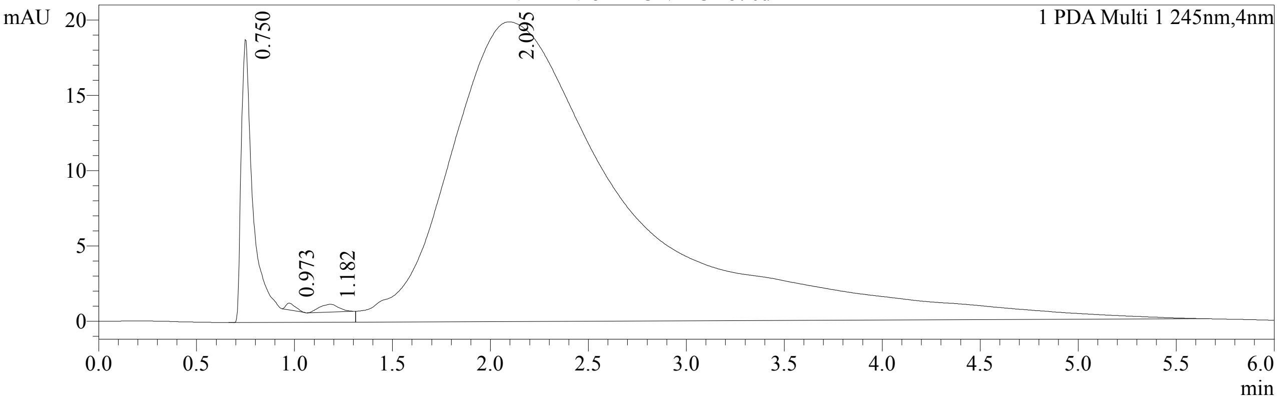

NARAN45D12C-VITC-16.lcd

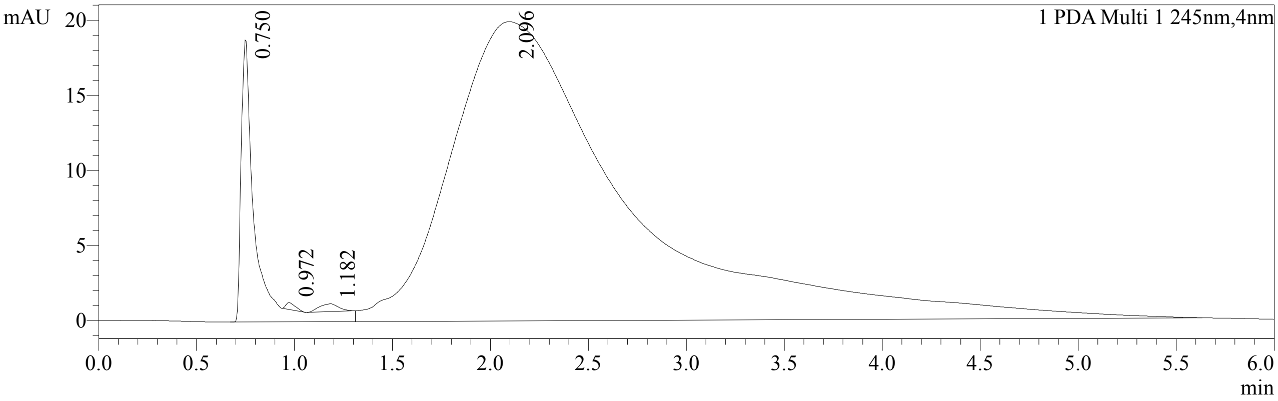

NARAN45D14C-VITC-05.lcd

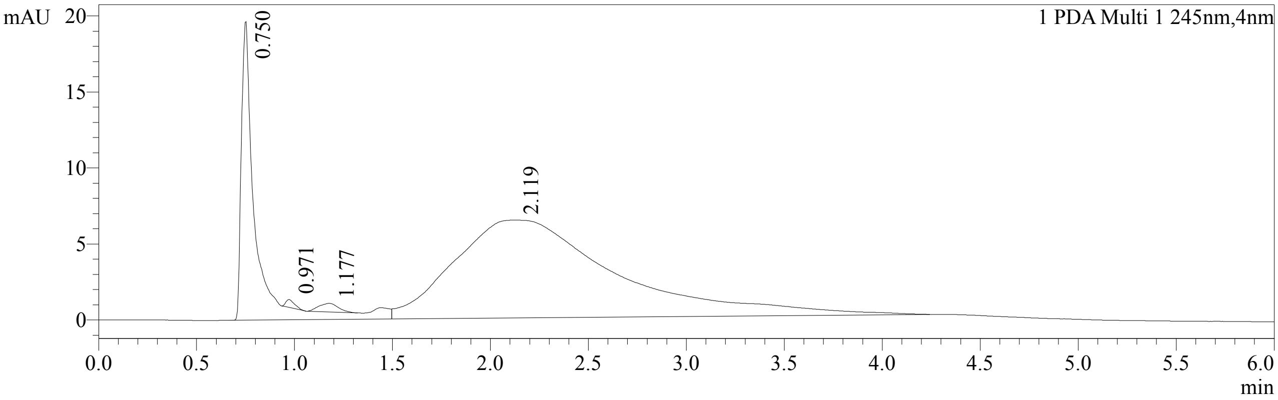

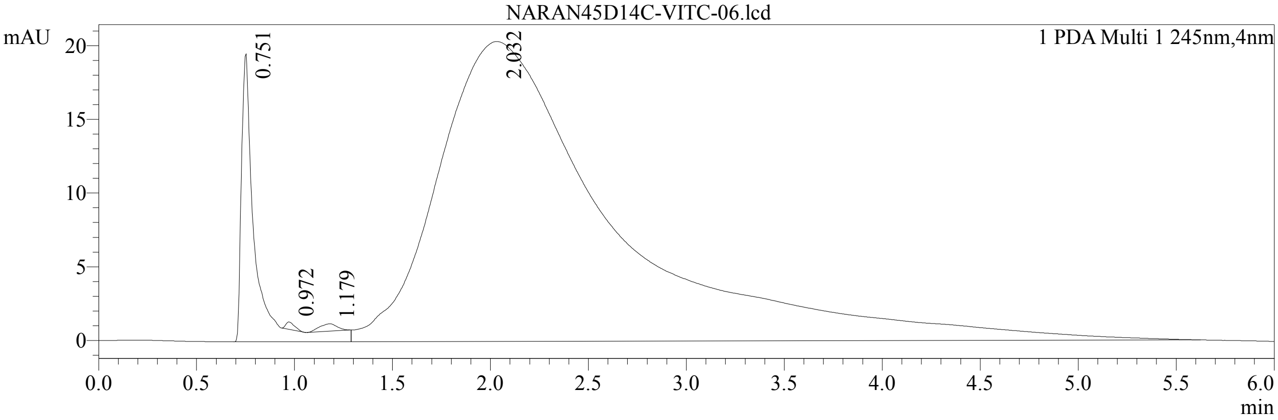
NARAN45D14C-VITC-06.lcd
mAU
1 PDA Multi 1 245nm,4nm
0.751
0.972
1.179
2.032
min

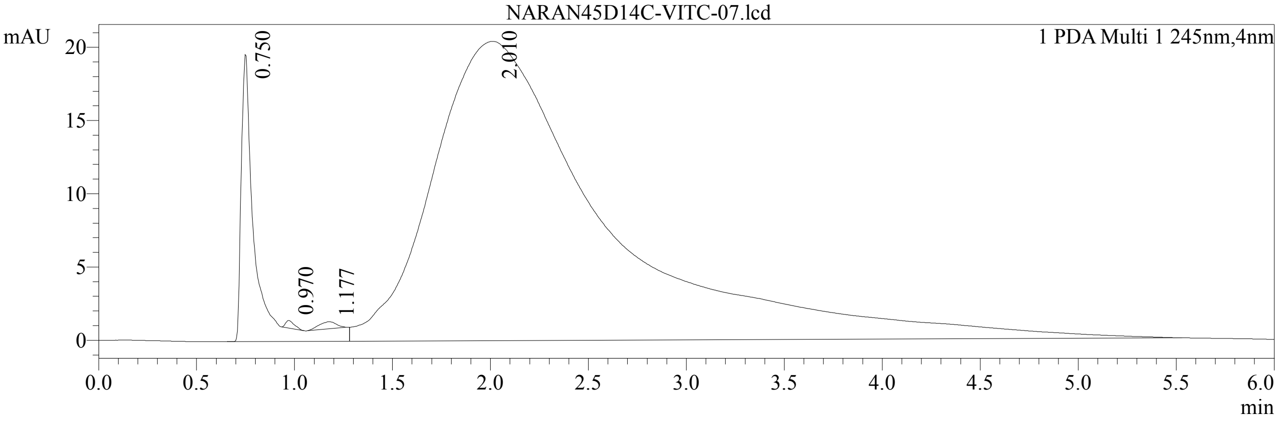
NARAN45D14C-VITC-07.lcd
mAU
1 PDA Multi 1 245nm,4nm
0.750
0.970
1.177
2.010
min

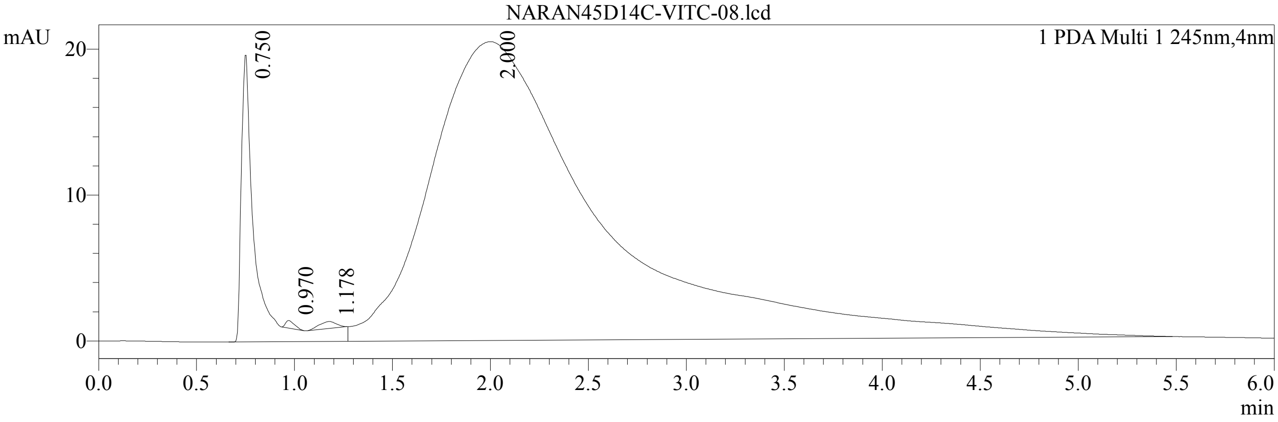
NARAN45D14C-VITC-08.lcd
mAU
1 PDA Multi 1 245nm,4nm
0.750
0.970
1.178
2.000
min

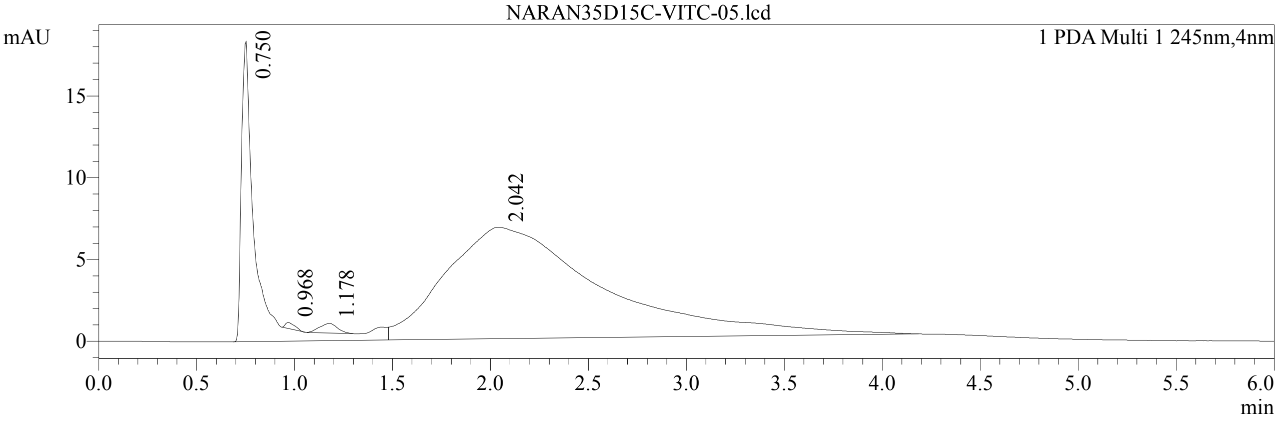
NARAN35D15C-VITC-05.lcd
mAU
1 PDA Multi 1 245nm,4nm
0.750
0.968
1.178
2.042
min

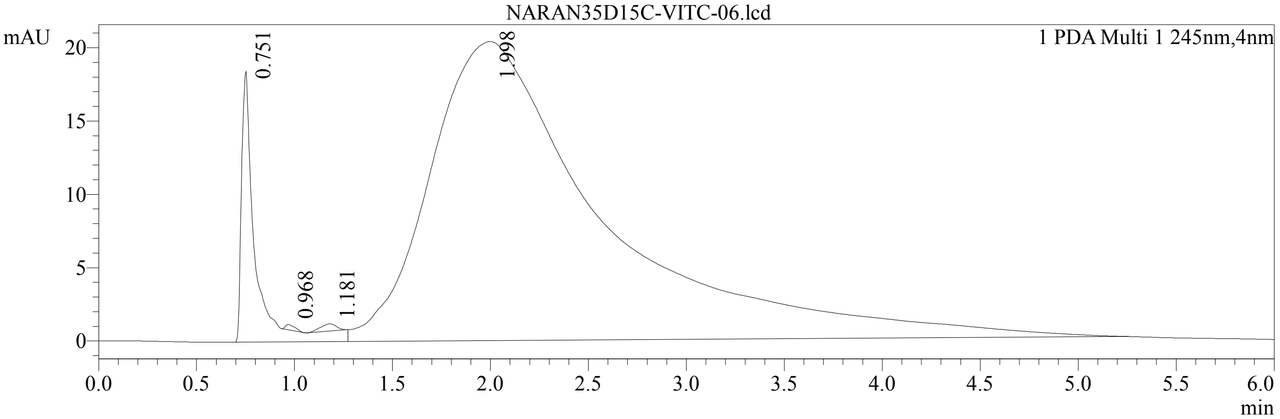
NARAN35D15C-VITC-06.lcd
mAU
1 PDA Multi 1 245nm,4nm
0.751
0.968
1.181
1.998
min

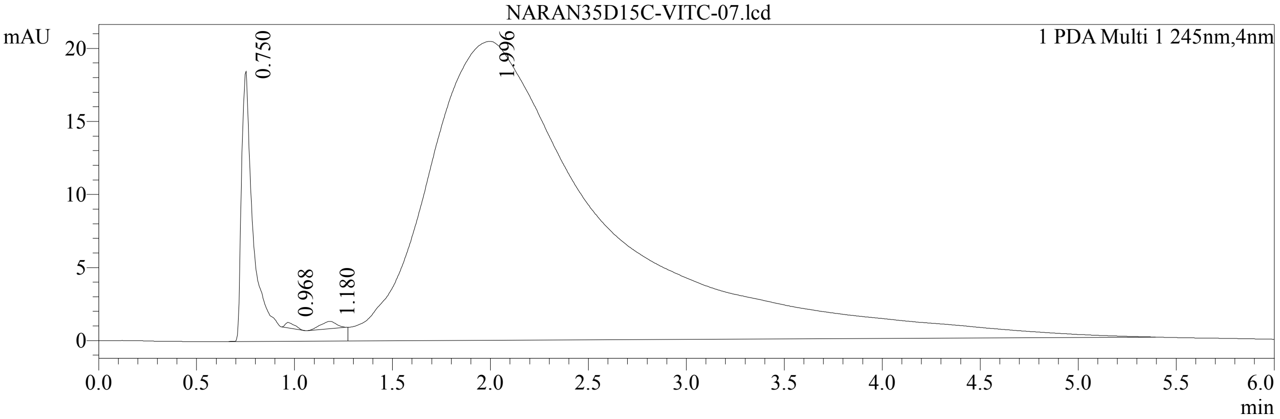
NARAN35D15C-VITC-07.lcd
mAU
1 PDA Multi 1 245nm,4nm
0.750
0.968
1.180
1.996
min

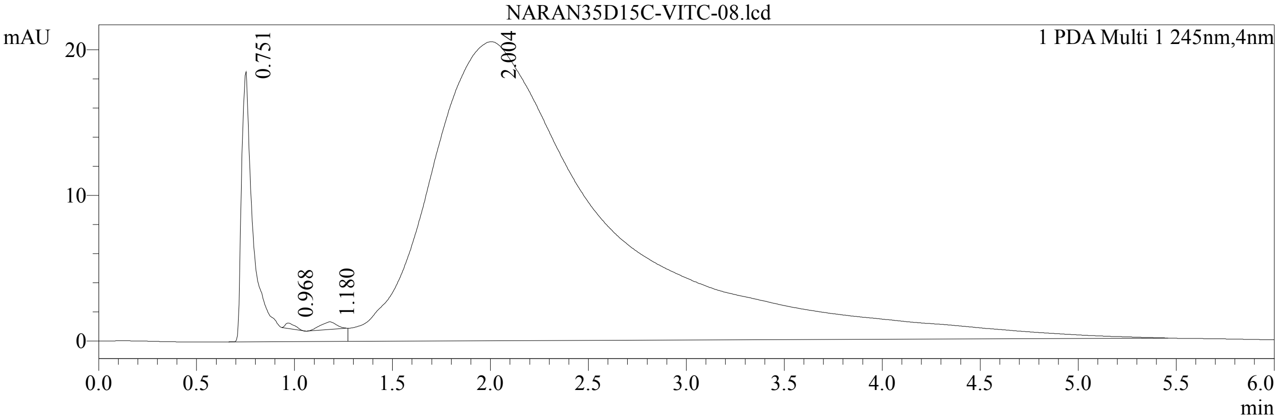
NARAN35D15C-VITC-08.lcd
mAU
1 PDA Multi 1 245nm,4nm
0.751
0.968
1.180
2.004
min

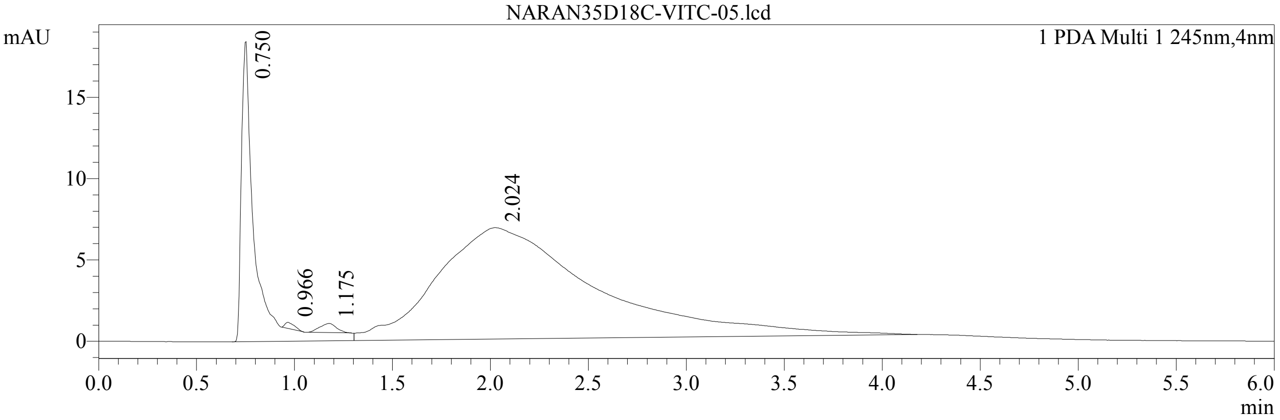
NARAN35D18C-VITC-05.lcd
mAU
1 PDA Multi 1 245nm,4nm
0.750
0.966
1.175
2.024
min

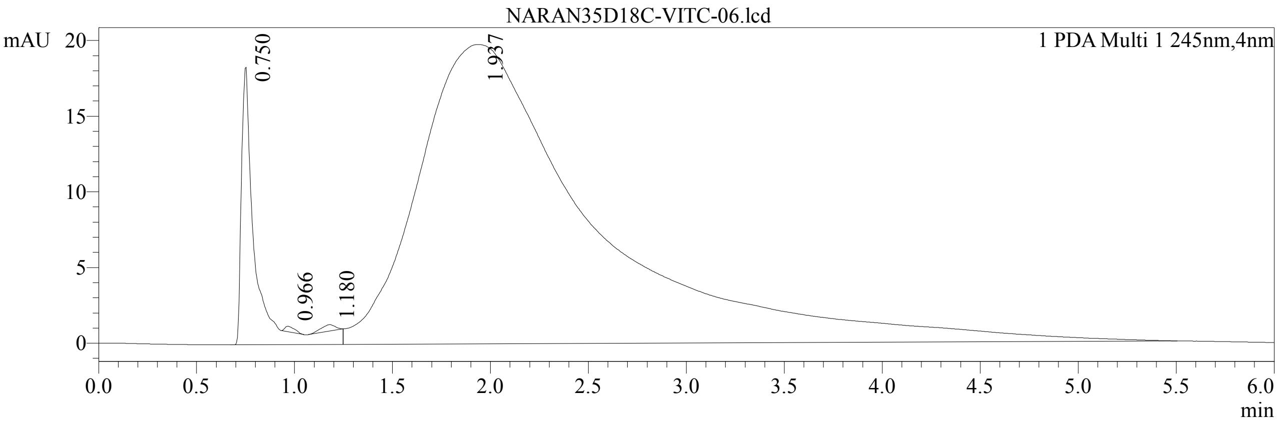
NARAN35D18C-VITC-06.lcd
mAU
1 PDA Multi 1 245nm,4nm
0.750
0.966
1.180
1.937
min

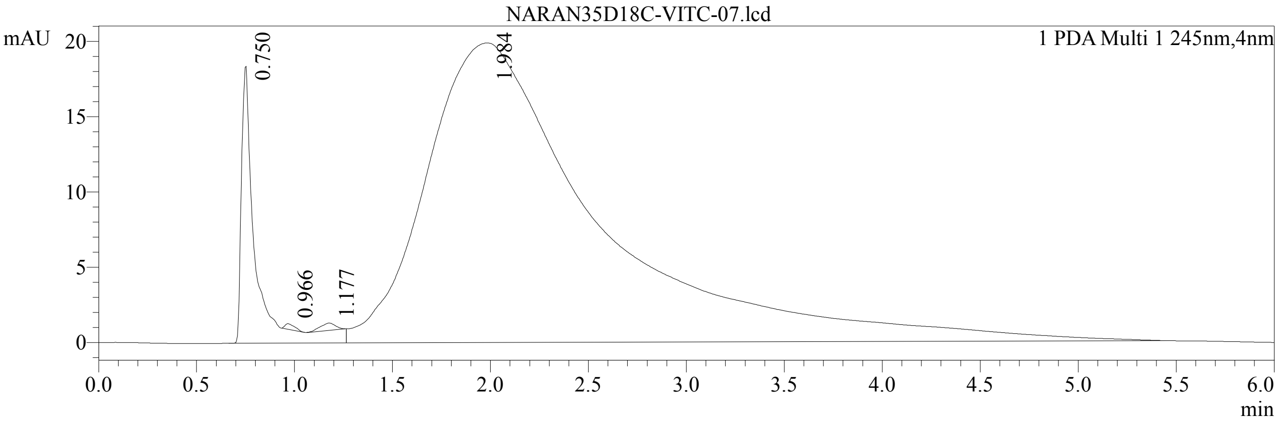
NARAN35D18C-VITC-07.lcd
mAU
1 PDA Multi 1 245nm,4nm
0.750
0.966
1.177
1.984
min

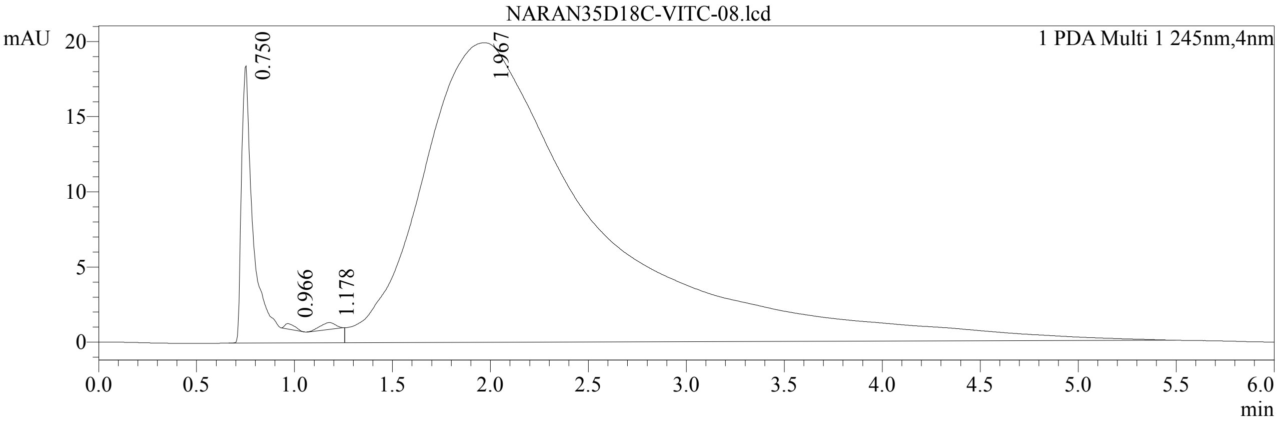
NARAN35D18C-VITC-08.lcd
mAU
1 PDA Multi 1 245nm,4nm
0.750
0.966
1.178
1.967
min

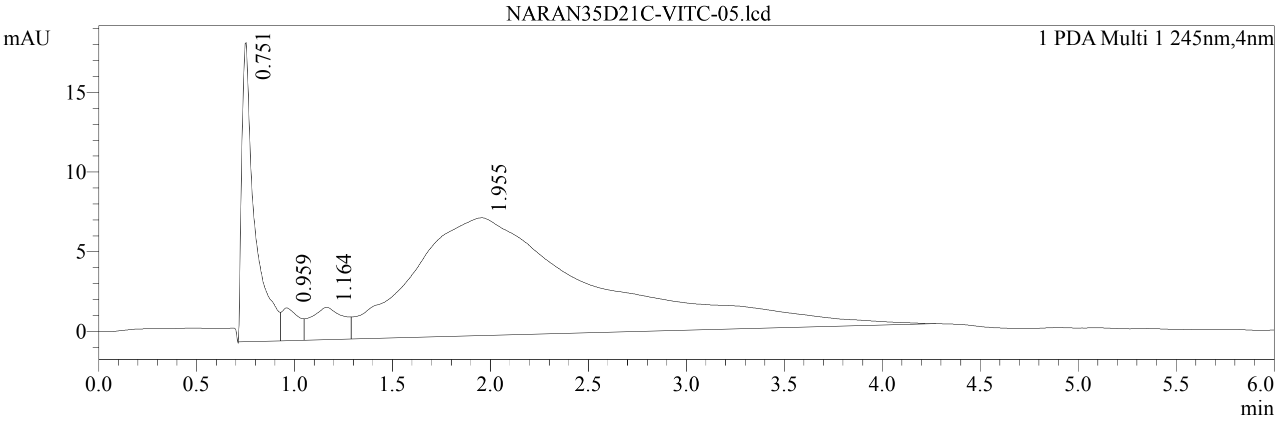
NARAN35D21C-VITC-05.lcd
mAU
1 PDA Multi 1 245nm,4nm
0.751
0.959
1.164
1.955
min

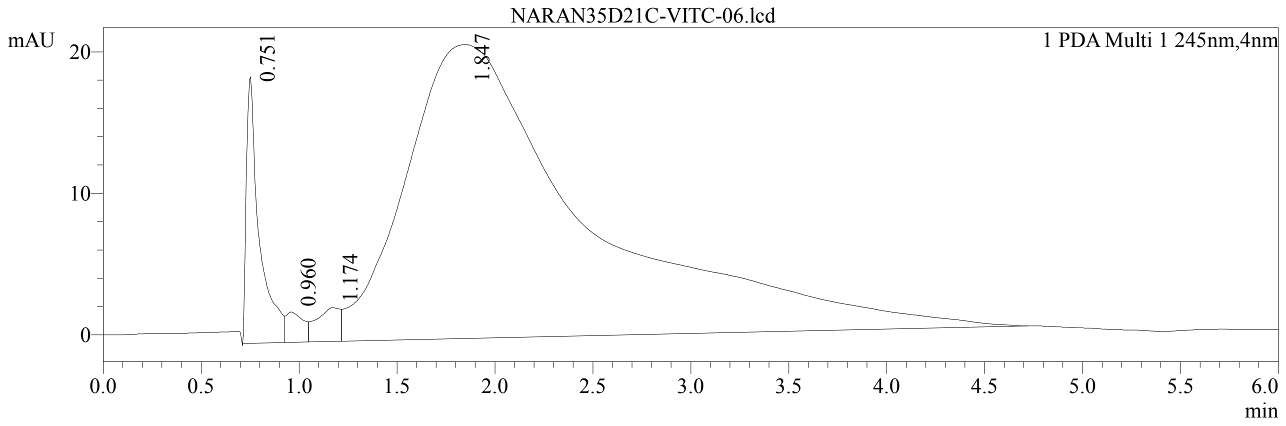

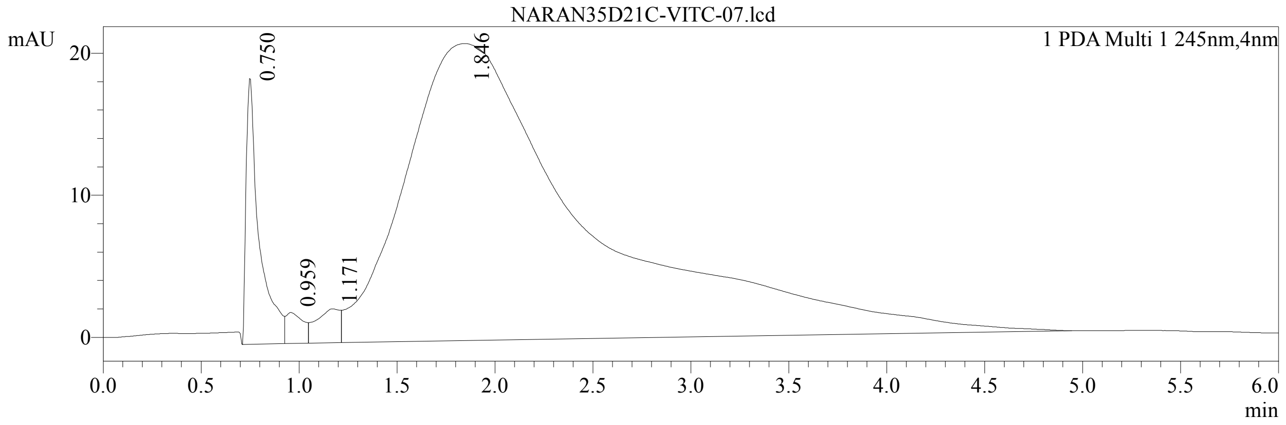

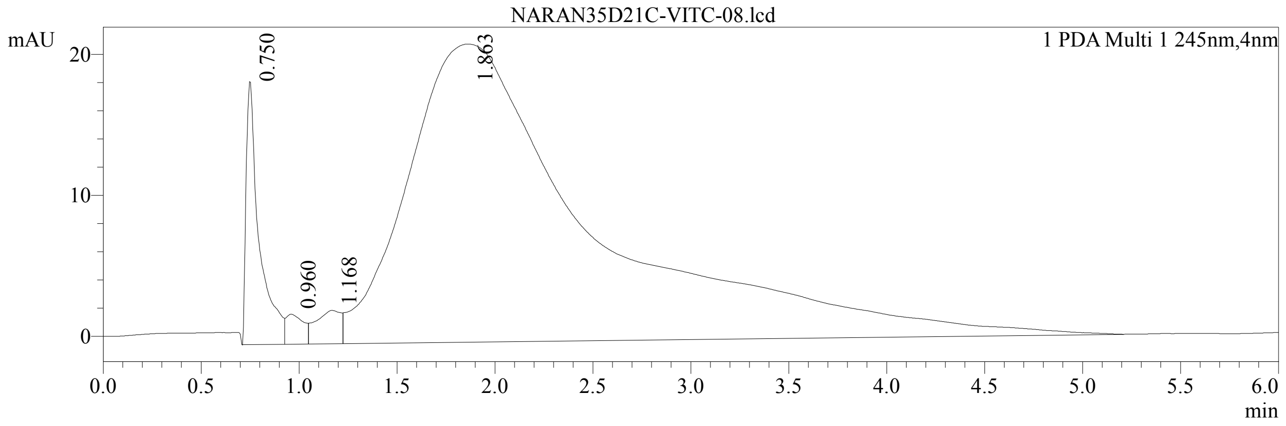

<< PDA >>

Title	Sample Name	Sample ID	VIT C
NARAN55-D1D-VITC-07.lcd	PROT-VITC-07	NARAN55-D1D-VITC-07	50.077
NARAN55-D1D-VITC-08.lcd	PROT-VITC-08	NARAN55-D1D-VITC-08	55.546
NARAN55-D1D-VITC-09.lcd	PROT-VITC-09	NARAN55-D1D-VITC-09	53.539
NARAN45D2C-VITC-01.lcd	PROT-VITC-01	NARAN45D2C-VITC-01	41.748
NARAN45D2C-VITC-02.lcd	PROT-VITC-02	NARAN45D2C-VITC-02	40.675
NARAN45D2C-VITC-03.lcd	PROT-VITC-03	NARAN45D2C-VITC-03	55.185
NARAN45D2C-VITC-04.lcd	PROT-VITC-04	NARAN45D2C-VITC-04	38.654
NARAN55D2C-VITC-06.lcd	PROT-VITC-06	NARAN55D2C-VITC-06	41.807
NARAN55D2C-VITC-07.lcd	PROT-VITC-07	NARAN55D2C-VITC-07	43.696
NARAN55D2C-VITC-08.lcd	PROT-VITC-08	NARAN55D2C-VITC-08	55.330
NARAN55D2C-VITC-05.lcd	PROT-VITC-05	NARAN55D2C-VITC-05	49.457
NARAN35D3C-VITC-01.lcd	PROT-VITC-01	NARAN35D3C-VITC-01	45.142
NARAN35D3C-VITC-02.lcd	PROT-VITC-02	NARAN35D3C-VITC-02	6.401
NARAN35D3C-VITC-03.lcd	PROT-VITC-03	NARAN35D3C-VITC-03	30.073
NARAN35D3C-VITC-04.lcd	PROT-VITC-04	NARAN35D3C-VITC-04	31.123
NARAN55D3C-VITC-05.lcd	PROT-VITC-05	NARAN55D3C-VITC-05	36.471
NARAN55D3C-VITC-06.lcd	PROT-VITC-06	NARAN55D3C-VITC-06	39.564
NARAN55D3C-VITC-07.lcd	PROT-VITC-07	NARAN55D3C-VITC-07	38.002
NARAN55D3C-VITC-08.lcd	PROT-VITC-08	NARAN55D3C-VITC-08	38.708
NARAN45D4C-VITC-01.lcd	PROT-VITC-01	NARAN45D4C-VITC-01	34.355
NARAN45D4C-VITC-02.lcd	PROT-VITC-02	NARAN45D4C-VITC-02	35.801
NARAN45D4C-VITC-03.lcd	PROT-VITC-03	NARAN45D4C-VITC-03	34.888
NARAN45D4C-VITC-04.lcd	PROT-VITC-04	NARAN45D4C-VITC-04	34.952

Title	Sample Name	Sample ID	VIT C
NARAN55D4C-VITC-05.lcd	PROT-VITC-05	NARAN55D4C-VITC-05	39.716
NARAN55D4C-VITC-06.lcd	PROT-VITC-06	NARAN55D4C-VITC-06	39.744
NARAN55D4C-VITC-07.lcd	PROT-VITC-07	NARAN55D4C-VITC-07	40.029
NARAN55D4C-VITC-08.lcd	PROT-VITC-08	NARAN55D4C-VITC-08	38.813
NARAN55D5C-VITC-05.lcd	PROT-VITC-05	NARAN55D5C-VITC-05	41.744
NARAN55D5C-VITC-06.lcd	PROT-VITC-06	NARAN55D5C-VITC-06	39.522
NARAN55D5C-VITC-07.lcd	PROT-VITC-07	NARAN55D5C-VITC-07	39.242
NARAN55D5C-VITC-08.lcd	PROT-VITC-08	NARAN55D5C-VITC-08	39.268
NARAN35D6C-VITC-01.lcd	PROT-VITC-01	NARAN35D6C-VITC-01	35.253
NARAN35D6C-VITC-02.lcd	PROT-VITC-02	NARAN35D6C-VITC-02	35.946
NARAN35D6C-VITC-03.lcd	PROT-VITC-03	NARAN35D6C-VITC-03	35.585
NARAN35D6C-VITC-04.lcd	PROT-VITC-04	NARAN35D6C-VITC-04	35.802
NARAN45D6C-VITC-05.lcd	PROT-VITC-05	NARAN45D6C-VITC-05	37.483
NARAN45D6C-VITC-06.lcd	PROT-VITC-06	NARAN45D6C-VITC-06	37.704
NARAN45D6C-VITC-07.lcd	PROT-VITC-07	NARAN45D6C-VITC-07	37.929
NARAN45D6C-VITC-08.lcd	PROT-VITC-08	NARAN45D6C-VITC-08	38.001
NARAN55D6C-VITC-09.lcd	PROT-VITC-09	NARAN55D6C-VITC-09	41.917
NARAN55D6C-VITC-10.lcd	PROT-VITC-10	NARAN55D6C-VITC-10	42.481
NARAN55D6C-VITC-11.lcd	PROT-VITC-11	NARAN55D6C-VITC-11	42.113
NARAN55D6C-VITC-12.lcd	PROT-VITC-12	NARAN55D6C-VITC-12	42.184
NARAN55D7C-VITC-09.lcd	PROT-VITC-09	NARAN55D7C-VITC-09	42.517
NARAN55D7C-VITC-10.lcd	PROT-VITC-10	NARAN55D7C-VITC-10	42.470
NARAN55D7C-VITC-11.lcd	PROT-VITC-11	NARAN55D7C-VITC-11	42.755
NARAN55D7C-VITC-12.lcd	PROT-VITC-12	NARAN55D7C-VITC-12	43.140
NARAN45D8C-VITC-05.lcd	PROT-VITC-09	NARAN45D7C-VITC-05	37.230
NARAN45D8C-VITC-06.lcd	PROT-VITC-10	NARAN45D7C-VITC-06	37.529
NARAN45D8C-VITC-07.lcd	PROT-VITC-11	NARAN45D7C-VITC-07	38.089
NARAN45D8C-VITC-08.lcd	PROT-VITC-12	NARAN45D7C-VITC-08	37.861
NARAN35D9C-VITC-05.lcd	PROT-VITC-05	NARAN35D9C-VITC-05	35.668
NARAN35D9C-VITC-06.lcd	PROT-VITC-06	NARAN35D9C-VITC-06	36.181
NARAN35D9C-VITC-07.lcd	PROT-VITC-07	NARAN35D9C-VITC-07	36.958
NARAN35D9C-VITC-08.lcd	PROT-VITC-08	NARAN35D9C-VITC-08	37.273
NARAN45D10C-VITC-05.lcd	PROT-VITC-05	NARAN45D10C-VITC-05	37.891
NARAN45D10C-VITC-06.lcd	PROT-VITC-06	NARAN45D10C-VITC-06	38.332
NARAN45D10C-VITC-07.lcd	PROT-VITC-07	NARAN45D10C-VITC-07	39.507
NARAN45D10C-VITC-08.lcd	PROT-VITC-08	NARAN45D10C-VITC-08	39.761
NARAN35D12C-VITC-09.lcd	PROT-VITC-09	NARAN35D12C-VITC-09	37.079
NARAN35D12C-VITC-10.lcd	PROT-VITC-10	NARAN35D12C-VITC-10	35.998
NARAN35D12C-VITC-11.lcd	PROT-VITC-11	NARAN35D12C-VITC-11	36.769
NARAN35D12C-VITC-12.lcd	PROT-VITC-12	NARAN35D12C-VITC-12	36.835
NARAN45D12C-VITC-13.lcd	PROT-VITC-13	NARAN45D12C-VITC-13	39.119
NARAN45D12C-VITC-14.lcd	PROT-VITC-14	NARAN45D12C-VITC-14	39.152
NARAN45D12C-VITC-15.lcd	PROT-VITC-15	NARAN45D12C-VITC-15	39.041
NARAN45D12C-VITC-16.lcd	PROT-VITC-16	NARAN45D12C-VITC-16	38.903
NARAN45D14C-VITC-05.lcd	PROT-VITC-05	NARAN45D14C-VITC-05	40.729
NARAN45D14C-VITC-06.lcd	PROT-VITC-06	NARAN45D14C-VITC-06	39.415
NARAN45D14C-VITC-07.lcd	PROT-VITC-07	NARAN45D14C-VITC-07	40.452
NARAN45D14C-VITC-08.lcd	PROT-VITC-08	NARAN45D14C-VITC-08	40.706
NARAN35D15C-VITC-05.lcd	PROT-VITC-05	NARAN35D15C-VITC-05	38.206
NARAN35D15C-VITC-06.lcd	PROT-VITC-06	NARAN35D15C-VITC-06	37.060
NARAN35D15C-VITC-07.lcd	PROT-VITC-07	NARAN35D15C-VITC-07	38.256
NARAN35D15C-VITC-08.lcd	PROT-VITC-08	NARAN35D15C-VITC-08	38.131
NARAN35D18C-VITC-05.lcd	PROT-VITC-05	NARAN35D18C-VITC-05	35.892
NARAN35D18C-VITC-06.lcd	PROT-VITC-06	NARAN35D18C-VITC-06	37.053
NARAN35D18C-VITC-07.lcd	PROT-VITC-07	NARAN35D18C-VITC-07	37.795
NARAN35D18C-VITC-08.lcd	PROT-VITC-08	NARAN35D18C-VITC-08	37.865
NARAN35D21C-VITC-05.lcd	PROT-VITC-05	NARAN35D21C-VITC-05	34.636
NARAN35D21C-VITC-06.lcd	PROT-VITC-06	NARAN35D21C-VITC-06	34.853
NARAN35D21C-VITC-07.lcd	PROT-VITC-07	NARAN35D21C-VITC-07	35.051
NARAN35D21C-VITC-08.lcd	PROT-VITC-08	NARAN35D21C-VITC-08	34.820
Average			39.381
%RSD			12.057
Maximum			55.546
Minimum			30.073
Standard Deviation			4.748
